# Systematic Prediction of Antifungal Drug Synergy by Chemogenomic Screening in *Saccharomyces cerevisiae*

**DOI:** 10.3389/ffunb.2021.683414

**Published:** 2021-07-02

**Authors:** Hamid Gaikani, Andrew M. Smith, Anna Y. Lee, Guri Giaever, Corey Nislow

**Affiliations:** ^1^Faculty of Pharmaceutical Sciences, University of British Columbia, Vancouver, BC, Canada; ^2^Department of Chemistry, University of British Columbia, Vancouver, BC, Canada; ^3^Donnelly Centre for Cellular and Biomedical Research, University of Toronto, Toronto, ON, Canada; ^4^Department of Biochemistry and Molecular Biology, University of British Columbia, Vancouver, BC, Canada

**Keywords:** drug synergy, drug combinations, drug–gene interaction, antifungal, chemogenomics

## Abstract

Since the earliest days of using natural remedies, combining therapies for disease treatment has been standard practice. Combination treatments exhibit synergistic effects, broadly defined as a greater-than-additive effect of two or more therapeutic agents. Clinicians often use their experience and expertise to tailor such combinations to maximize the therapeutic effect. Although understanding and predicting biophysical underpinnings of synergy have benefitted from high-throughput screening and computational studies, one challenge is how to best design and analyze the results of synergy studies, especially because the number of possible combinations to test quickly becomes unmanageable. Nevertheless, the benefits of such studies are clear—by combining multiple drugs in the treatment of infectious disease and cancer, for instance, one can lessen host toxicity and simultaneously reduce the likelihood of resistance to treatment. This study introduces a new approach to characterize drug synergy, in which we extend the widely validated chemogenomic HIP–HOP assay to drug combinations; this assay involves parallel screening of comprehensive collections of barcoded deletion mutants. We identify a class of “combination-specific sensitive strains” that introduces mechanisms for the synergies we observe and further suggest focused follow-up studies.

## Introduction

Drugs and drug-like molecules are powerful molecular tools that can act by rapid and reversible inhibition of a specific protein or other biomolecule in cells. Such chemical perturbations, while similar to genetic manipulations, have several experimental advantages: they are tunable, fast-acting, often reversible, and applicable across large evolutionary distances, e.g., from yeast to human. Drugs can be easily combined to simultaneously modulate multiple proteins' activities, and in fact, the modulation of gene products by administering a combination of drugs can be vital for a successful course of treatment (Keith et al., 2005). The clinical success of chemical combination therapies has motivated our empirical study of synergistic chemical interactions. These data can then be assessed to predict how two drugs might interact in a biological system. To study the potential interactions, several mathematical models of drug synergy are available (Loewe, 1928; Bliss, 1939; Lehár et al., 2007); two widely used approaches are the Bliss model of independence (Bliss, 1939) and the Loewe additivity model (Loewe, 1928, 1957). Neither model is able to explain all drug synergies, and no mathematical model is suited for all observed chemical interactions, indicating the complexity of the problem.

Invasive fungal infections (IFI) are lethal threats to human health, and they cause almost two million worldwide deaths annually. In 2018, the death rate among patients suffering from IFI was reported to be 28.8% (Webb et al., 2018). At present, the available therapies, particularly for invasive infections, are limited to four categories of antifungal drugs; azoles, polyenes, echinocandins, and 5-flucytosine (Perfect, 2017), and the clinical results from most IFI cases are not optimal. In addition, emerging pathogens resistant to common antifungals (Fairlamb et al., 2016) such as the pan drug-resistant yeast *Candida auris* have spread in health care facilities globally (Meis and Voss, 2019; Zhang et al., 2020). One potential solution to the dearth of effective treatments is to explore the antifungal efficacy of novel drug combinations, including those prescribed for diverse indications (Livengood et al., 2020). The use of drug combinations gives rise to several opportunities: (1) it has been proven, both empirically and theoretically, that drugs that are synergistic for a particular effect do not tend to show synergy for side effects (Cokol et al., 2011), (2) the dose of individual agents with serious side effects can be reduced in a combination, (3) synergistic antifungal activity increases therapy potency and reduces lengths of treatment, and (4) compared with monotherapy, it minimizes the risk for antifungal resistance (Livengood et al., 2020). Based upon The National Institutes of Health (NIH) reports on ClinicalTrials.gov, as of May 2021, there are ~500 on-going or completed clinical trials involving antifungal drug combinations, but the success rate of such trials has been modest.

Considering the limited number of drugs available for IFI treatment (Hill et al., 2013), we sought to expand upon our strategy to use yeast as a eukaryotic model to screen any drug that inhibits the growth of, or kills yeast—*Saccharomyces cerevisiae* in this study. Even though such drugs may be active against the host itself, our rationale is that using these drugs could lower host-dependent side-effects because each agent in a combination is typically applied in lower doses. Screens using select combinations on *Candida* spp. and *Saccharomyces* have been performed, but in this we work cast a broader, unbiased net (Hill et al., 2013; Shekhar-Guturja et al., 2016). Specifically, in this study, we selected 11 compounds based upon their well-characterized targets in yeast, and among all possible combinations, drug pairs that empirically showed synergy were used in HIP–HOP assays—a validated genome-wide screen based on HaploInsufficiency Profiling (HIP) and HOmozygous Profiling (HOP) to quantify the relative abundance of uniquely tagged yeast deletion strains. Briefly, in the rapid and cost-effective HIP assay, a complete collection of heterozygous deletion strains is pooled, grown in the presence of the compound, and sampled as a function of time. Molecular barcodes incorporated into each strain allow parallel analysis and relative strain abundance to be quantitatively assessed either by hybridization to oligonucleotide arrays, or more recently, by Next-Generation Sequencing Technologies. The result is a list of genes ranked in order of their importance for growth and survival, a quantitative metric termed “fitness.” Strains most sensitive to drugs often carry deletions in genes that encode the drug target. Once the primary mechanism has been identified and confirmed in secondary genetic and/or biochemical assays, further pathway-specific genes that act to buffer the drug target pathway can be uncovered using our HOP assay. This assay identifies the drug effects on the non-essential fraction of the genome and reveals the genes important for buffering the drug target pathway. As described in this study, we modified the HIP–HOP assay to study drug combinations. Using this approach, we were able to: (1) identify numerous synergistic combinations, (2) quantify this synergy and identify combination-specific sensitive strains on a genome-wide scale. This study is intended to generate a new strategy to predict drug synergy by using comprehensive genome-wide screens. None of the drug combinations identified here were actually tested in a fungal pathogen. However, we are hoping that this study will inspire research on relevant fungal pathogens.

## Methods

### Pair-Wise Screening of Drug Combinations

**Figure 1:**
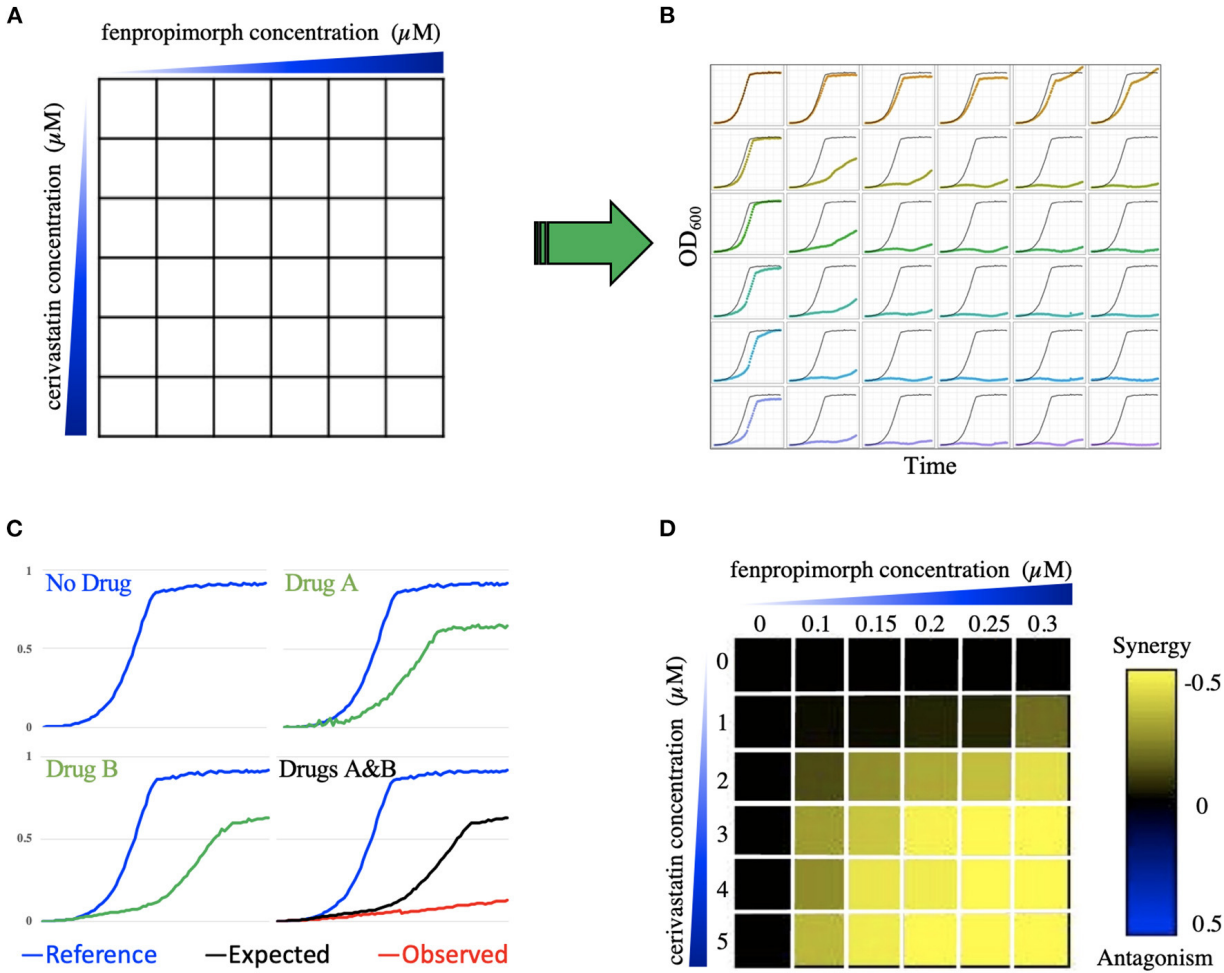
Combination screening and transformation of growth data into a quantitative metric. **(A)** Illustrates the combination of compounds cerivastatin and fenpropimorph as an example. A 6-by-6 dose matrix is screened with an increase of each drug along the x and y axes. The blue triangles represent increasing concentration of drugs. In **(B)** each square in the 6-by-6 matrix is represented by a growth curve for each drug combination, which is optical density (OD_600_) vs. time. Panel **(C)** shows an example of greater than additive inhibition due to synergy. The blue growth curves represent the DMSO (no drug) control. Green curves show growth in the presence of compounds A and B. The combination of A and B is shown in the bottom right-hand cell in which the black line indicates the expected growth rate based on the multiplicative model, while the red curve is the actual cell growth in the drug combination. In the heatmap **(D)**, the color of each square reports the epsilon value generated using the multiplicative model, where black represents no interaction, yellow represents synergy, and blue represents antagonism. In this heatmap, the 6-by-6 dose response matrix of cerivastatin vs. fenpropimorph provides an example of synergy, because epsilon is negative (yellow). W_AB_ represents the relative growth of yeast in the presence of both drug A (cerivastatin) and drug B (fenpropimorph) when compared to a no-drug control (DMSO), W_A_ represents the relative growth of yeast in the presence of drug A when compared to a no drug control. The color-coded scale bar from yellow (synergy) to blue (antagonism) covers the spectrum where W_ab_ < W_a_ × W_b_, to no interaction W_ab_ = W_a_ × W_b_ to antagonism W_ab_ > W_a_ × W_b_.

To identify synergistic combinations, one should determine the effect of both individual agents and drug combinations on yeast growth rate. To accomplish this, we screened the drugs in a checkerboard matrix, in which, along each axis, one of the drugs is added at progressively higher doses. Drug concentrations were selected based on inhibitory concentration (IC), which was determined prior by prescreening the drugs' effects on the wild-type cell growth; concentrations are such that there is an IC0 (no drug), IC2, IC5, IC10, IC20, and IC50 for each drug in the matrix. Hence, each drug pair was screened in a 6-by-6 dose-response curve at the IC-values listed above. All possible pairs of the 11 drugs were screened, resulting in 55 combinations, including self-by-self drug combination. The diploid yeast strain BY4743 (derived from S288C) was used to screen these combinations and was grown in a 96-well microtiter plate at 30°C for 24 h in a TECAN optical density reader. The optical density (OD_600_) was measured at 15-min intervals. A diagram depicts how the combination screening was performed (Figure 1).

### Determination of Synergistic Combinations Based on Growth Curve Analysis

Following growth, data for all growth curves were extracted using AUDIT software (Coutin et al., 2020) as described. First, the curves were smoothed, and the area under the curve was calculated. The area under the curve was then compared to the area of no drug control (AREA_drug_/AREA_control_) to create an inhibition ratio. We then used the Bliss multiplicative model^3^ to calculate epsilon for each dose matrix, ε = Drug AB_ratio_ – (Drug A_ratio_ × Drug B_ratio_). Specifically, we considered “drug epsilon” to be the difference between the actual combined growth and the “expected” from the multiplication of the two single agents. For example, if drug A grows at 90% compared to no drug and drug B grows at 80% compared to no drug, the expected defect would be 90 × 80% (e.g., 72%). If the actual combination grows at 50% compared to no drug then epsilon would be 50–72% = −23% When epsilon is zero, then no interaction is observed; when epsilon is negative, there is a synergy, and positive epsilon denotes antagonism. An antagonistic interaction indicates that one of the drugs buffers the effect of the second agent—Figure 1 illustrates how growth data was transformed into a quantitative trait to determine epsilon.

### Drug–Gene Interaction Screening Using Isogenic Cultures

To determine whether a deletion mutant was hypersensitive to the drug, we had to know the growth rate of both the mutant and wild-type strains with and without drug. We used heterozygous deletion mutants of the known drug-targets (listed in Table 1). The yeast strains BY4743 and corresponding heterozygous mutants were grown, as isogenic cultures, in 96-well microtiter plates, at 30°C for 24 h in a TECAN optical density reader. The optical density (OD_600_) was measured at 15-min intervals. Here, the growth metric average generation time (aka AvgG) was used to assess the fitness of wild-type and mutant strains with and without drug; this metric is comprehensively described in the protocol written by our lab (Proctor et al., 2011). The generation time is calculated from the initial inflection point at the end of log phase until the second inflection point as the culture reaches stationary phase. We normalized each strain's fitness to the wild-type and subtracted any single mutant fitness that was contributed by any particular mutant, i.e., we normalized the various heterozygous mutants' growth to wildtype to take into account any fitness defect that was caused by haploinsufficiency.

**Table 1:** Compounds used and known targets.

**Compound**	**Protein target**	**Method**
cerivastatin	Hmg1 (Bischoff et al., 1997)	HOP
tunicamycin	Alg7 (Barnes et al., 1984)	MSP^*^/HIP
methotrexate	Dfr1 (Huang et al., 1992)	HIP
miconazole	Erg11 (Turi and Loper, 1992; Truan et al., 1994)	HIP
rapamycin	Tor2 (Heitman et al., 1991; Sabatini et al., 1994)	MSP/HIP
cantharidin	Glc7 (Li and Casida, 1992; Honkanen, 1993)	HIP
fenpropimorph	Erg24 (Marcireau et al., 1990; H. Lai et al., 1994)	HIP
latrunculin A	Act1 (Spector et al., 1983; Yarmola et al., 2000)	Resistance mapping
benomyl	Tub1 (Davidse and Flach, 1977; Sheir-Neiss et al., 1978)	HIP and mutant mapping
sodium fluoride	Ipp1 (Yan et al., 2008)	HIP
hydroxyurea	Rnr1 (Elledge and Davis, 1990; Tai et al., 2017)	HIP and resistance mapping

* *Multicopy suppression profiling (target overexpression)*.

### Predicting Synergistic Combinations via Chemogenomic Interactions

Following up on how drug–drug interactions predict drug–gene interactions – to predict synergy using chemogenomic data, we examined 18 datasets (see Table 2) and assessed if the known drug-targets listed in Table 1 were sensitive in any of the treatments based on the log_2_ ratio of control over treatment. We then identified combinations available in our laboratory for testing; 25 combinations were selected based on the availability of compounds present in the Giaever/Nislow laboratory as well as Boone lab, at the University of Toronto. To determine if this method can successfully predict synergistic combinations, the chances of observing synergy between randomly paired compounds need to be known.

**Table 2:** Hundred and ten fitness scores of heterozygous mutants in drugs.

	**Miconazole**	**Benomyl**	**Rapamycin**	**Fenpropimorph**	**Cantharidin**	**Sodium fluoride**	**Hydroxyurea**	**Methotrexate**	**Cerivastatin**	**Tunicamycin**	**Latrunculin A**
	**200 nM**	**0.02 μg/ml**	**6 nM**	**0.04%**	**250 μM**	**40 mM**	**80 mM**	**450 μM**	**2 μM**	**0.2 μM**	**0.5 μM**
erg11	**0.82**	**0.76**	0.94	0.54	0.95	1.00	0.92	0.99	**0.90**	0.99	**0.90**
tub1	**0.72**	**0.81**	**0.90**	0.42	0.69	**0.87**	**0.78**	**0.83**	**0.81**	**0.82**	0.35
tor2	0.53	**0.73**	**0.56**	0.68	**0.90**	0.98	**0.85**	0.96	**0.87**	**0.89**	0.65
erg24	0.55	**0.74**	**0.71**	**0.73**	**0.88**	**0.89**	**0.83**	**0.90**	**0.77**	**0.87**	0.68
glc7	0.52	**0.79**	0.65	0.50	**0.58**	0.95	**0.83**	**0.90**	**0.88**	**0.83**	0.68
ipp1	0.61	**0.74**	**0.72**	**0.78**	0.91	**0.71**	**0.89**	0.97	0.99	**0.89**	0.68
rnr1	0.64	0.92	0.73	0.56	0.93	0.96	**0.87**	0.94	**0.90**	0.93	0.68
dfr1	0.55	0.92	**0.80**	0.68	0.93	1.04	**0.87**	**0.78**	0.97	0.91	**0.75**
hmg1	0.56	1.07	**0.80**	0.68	0.97	1.05	**0.90**	1.01	**0.75**	0.91	**0.87**
alg7	**0.76**	1.05	**0.81**	0.55	0.99	1.05	0.94	1.04	0.96	**0.79**	0.96

*Combinations with bold font passed the cut-off of ≥10% fitness defect. Red denotes expected drug-induced haploinsufficiency (e.g., when considering an ERG11 Het, the expectation is that this strain would grow slower than its wild-type counterpart in miconazole because miconazole targets the ERG11 protein.); yellow cells indicate predicted haploinsufficiency interactions based on synergistic drug combinations (i.e., based on the drug–drug synergy we expect a drug–gene interaction). Numbers in each box represent the average generation time (AvgG) of the mutant's fitness relative to wild type in the drug condition. Bold numbers represent interactions causing ≥10% fitness defect, and interactions causing ≥30% are shown as underlined values*.

### Determination of Background Synergy Rate and Experimental Validation of Predicted Combinations

To define enrichment for synergistic combinations, the chances of observing synergy between randomly paired compound combinations must be known. To address this, 105 combinations in a 4-by-4 dosage matrix were screened. We used a smaller matrix in this experiment to maximize the number of combinations that could be screened in a short time. As a result, six combinations can be screened per 96-well plate per TECAN plate reader, instead of two combinations when screened in a 6-by-6 matrix. The drug concentrations were such that there was an IC0 (no drug), IC10, IC20, and IC50 for each drug in the matrix (Table 2). The yeast strain, BY4743, was used to screen these combinations and was grown in a 96-well microtiter plate at 30°C for 24 h in a TECAN optical density reader. The optical density (OD_600_) was measured at 15-min intervals.

### Pooled Competitive Growth Assays

Two deletion pools, a homozygous deletion pool of 5054 strains representing non-essential genes and a heterozygous pool of 1,194 strains representing genes essential for viability, were thawed and diluted in YPD to an OD_600_ of 0.0625; 700 μl cultures were then grown at 30°C with a chemical inhibitor(s) applied at a dose that produced 10–20% growth inhibition of wild-type. An automated liquid handler robot was used to maintain logarithmic growth of pools by collecting 0.7 OD_600_s of heterozygous pool following 20 generations of growth, and 1.4 OD_600_s of homozygous pool following five generations of growth, for further processing as described below.

### Assessing Fitness of Barcoded Yeast Strains by Barcode Microarray

Except where indicated, pooled assays were performed as described in the protocol by Pierce et al. (2007). Genomic DNA was isolated from cells and barcodes, amplified, and hybridized to barcode microarrays, where each barcode deletion mutant is represented by 10 hybridization signals (the uptag and downtag for each strain are each represented on the array five times; Pierce et al., 2007). Array measurements were quantile normalized such that all tags hybridized with the sample pool had similar distributions. Following normalization, we applied a correction factor to the array data to correct for feature saturation (Pierce et al., 2007) and determined the fitness of each barcoded deletion strain using the average of both barcodes. A Z-Score was calculated based on the average barcodes signal intensity against a control probe sets distribution. Positive fitness defect scores signify a decrease in strain abundance during drug treatment.

### Haploinsufficiency Profiling and HOmozygous Profiling of Synergistic Combinations

A key parameter in performing genome-wide screens in yeast is to determine an appropriate screening dose. This value has been empirically determined to be 10–30% of inhibition of wild type growth (Lee et al., 2014). In practice, when performing synergy screens with two agents, one must eliminate any effects due to the action of a single agent alone. Therefore, we screened each single agent at its IC20, as well as at the dose that was used to generate a combined IC20. We therefore needed data from both the combination and individual agents. For each combination genome-wide assays five screens were performed. Specifically, Agent A at its IC10-30, Agent A the dose used when combined (usually an IC2), along with Agent B at its IC10-30, Agent B at the dose used when combined (usually an IC2). Accordingly, each genome-wide synergy assay comprises five separate screens: (1) the combined screen (A+B), (2, 3) each single agent at an IC10–30 (A and B), (4, 5) each single agent at the doses used in A+B.

### Analysis of Combination Profiles

#### Defining the Sensitivity Score

For each drug treatment, a Z-Score based on the averaged Up and DOWN barcode signal intensity was calculated. Using a Z-Score of >2, we defined a list of sensitive strains in each treatment. Filtering out strains that arose from the single drug treatments, we were able to identify unique, combination-specific sensitive strains. To aid in the analysis of drug combinations, we defined factor ε that is the sensitivity of genes in the combination minus the sum of sensitivity in the single agents—ε = Z-Score_AB_ – (Z-Score_A_ × Z-Score_B_).

#### Clustering of Combination HIP–HOP Profiles Sensitivity Scores

We took raw intensity values from the barcode microarrays and normalized the logged raw intensities using a method called Supervised Normalization of Microarrays (SNM). Supervised Normalization of Microarrays (Mecham et al., 2010) was supplied with batch definitions—each batch as the arrays that have the same chip date. We also supplied this method with array descriptors corresponding to the chemical treatments (i.e., which compounds were used); this is meant to preserve biologically relevant signal. After SNM, we selected either the uptag or downtag for each strain based on the lowest variation coefficient to avoid noisy tags. The logged intensities were then used to compute robust Z-Scores. We used the median and median absolute deviation to calculate the Z-Score, and then clustered strains and chemical treatments separately. The similarity between strains/chemical treatments was based on the Pearson correlation of Z-Scores.

#### Examination of Gene-Ontology Terms

Gene-Ontology has the aim to standardize terms for describing gene products. This vocabulary defines a set of cell terms for which a gene can be annotated to. These annotations cover a vast range from location within the cell to specific cellular functions such as nucleotide excision repair. In this study, we used Gene-Ontology terms with more than five genes and <200 genes. To determine enrichment, we used the sensitivity score. Following ranking each gene sensitive in a specific combination, we used Gene Set Enrichment Analysis (GSEA; Subramanian et al., 2005) to determine enrichment in each category.

#### Visualization of HIP–HOP Screens

To facilitate the visualization of the single agent and combination screens, we provide a custom shiny app to (1) upload excel or text files, (2) visualize each screen as a scatterplot (HIP and HOP plots side-by-side), (3) rapidly identify combination specific strains (red), significantly sensitive strains (green), and non-significantly sensitive strains (violet), and (4) the significant strains are detailed below the plots with hyperlinks. **Figure 4** provides a static view of the output of this app which can be accessed here: https://ggshiny.shinyapps.io/GOappCN/

Additional features allow for customization, including selecting axis limits, label font sized, thresholding for significance, and sizing of data points.

## Results

### Synergy Screens

Using our database of 3,200 drugs and drug-like molecules (Lee et al., 2014) we selected 11 compounds (Table 1) and screened all possible combinations of these drugs in a 6-by-6 dosage matrix using growth curve analysis (Figure 1). The growth data were analyzed, using a computer program (AUDIT; Coutin et al., 2020) that converts raw absorbance values into growth curves. Next, to determine potential synergy, corresponding growth curve data were examined to produce the average epsilon score for the drug combination 6-by-6 matrix (defined as AvgS) from which we generated heatmaps (Figure 1D; drug screens, heatmaps, and MATLAB code are available in Supplementary Data Files 1 and 2). If two individual drugs act independently, their effects are expected to combine multiplicatively. In other words, if a drug affecting gene *x* causes a fitness effect *W*_*x*_, and a drug affecting gene y causes a fitness effect *W*_*y*_, then the total effect of the drug combination (*W*_*xy*_) is predicted to be *W*_*x*_ × *W*_*y*_. For our purposes, we measured the deviation epsilon (ε) from this expectation (where ε_*xy*_ = *W*_*xy*_ – *W*_*x*_ × *W*_*y*_) (Onge et al., 2007; Díaz-Mejía et al., 2018). Using this score and a threshold of AvgS < −0.05 to score a combination as synergistic, 33% of all combinations showed synergy. “Large-scale experimental drug synergy screens have found that synergistic drug pairs are rare (4–10%) (Cokol et al., 2011).” Hence, given this unexpectedly high level of synergy, we applied additional filters such as additivity (Loewe, 1928, 1953, 1957), highest single agent, and a potentiation model (Lehár et al., 2007). Finally, we identified 10 combinations that deviated from expectation in all 4 models and were therefore classified as synergistic. Interestingly, fenpropimorph vs. miconazole, fenpropimorph vs. cerivastatin, and miconazole vs. cerivastatin all possessed a strong synergy when combined. These are the only compound pairs that target the same pathway—consistent with the idea that drugs targeting the same essential pathway can be an effective means to produce synergistic combinations.

### Using Synergistic Drug Combinations to Predict Drug–Gene Interactions

Having identified a high-confidence set of synergistic drug–drug interactions, we tested if these interactions could be recapitulated by combining the relevant drug–gene interactions. Our rationale was that if two drugs were synergistic, a loss-of-function mutation (as exemplified in the heterozygous state) in one of the known drug-targets would confer hypersensitivity to the second compound. In other words, if Drug A targets protein A and Drug B targets protein B to produce synergy, then one would predict that Drug A when combined with a loss-of-function mutant in B, should phenocopy the drug combination (it is worth to mention that loss-of-function screens of heterozygotes assume they are acting as recessive alleles because they do not manifest a growth defect in the absence drug). In our experience, all the heterozygote diploids behave as loss-of-function alleles with respect to their response to drug. Because each deletion allele is grown competitively along with ~6,000 strains that are wildtype at the locus they behave as recessive alleles. To empirically test this prediction, we selected heterozygous deletion mutants of the known drug targets and challenged them with each drug listed in Table 1, and the results were compared to data derived from the combination screens.

Eleven heterozygous deletion mutants—each deleted for one of the known drug-targets—along with a wild type control strain were profiled in each drug, resulting in 121 drug–gene interaction tests (i.e., 11 drugs against 11 heterozygous deletion mutants). The act1 heterozygote displayed a significant fitness defect without drug treatment and was eliminated from further analysis. The remaining 110 drug–gene interactions were examined for drug sensitivity using a cut-off of ≥10% fitness defect (i.e., an inhibitory concentration of 10 or IC10) and identified 76 negative drug–gene interactions (bold numbers in Table 2) among them, 10 are the expected HIP-drug or gene–drug interactions (i.e., a drug-target mutant being sensitive to a drug known to target the product of that locus; red cells in Table 2), while the remaining 66 negative drug–gene interactions were novel. Among the 66 negative drug–gene interactions, 17 of the 18 predicted interactions (yellow cells in Table 2) showed a fitness defect ≥10%, giving a significant enrichment (*p*-value = 0.003) of drug–gene interactions, which are predicted by synergistic combinations. Using a more stringent cut-off of greater than a 30% fitness defect, we found 23 negative drug–gene interactions (underlined numbers in Table 2), 8 of which were from the predicted drug–gene interactions (*p*-value = 0.0002); Table 2. Situations where two loss-of-functions—caused by either mutation or drug inhibition—leads to a more deleterious effect than the fitness reduction expected from the combined loss of individual genes are referred to as negative or aggravating interactions (for instance, synthetic sickness, or synthetic lethality; Beltrao et al., 2010).

### Random Combinations of Compounds Are Rarely Synergistic

**Figure 2:**
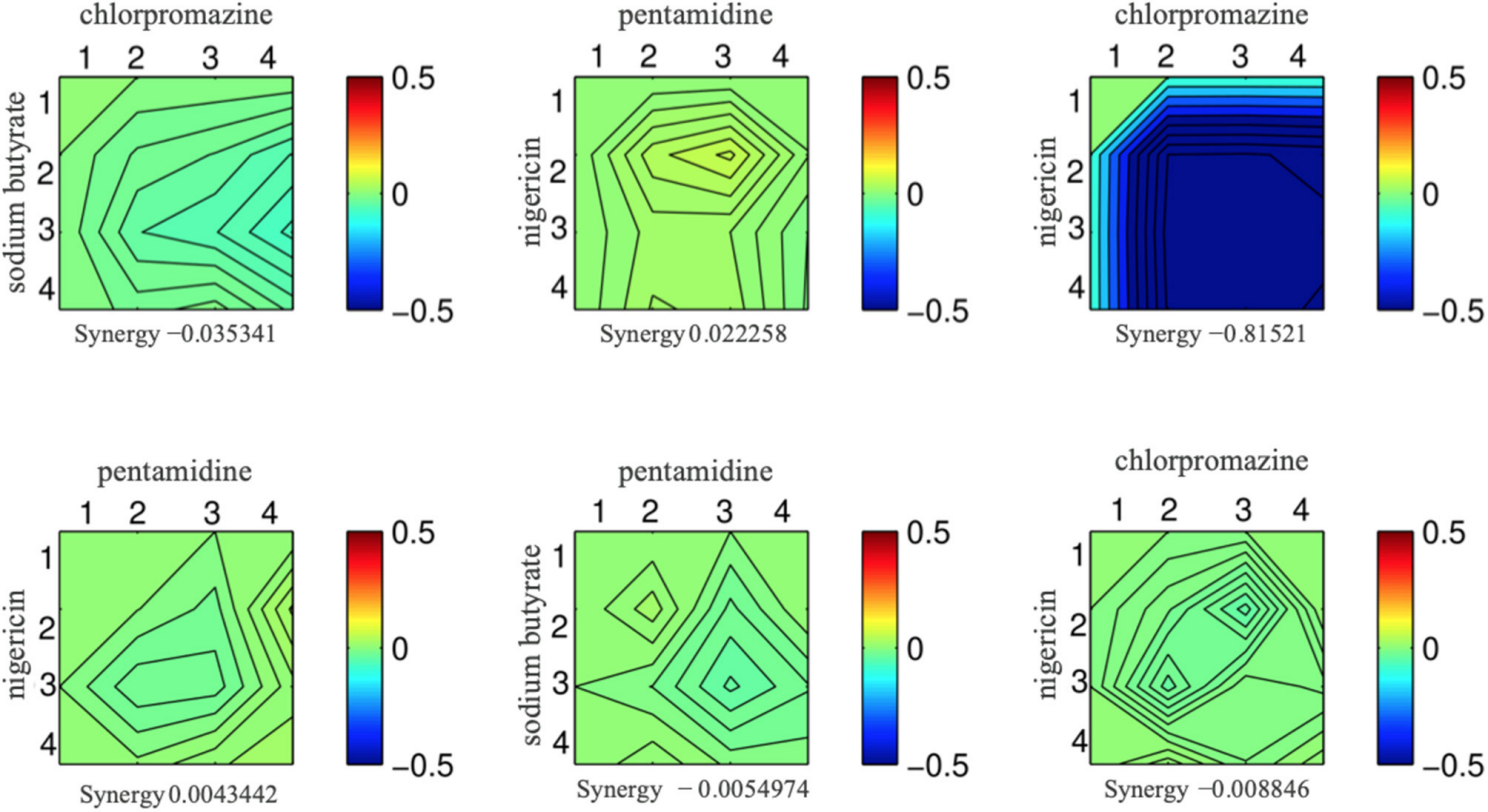
Six examples from the set of drug combinations screened in a 4-by-4 dose matrixes. Each drug is present at 3 doses and a no drug control while the concentration increases along the x and y axis. The color of the heat map represents epsilon generated from the multiplicative model; blue indicates a synergistic interaction, and the epsilon score is presented underneath each combination.

To put our drug-drug synergy observations in context, we sought to determine the chances of observing synergy when two randomly selected compounds were combined. In other words, establishing the likelihood observing synergistic effects of any two compounds would then allow one to calculate any enrichment over random chance. Accordingly, we tested all pairwise combinations of 15 random compounds in a 4-by-4 dosage matrix (Table 3). Our criteria for selecting such “random compounds” included (i) they were bioactive in yeast, and (ii) their HIP–HOP profiles showed a similar number of sensitive strains when compared to the compounds in Table 2. We further evaluated these compounds by mapping them onto the synthetic genetic array (SGA) network of gene–gene interactions (Costanzo et al., 2010). Using the multiplicative synergy model, 17% of these random combinations were synergistic (ε < −0.20) which dropped to 9.5% of combinations when the over-represented compounds that affect the cell wall and secretion were excluded. This value is similar to previously reported combination screening studies, which report ~10% baseline synergy in any combination (Yeh et al., 2006; Lehár et al., 2007; Farha and Brown, 2010). Figure 2 represents an example of synergy prediction screens in a 4-by-4 matrix (all the SGA heatmaps are available in Supplementary Data File 3).

**Table 3:** Drugs and chemical probes and their concentrations used in a 4-by-4 dose matrix to determine baseline synergy.

**Drug**	**Yeast target**	**Dose 1**	**Dose 2**	**Dose 3**	**Dose 4**	**Units**
Cisplatin	DNA	0	100.0	150.0	200.0	μM
MMS	DNA	0	136	318	454	μM
Camptothecin	TOP2	0	250	500	1,000	μM
Caspofungin	FKS1	0	0.007	0.0072	0.0075	μM
Chlorpromazine	Unknown	0	20.0	21.0	22.0	μM
Hygromycin B	Unknown	0	10.0	15.0	20.0	μM
Nocodazole	TUB1	0	11.0	12.0	13.0	μM
Cytochalasin A	ACT1	0	40.0	50.0	60.0	μM
Pentamidine	Unknown	0	75.0	115.0	130.0	μM
Nigericin	Unknown	0	20.0	25.0	30.0	nM
Neomycin sulfate	Unknown	0	175.0	200.0	225.0	μM
Sodium butyrate	HDACs	0	40,000.0	60,000.0	80,000.0	μM
4108395	Unknown	0	9.0	11.0	13.0	μM
2848077	Unknown	0	1.4	1.7	2.0	μM
5962639	Unknown	0	150.0	200.0	250.0	μM

*The three compounds indicated by their PubChem CID are from supplementary database of Lee et al. (2014)*.

### Predicting Synergy

“Chemical space—a term used to encompass all possible small (>500 atoms) organic molecules, including those in biological systems— is vast” (Odling-smee and Dobson, 2004). Furthermore, the current purchasable, and readily screenable compounds comprise approximately 8 million unique compounds (Chuprina et al., 2010). Screening each of these molecules as single agents is quite daunting, while screening all pairwise combinations is impossible. The number of potential combinations is (*N*^2^ – *N*)/2, with the number of starting compounds being (*N*). To expedite the identification of synergistic combinations, we test if we could uncover synergistic combinations from combination chemogenomic data.

**Figure 3:**
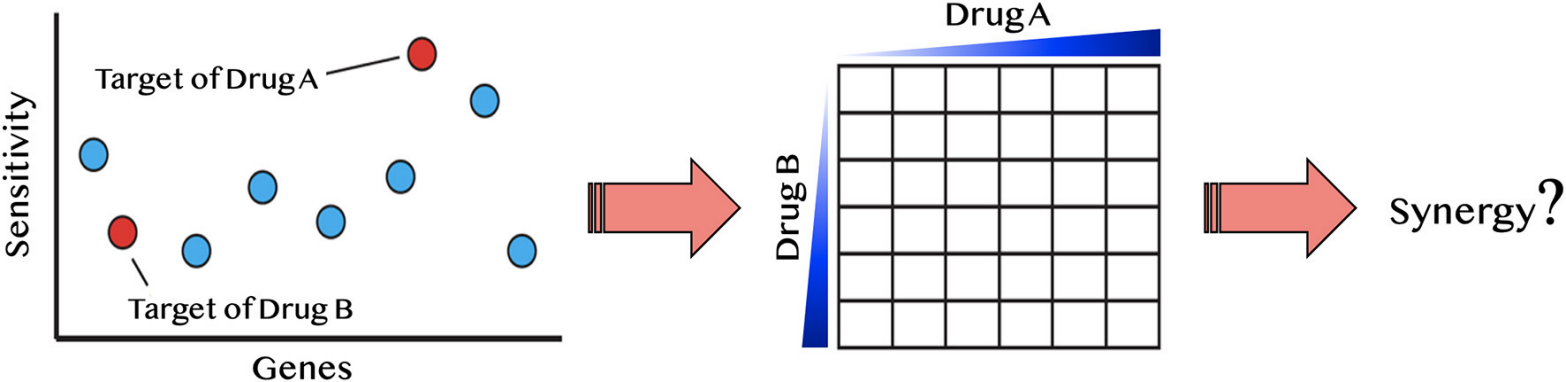
Diagram of synergy prediction method. The HIP–HOP profile for drug A, in which red circles are essential genes and blue are nonessential genes. Genes are listed on the x-axis and sensitivity to drug A is on the y-axis. The most sensitive strain in this example is the target of drug A. However, another essential gene also displays sensitivity to drug A. This gene is the known drug-target of drug B. Using the synergy prediction method, drug A and drug B would be predicted to be synergistic. By testing them in a drug dosage matrix, one can determine if this assumption is true.

We reasoned that if a drug induces a fitness defect in a particular gene-deletion mutant, but does not directly inhibit that gene product, then this drug might be synergistic when combined with a second compound that does inhibit that gene product. To survey the possible drugs and mutants that satisfy these criteria, we first used our lab database of several thousand chemogenomic assays (Lee et al., 2014) to define when a heterozygous deletion mutant of a known drug-target is sensitive. We then sought to uncover synergistic interactions so the drug can be paired with a second drug that inhibits the known drug-target (Figure 3).

This principle is illustrated with miconazole, an antifungal that targets the enzyme Erg11. Because the *hmg1*Δ deletion strain is also sensitive to miconazole, we hypothesized that the combination of miconazole and an HMG1 inhibitor (e.g., cerivastatin), would be synergistic. To test this hypothesis, we first examined the dataset in reference (Hillenmeyer et al., 2008) and all single-agent screens performed in our lab to ask if any of the drug targets in Table 1 exhibited a fitness defect (Supplementary Data File 4). From this survey, 26 predicted synergistic combinations were selected and empirically tested to determine if their synergy rate was greater than the background synergy rate of 17%. We found ~50% of the tested pairs were synergistic (ε < −0.20): a 3.1-fold significant enrichment over random pairs (*p*-value < 0.01). This approach is conceptually distinct from another synergy prediction method introduced by Jansen et al. (2009) (a bioinformatics-based approach predicting antifungal synergy using chemogenomic profiles to identify compound profiles that have a statistically significant degree of similarity to a fluconazole profile). In contrast, our approach is empirical, asking if a drug, selected based on its drug–gene interactions in HIP–HOP, can induce synergy.

### HIP–HOP Combination Profiles

**Figure 4:**
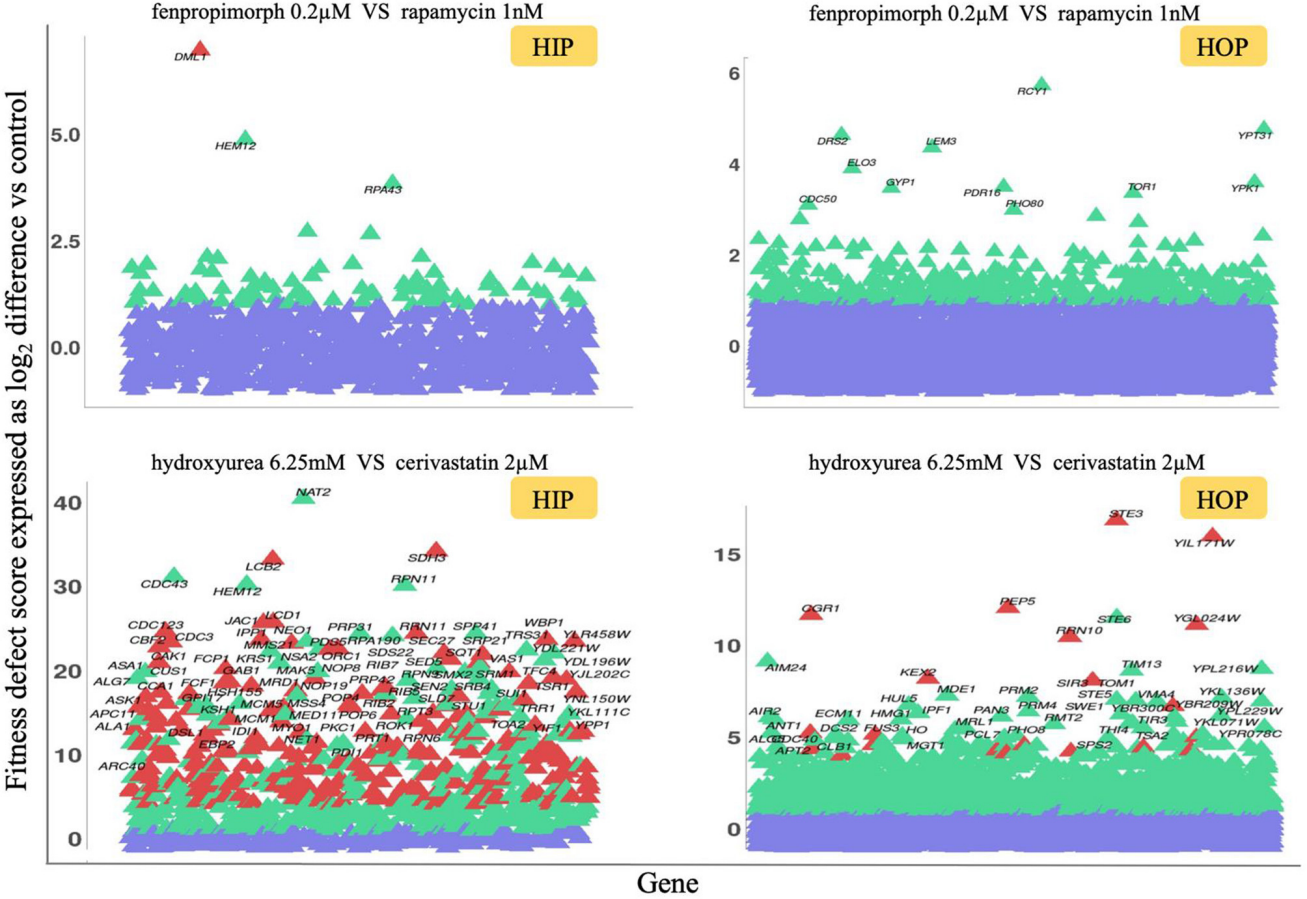
HIP–HOP screening of select drug combinations. For each combination screen, a drug combination that inhibited wild-type yeast growth by 20% was selected and screened alongside each single agent. To identify combination-specific strains, we required that the fitness defect in the combination be 2.0 or greater and that in each single agent, the fitness defect for that strain did not exceed 2.0. For clarity, the heterozygous essential (HIP scatterplots) and homozygous non-essential (HOP scatterplots) data are plotted separately. Significantly sensitive strains are highlighted in green, and combination-specific strains are depicted in red, while violet shows non-significantly sensitive strains. The fitness defect scores are shown on the y-axes. In the case of the fenpropimorph vs. rapamycin combination (top plots), only a single strain, the essential gene *DML1*, was identified as a combination-specific strain. This gene product has been implicated in diverse aspects of mitochondrial function. For the hydroxyurea vs. cerivastatin combination (bottom plots), a larger number of combination-specific strains are apparent. Among these are essential genes involved in sphingolipid biosynthesis (*LCB2*), mitochondrial metabolism (*SDH3, JAC1*) as well as cell cycle checkpoints, and protein degradation at the metaphase anaphase transition (*LCD1, CDC23*, and *CBF2*). Non-essential strains specific to this combination include those involved in response to diverse stresses (*STE3, CGR1*) and targeted protein degradation (*PEP5, KEX2*).

We next used a variation of the HIP–HOP assay to screen some of the newly identified synergistic drug combinations, testing compound combinations genome-wide. Based on these tests, all 14 confirmed synergistic combinations and 12 non-synergistic combinations, and each single agent was used for chemogenomic screening (Supplementary Data File 5). We then used the chemogenomics approach described by Lee et al. (2014) to identify any significantly sensitive strain in all screens. To further scrutinize the drug combinations, we defined the epsilon value (ε) as a fraction of uniquely sensitive genes, in both synergistic and non-synergistic pairs. We defined uniquely sensitive genes as those that are significant only when both compounds are tested together at a fitness defect score of 2.0 or greater. Two examples are shown in Figure 4, and the entire dataset of single agent and combination HIP–HOP profiles which is visualizable in our shiny app is in Supplementary Data File 4 and 5.

Although the primary goal of these genome-wide combinations is to serve as a resource for focused tests of individual combination-specific genes, several high-level observations are noteworthy: (i) combinations vary greatly in the number of specifically sensitive genes, (ii) in some cases the combination-specific strains appear to be subject to potentiation by one of the two agents (i.e., these strains can be detected at higher doses of the single agents (Hoon et al., 2008), and (iii) the combination-specific genes identified are consistent with known mechanisms of actions of one or both of the drugs used in the combination.

**Figure 5:**
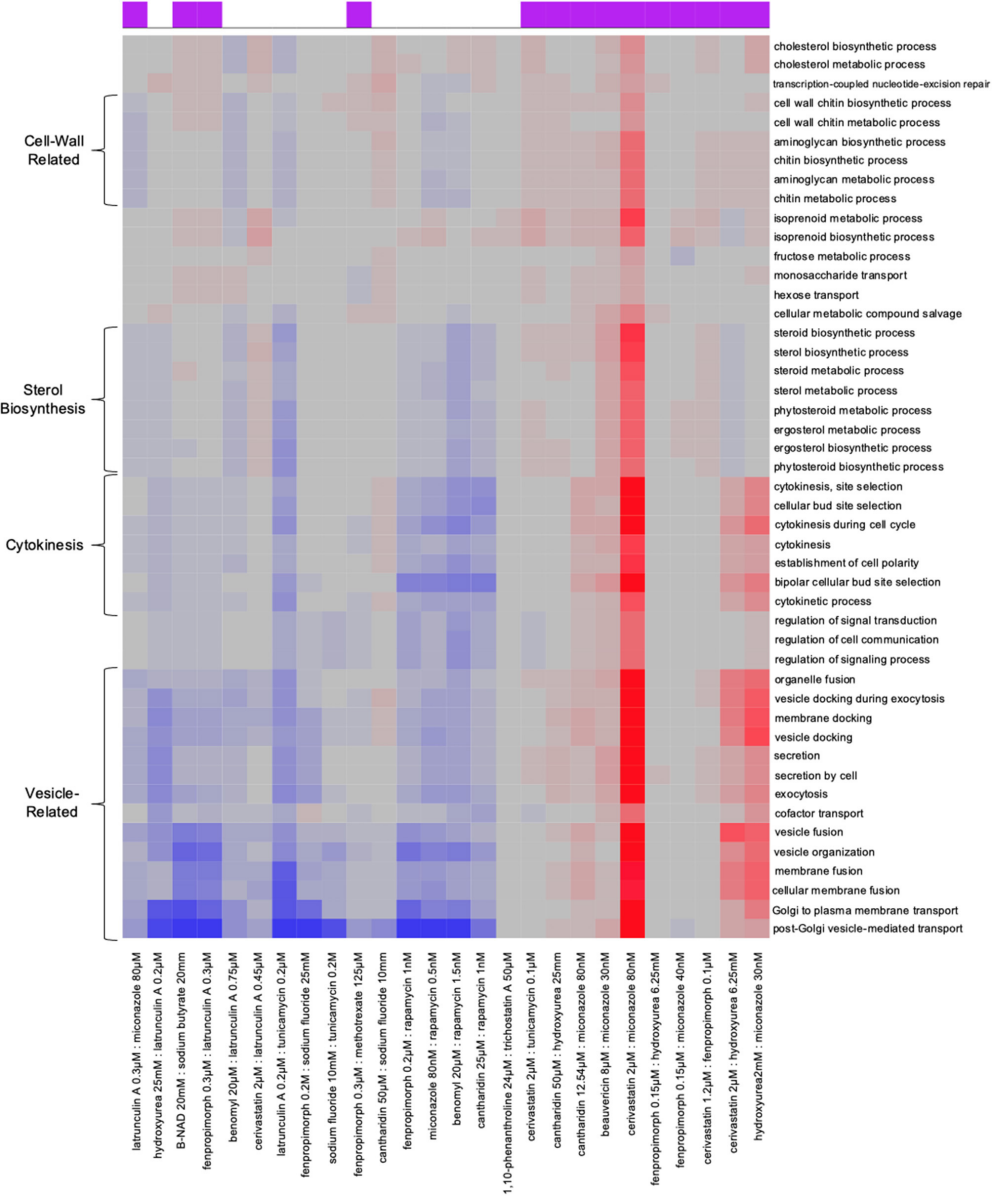
Combination-specific Gene-Ontology (GO) enrichment HIP–HOP data. Examination of Gene-Ontology enrichment using ε score. In the clustered heat map generated from the ε scores, on the x-axis we have each drug combination listed on the bottom; at the top on the x-axis is a purple box which denotes if a combination is synergistic or not. GO terms are denoted on the y-axis. Red shows a particular GO term is highly enriched in the combination, gray denotes no enrichment, and blue shows significant enrichment among genes with low ε scores.

We examined each combination screen, both synergistic and non-synergistic, and examined the biochemical pathways enriched in each pathway. Examining the Gene Ontology (GO) enrichments via the synergy score (Figure 5), we found that each combination provides a unique signature. For instance, the miconazole-cerivastatin combination screen was enriched for gene deletion strains involved in cell wall, cytokinesis, vesicle-related processes, and sterol biosynthesis. In contrast, the miconazole-hydroxyurea screen is enriched for vesicle-related processes and cytokinesis but not for cell wall-related or sterol biosynthesis processes. These combination-enriched GO terms can identify which cellular processes are providing resistance to the combination and could help to understand the mechanism of synergy on a combination-specific basis.

## Discussion

In this study, we used genome-wide chemogenomic profiling to select drug combinations for synergy testing then confirmed our predictions using combined chemogenomic assays. An interesting observation from the drug combination data is that the three inhibitors affecting the ergosterol pathway were highly synergistic when applied in combination, suggesting that compounds that inhibit different points within a pathway are more likely to be synergistic, consistent with (Zimmermann et al., 2007) and the observations by (Cokol et al., 2011) that found similar compounds to be “promiscuously” synergistic. We further demonstrated that, among the synergistic combinations, 78% of the combinations tested (Table 1) were the result of combining an ergosterol inhibitor with a second agent. This indicates that the ergosterol inhibitors are highly synergistic with other agents, which is likely due to their effect on the yeast cell membrane, thereby allowing compounds more effective entry (Farha and Brown, 2010).

Using a modified genome-wide assay we demonstrated that synergistic combinations result in uniquely sensitive strains that are specific to the combinations and are not observed in either of the single agents. Because we used stringent cut-offs, the difference we found between synergistic and non-synergistic combinations likely represents a minimum level of enrichment. We also found that each combination has its own pathway and GO enrichments (Figure 5).

During the course of this work, we confirmed that these drug–drug interactions (derived from drug combination treatment) can be recapitulated using drug–gene interactions by directly assaying loss-of-function (heterozygous deletion) mutants for a drug's known target with a drug that inhibits a synergistic target. We further found that drug–gene interactions derived from synergistic drug–drug interactions were enriched for negative interactions. To extrapolate these observations, we analyzed our single-agent chemogenomic screening data to predict combinations that might exhibit synergy. Given that we observed the baseline level of synergy between 10 and 17%, between 83 and 90% of any random combination should not be synergistic. Our approach reduces the screening required by at least 3.1-fold. This experimental approach involves: (1) using chemogenomic data to identify drugs able to make known drug-targets haploinsufficient, (2) pairing the strain with the expected drug, and (3) screening a dose matrix for synergy. In a pilot of 26 combinations, we identified 14 synergistic combinations. This method is easily adaptable to include new drug-targets, as we limited our search to 11 well-characterized drug-targets in Table 1 and only examined one dataset (Hillenmeyer et al., 2008).

Synergistic effects (between either genes or drugs) have received renewed attention, especially in light of increasingly sophisticated computational approaches and the precise genome engineering possible with CRISPR-based technology. For example, Cokol et al. (2018) developed a computational framework called MAGENTA to investigate the impact of microenvironment on antibiotic combinations, stating that it enables tailoring antibiotic therapies based on the pathogen microenvironment. To predict synergistic or antagonistic interactions on various microenvironments, MAGENTA leverages chemogenomic profiles of both single drugs and metabolic perturbation. They reported several synergistic combinations against *Escherichia coli* and *A. baumannii*, and predicted bactericidal drug-combinations' effectiveness when grown in glycerol media and classified genes in the glycolysis and glyoxylate pathways as top predictors of synergy and antagonism, respectively (Cokol et al., 2018).

In 2016, Wong et al. leveraged combinatorial genetics en masse (CombiGEM) to systematically study gene and drug combinations modulating biological phenotypes (Wong et al., 2016). Combi-GEM allows for the rapid construction of barcoded, combinatorial genetic libraries that can be quantified with high-throughput sequencing. They applied CombiGEM-CRISPR to generate a library of 23,409 barcoded dual guide-RNA (gRNA) combinations, performing a high-throughput pooled screen to find gene pairs that combine to inhibit ovarian cancer cell growth. In the same study, small-molecule drug pairs were tested against the pairwise synthetic lethal hits, revealing that they exert synergistic antiproliferative effects against ovarian cancer cells (Wong et al., 2016).

Combining chemogenomics and genetic interactions, Weinstein et al. (2018) studied antifungal combinations applied to two yeast species, *C. albicans* and *S. cerevisiae*. This study showed that, both synergistic and antagonistic combinations increase the cell-type selectivity of growth-inhibiting drugs. The authors speculate that drug interactions might shift selectivity in comparison to single-drug effects in mixed microbial communities. Indeed, few drugs or drug combinations should be expected to encounter the idealized conditions in laboratory experiments—the variations observed by Weinstein et al. (2018) can change the selectivity of compounds, i.e., inverting, diminishing, or enhancing therapeutic windows.

In a recent CRISPR/Cas9 screen Huang et al. sought to identify genes whose depletion causes synthetic lethality with the broad-acting but not particularly potent Aurora kinase inhibitor VX-680 (Huang et al., 2020). They reported that HCT116 cells showed hypersensitivity to VX-680 when Haspin—a serine/threonine-protein kinase encoded by the GSG2 gene—was either depleted by CRISPR knockout or with Haspin inhibitors, confirming the synergistic effect between VX-680 and Haspin depletion or inhibition (Huang et al., 2020). Recently, Zhou et al. reported a CRISPR-based, multi-gene, knockout screening system for assembly of barcoded, high-order combinatorial guide RNA libraries, en masse. Although combination therapies promise to improve treatment efficiency of various diseases, only a few effective drug combinations—especially those employing three or more drugs (see Table S1 in reference Zhou et al., 2020)—have been introduced so far. Zhou et al. used this approach to systematically identify both pairwise and three-agent synergistic therapeutic target combinations. Their study claimed to uncover double- and triple-combinations that suppressed cancer cell growth and afforded protection against Parkinson's disease-associated toxicity (Zhou et al., 2020).

## Conclusion

In this work, we introduce a strategy to use comprehensive genome-wide screens to first predict compounds that might be synergistic and then test novel combinations empirically. This approach should be extensible to other models and allow for a rational approach to selecting effective drug combinations. Though none of the drug combinations identified here were tested in a fungal pathogen, we hope this study sheds some light on this research field and would inspire other scholars work on relevant fungal pathogens.

## Data Availability Statement

The original contributions presented in the study are included in the article/Supplementary Materials, further inquiries can be directed to the corresponding author/s.

## Author Contributions

CN and AS designed the experiments. AS performed the experiments. CN, HG, and AS wrote the paper. GG supervised the data analysis. All authors analyzed the data, contributed to the article, and approved the submitted version.

## Conflict of Interest

The authors declare that the research was conducted in the absence of any commercial or financial relationships that could be construed as a potential conflict of interest.

## References

[B1] Barnes G. Hansen W. J. Holcomb C. L. Rine J. (1984). Asparagine-linked glycosylation in *Saccharomyces cerevisiae*: genetic analysis of an early step. Mol. Cell. Biol. 4, 2381–2388. 10.1128/MCB.4.11.2381 6096695 PMC369068

[B2] Beltrao P. Cagney G. Krogan N. J. (2010). Quantitative genetic interactions reveal biological modularity. Cell 141, 739–745. 10.1016/j.cell.2010.05.019 20510918 PMC2946632

[B3] Bischoff H. Angerbauer R. Bender J. Bischoff E. Faggiotto A. Petzinna D.. (1997). Cerivastatin: pharmacology of a novel synthetic and highly active HMG-CoA reductase inhibitor. Atherosclerosis 135, 119–130. 10.1016/S0021-9150(97)00188-3 9395280

[B4] Bliss C. I. (1939). The toxicity of poisons applied jointly. Ann. Appl. Biol. 26, 585–615. 10.1111/j.1744-7348.1939.tb06990.x

[B5] Chuprina A. Lukin O. Demoiseaux R. Buzko A. Shivanyuk A. (2010). Drug- and Lead-likeness, target class, and molecular diversity analysis of 7.9 million commercially available organic compounds provided by 29 suppliers. J. Chem. Inf. Model. 50, 470–479. 10.1021/ci900464s 20297844

[B6] Cokol M. Chua H. N. Tasan M. Mutlu B. Weinstein Z. B. Suzuki Y.. (2011). Systematic exploration of synergistic drug pairs. Mol. Syst. Biol. 7:544. 10.1038/msb.2011.71 22068327 PMC3261710

[B7] Cokol M. Li C. Chandrasekaran S. (2018). Chemogenomic model identifies synergistic drug combinations robust to the pathogen microenvironment. PLOS Comput. Biol. 14:e1006677. 10.1371/journal.pcbi.1006677 30596642 PMC6329523

[B8] Costanzo M. Baryshnikova A. Bellay J. Kim Y. Spear E. D. Sevier C. S.. (2010). The genetic landscape of a cell. Science 327, 425–431. 10.1126/science.1180823 20093466 PMC5600254

[B9] Coutin N. P. J. Giaever G. Nislow C. (2020). Interactively AUDIT your growth curves with a suite of R packages. G3 (Bethesda). 10, 933–943. 10.1534/g3.119.400898 31974098 PMC7056965

[B10] Davidse L. C. Flach W. (1977). Differential binding of methyl benzimidazol-2-yl carbamate to fungal tubulin as a mechanism of resistance to this antimitotic agent in mutant strains of *Aspergillus nidulans*. J. Cell Biol. 72, 174–193. 10.1083/jcb.72.1.174 12184 PMC2110979

[B11] Díaz-Mejía J. J. Celaj A. Mellor J. C. Coté A. Balint A. Ho B.. (2018). Mapping DNA damage-dependent genetic interactions in yeast via party mating and barcode fusion genetics. Mol. Syst. Biol. 14, 1–17. 10.15252/msb.20177985 29807908 PMC5974512

[B12] Elledge S. J. Davis R. W. (1990). Two genes differentially regulated in the cell cycle and by DNA-damaging agents encode alternative regulatory subunits of ribonucleotide reductase. Genes Dev. 4, 740–751. 10.1101/gad.4.5.740 2199320

[B13] Fairlamb A. H. Gow N. A. R. Matthews K. R. Waters A. P. (2016). Drug resistance in eukaryotic microorganisms. Nat. Microbiol. 1:16092. 10.1038/nmicrobiol.2016.92 27572976 PMC5215055

[B14] Farha M. A. Brown E. D. (2010). Chemical probes of *Escherichia coli* uncovered through chemical-chemical interaction profiling with compounds of known biological activity. Chem. Biol. 17, 852–862. 10.1016/j.chembiol.2010.06.008 20797614

[B15] Heitman J. Movva N. R. Hall M. N. (1991). Targets for cell cycle arrest by the immunosuppressant rapamycin in yeast. Science 253, 905–909. 10.1126/science.1715094 1715094

[B16] Hill J. A. Ammar R. Torti D. Nislow C. Cowen L. E. (2013). Genetic and genomic architecture of the evolution of resistance to antifungal drug combinations. PLOS Genet. 9:e1003390. 10.1371/journal.pgen.1003390 23593013 PMC3617151

[B17] Hillenmeyer M. E. Fung E. Wildenhain J. Pierce S. E. Hoon S. Lee W.. (2008). The chemical genomic portrait of yeast: uncovering a phenotype for all genes. Science 320, 362–365. 10.1126/science.1150021 18420932 PMC2794835

[B18] Honkanen R. E. (1993). Cantharidin, another natural toxin that inhibits the activity of serine/threonine protein phosphatases types 1 and 2A. FEBS Lett. 330, 283–286. 10.1016/0014-5793(93)80889-3 8397101

[B19] Hoon S. M Smith A. Wallace I. M. Suresh S. Miranda M. Fung E.. (2008). An integrated platform of genomic assays reveals small-molecule bioactivities. Nat. Chem. Biol. 4, 498–506. 10.1038/nchembio.100 18622389

[B20] Huang M. Feng X. Su D. Wang G. Wang C. Tang M.. (2020). Genome-wide CRISPR screen uncovers a synergistic effect of combining Haspin and Aurora kinase B inhibition. Oncogene 39, 4312–4322. 10.1038/s41388-020-1296-2 32300176 PMC7291820

[B21] Huang T. Barclay B. J. Kalman T. I. von Borstel R. C. Hastings P. J. (1992). The phenotype of a dihydrofolate reductase mutant of *Saccharomyces cerevisiae*. Gene 121, 167–171. 10.1016/0378-1119(92)90177-Q 1427091

[B22] Jansen G. Lee A. Y. Epp E. Fredette A. Surprenant J. Harcus D.. (2009). Chemogenomic profiling predicts antifungal synergies. Mol. Syst. Biol. 5, 1–13. 10.1038/msb.2009.95 20029371 PMC2824495

[B23] Keith C. T. Borisy A. A. Stockwell B. R. (2005). Multicomponent therapeutics for networked systems. Nat. Rev. Drug Discov. 4, 71–78. 10.1038/nrd1609 15688074

[B24] Lai M. H. Bard M. Pierson C. A. F. Alexander J. F. Goebl M. Carter G. T.. (1994). The identification of a gene family in the *Saccharomyces cerevisiae* ergosterol biosynthesis pathway. Gene 140, 41–49. 10.1016/0378-1119(94)90728-5 8125337

[B25] Lee A. Y. St.Onge R. P. Proctor M. J. Wallace I. M. Nile A. H. Spagnuolo P. A.. (2014). Mapping the cellular response to small molecules using chemogenomic fitness signatures. Science 344, 208–211. 10.1126/science.1250217 24723613 PMC4254748

[B26] Lehár J. Zimmermann G. R. Krueger A. S. Molnar R. A. Ledell J. T. Heilbut A. M.. (2007). Chemical combination effects predict connectivity in biological systems. Mol. Syst. Biol. 3:80. 10.1038/msb4100116 17332758 PMC1828746

[B27] Li Y. M. Casida J. E. (1992). Cantharidin-binding protein: identification as protein phosphatase 2A. Proc. Natl. Acad. Sci. U.S.A. 89, 11867–11870. 10.1073/pnas.89.24.11867 1334551 PMC50658

[B28] Livengood S. J. Drew R. H. Perfect J. R. (2020). Combination therapy for invasive fungal infections. Curr. Fungal Infect. Rep. 14, 40–49. 10.1007/s12281-020-00369-4

[B29] Loewe S. (1928). Die quantitativen probleme der pharmakologie. Ergebnisse Physiol. 27, 47–187. 10.1007/BF02322290

[B30] Loewe S. (1953). The problem of synergism and antagonism of combined drugs. Arzneimittelforschung 3, 285–290. 13081480

[B31] Loewe S. (1957). Antagonisms and antagonists. Pharmacol. Rev. 9, 237–242 13465303

[B32] Marcireau C. Guilloton M. Karst F. (1990). *In vivo* effects of fenpropimorph on the yeast *Saccharomyces cerevisiae* and determination of the molecular basis of the antifungal property. Antimicrob. Agents Chemother. 34, 989–993. 10.1128/aac.34.6.989 2203312 PMC171744

[B33] Mecham B. H. Nelson P. S. Storey J. D. (2010). Supervised normalization of microarrays. Bioinformatics 26, 1308–1315. 10.1093/bioinformatics/btq118 20363728 PMC2865860

[B34] Meis J. F. Voss A. (2019). *Candida auris* in an intensive care setting. N. Engl. J. Med. 380, 890–891. 10.1056/NEJMc1900112 30811923

[B35] Odling-smee L. Dobson C. M. (2004). Insight chemical space and biology. Nature. 432, 824–828. 10.1038/nature03192 15602547

[B36] Onge R. P. S. Mani R. Oh J. Proctor M. Fung E. Davis R. W.. (2007). Systematic pathway analysis using high-resolution fitness profiling of combinatorial gene deletions. Nat. Genet. 39, 199–206. 10.1038/ng1948 17206143 PMC2716756

[B37] Perfect J. R. (2017). The antifungal pipeline: a reality check. Nat. Rev. Drug Discov. 16, 603–616. 10.1038/nrd.2017.46 28496146 PMC5760994

[B38] Pierce S. E. Davis R. W. Nislow C. Giaever G. (2007). Genome-wide analysis of barcoded *Saccharomyces cerevisiae* gene-deletion mutants in pooled cultures. Nat. Protoc. 2, 2958–2974. 10.1038/nprot.2007.427 18007632

[B39] Proctor M. Urbanus M. L. Fung E. L. Jaramillo D. F. Davis R. W. Nislow C.. (2011). The Automated Cell: Compound and Environment Screening System (ACCESS) for chemogenomic screening, in Yeast Systems Biology: Methods and Protocols, eds Castrillo J. I. Oliver S. G. (Totowa, NJ: Humana Press), 239–269. 10.1007/978-1-61779-173-4_15 21863492

[B40] Sabatini D. M. Erdjument-Bromage H. Lui M. Tempst P. Snyder S. H. (1994). RAFT1: a mammalian protein that binds to FKBP12 in a rapamycin-dependent fashion and is homologous to yeast TORs. Cell 78, 35–43. 10.1016/0092-8674(94)90570-3 7518356

[B41] Sheir-Neiss G. Lai M. H. Morris N. R. (1978). Identification of a gene for beta-tubulin in *Aspergillus nidulans*. Cell 15, 639–647. 10.1016/0092-8674(78)90032-6 363278

[B42] Shekhar-Guturja T. Gunaherath G. M. K. B. Wijeratne E. M. K. Lambert J.-P. Averette A. F. Lee S. C.. (2016). Dual action antifungal small molecule modulates multidrug efflux and TOR signaling. Nat. Chem. Biol. 12, 867–875. 10.1038/nchembio.2165 27571477 PMC5030160

[B43] Spector I. Shochet N. R. Kashman Y. Groweiss A. (1983). Latrunculins: novel marine toxins that disrupt microfilament organization in cultured cells. Science 219, 493–495. 10.1126/science.6681676 6681676

[B44] Subramanian A. Tamayo P. Mootha V. K. Mukherjee S. Ebert B. L. Gillette M. A.. (2005). Gene set enrichment analysis: a knowledge-based approach for interpreting genome-wide expression profiles. Proc. Natl. Acad. Sci. U.S.A. 102, 15545–15550. 10.1073/pnas.0506580102 16199517 PMC1239896

[B45] Tai A. Kamei Y. Mukai Y. (2017). The forkhead-like transcription factor (Fhl1p) maintains yeast replicative lifespan by regulating ribonucleotide reductase 1 (RNR1) gene transcription. Biochem. Biophys. Res. Commun. 488, 218–223. 10.1016/j.bbrc.2017.05.038 28495531

[B46] Truan G. Epinat J.-C. Rougeulle C. Cullin C. Pompon D. (1994). Cloning and characterization of a yeast cytochrome b5-encoding gene which suppresses ketoconazole hypersensitivity in a NADPH-P-450 reductase-deficient strain. Gene 142, 123–127. 10.1016/0378-1119(94)90366-2 8181746

[B47] Turi T. G. Loper J. C. (1992). Multiple regulatory elements control expression of the gene encoding the *Saccharomyces cerevisiae* cytochrome P450, lanosterol 14 alpha-demethylase (ERG11). J. Biol. Chem. 267, 2046–2056. 1730736

[B48] Webb B. J. Ferraro J. P. Rea S. Kaufusi S. Goodman B. E. Spalding J. (2018). Epidemiology and clinical features of invasive fungal infection in a US Health Care Network. Open Forum Infect. Dis. 5:ofy187. 10.1093/ofid/ofy187 30151412 PMC6104777

[B49] Weinstein Z. B. Kuru N. Kiriakov S. Palmer A. C. Khalil A. S. Clemons P. A.. (2018). Modeling the impact of drug interactions on therapeutic selectivity. Nat. Commun. 9, 1–9. 10.1038/s41467-018-05954-3 30150706 PMC6110842

[B50] Wong A. S. L. Choi G. C. G. Cui C. H. Pregernig G. Milani P. Adam M.. (2016). Multiplexed barcoded CRISPR-Cas9 screening enabled by CombiGEM. Proc. Natl. Acad. Sci. U.S.A. 113, 2544–2549. 10.1073/pnas.1517883113 26864203 PMC4780610

[B51] Yan Z. Costanzo M. Heisler L. E. Paw J. Kaper F. Andrews B. J.. (2008). Yeast Barcoders: a chemogenomic application of a universal donor-strain collection carrying bar-code identifiers. Nat. Methods 5, 719–725. 10.1038/nmeth.1231 18622398

[B52] Yarmola E. G. Somasundaram T. Boring T. A. Spector I. Bubb M. R. (2000). Actin-latrunculin A structure and function. Differential modulation of actin-binding protein function by latrunculin A. J. Biol. Chem. 275, 28120–28127. 10.1074/jbc.M004253200 10859320

[B53] Yeh P. Tschumi A. I. Kishony R. (2006). Functional classification of drugs by properties of their pairwise interactions. Nat. Genet. 38, 489–494. 10.1038/ng1755 16550172

[B54] Zhang F. Zhao M. Braun D. R. Ericksen S. S. Piotrowski J. S. Nelson J.. (2020). A marine microbiome antifungal targets urgent-threat drug-resistant fungi. Science 370, 974–978. 10.1126/science.abd6919 33214279 PMC7756952

[B55] Zhou P. Chan B. K. C. Wan Y. K. Yuen C. T. L. Choi G. C. G. Li X.. (2020). A three-way combinatorial CRISPR screen for analyzing interactions among druggable targets. Cell Rep. 32:108020. 10.1016/j.celrep.2020.108020 32783942

[B56] Zimmermann G. R. Lehár J. Keith C. T. (2007). Multi-target therapeutics: when the whole is greater than the sum of the parts. Drug Discov. Today 12, 34–42. 10.1016/j.drudis.2006.11.008 17198971

