# Supplementary material for: Systematic Prediction of Antifungal Drug Synergy by Chemogenomic Screening in Saccharomyces cerevisiae
Source: Front Fungal Biol. 2021 Jul 2;2:683414. doi: 10.3389/ffunb.2021.683414 (PMC10512392; doi:10.3389/ffunb.2021.683414)
Supplement: Supplementary file 1 [file Data_Sheet_1.ZIP › Supplementary data/Sypplementary_data_2_(Drug_screens_heatmaps & MATLAB Script)/Drug_screens_heatmaps.pdf]

HEAT MAPS

Method

- grew BY4743 O/N culture
- diluted down to OD600 = 0.0625
- put 98 ul in 96 well plate
- added 1 ul of 100X drug A stock
- added 1 ul of 100X drug B stock
- grew for 95 runs

Drugs
Fenpropimorph
Miconazole
Cantharidin
Methotrexate
Benomyl
Hydroxyurea
NaF
Rapamycin
Tunicamycin
Latrunculin A
Cerivastatin

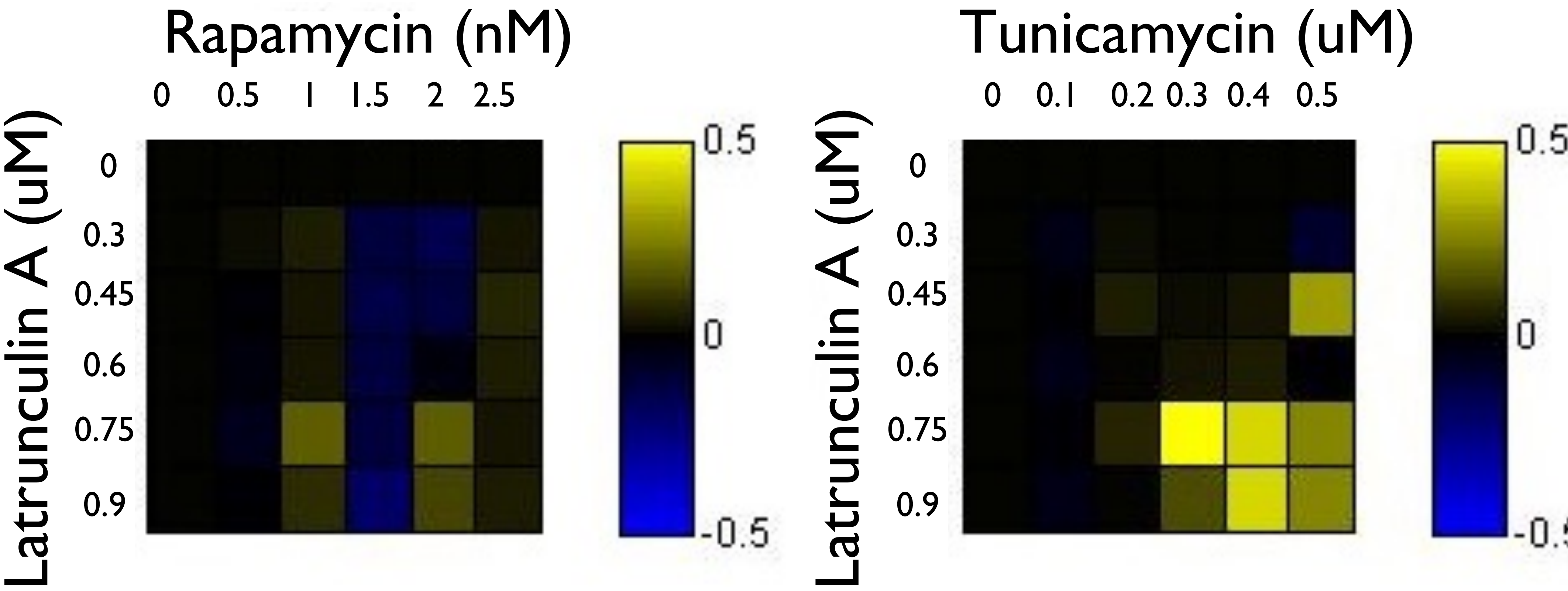
Rapamycin (nM)
0 0.5 1 1.5 2 2.5
Latrunculin A (uM)
0 0.3 0.45 0.6 0.75 0.9
0.5
0
-0.5
Tunicamycin (uM)
0 0.1 0.2 0.3 0.4 0.5
Latrunculin A (uM)
0 0.3 0.45 0.6 0.75 0.9
0.5
0
-0.5

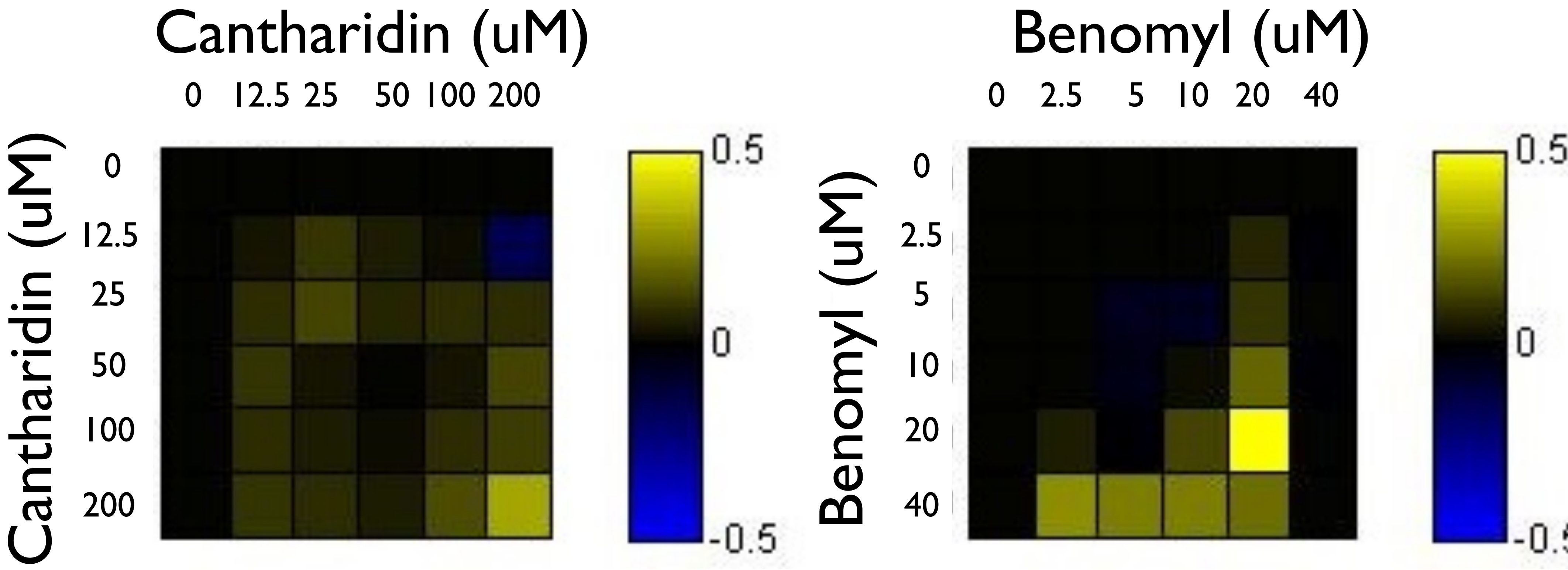
Cantharidin (uM)
0 12.5 25 50 100 200
Cantharidin (uM)
0
12.5
25
50
100
200
0.5
0
-0.5
Benomyl (uM)
0 2.5 5 10 20 40
Benomyl (uM)
0
2.5
5
10
20
40
0.5
0
-0.5

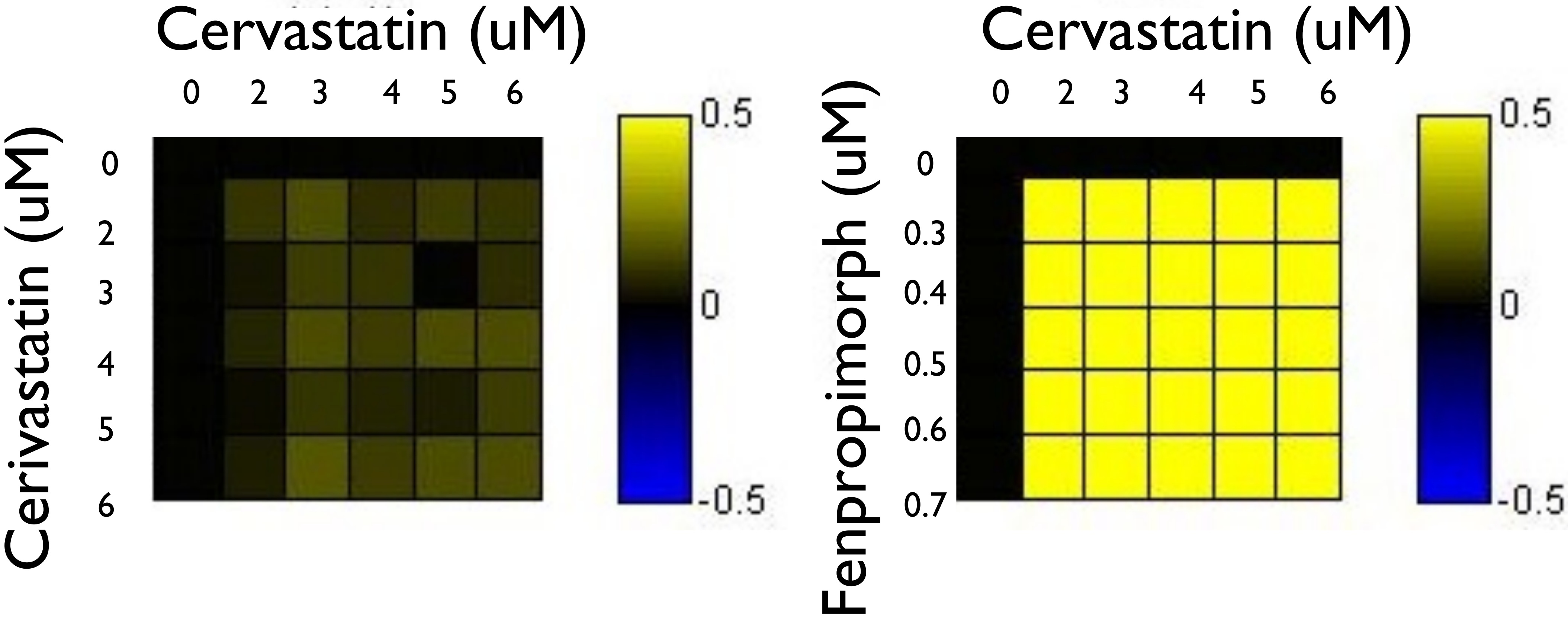
Cervastatin (uM)
0 2 3 4 5 6
Cerivastatin (uM)
0 2 3 4 5 6
0.5
0
-0.5
Cervastatin (uM)
0 2 3 4 5 6
Fenpropimorph (uM)
0 0.3 0.4 0.5 0.6 0.7
0.5
0
-0.5

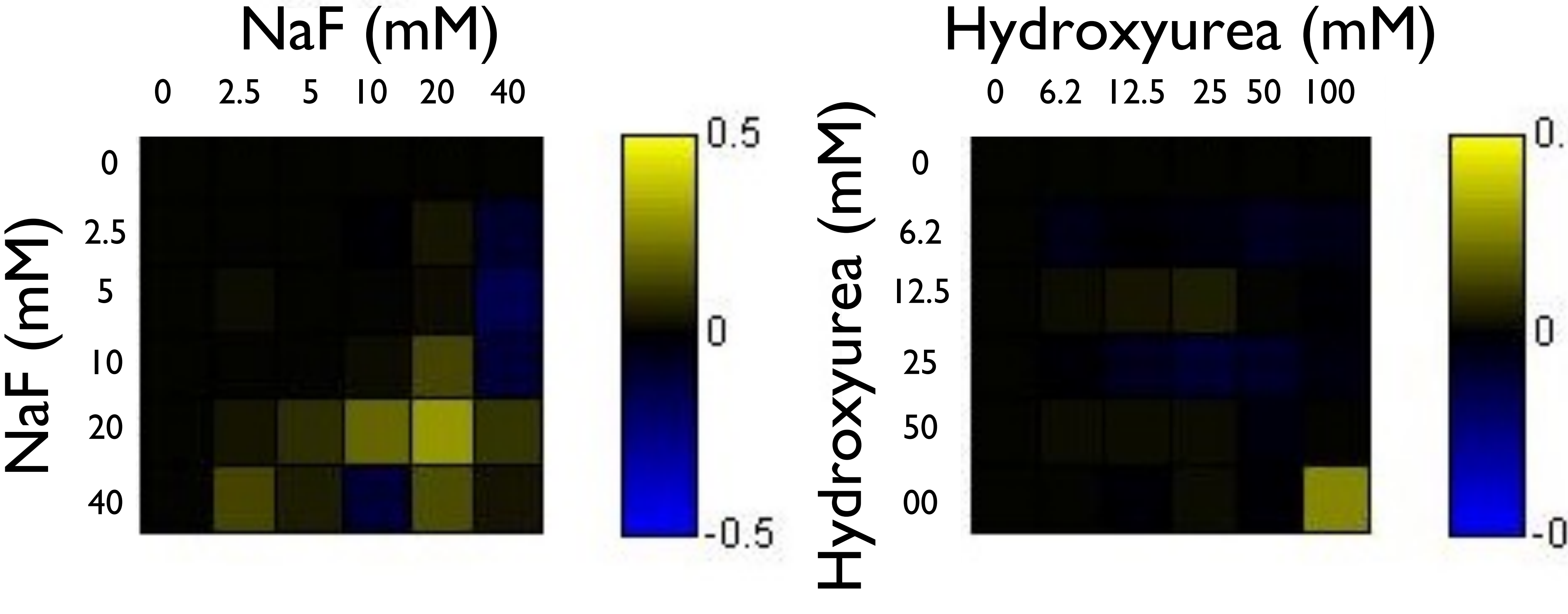
NaF (mM)
0 2.5 5 10 20 40
NaF (mM)
0 2.5 5 10 20 40
0.5
0
-0.5
Hydroxyurea (mM)
0 6.2 12.5 25 50 100
Hydroxyurea (mM)
0 6.2 12.5 25 50 00
0
-0

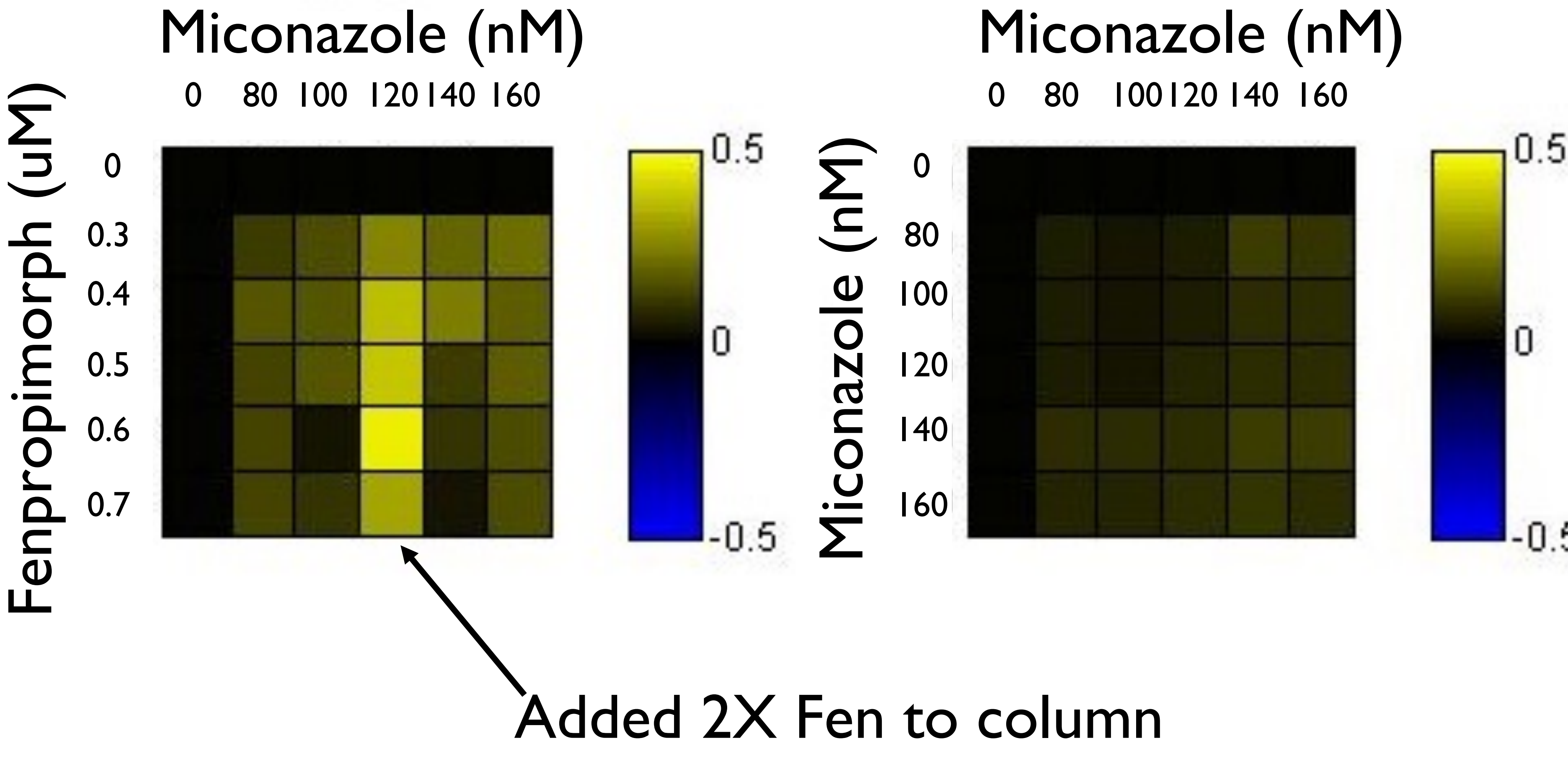
Miconazole (nM)
0 80 100 120 140 160
Fenpropimorph (uM)
0
0.3
0.4
0.5
0.6
0.7
0.5
0
-0.5
Miconazole (nM)
0 80 100 120 140 160
Miconazole (nM)
0
80
100
120
140
160
0.5
0
-0.5
Added 2X Fen to column

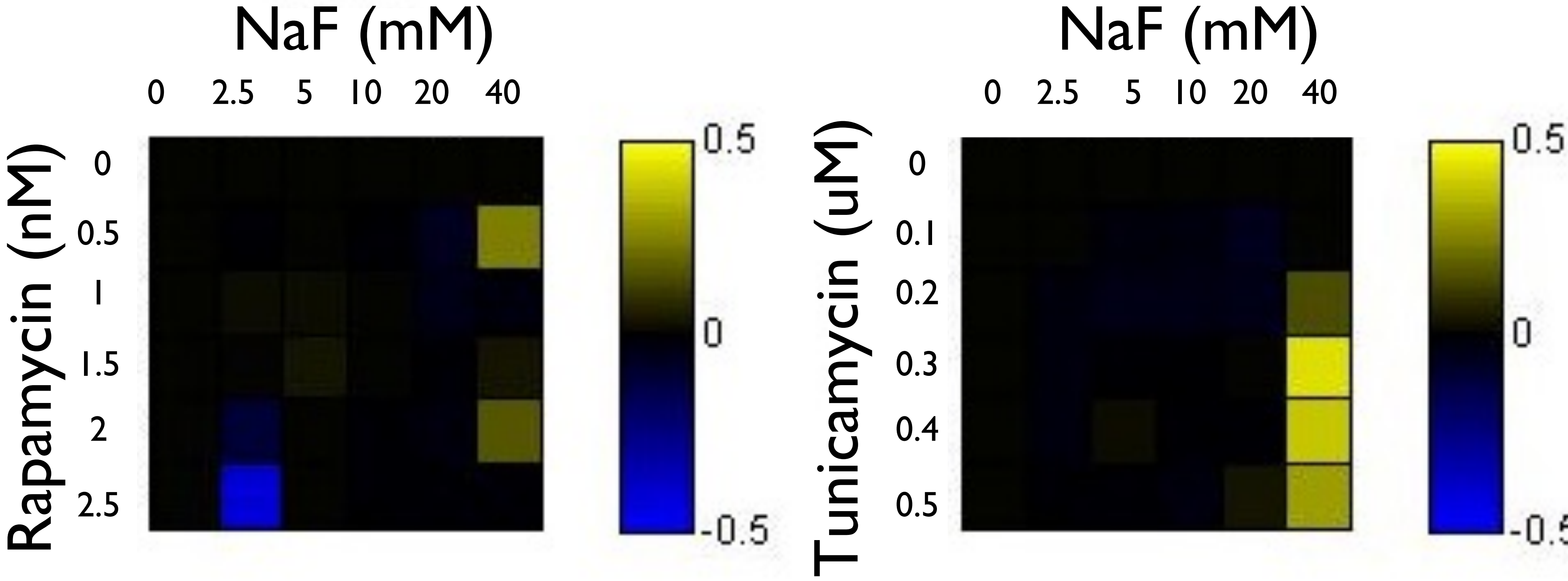
NaF (mM)
0 2.5 5 10 20 40
Rapamycin (nM)
0
0.5
1
1.5
2
2.5
0.5
0
-0.5
NaF (mM)
0 2.5 5 10 20 40
Tunicamycin (uM)
0
0.1
0.2
0.3
0.4
0.5
0.5
0

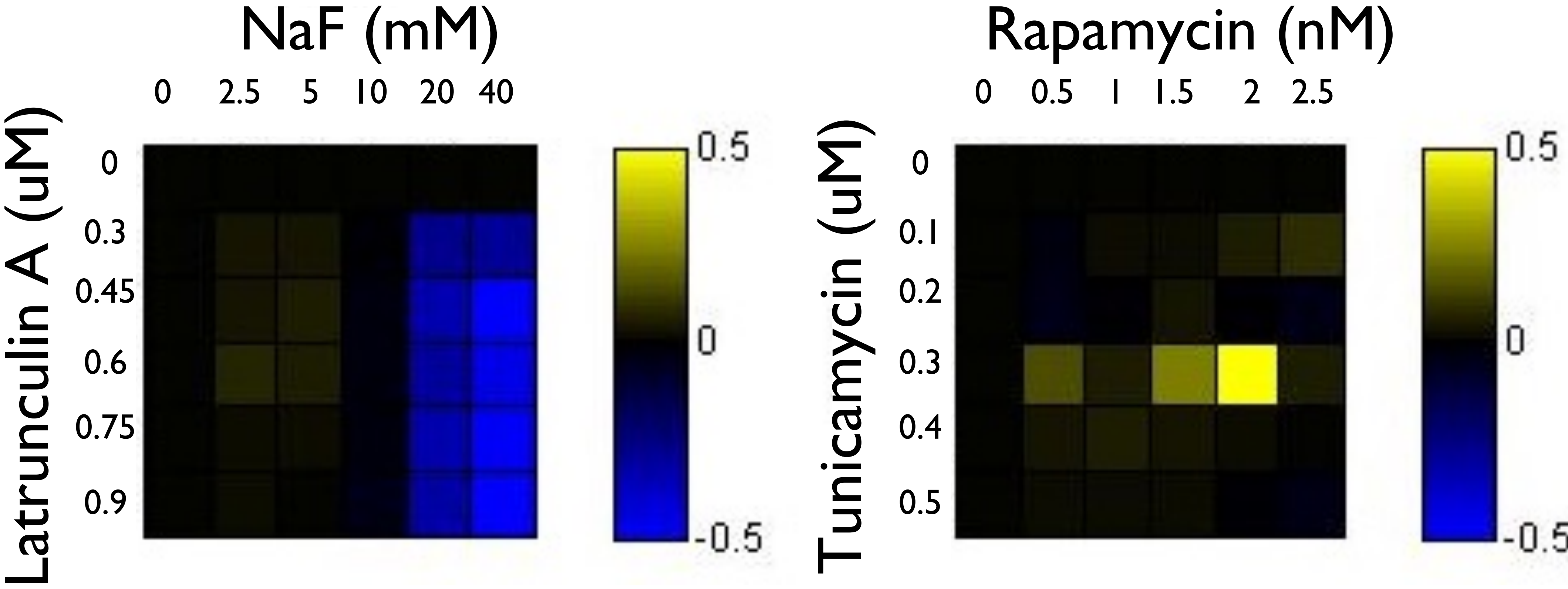
NaF (mM)
0 2.5 5 10 20 40
Latrunculin A (uM)
0
0.3
0.45
0.6
0.75
0.9
0.5
0
-0.5
Rapamycin (nM)
0 0.5 1 1.5 2 2.5
Tunicamycin (uM)
0
0.1
0.2
0.3
0.4
0.5
0.5
0
-0.5

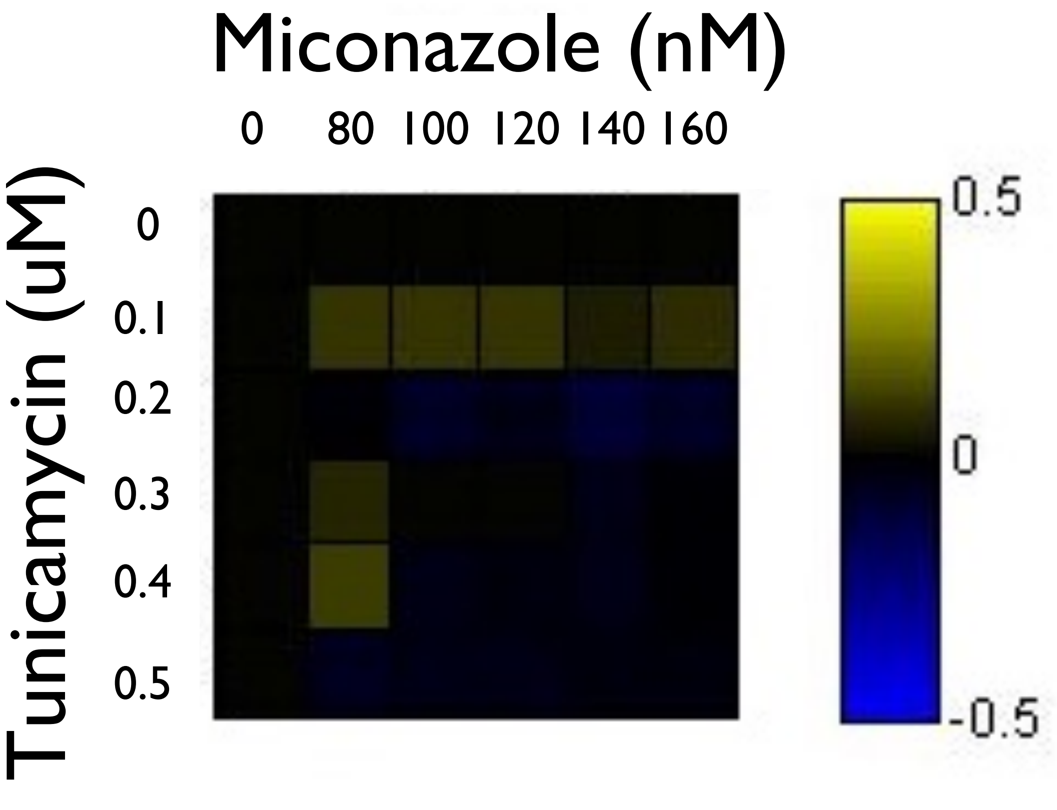
Miconazole (nM)
0 80 100 120 140 160
Tunicamycin (uM)
0
0.1
0.2
0.3
0.4
0.5
0.5
0
-0.5

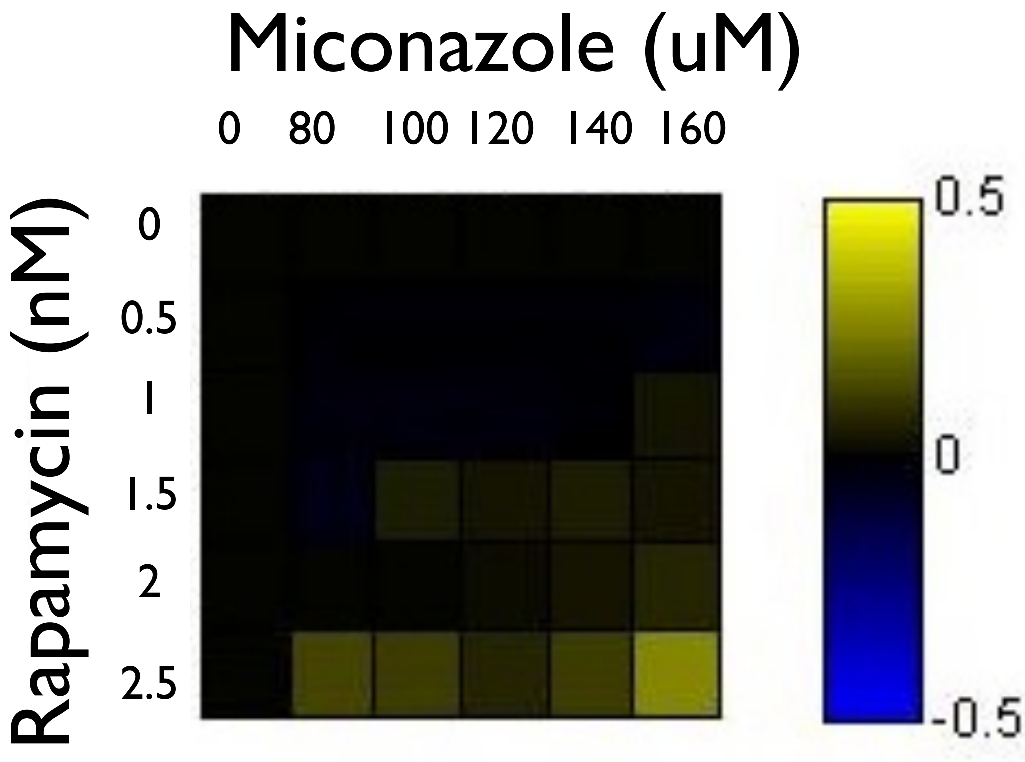
Miconazole (uM)
0 80 100 120 140 160
Rapamycin (nM)
0
0.5
1
1.5
2
2.5
0.5
0
-0.5

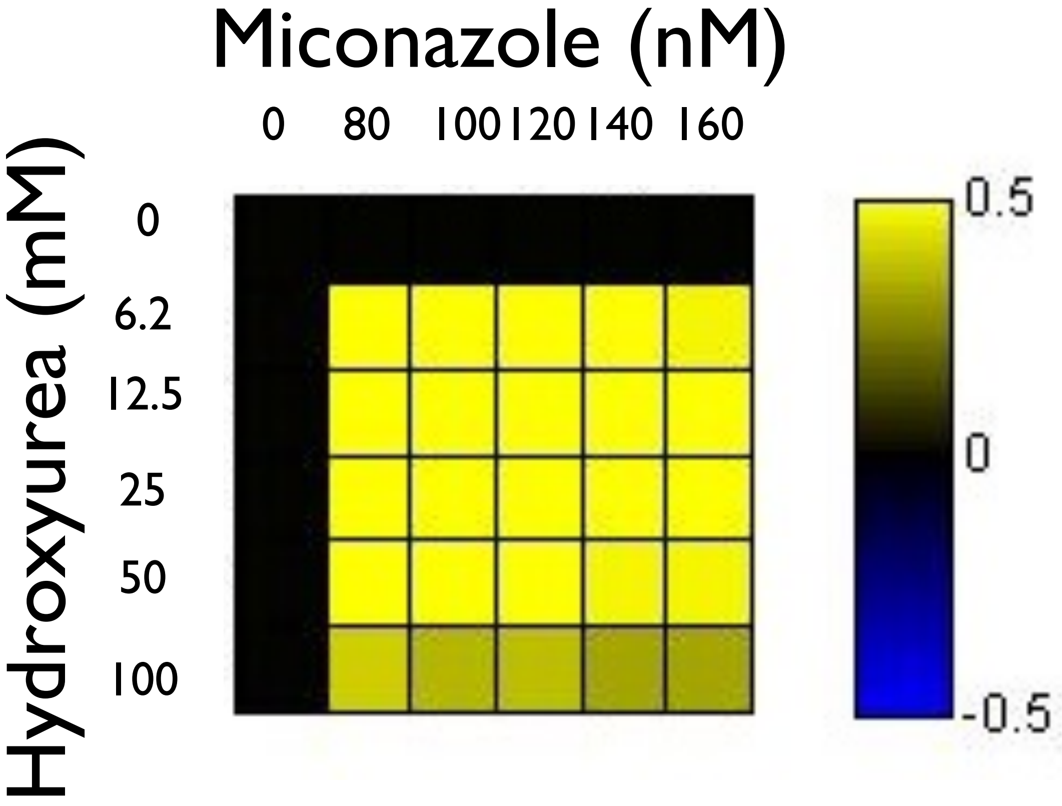
Miconazole (nM)
0 80 100 120 140 160
Hydroxyurea (mM)
0
6.2
12.5
25
50
100
0.5
0
-0.5

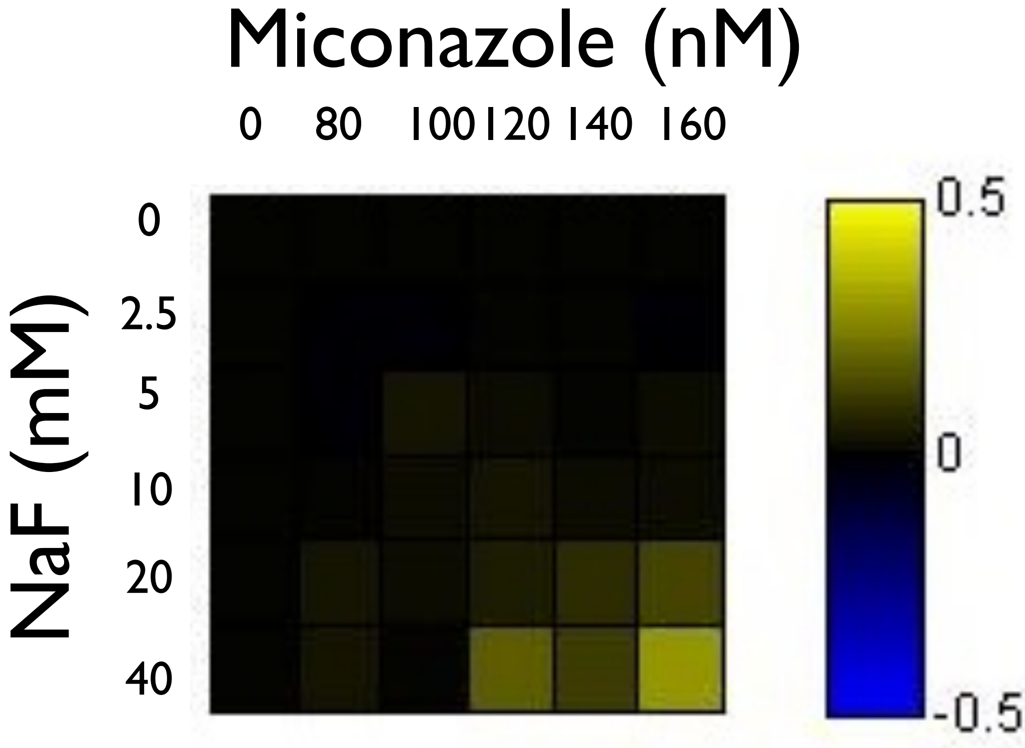
Miconazole (nM)
0 80 100 120 140 160
NaF (mM)
0
2.5
5
10
20
40
0.5
0
-0.5

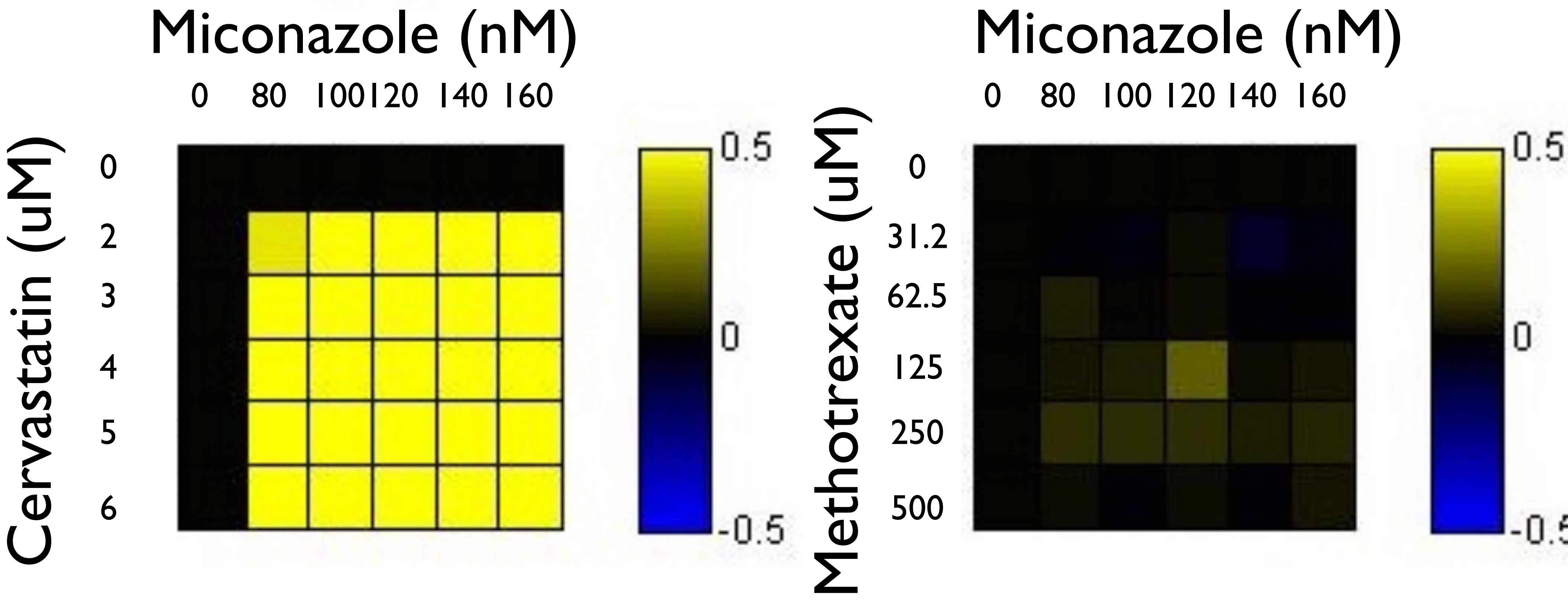
Miconazole (nM)
0 80 100120 140 160
Cervastatin (uM)
0
2
3
4
5
6
0.5
0
-0.5
Miconazole (nM)
0 80 100 120 140 160
Methotrexate (uM)
0
31.2
62.5
125
250
500
0.5
0

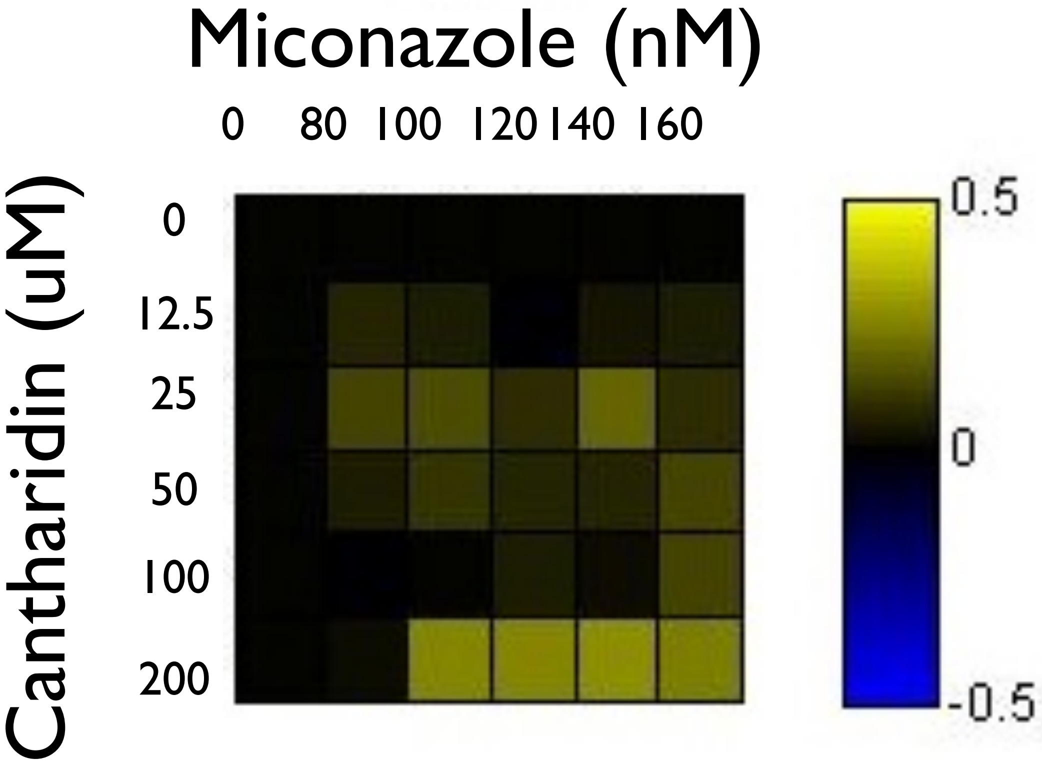
Miconazole (nM)
0 80 100 120 140 160
Cantharidin (uM)
0
12.5
25
50
100
200
0.5
0
-0.5

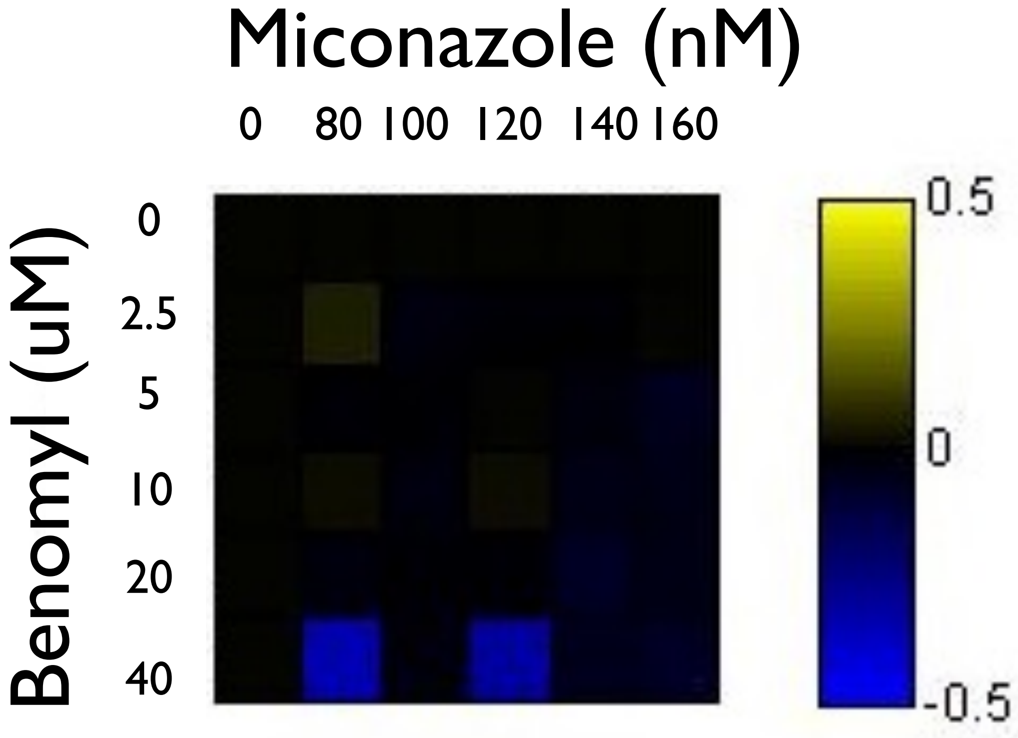
Miconazole (nM)
0 80 100 120 140 160
Benomyl (uM)
0
2.5
5
10
20
40
0.5
0
-0.5

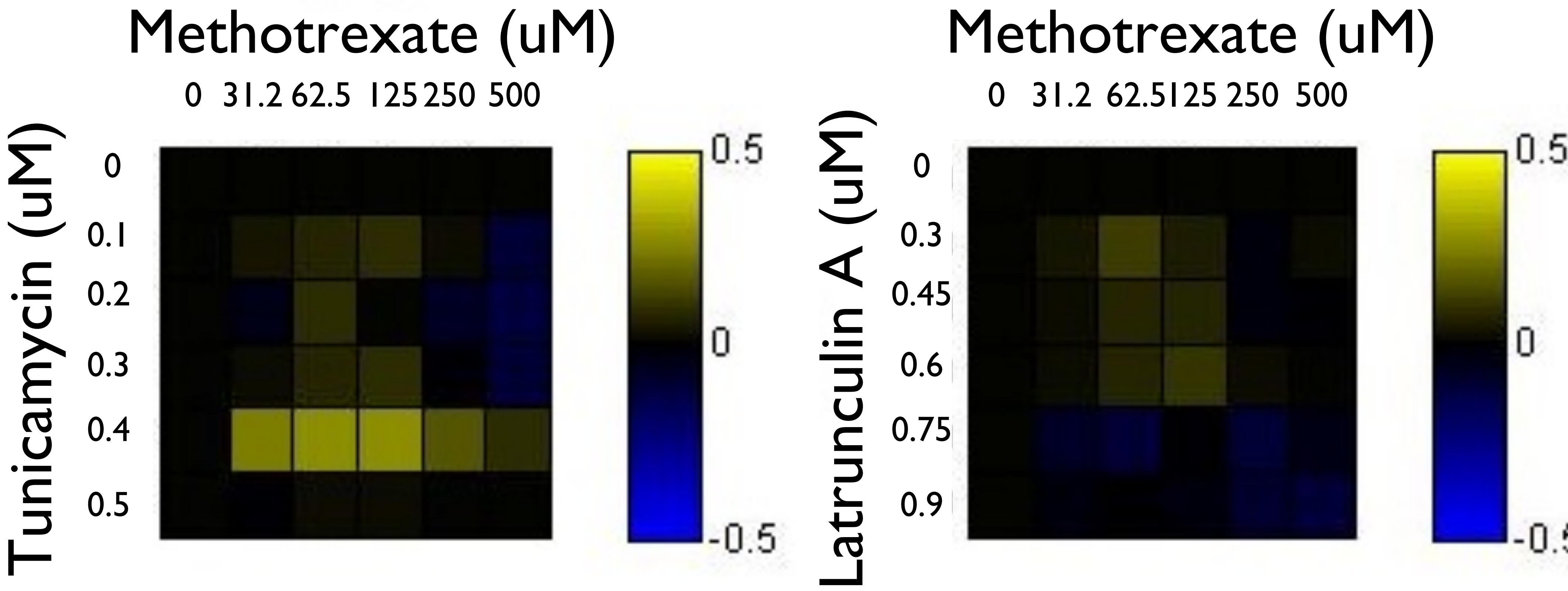
Methotrexate (uM)
0 31.2 62.5 125 250 500
Tunicamycin (uM)
0
0.1
0.2
0.3
0.4
0.5
0.5
0
-0.5
Methotrexate (uM)
0 31.2 62.5125 250 500
Latrunculin A (uM)
0
0.3
0.45
0.6
0.75
0.9
0.5
0

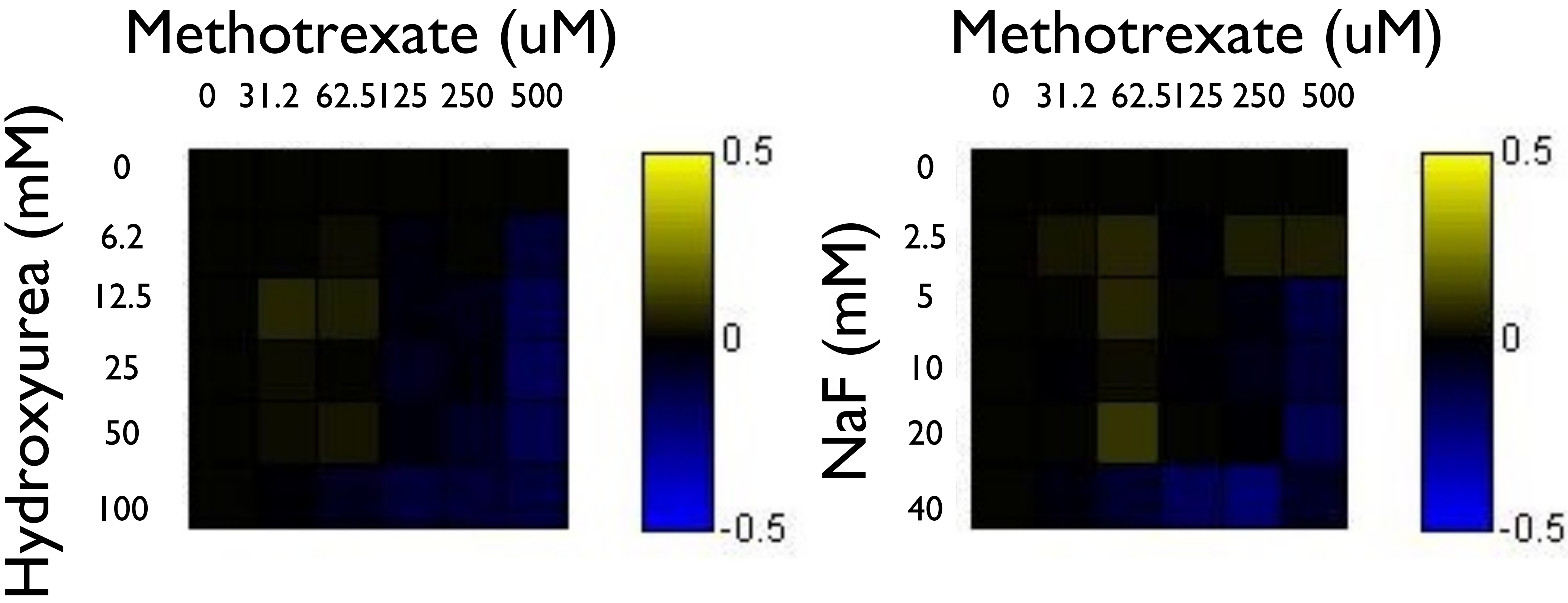
Methotrexate (uM)
0 31.2 62.5125 250 500
Hydroxyurea (mM)
0
6.2
12.5
25
50
100
0.5
0
-0.5
Methotrexate (uM)
0 31.2 62.5125 250 500
NaF (mM)
0
2.5
5
10
20
40
0.5
0
-0.5

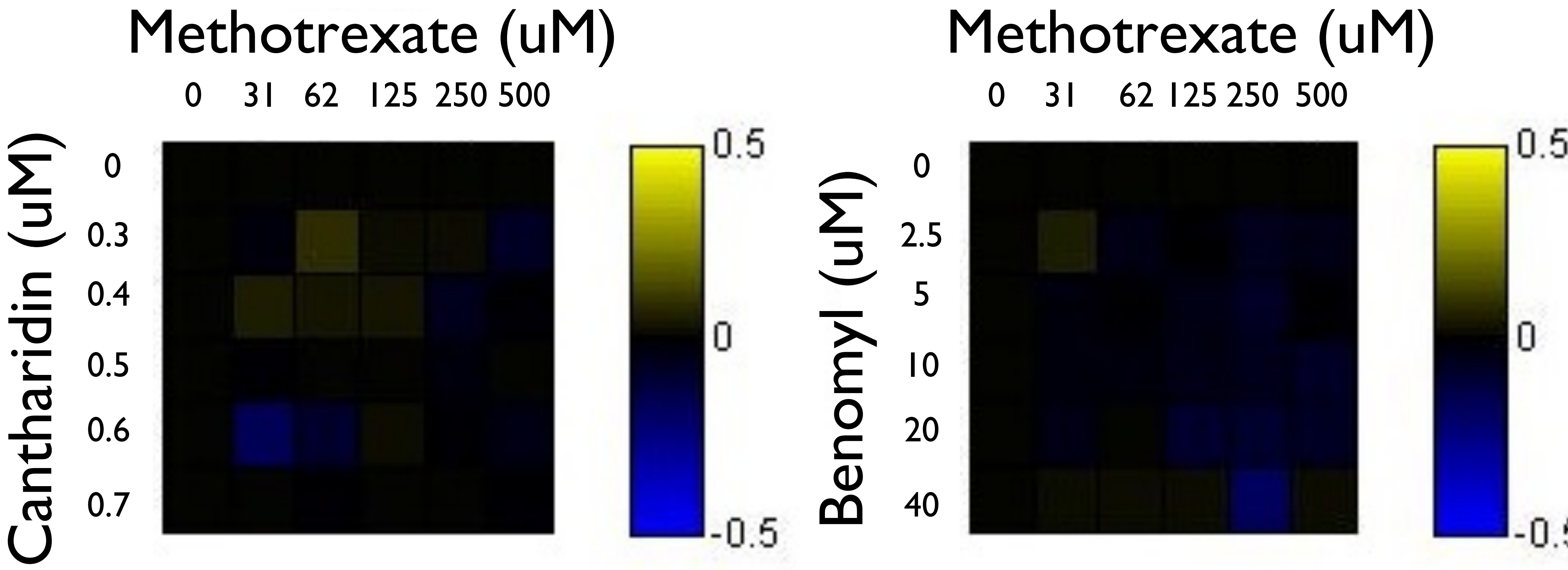
Methotrexate (uM)
0 31 62 125 250 500
Cantharidin (uM)
0
0.3
0.4
0.5
0.6
0.7
0.5
0
-0.5
Methotrexate (uM)
0 31 62 125 250 500
Benomyl (uM)
0
2.5
5
10
20
40
0.5
0
-0.5

REPEAT

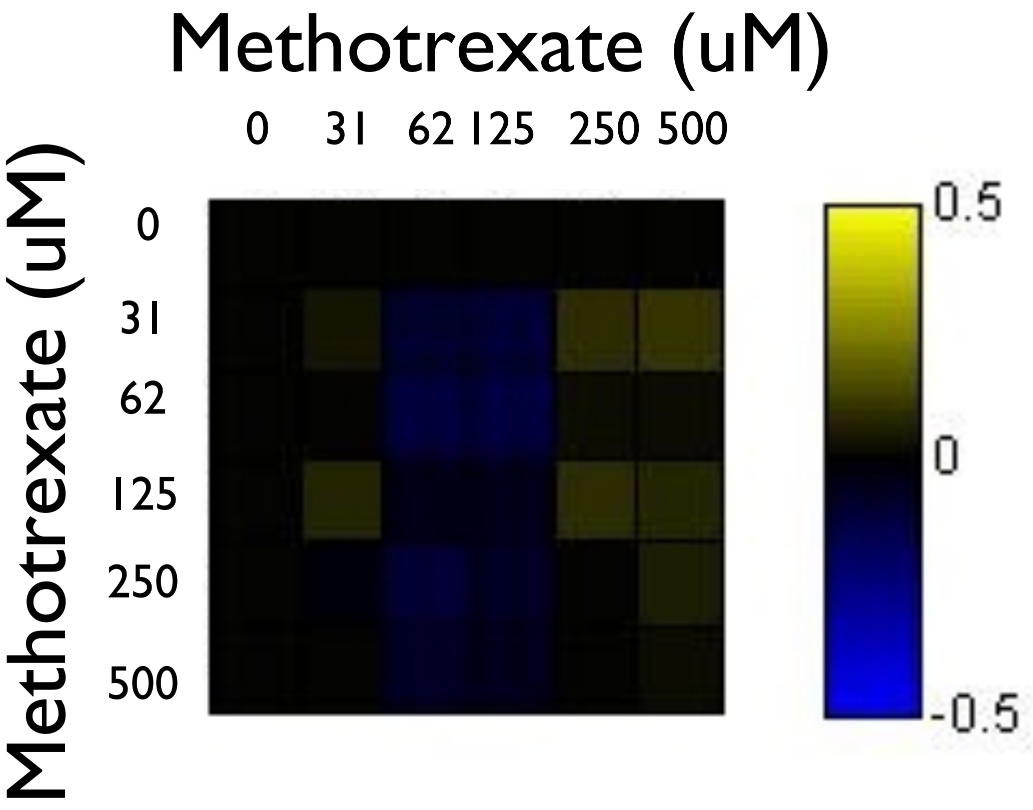
Methotrexate (uM)
0 31 62 125 250 500
Methotrexate (uM)
0
31
62
125
250
500
0.5
0
-0.5

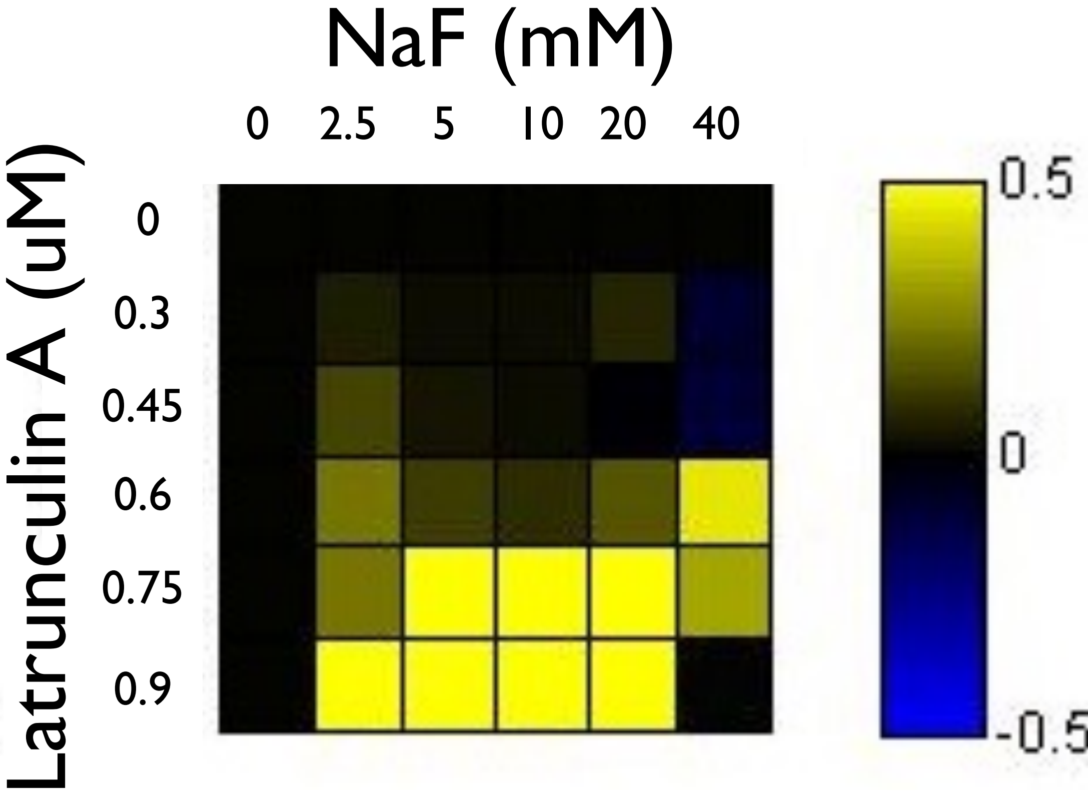
NaF (mM)
0 2.5 5 10 20 40
Latrunculin A (uM)
0
0.3
0.45
0.6
0.75
0.9
0.5
0
-0.5

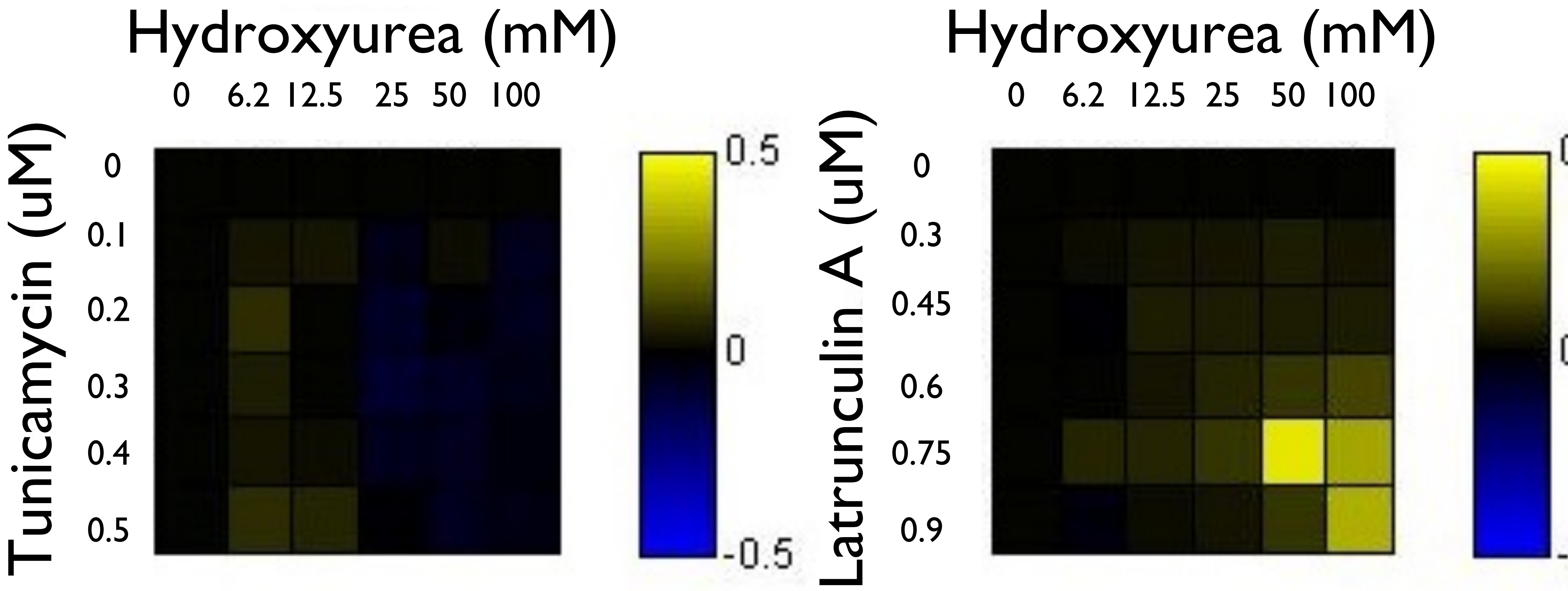

Hydroxyurea (mM)
0 6.2 12.5 25 50 100
Tunicamycin (uM)
0
0.1
0.2
0.3
0.4
0.5
0.5
0
-0.5
Hydroxyurea (mM)
0 6.2 12.5 25 50 100
Latrunculin A (uM)
0
0.3
0.45
0.6
0.75
0.9

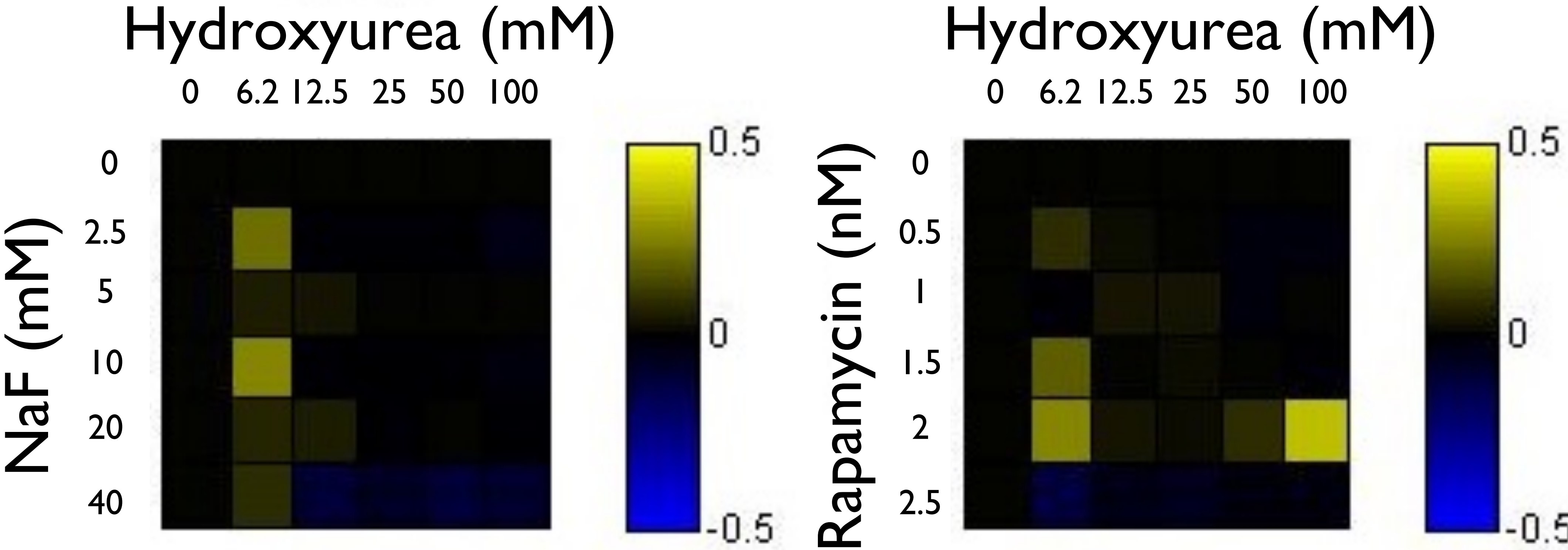

Hydroxyurea (mM)
0 6.2 12.5 25 50 100
NaF (mM)
0
2.5
5
10
20
40
0.5
0
-0.5
Hydroxyurea (mM)
0 6.2 12.5 25 50 100
Rapamycin (nM)
0
0.5
1
1.5
2
2.5
0.5
0
-0.5

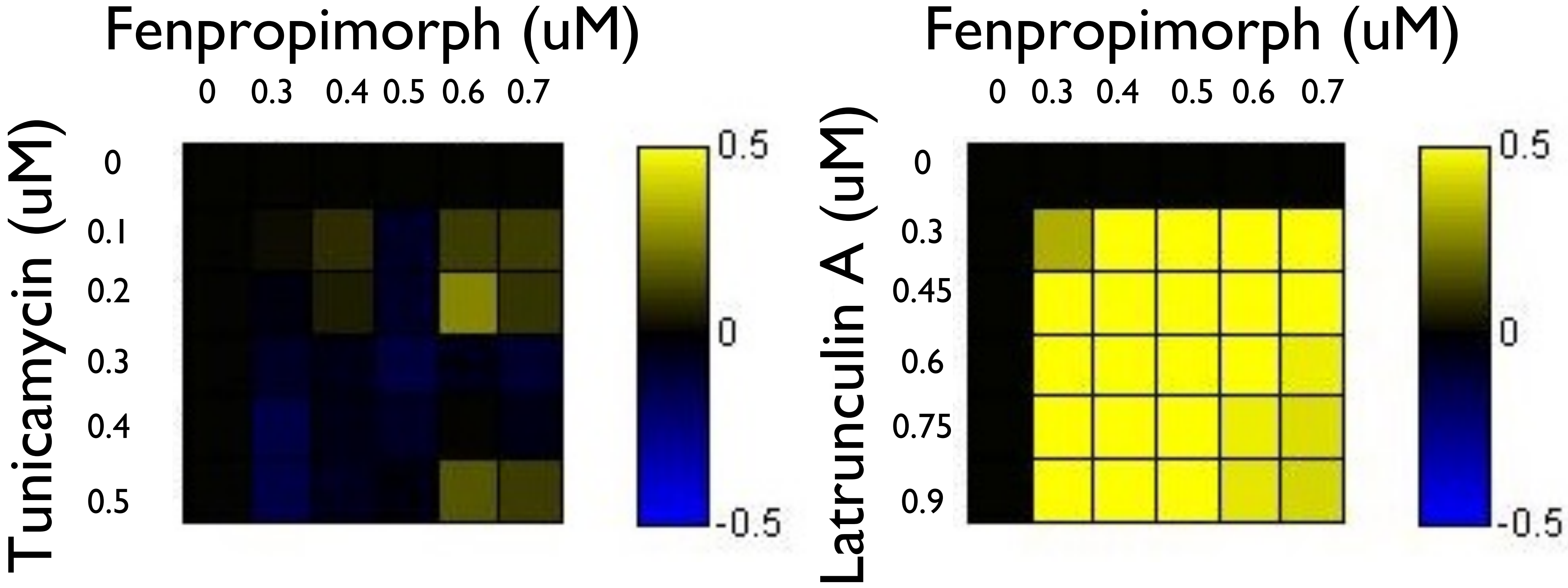

Fenpropimorph (uM)
0 0.3 0.4 0.5 0.6 0.7
Tunicamycin (uM)
0
0.1
0.2
0.3
0.4
0.5
0.5
0
-0.5
Fenpropimorph (uM)
0 0.3 0.4 0.5 0.6 0.7
Latrunculin A (uM)
0
0.3
0.45
0.6
0.75
0.9
0.5
0
-0.5

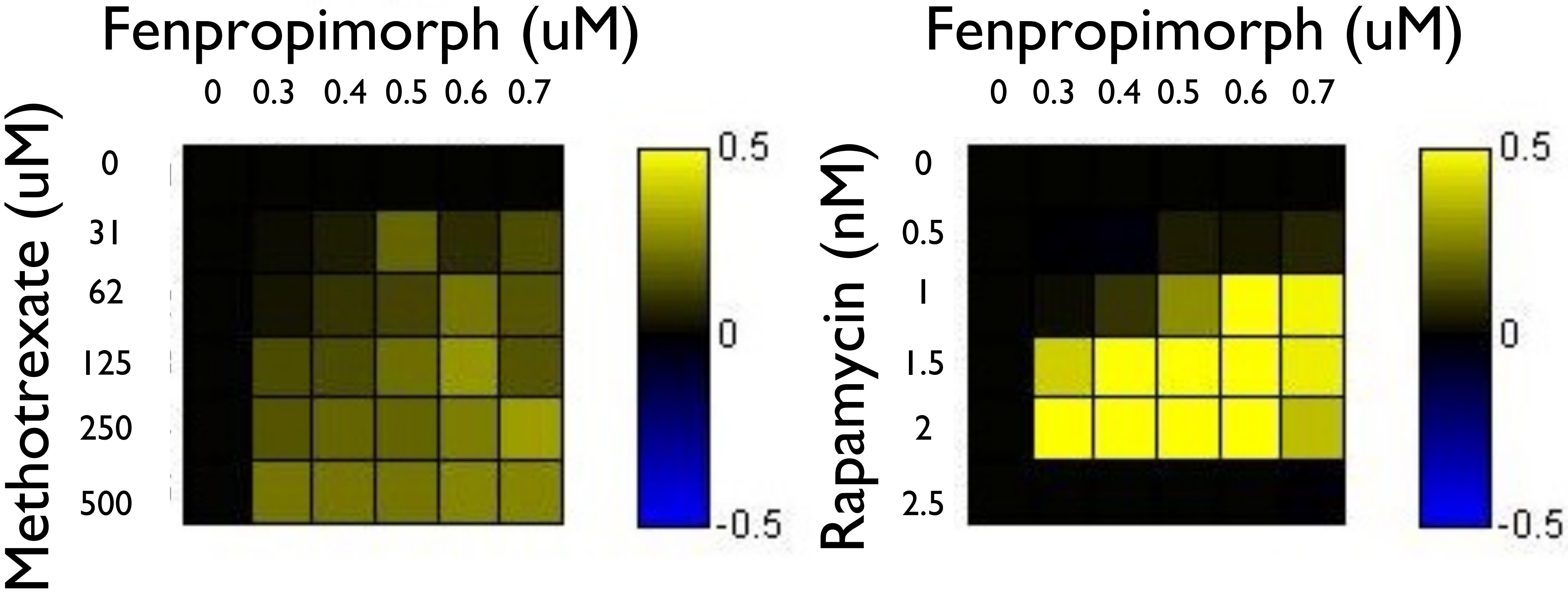
Fenpropimorph (uM)
0 0.3 0.4 0.5 0.6 0.7
Methotrexate (uM)
0 31 62 125 250 500
0.5 0 -0.5
Fenpropimorph (uM)
0 0.3 0.4 0.5 0.6 0.7
Rapamycin (nM)
0 0.5 1 1.5 2 2.5
0.5 0 -0.5

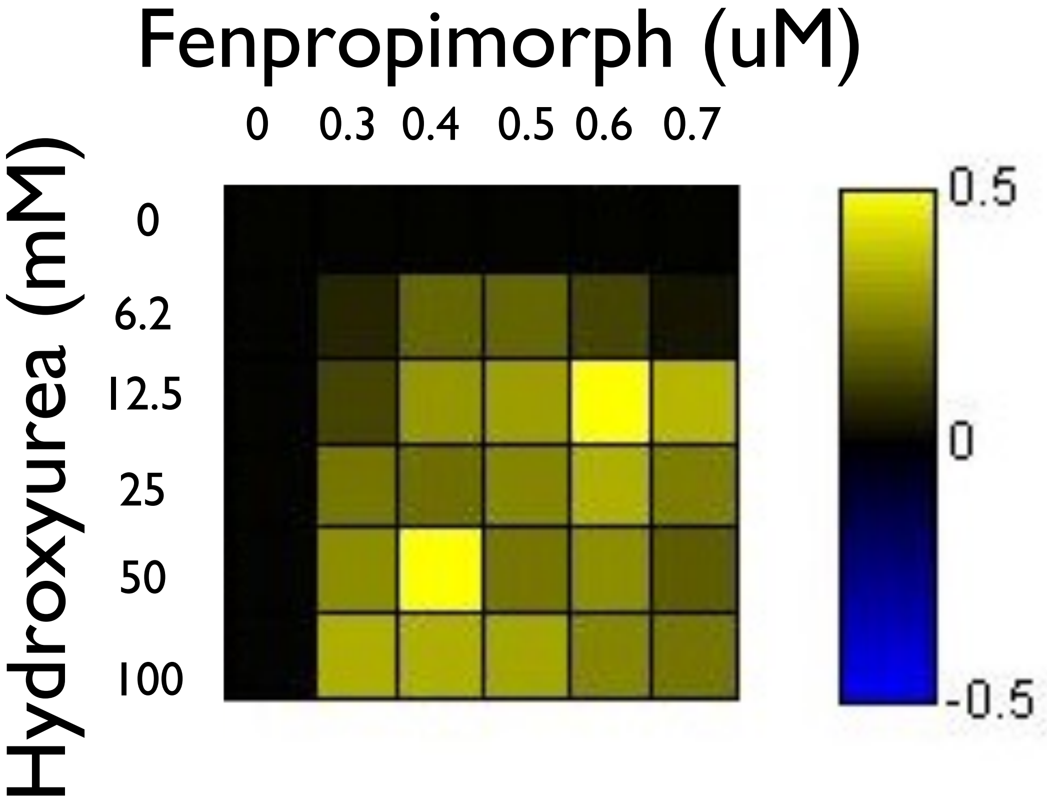

Fenpropimorph (uM)
0 0.3 0.4 0.5 0.6 0.7
Hydroxyurea (mM)
0
6.2
12.5
25
50
100
0.5
0
-0.5

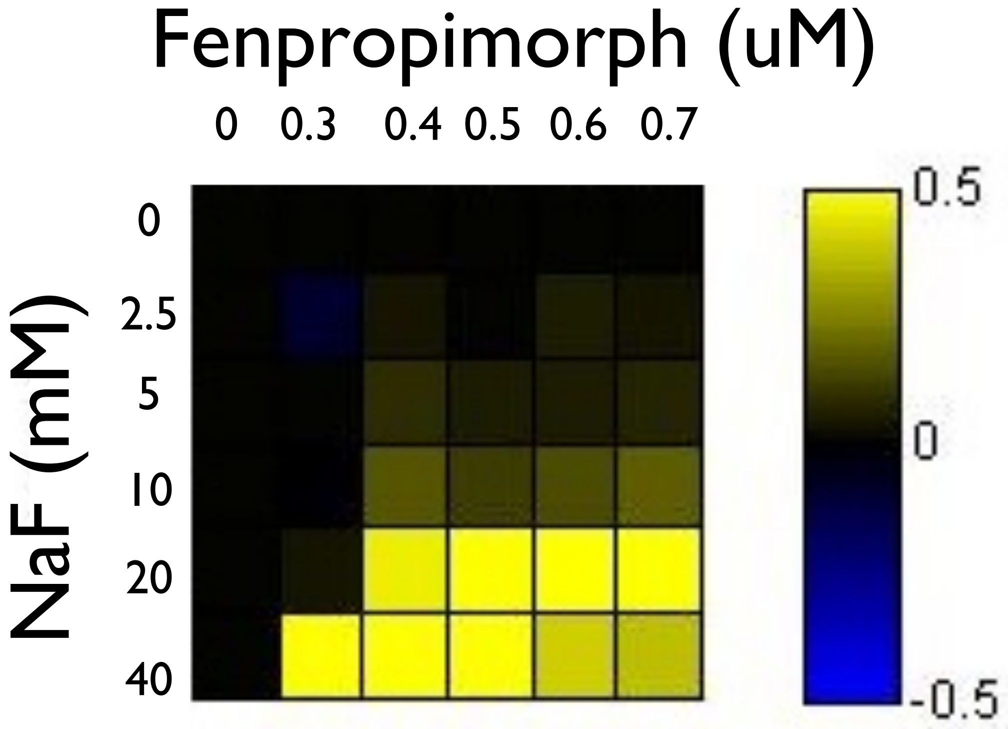

Fenpropimorph (uM)
0 0.3 0.4 0.5 0.6 0.7
NaF (mM)
0
2.5
5
10
20
40
0.5
0
-0.5

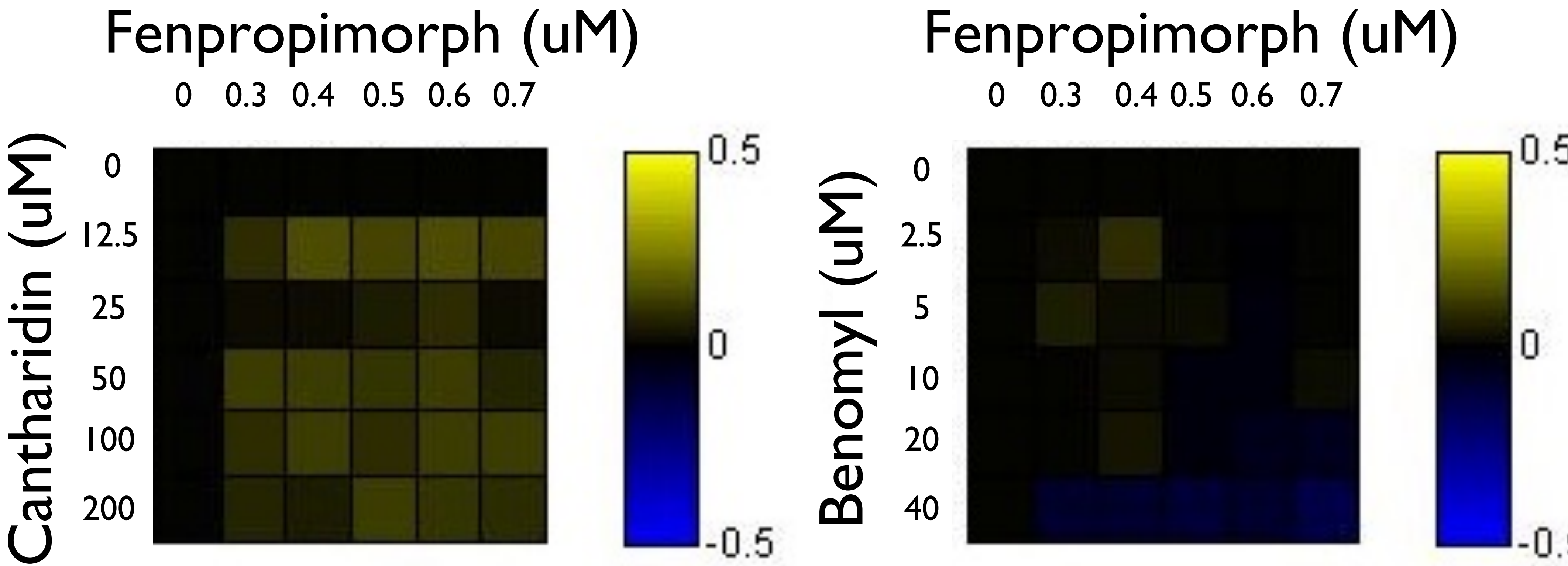

Fenpropimorph (uM)
0 0.3 0.4 0.5 0.6 0.7
Cantharidin (uM)
0
12.5
25
50
100
200
0.5
0
-0.5
Fenpropimorph (uM)
0 0.3 0.4 0.5 0.6 0.7
Benomyl (uM)
0
2.5
5
10
20
40
0

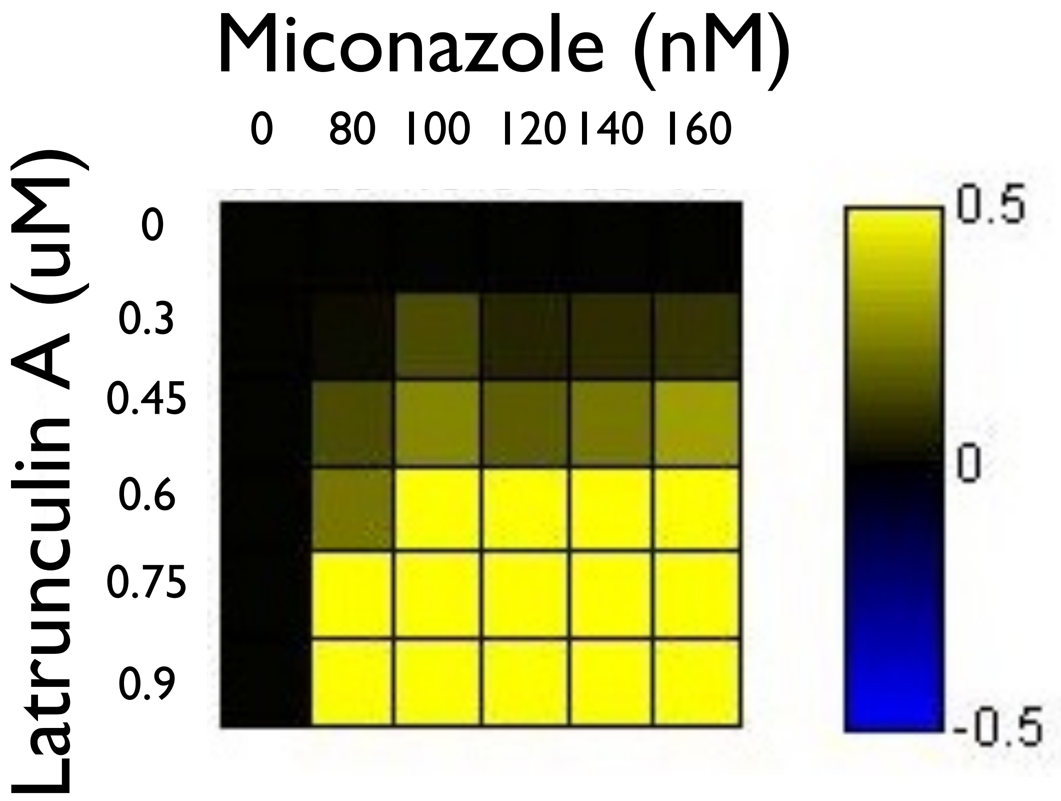

Miconazole (nM)
0
80
100
120
140
160
Latrunculin A (uM)
0
0.3
0.45
0.6
0.75
0.9
0.5
0
-0.5

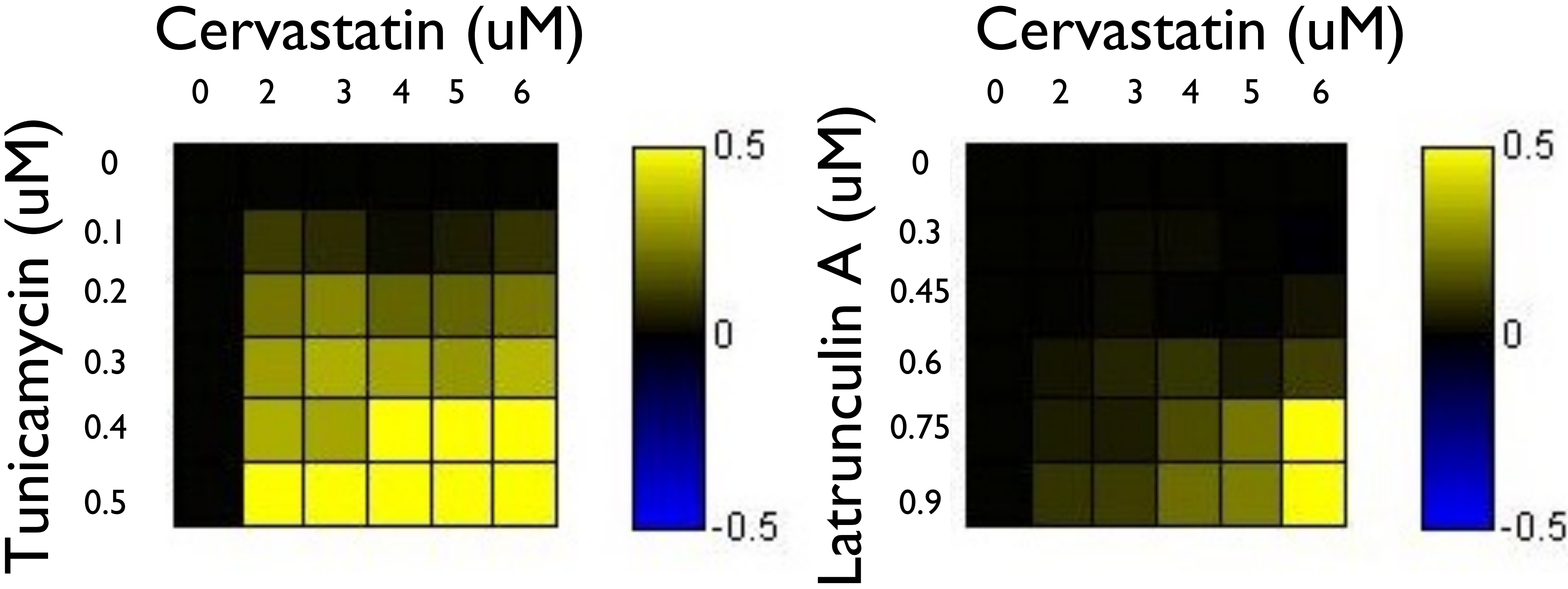
Cervastatin (uM)
0 2 3 4 5 6
Tunicamycin (uM)
0
0.1
0.2
0.3
0.4
0.5
0.5
0
-0.5
Cervastatin (uM)
0 2 3 4 5 6
Latrunculin A (uM)
0
0.3
0.45
0.6
0.75
0.9
0.5
0
-0.5

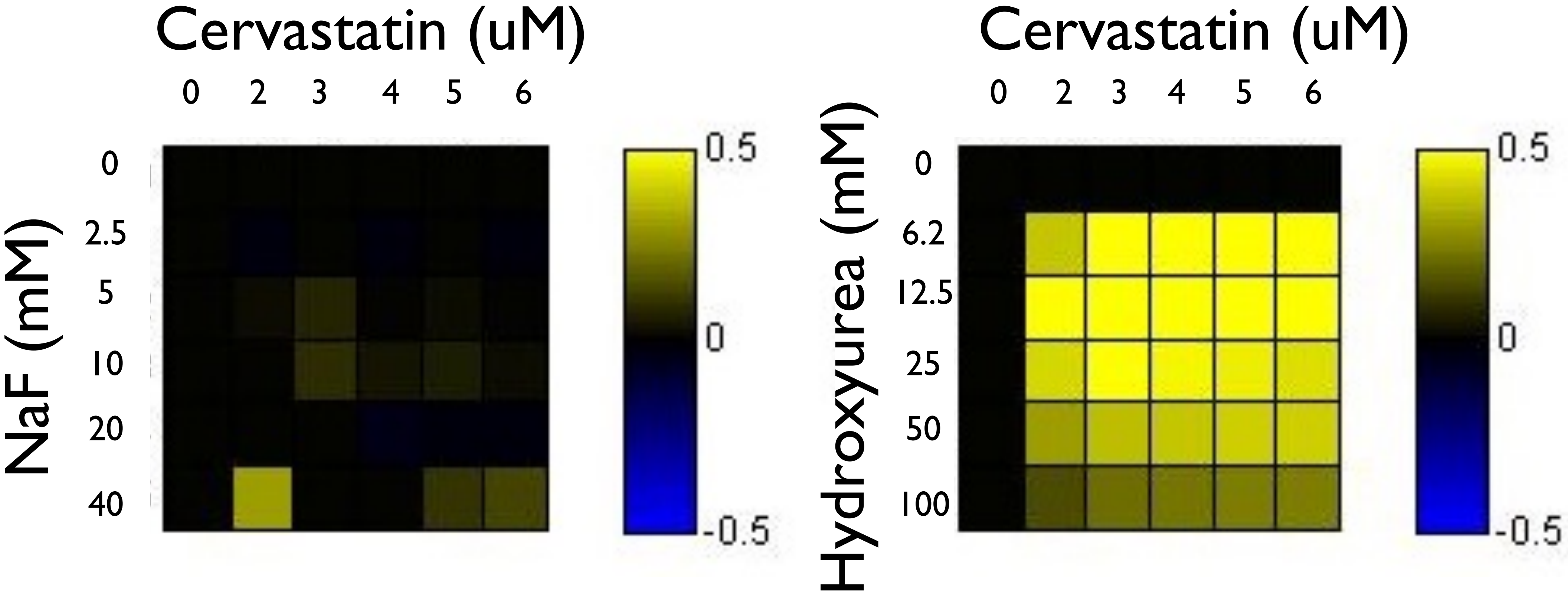
Cervastatin (uM)
0 2 3 4 5 6
NaF (mM)
0
2.5
5
10
20
40
0.5
0
-0.5
Cervastatin (uM)
0 2 3 4 5 6
Hydroxyurea (mM)
0
6.2
12.5
25
50
100
0.5
0
-0.5

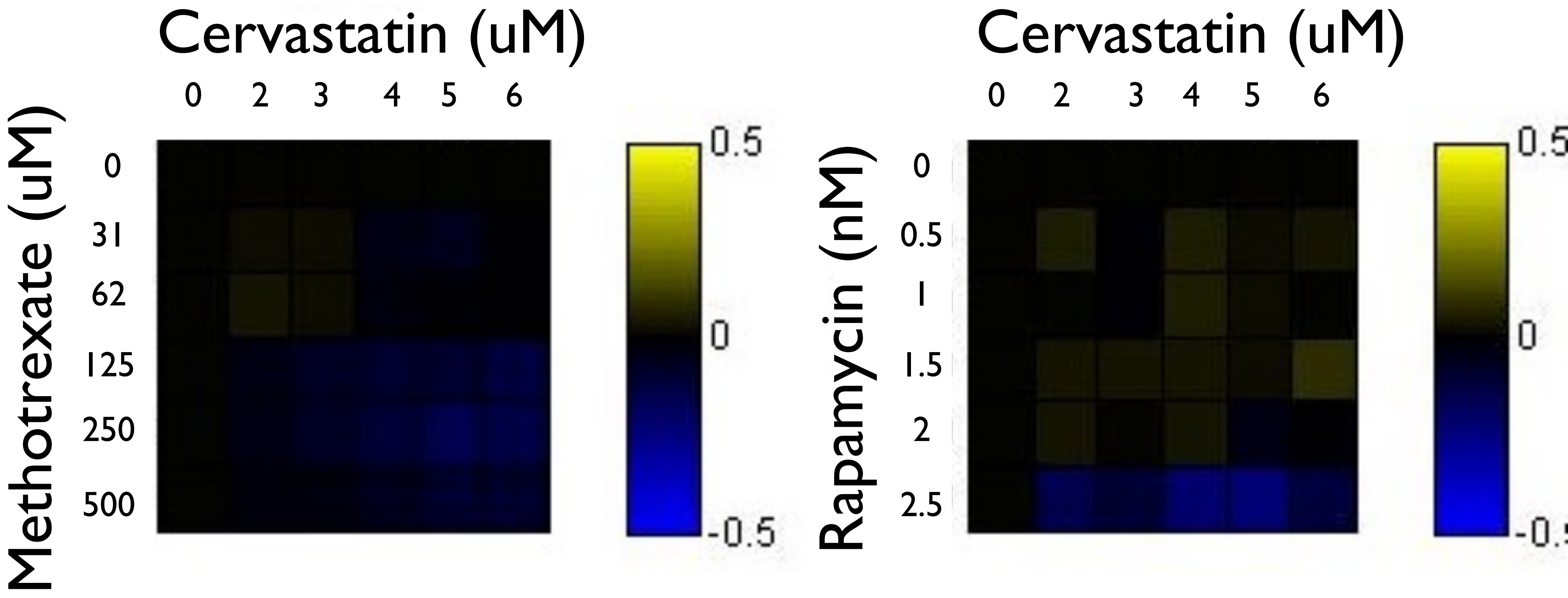
Cervastatin (uM)
0 2 3 4 5 6
Methotrexate (uM)
0
31
62
125
250
500
0.5
0
-0.5
Cervastatin (uM)
0 2 3 4 5 6
Rapamycin (nM)
0
0.5
1
1.5
2
2.5
0.5
0

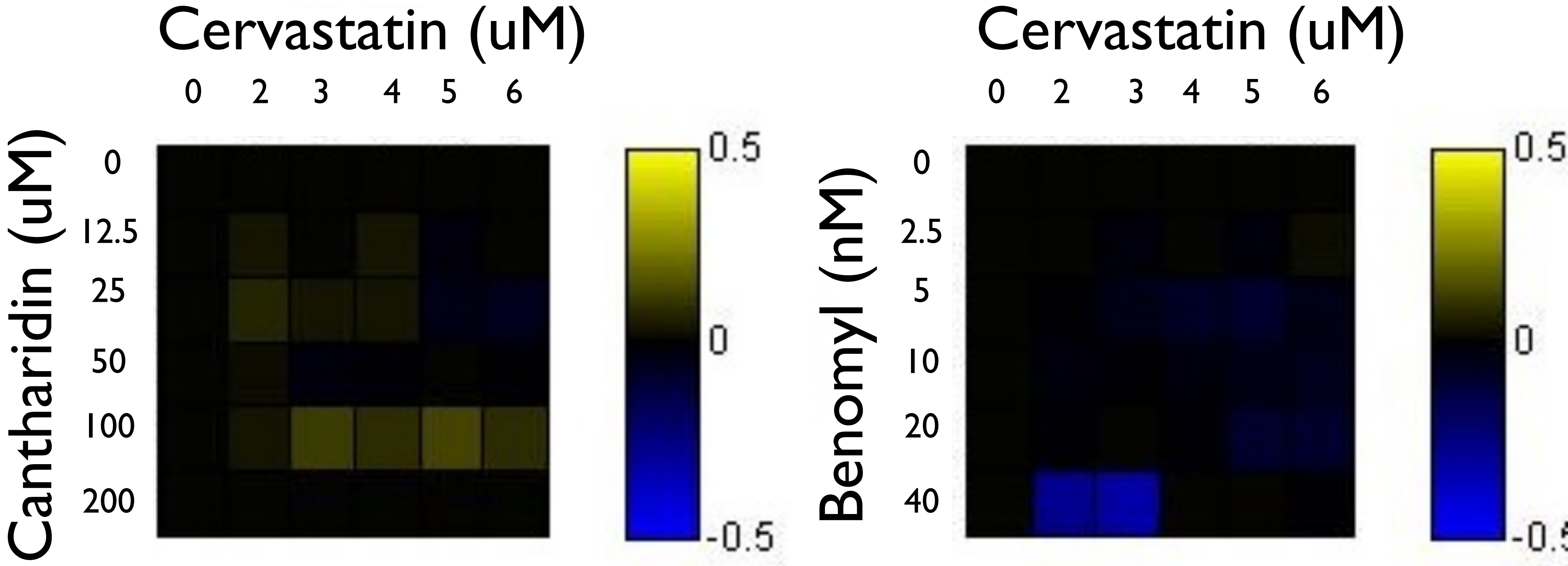

Cervastatin (uM)
0 2 3 4 5 6
Cantharidin (uM)
0
12.5
25
50
100
200
0.5
0
-0.5
Cervastatin (uM)
0 2 3 4 5 6
Benomyl (nM)
0
2.5
5
10
20
40
0.5
0

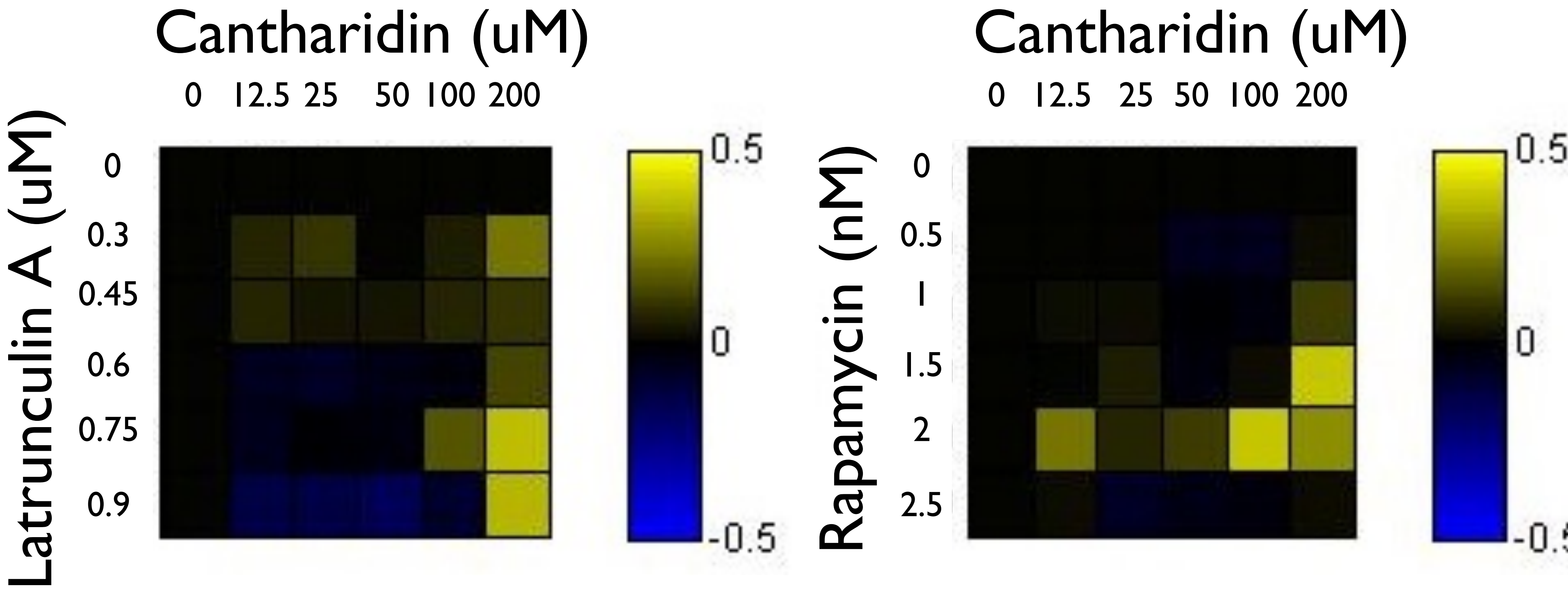
Cantharidin (uM)
0 12.5 25 50 100 200
Latrunculin A (uM)
0
0.3
0.45
0.6
0.75
0.9
0.5
0
-0.5
Cantharidin (uM)
0 12.5 25 50 100 200
Rapamycin (nM)
0
0.5
1
1.5
2
2.5
0.5
0
-0.5

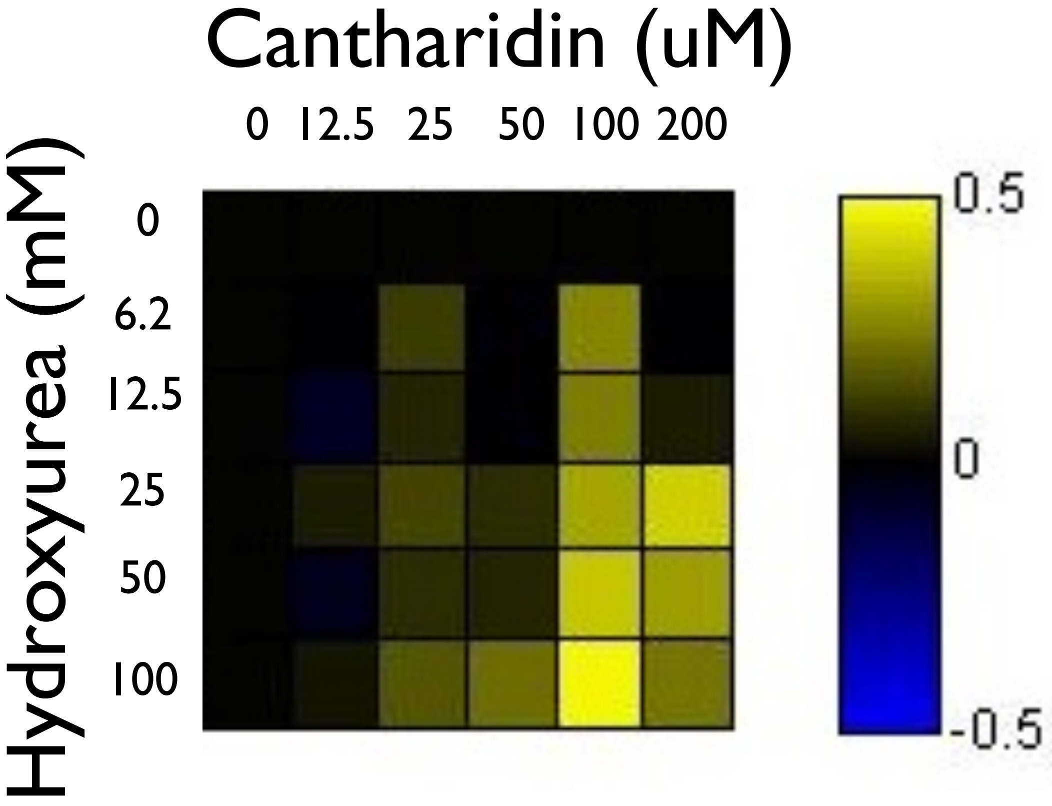
Cantharidin (uM)
0 12.5 25 50 100 200
Hydroxyurea (mM)
0
6.2
12.5
25
50
100
0.5
0
-0.5

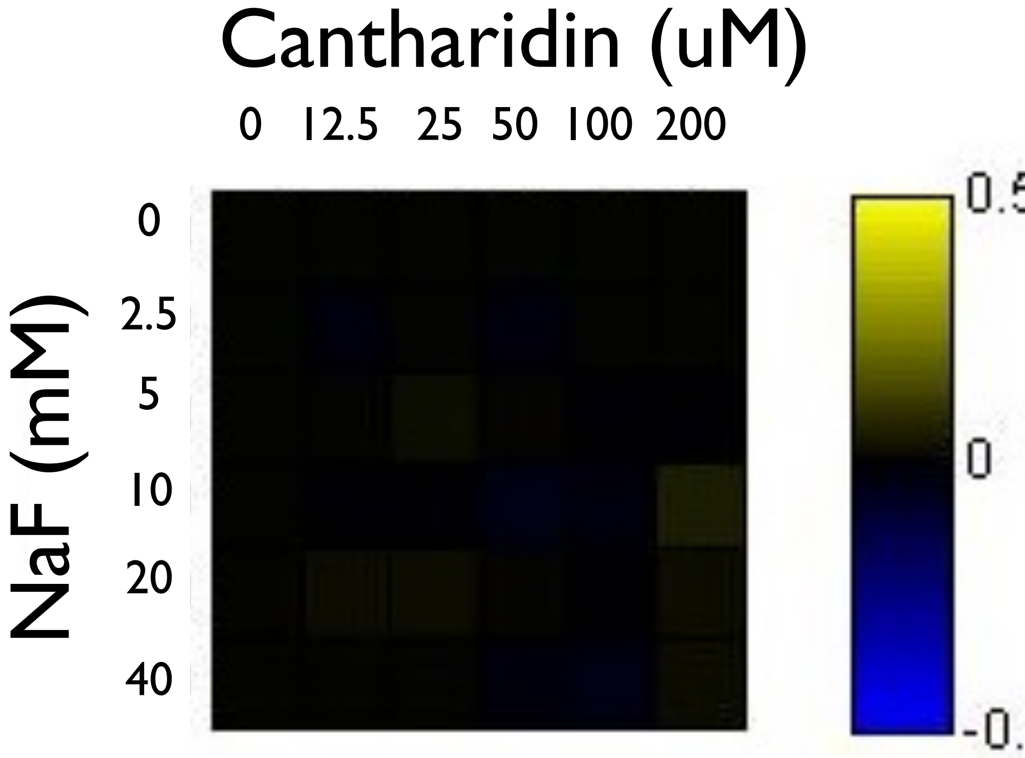
Cantharidin (uM)
0 12.5 25 50 100 200
NaF (mM)
0
2.5
5
10
20
40
0

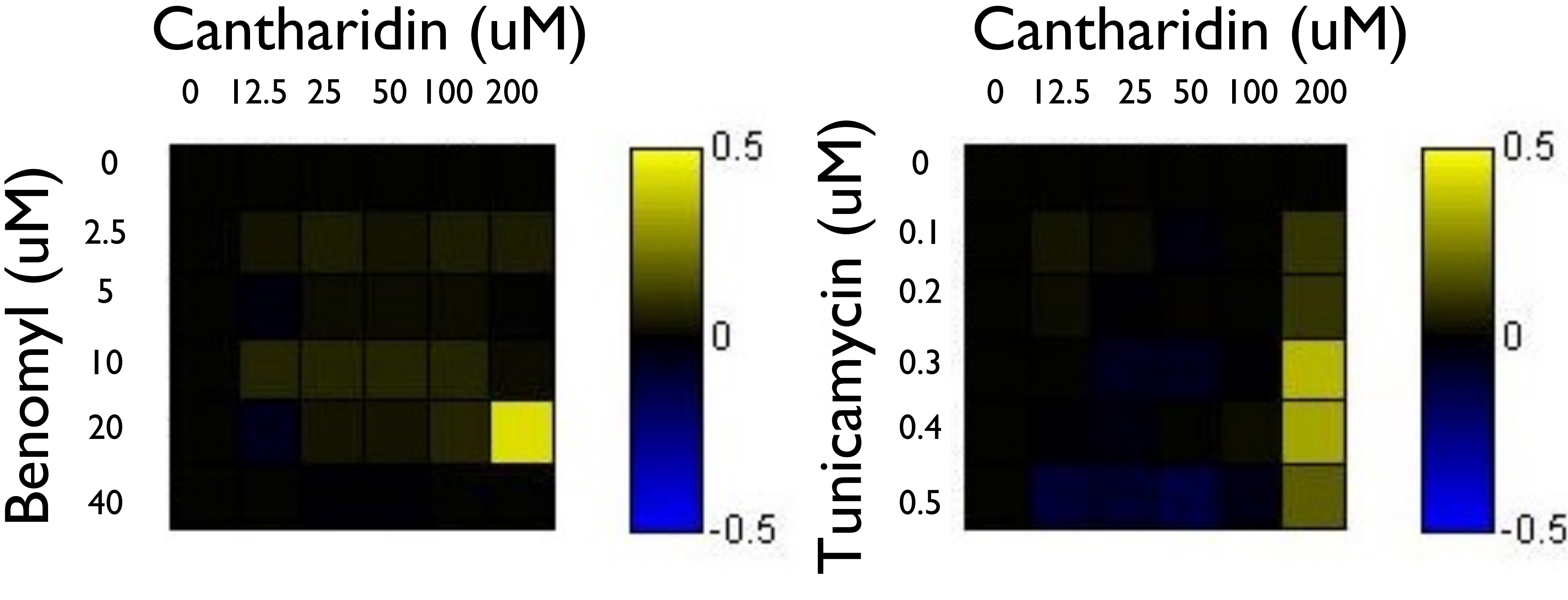
Cantharidin (uM)
0 12.5 25 50 100 200
Benomyl (uM)
0
2.5
5
10
20
40
0.5
0
-0.5
Cantharidin (uM)
0 12.5 25 50 100 200
Tunicamycin (uM)
0
0.1
0.2
0.3
0.4
0.5
0.5
0
-0.5

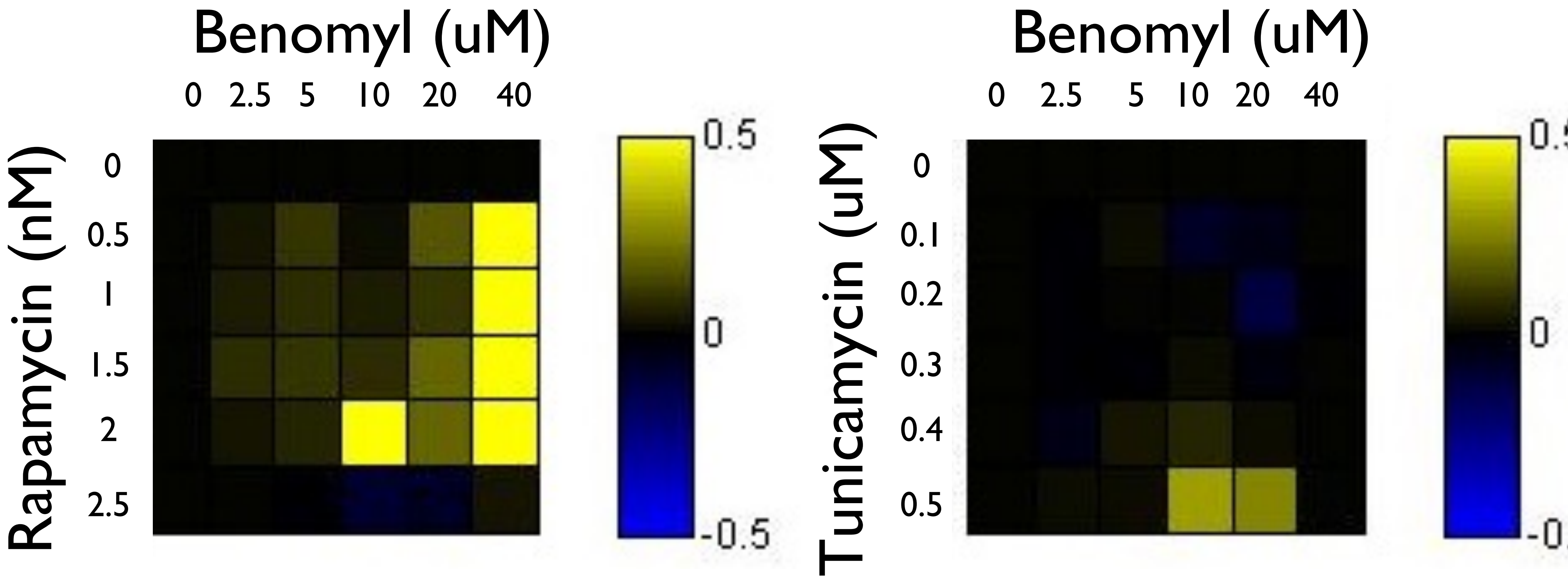
Benomyl (uM)
0 2.5 5 10 20 40
Rapamycin (nM)
0
0.5
1
1.5
2
2.5
0.5
0
-0.5
Benomyl (uM)
0 2.5 5 10 20 40
Tunicamycin (uM)
0
0.1
0.2
0.3
0.4
0.5
0

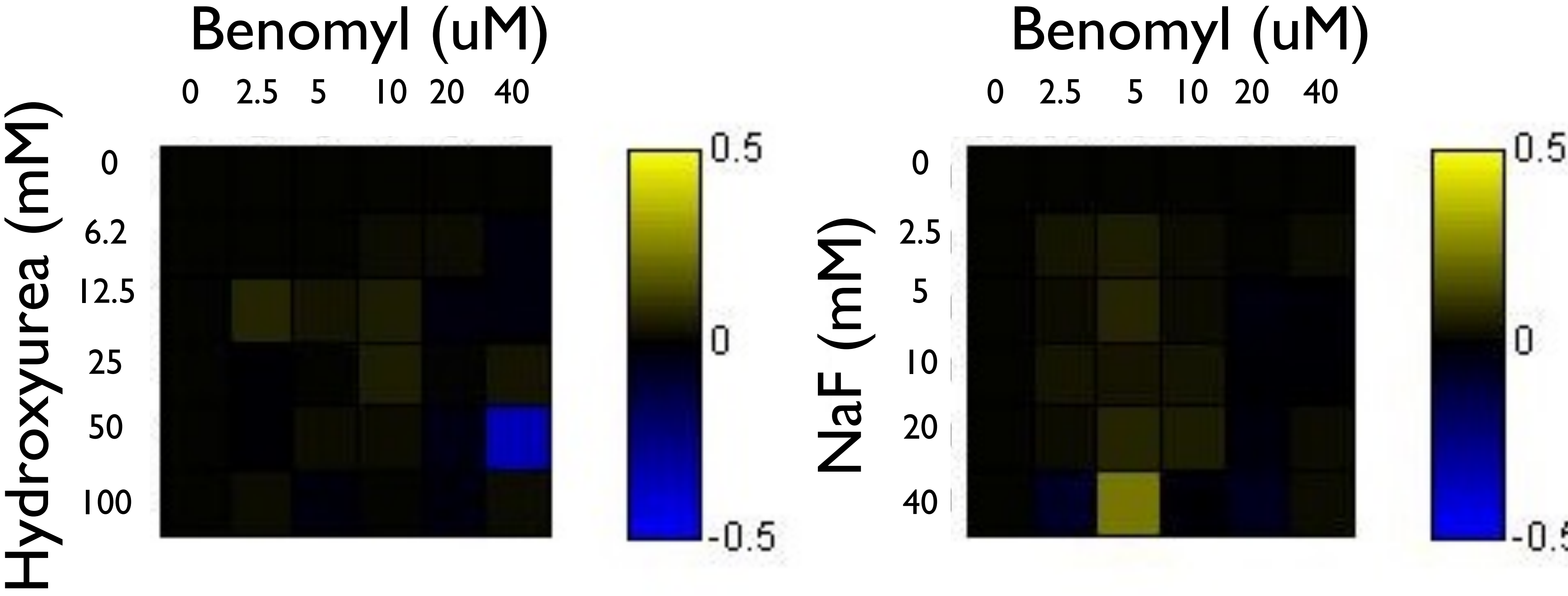

Benomyl (uM)
0 2.5 5 10 20 40
Hydroxyurea (mM)
0
6.2
12.5
25
50
100
0.5
0
-0.5
Benomyl (uM)
0 2.5 5 10 20 40
NaF (mM)
0
2.5
5
10
20
40
0.5
0

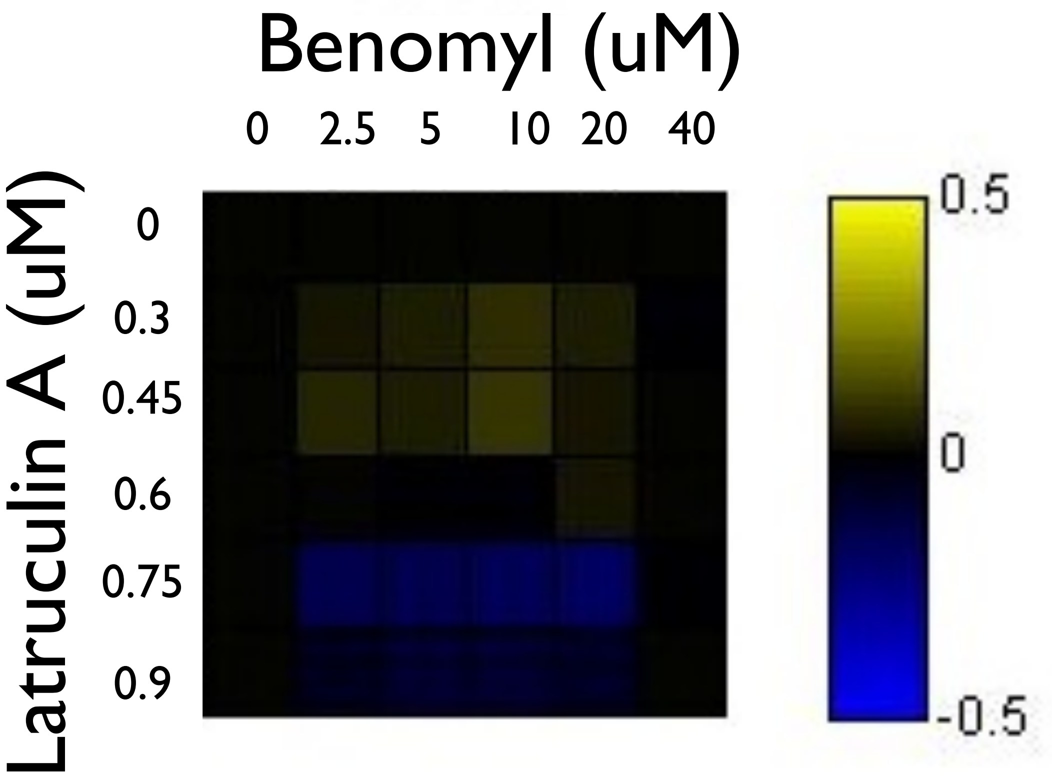
Benomyl (uM)
0 2.5 5 10 20 40
Latrculin A (uM)
0
0.3
0.45
0.6
0.75
0.9
0.5
0
-0.5

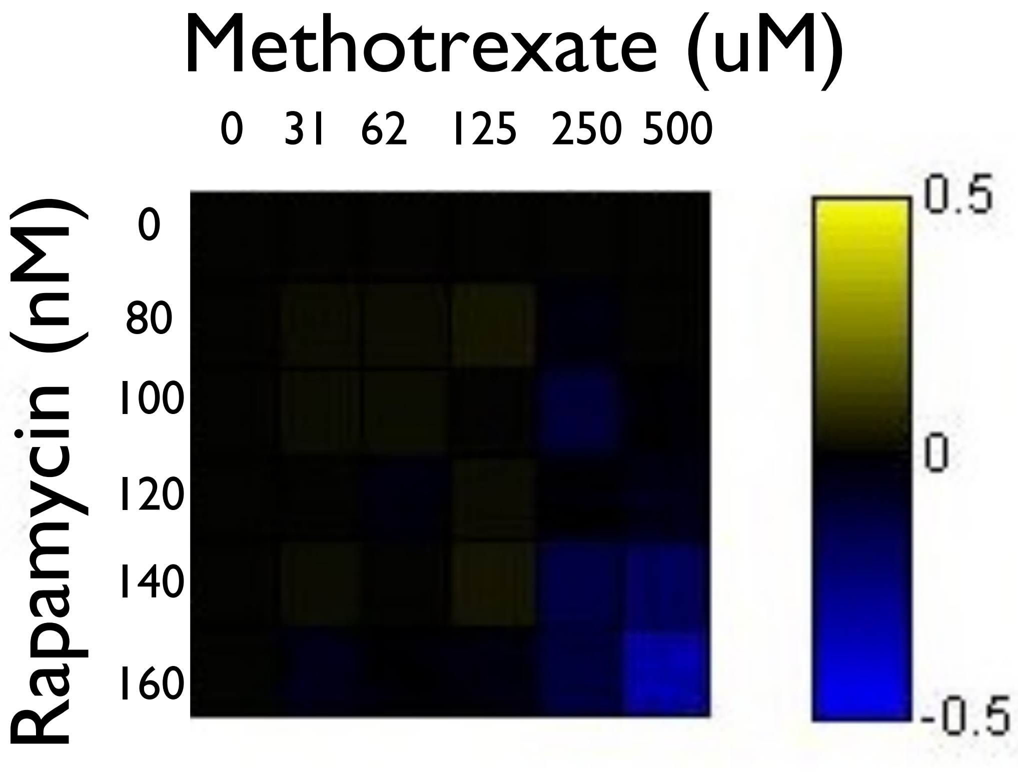
Methotrexate (uM)
0 31 62 125 250 500
Rapamycin (nM)
0
80
100
120
140
160
0.5
0
-0.5

Repeat 1

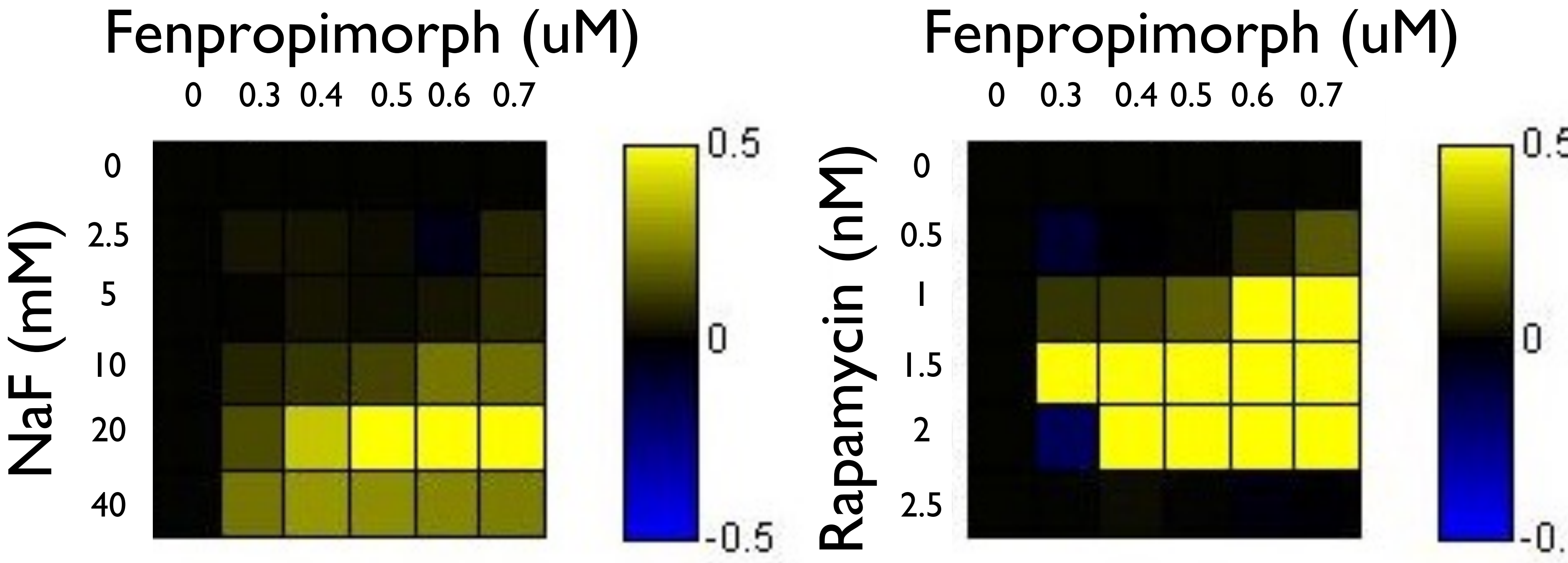
Fenpropimorph (uM)
0 0.3 0.4 0.5 0.6 0.7
NaF (mM)
0 2.5 5 10 20 40
0.5
0
-0.5
Fenpropimorph (uM)
0 0.3 0.4 0.5 0.6 0.7
Rapamycin (nM)
0 0.5 1 1.5 2 2.5
0

Repeat 1

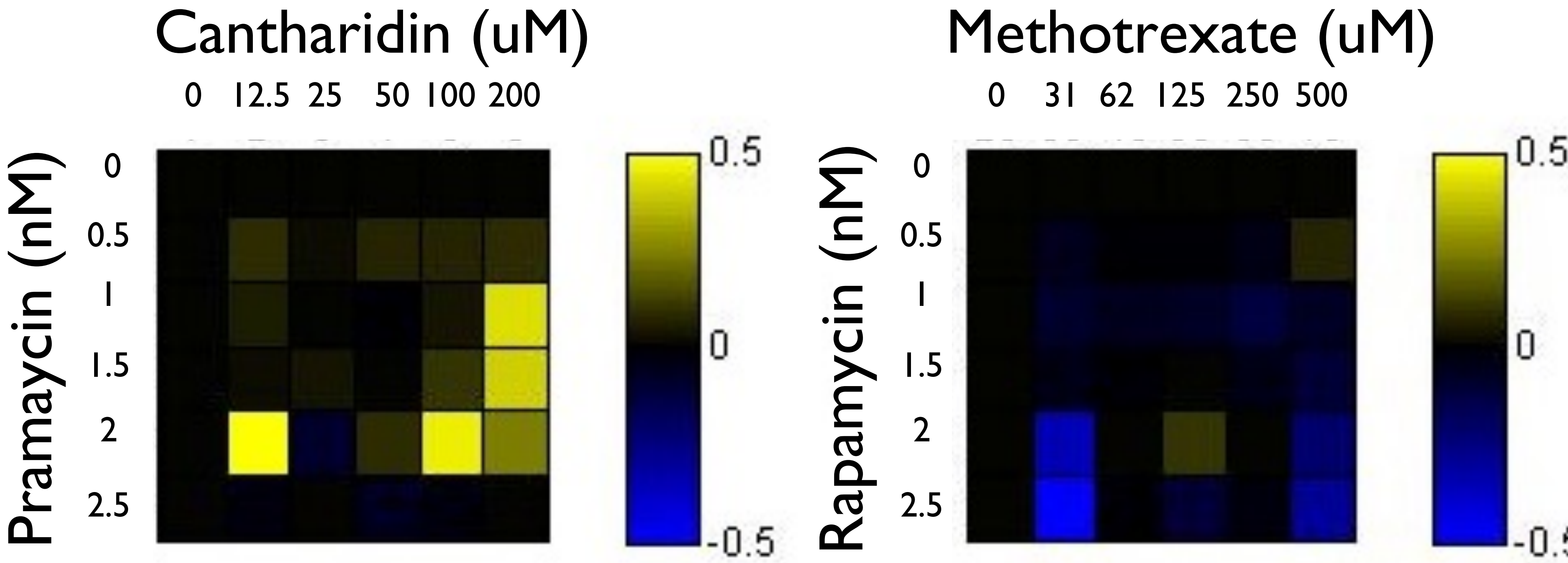

Cantharidin (uM)
0 12.5 25 50 100 200
Pramaycin (nM)
0
0.5
1
1.5
2
2.5
0.5
0
-0.5
Methotrexate (uM)
0 31 62 125 250 500
Rapamycin (nM)
0
0.5
1
1.5
2
2.5
0.5
0

Repeat 1

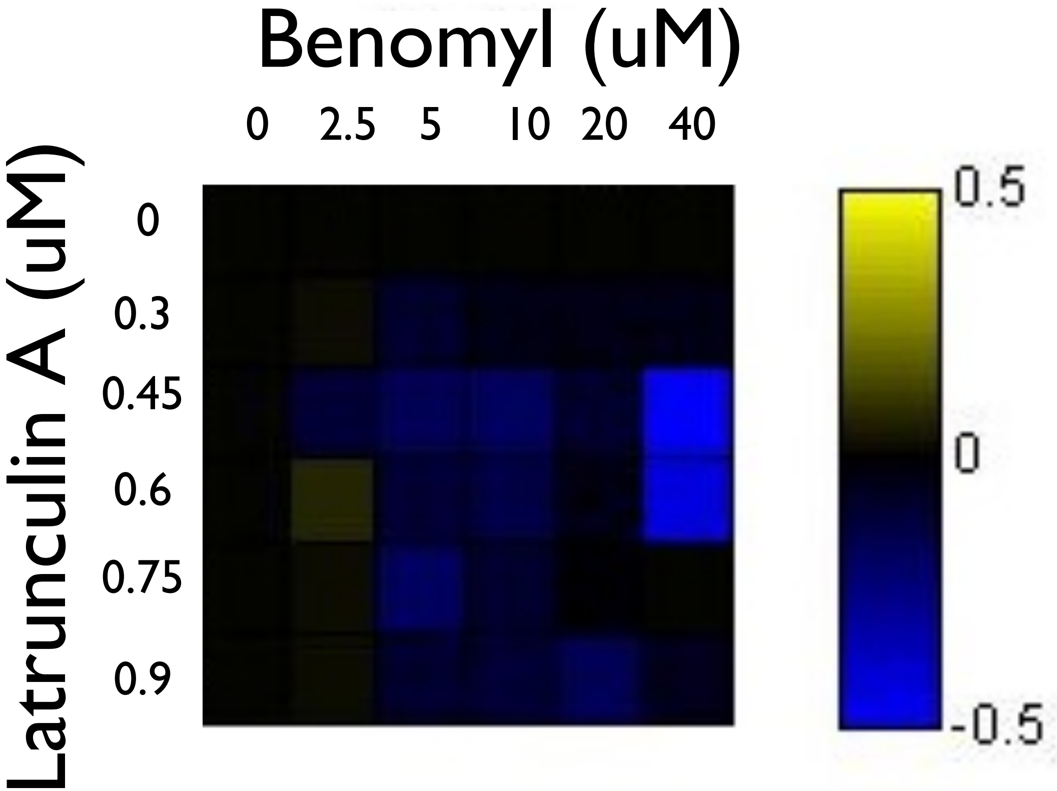
Benomyl (uM)
0 2.5 5 10 20 40
Latrunculin A (uM)
0
0.3
0.45
0.6
0.75
0.9
0.5
0
-0.5

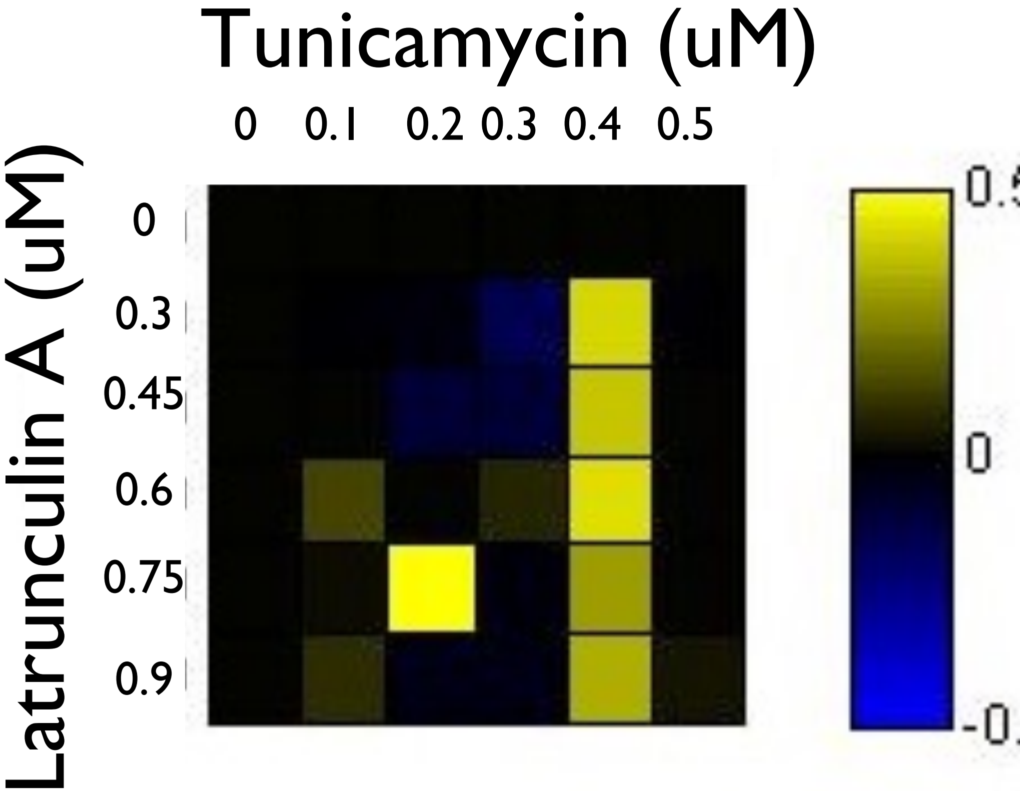
Tunicamycin (uM)
0 0.1 0.2 0.3 0.4 0.5
Latrunculin A (uM)
0
0.3
0.45
0.6
0.75
0.9
0

Repeat 1

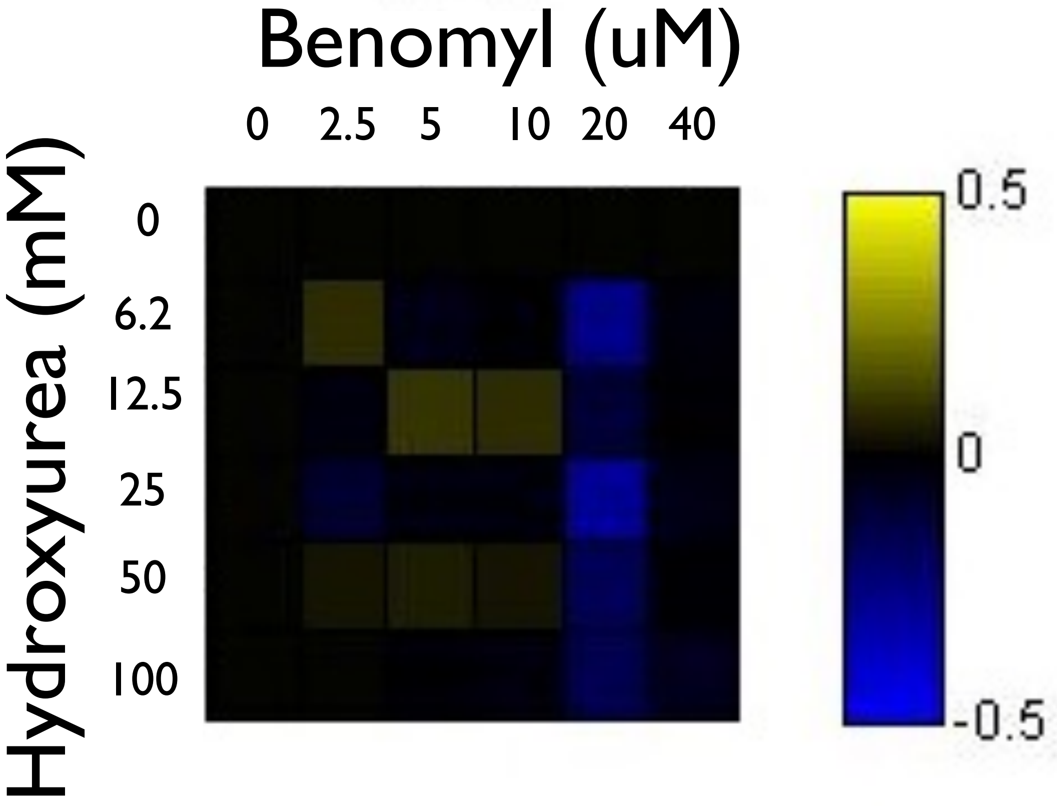
Benomyl (uM)
0 2.5 5 10 20 40
Hydroxyurea (mM)
0
6.2
12.5
25
50
100
0.5
0
-0.5

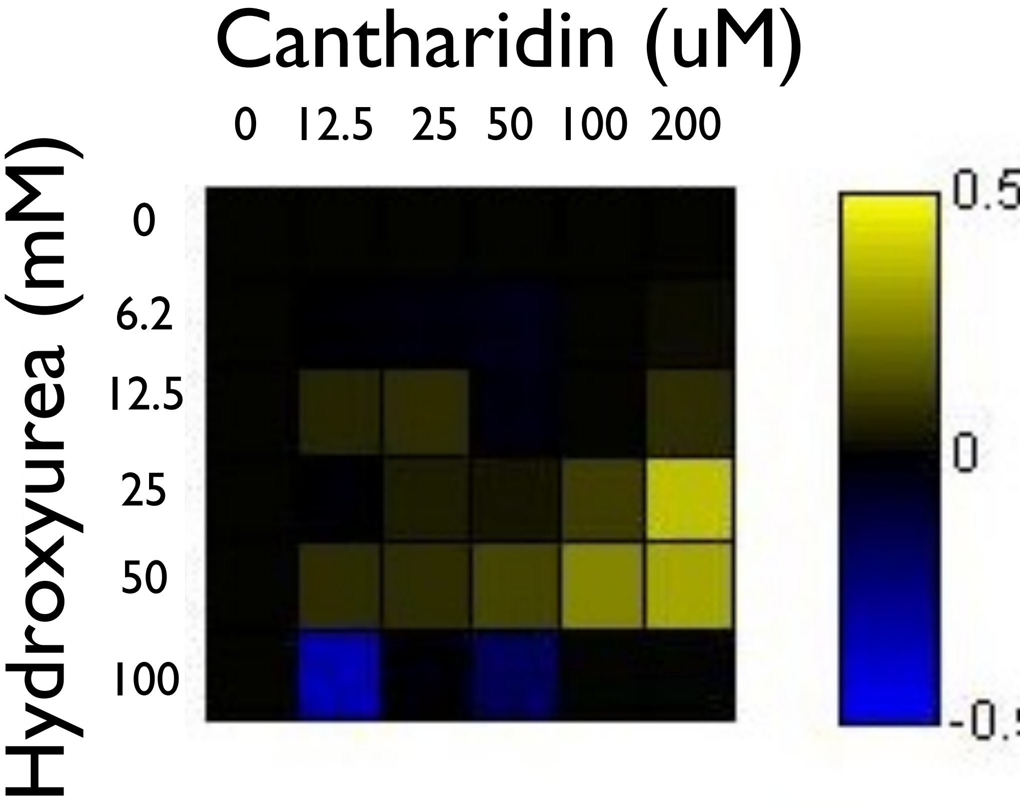
Cantharidin (uM)
0 12.5 25 50 100 200
Hydroxyurea (mM)
0
6.2
12.5
25
50
100
0.5
0

Repeat 1

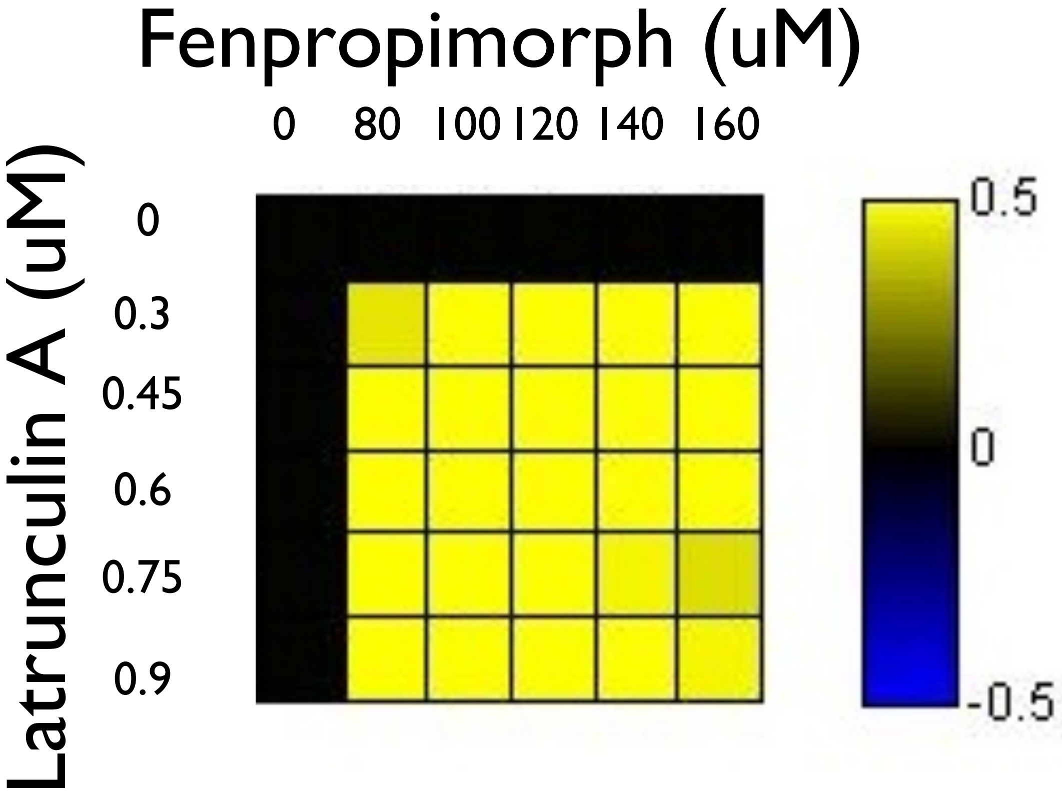
Fenpropimorph (uM)
0 80 100 120 140 160
Latrunculin A (uM)
0 0.3 0.45 0.6 0.75 0.9
0.5
0
-0.5

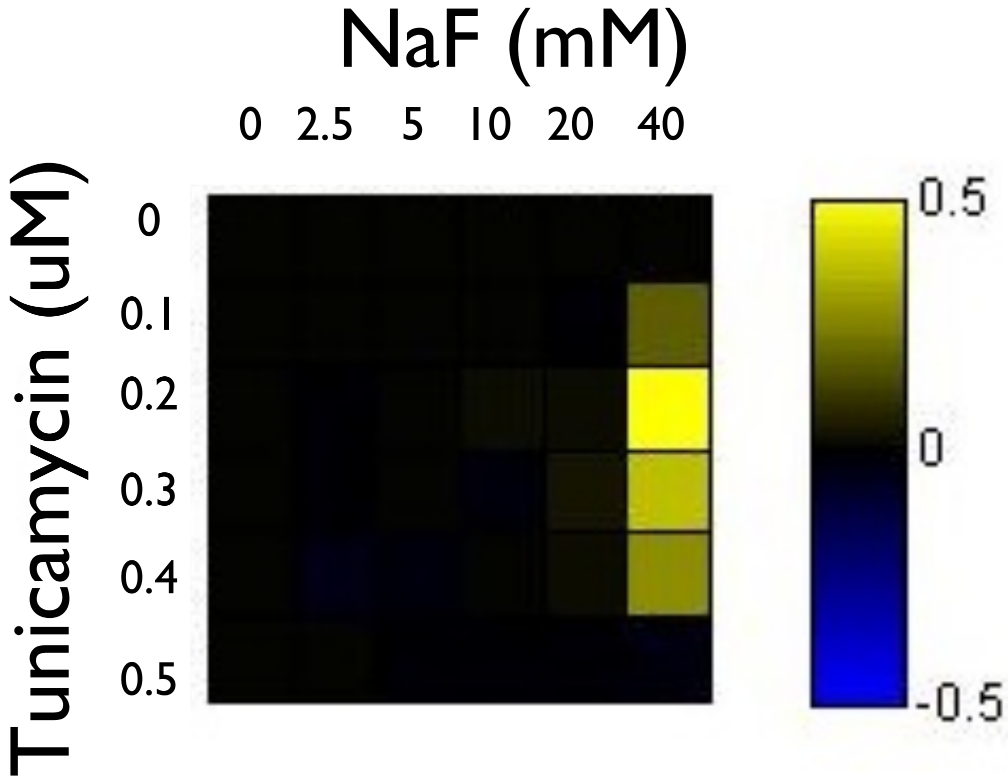
NaF (mM)
0 2.5 5 10 20 40
Tunicamycin (uM)
0 0.1 0.2 0.3 0.4 0.5
0.5
0
-0.5

Repeat 2

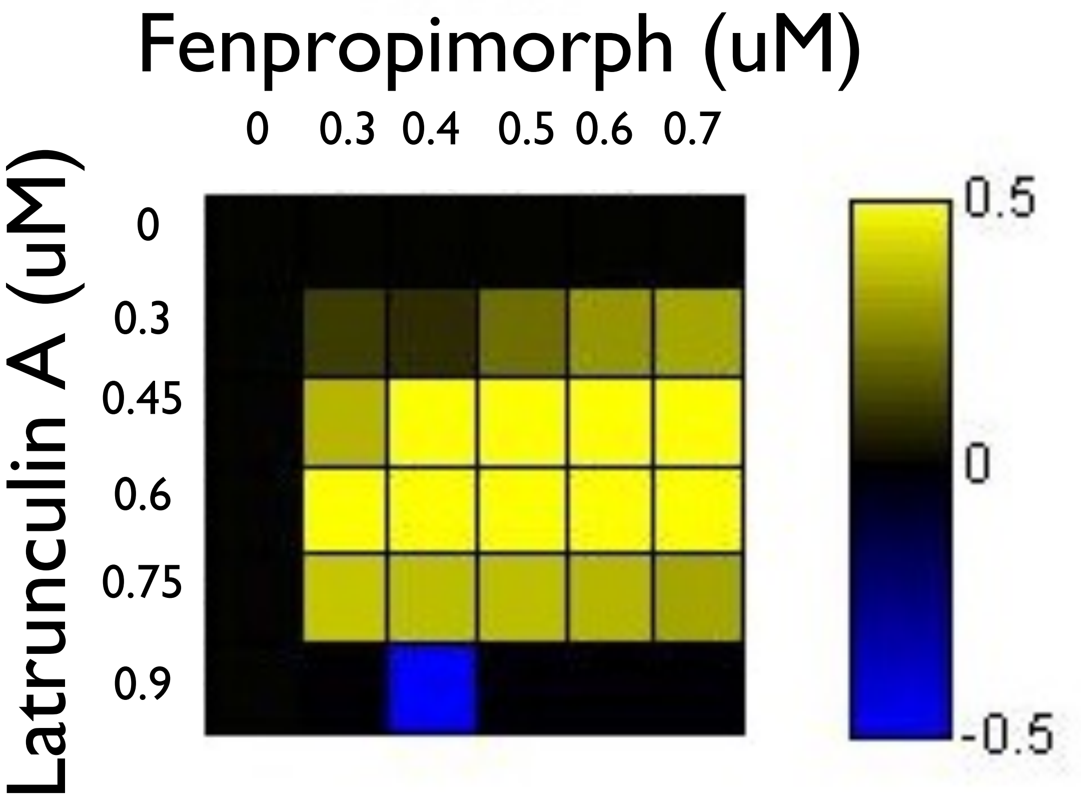

Fenpropimorph (uM)
0
0.3
0.4
0.5
0.6
0.7
Latrunculin A (uM)
0
0.3
0.45
0.6
0.75
0.9
0.5
0
-0.5

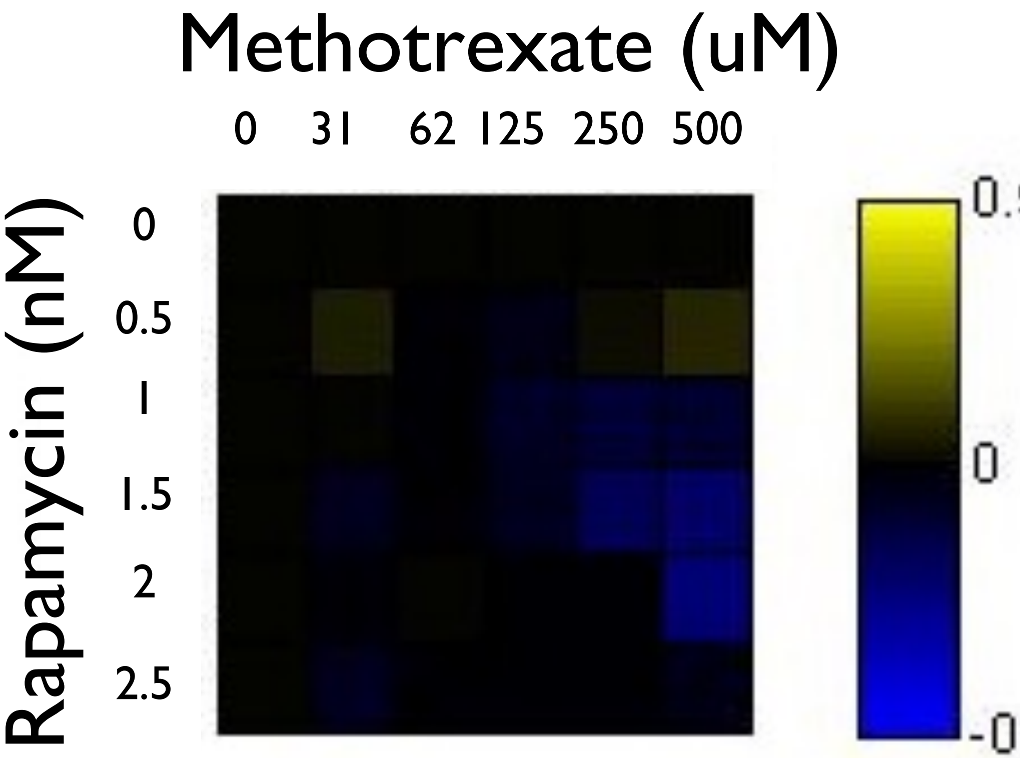

Methotrexate (uM)
0
31
62
125
250
500
Rapamycin (nM)
0
0.5
1
1.5
2
2.5
0

Repeat2

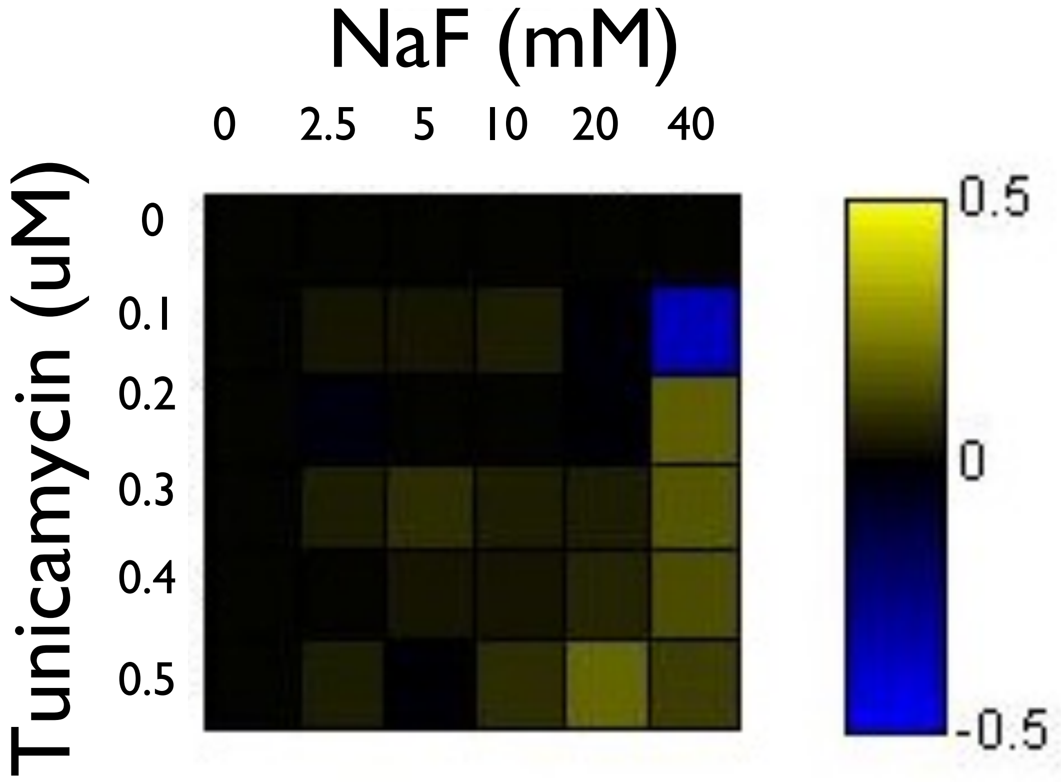
NaF (mM)
0 2.5 5 10 20 40
Tunicamycin (uM)
0
0.1
0.2
0.3
0.4
0.5
0.5
0
-0.5

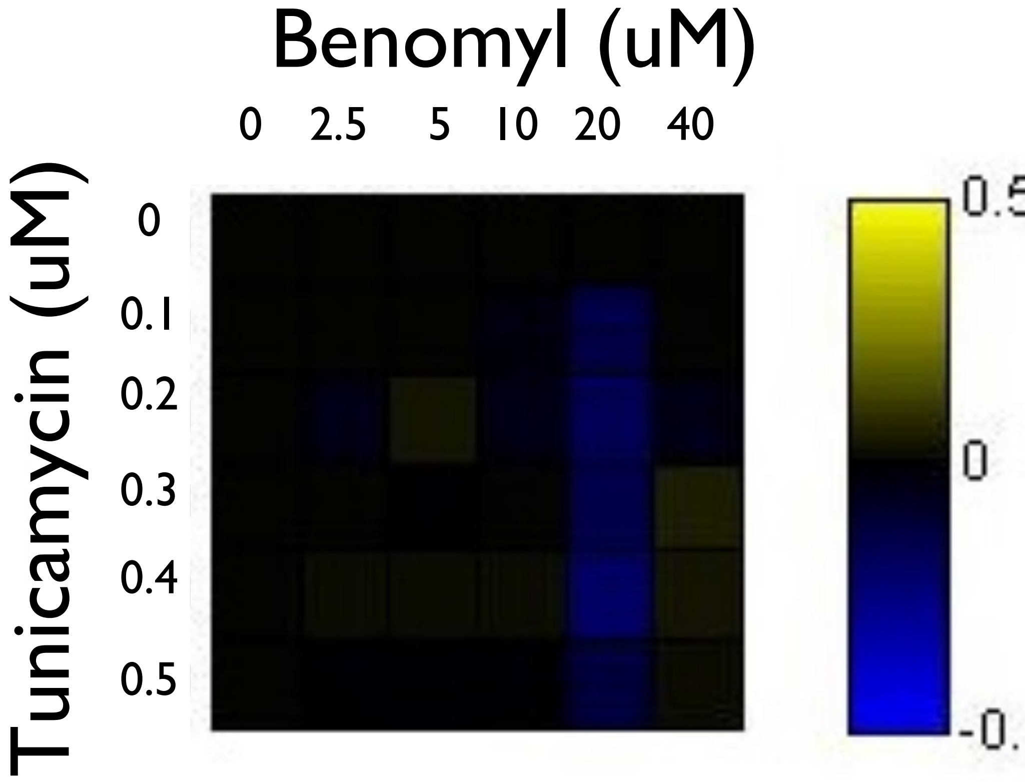
Benomyl (uM)
0 2.5 5 10 20 40
Tunicamycin (uM)
0
0.1
0.2
0.3
0.4
0.5
0
-0.

Repeat 2

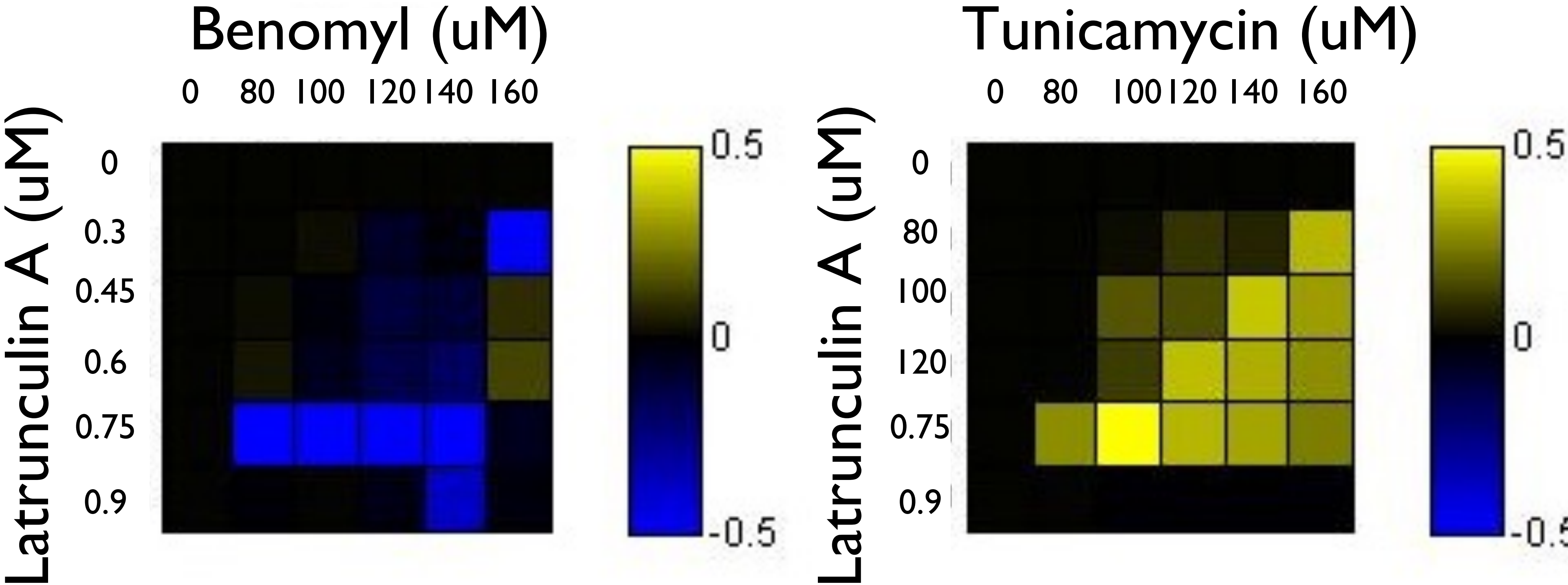
Benomyl (uM)
0 80 100 120 140 160
Latrunculin A (uM)
0
0.3
0.45
0.6
0.75
0.9
0.5
0
-0.5
Tunicamycin (uM)
0 80 100 120 140 160
Latrunculin A (uM)
0
80
100
120
0.75
0.9
0.5
0

Repeat

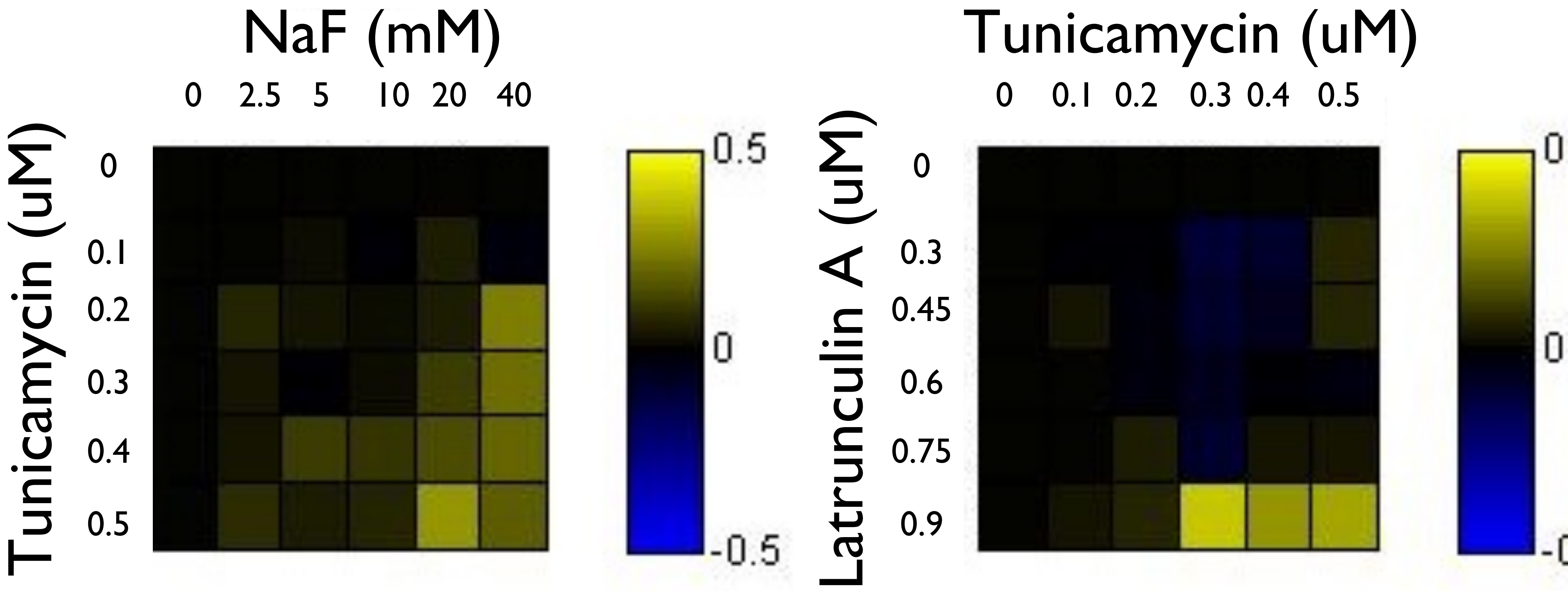

NaF (mM)
0
2.5
5
10
20
40
Tunicamycin (uM)
0
0.1
0.2
0.3
0.4
0.5
0.5
0
-0.5
Tunicamycin (uM)
0
0.1
0.2
0.3
0.4
0.5
Latrunculin A (uM)
0
0.3
0.45
0.6
0.75
0.9

Repeat

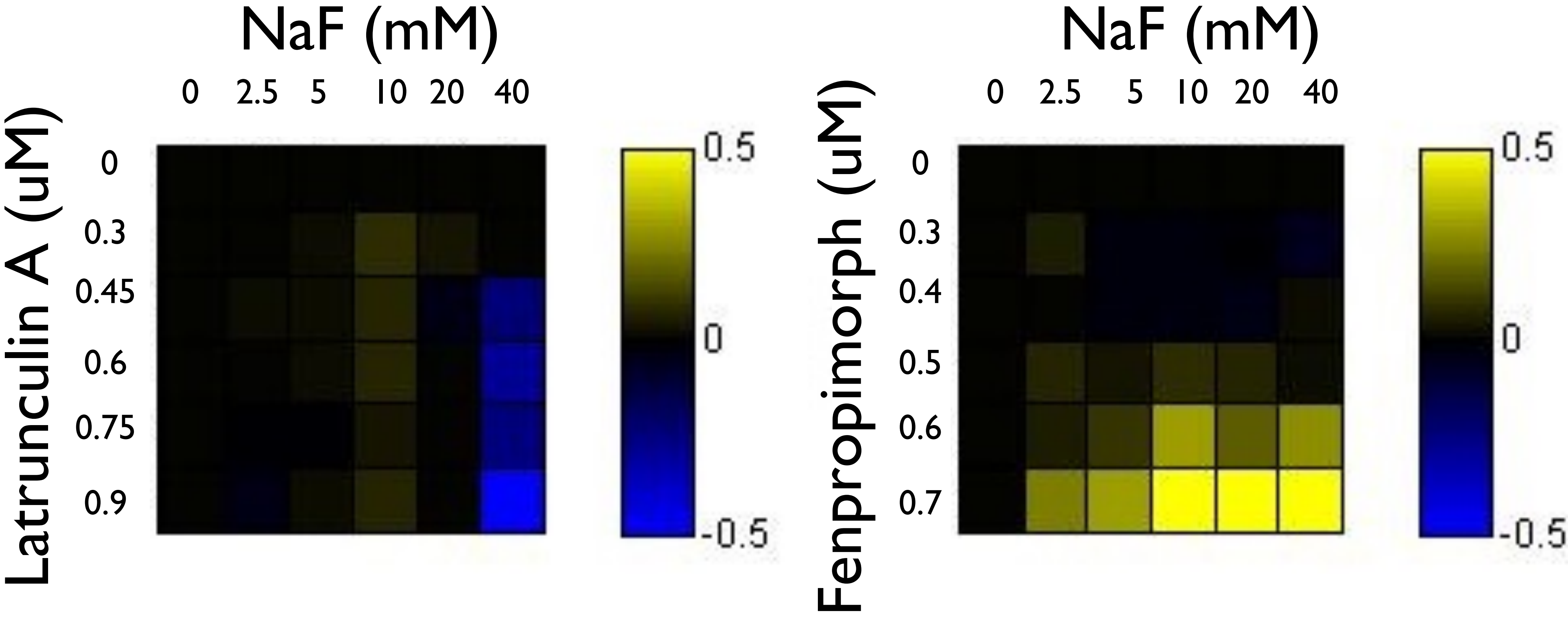
NaF (mM)
0 2.5 5 10 20 40
Latrunculin A (uM)
0
0.3
0.45
0.6
0.75
0.9
0.5
0
-0.5
NaF (mM)
0 2.5 5 10 20 40
Fenpropimorph (uM)
0
0.3
0.4
0.5
0.6
0.7
0.5
0
-0.5

Repeat

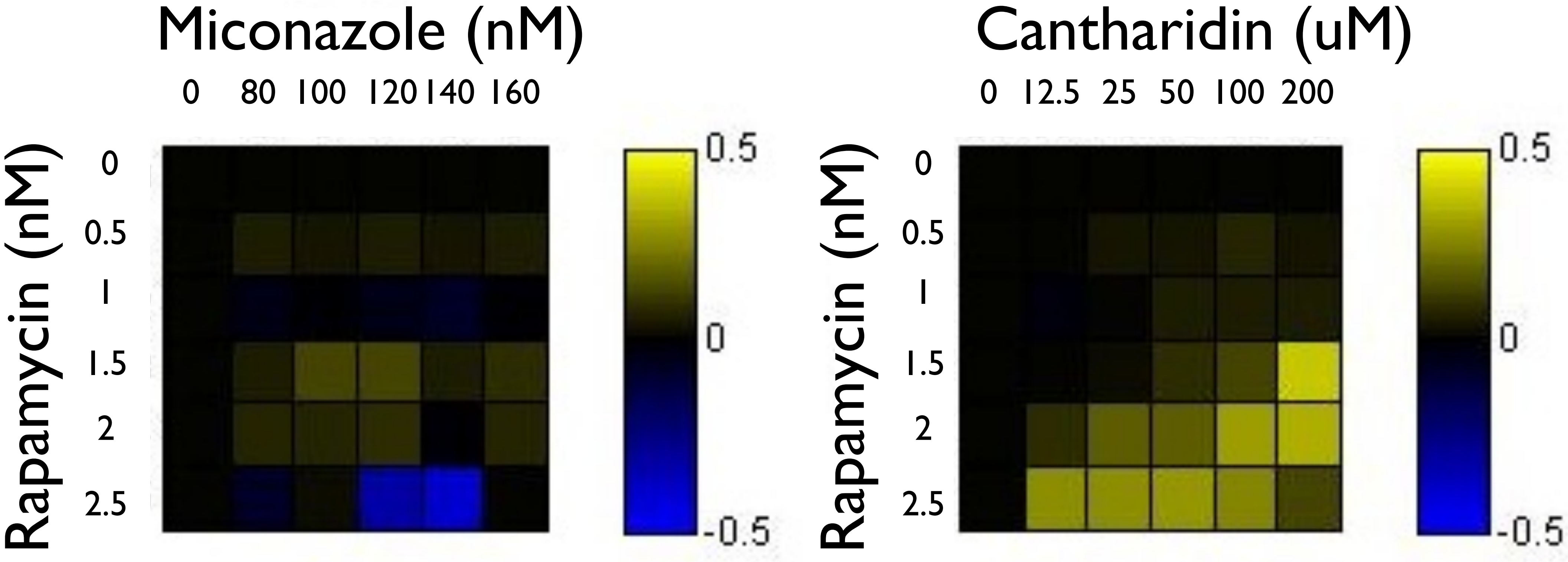
Miconazole (nM)
0 80 100 120 140 160
Rapamycin (nM)
0
0.5
1
1.5
2
2.5
0.5
0
-0.5
Cantharidin (uM)
0 12.5 25 50 100 200
Rapamycin (nM)
0
0.5
1
1.5
2
2.5
0.5
0
-0.5

Repeat

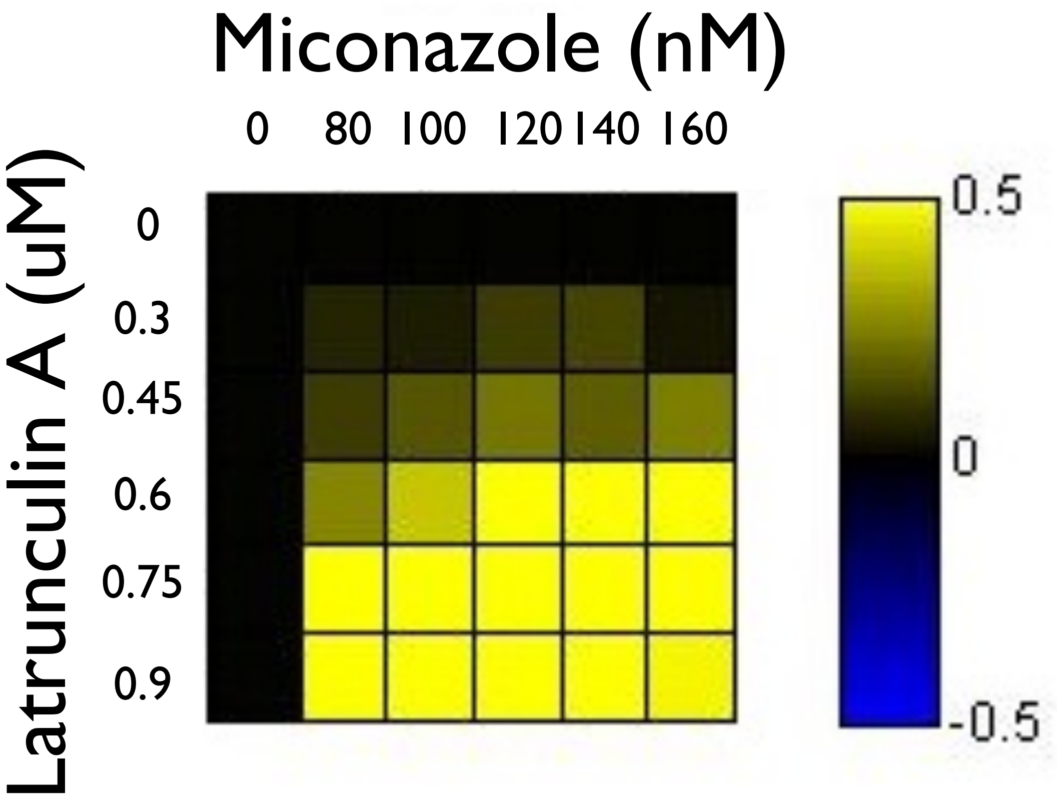

Miconazole (nM)
0
80
100
120
140
160
Latrunculin A (uM)
0
0.3
0.45
0.6
0.75
0.9
0.5
0
-0.5

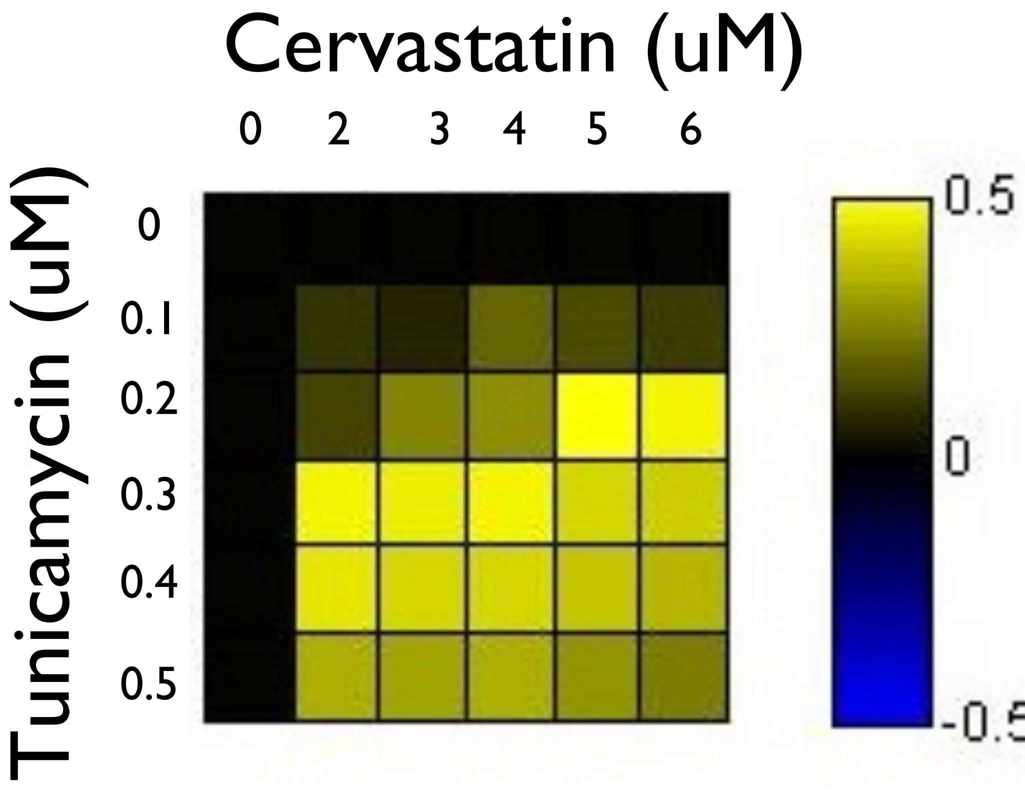

Cervastatin (uM)
0
2
3
4
5
6
Tunicamycin (uM)
0
0.1
0.2
0.3
0.4
0.5
0.5
0

Repeat

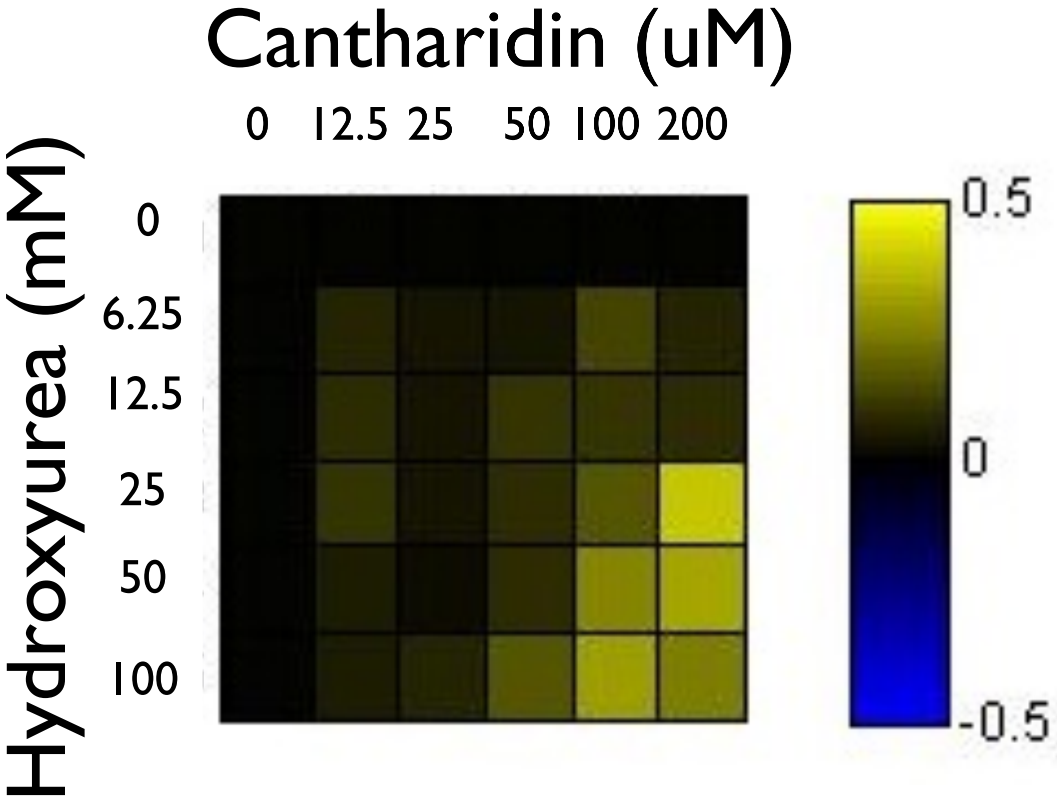
Cantharidin (uM)
0 12.5 25 50 100 200
Hydroxyurea (mM)
0
6.25
12.5
25
50
100
0.5
0
-0.5

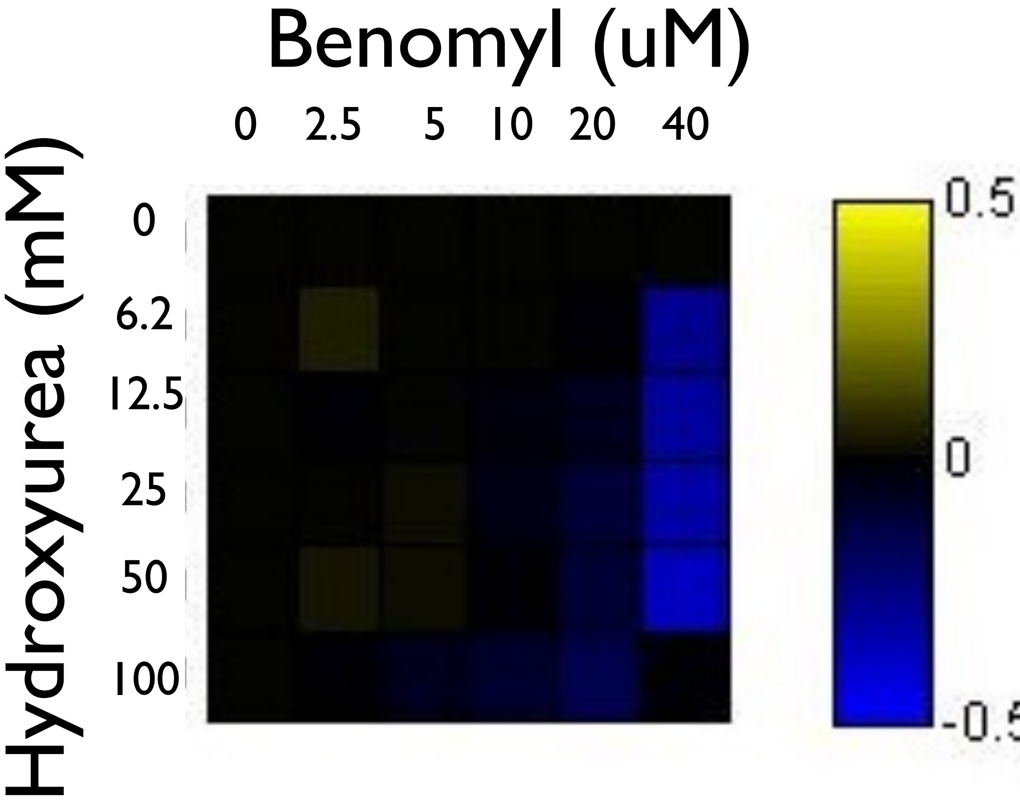
Benomyl (uM)
0 2.5 5 10 20 40
Hydroxyurea (mM)
0
6.2
12.5
25
50
100
0.5
0

Repeat

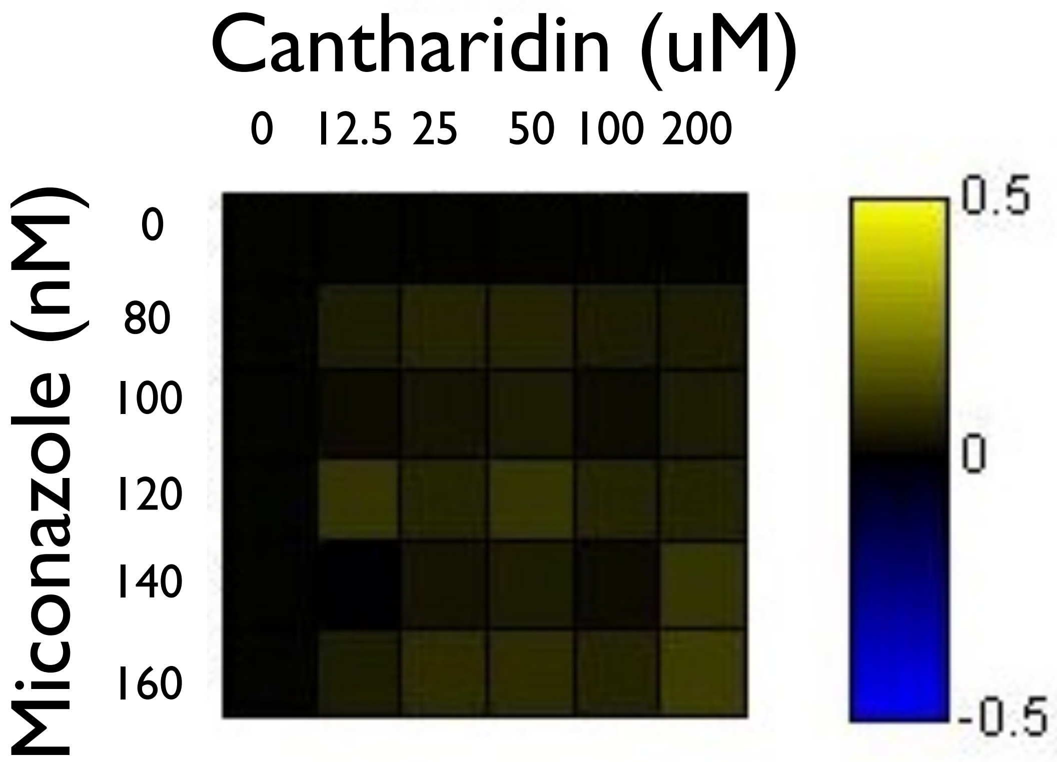

Cantharidin (uM)
0 12.5 25 50 100 200
Miconazole (nM)
0
80
100
120
140
160
0.5
0
-0.5

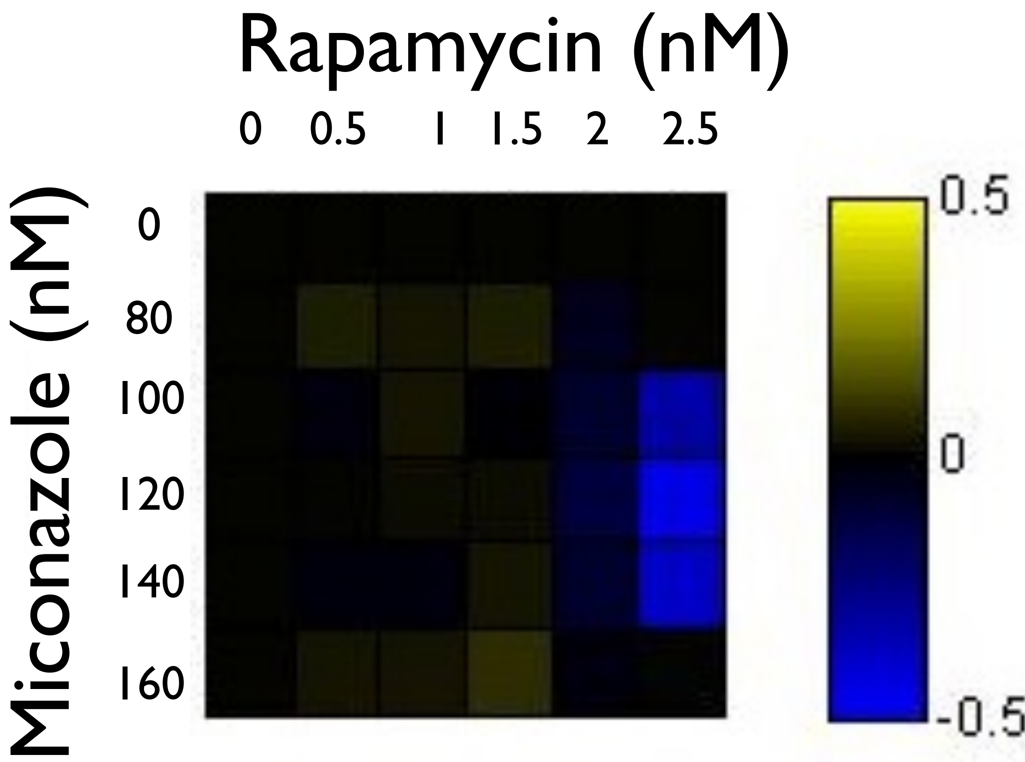

Rapamycin (nM)
0 0.5 1 1.5 2 2.5
Miconazole (nM)
0
80
100
120
140
160
0.5
0
-0.5

Repeat

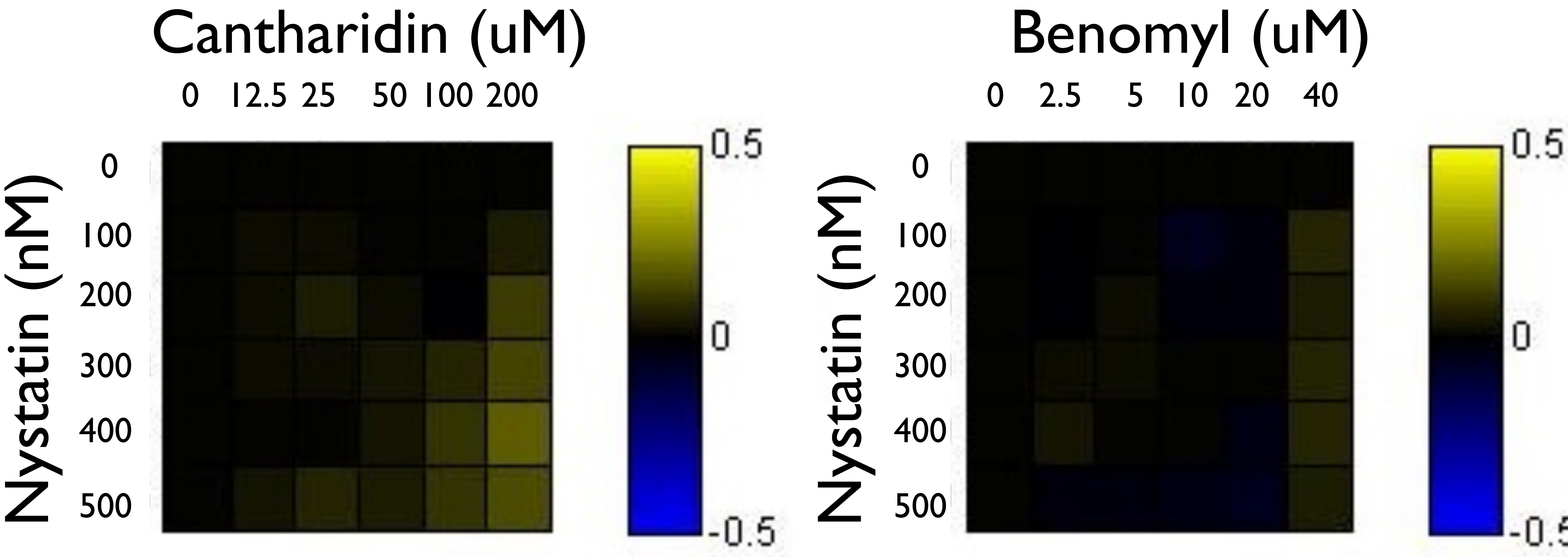
Cantharidin (uM)
0 12.5 25 50 100 200
Nystatin (nM)
0
100
200
300
400
500
0.5
0
-0.5
Benomyl (uM)
0 2.5 5 10 20 40
Nystatin (nM)
0
100
200
300
400
500
0.5
0

Repeat

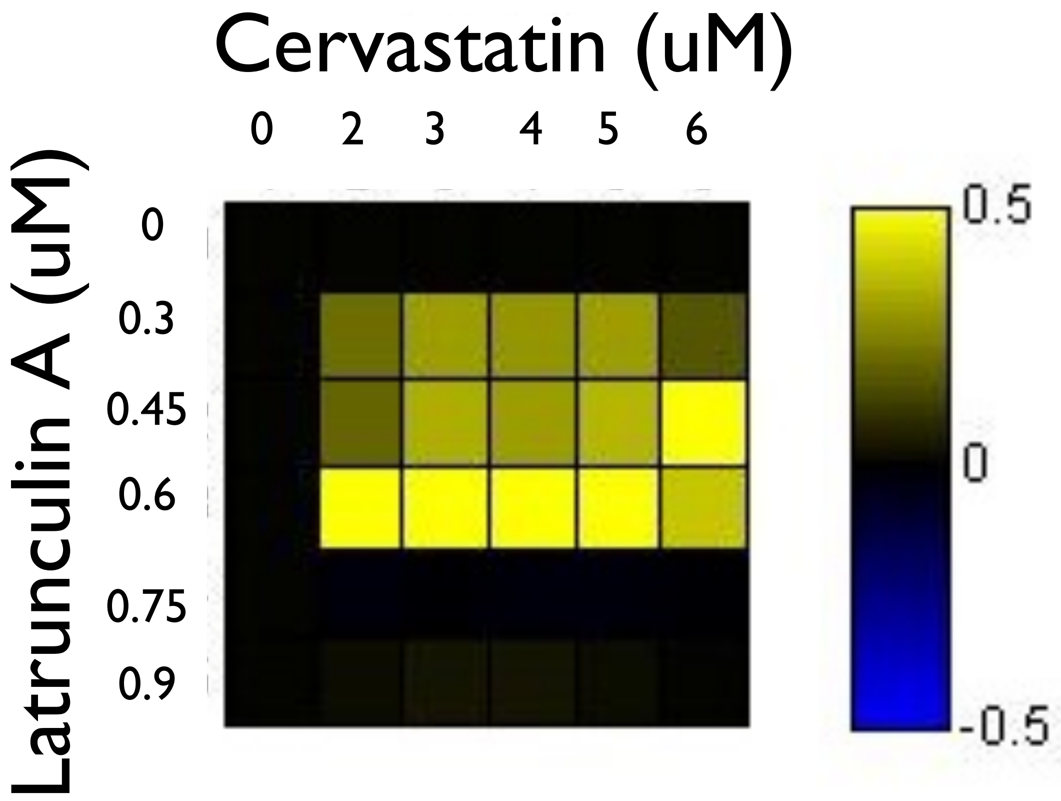

Cervastatin (uM)
0 2 3 4 5 6
Latrunculin A (uM)
0
0.3
0.45
0.6
0.75
0.9
0.5
0
-0.5

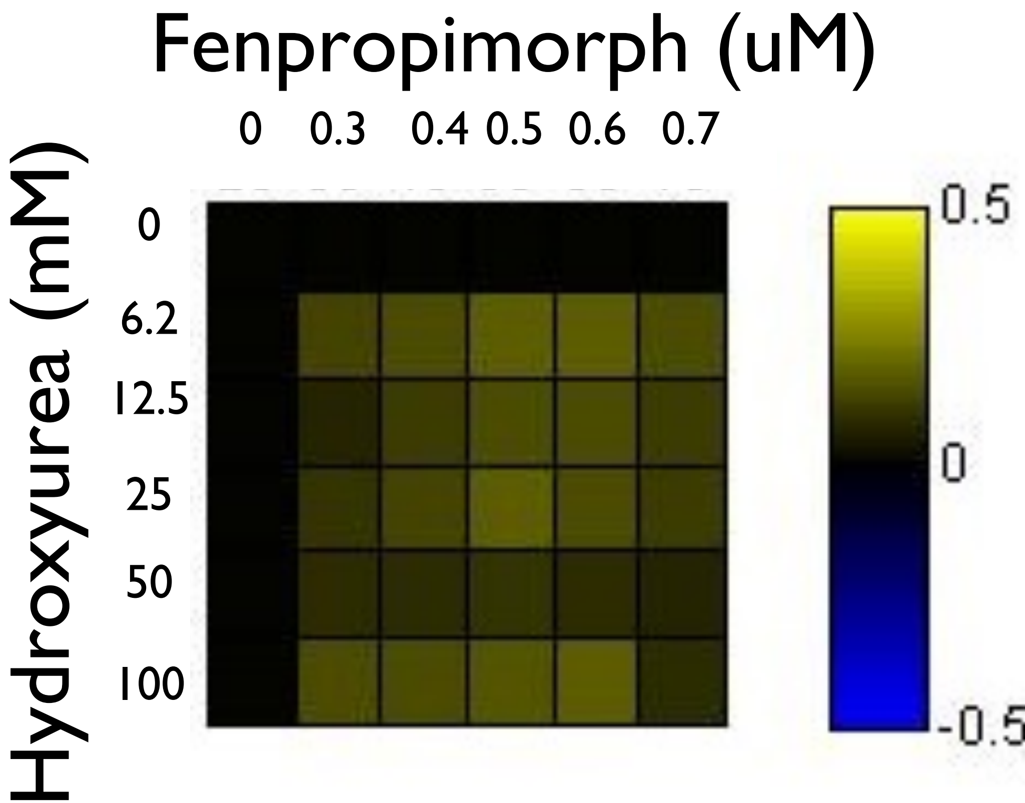

Fenpropimorph (uM)
0 0.3 0.4 0.5 0.6 0.7
Hydroxyurea (mM)
0
6.2
12.5
25
50
100
0.5
0
-0.5

Repeat - Lower Dosage

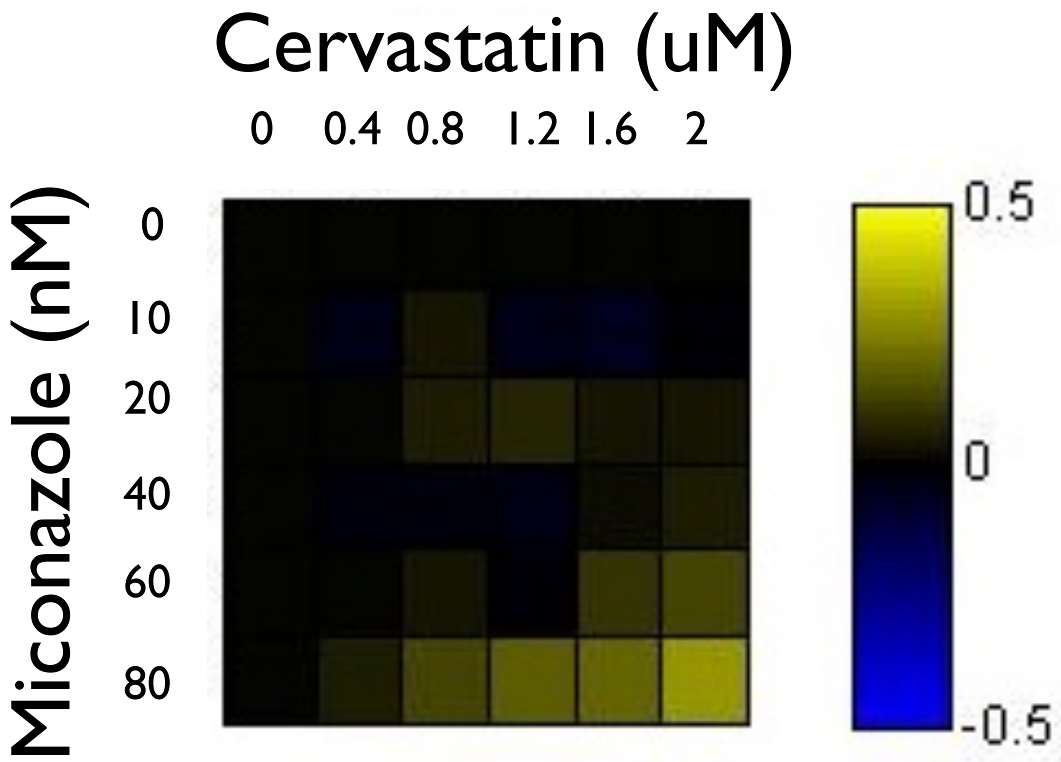

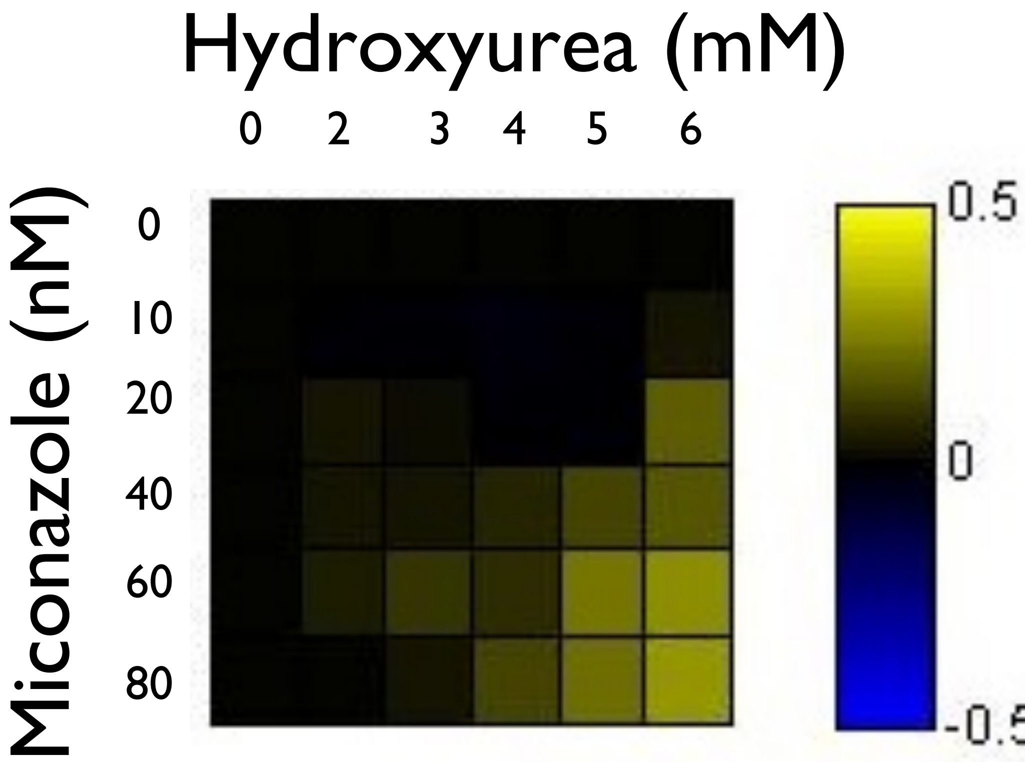

Repeat

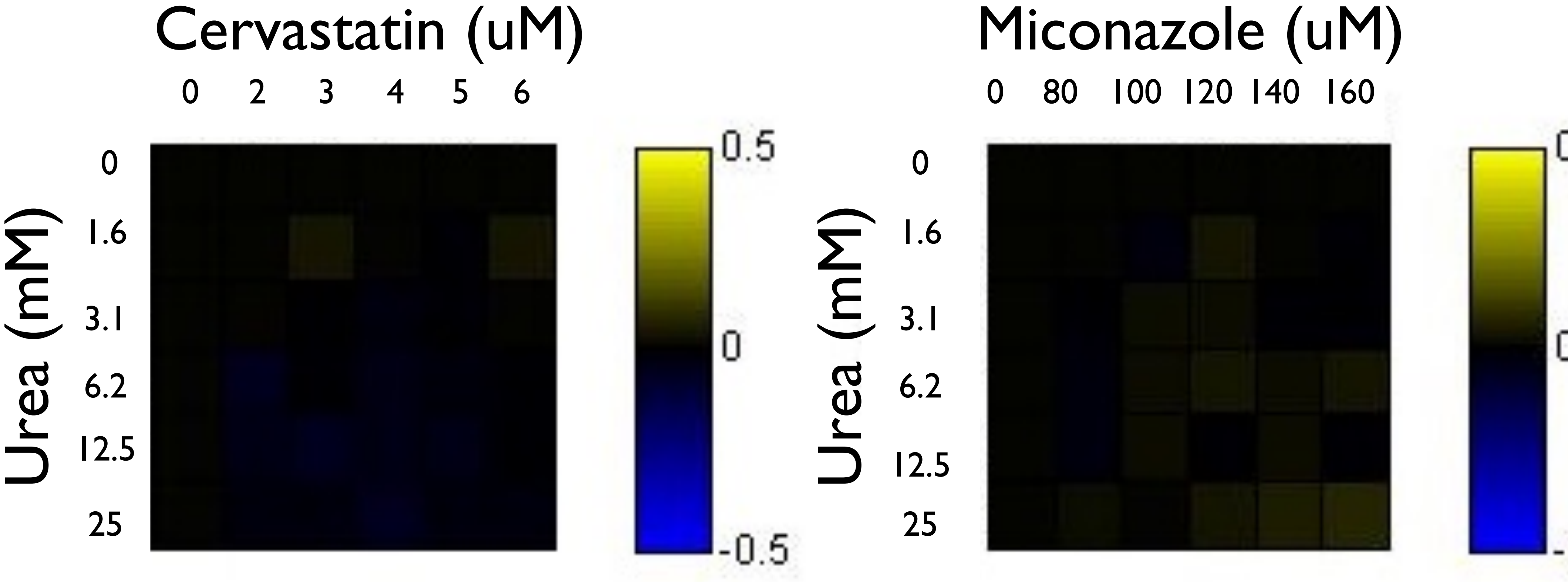

Cervastatin (uM)
0 2 3 4 5 6
Urea (mM)
0
1.6
3.1
6.2
12.5
25
0.5
0
-0.5
Miconazole (uM)
0 80 100 120 140 160
Urea (mM)
0
1.6
3.1
6.2
12.5
25

Repeat

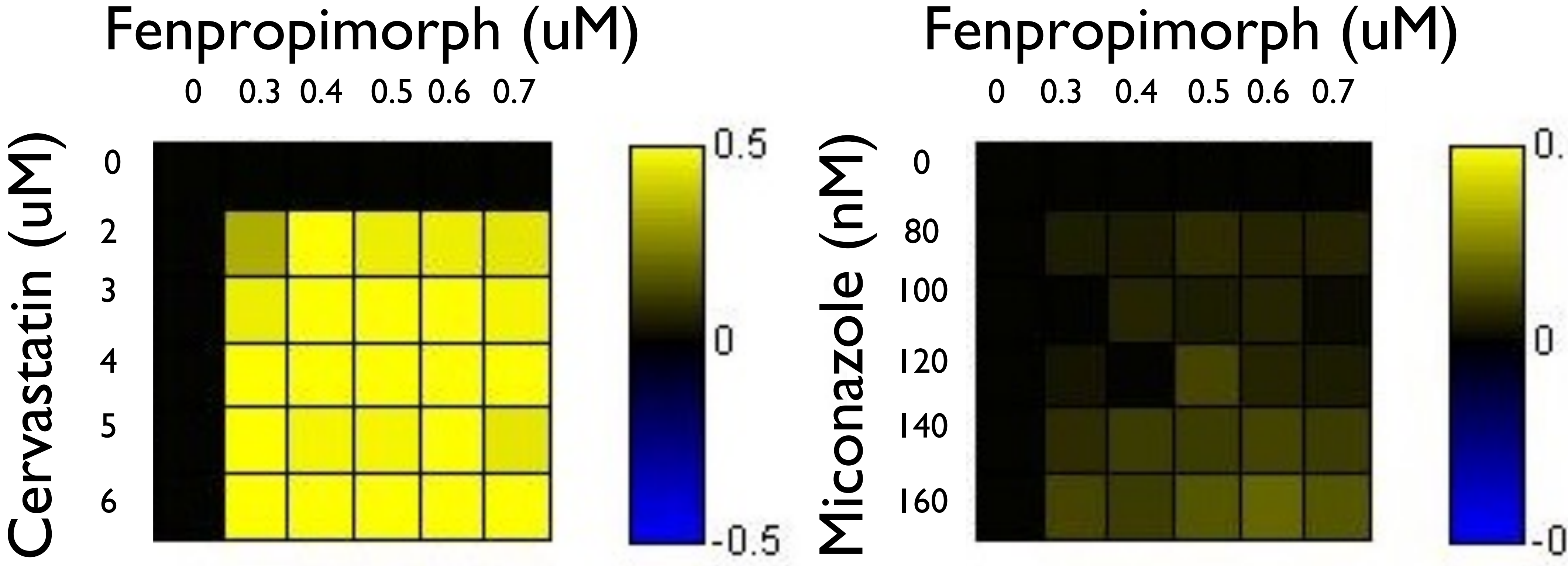
Fenpropimorph (uM)
0 0.3 0.4 0.5 0.6 0.7
Cervastatin (uM)
0
2
3
4
5
6
0.5
0
-0.5
Fenpropimorph (uM)
0 0.3 0.4 0.5 0.6 0.7
Miconazole (nM)
0
80
100
120
140
160
0

Repeat - Lower Dosage

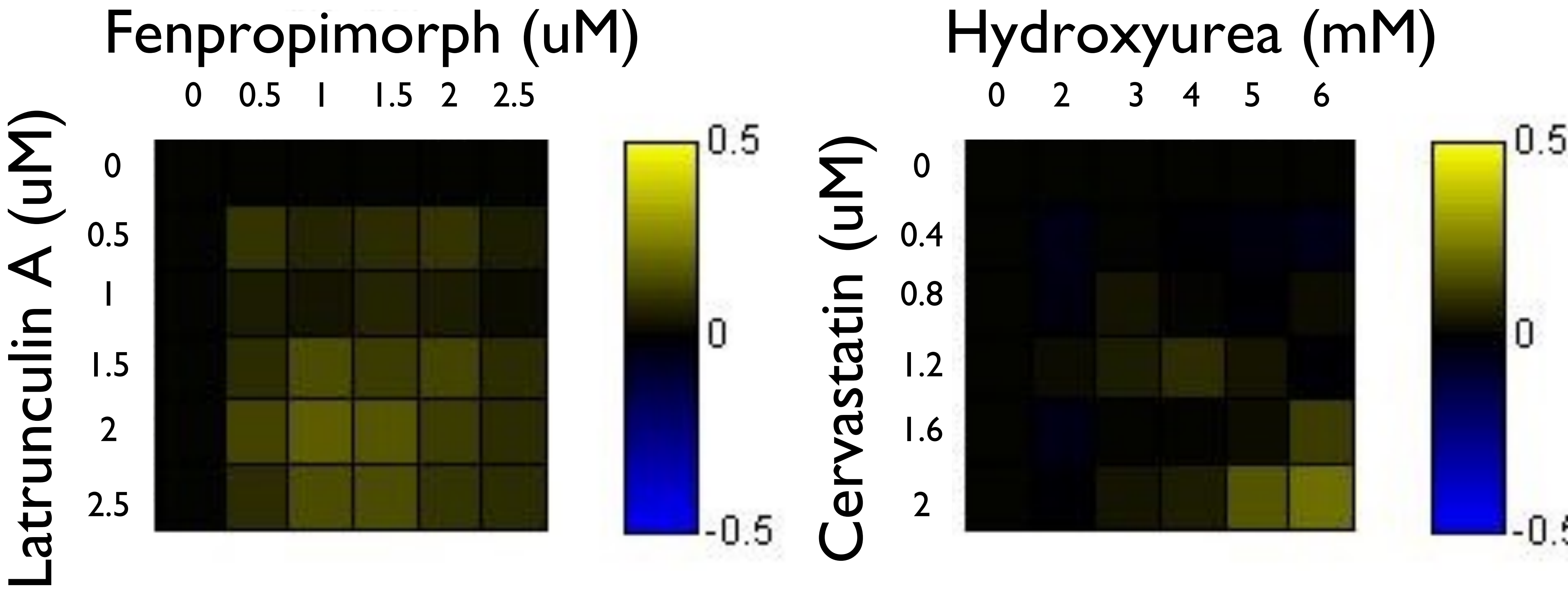

Repeat

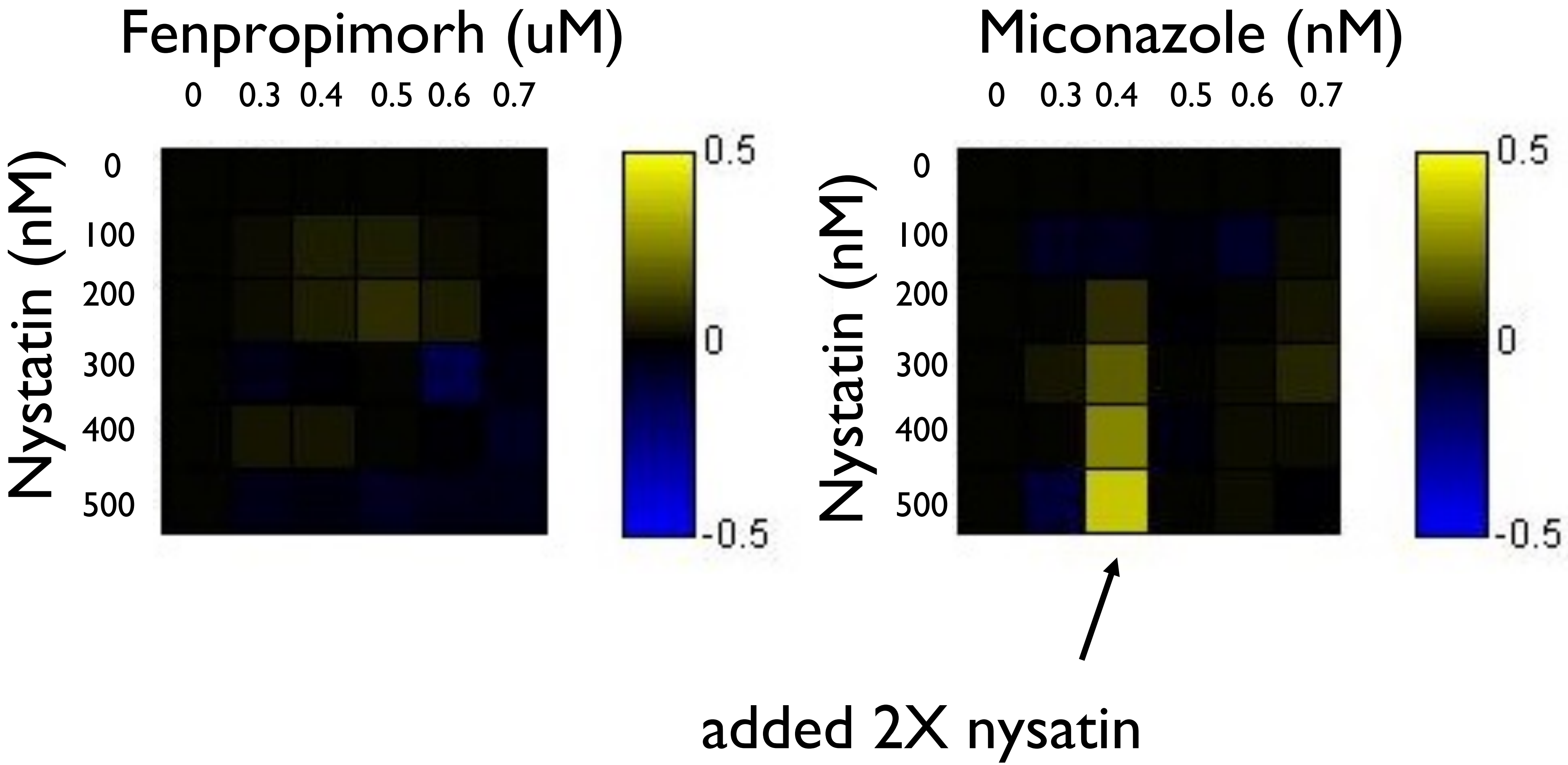
Fenpropimorh (uM)
0 0.3 0.4 0.5 0.6 0.7
Nystatin (nM)
0
100
200
300
400
500
0.5
0
-0.5
Miconazole (nM)
0 0.3 0.4 0.5 0.6 0.7
Nystatin (nM)
0
100
200
300
400
500
0.5
0
-0.5
added 2X nysatin

Repeat

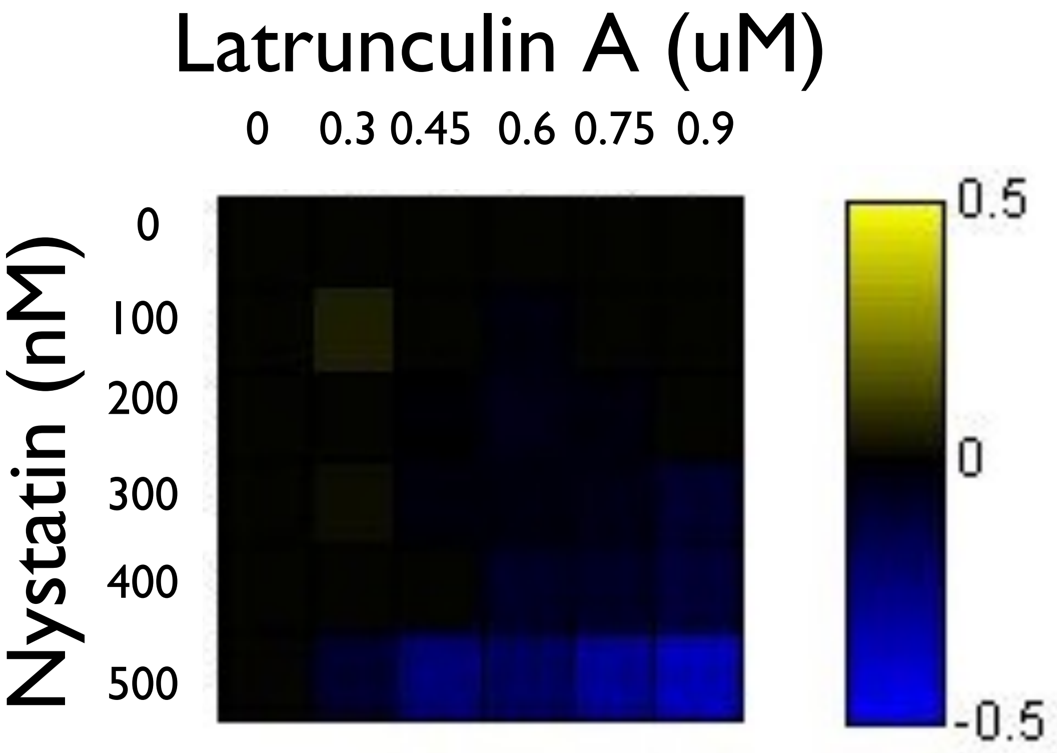
Latrunculin A (uM)
0 0.3 0.45 0.6 0.75 0.9
Nystatin (nM)
0
100
200
300
400
500
0.5
0
-0.5

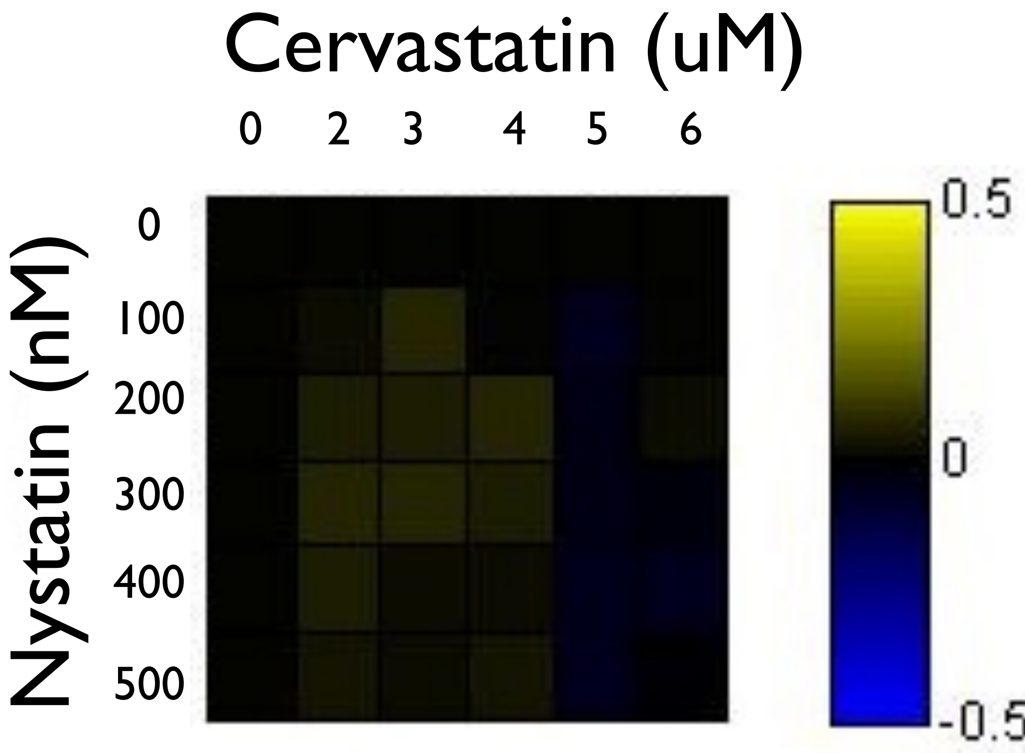
Cervastatin (uM)
0 2 3 4 5 6
Nystatin (nM)
0
100
200
300
400
500
0.5
0
-0.5

Repeat

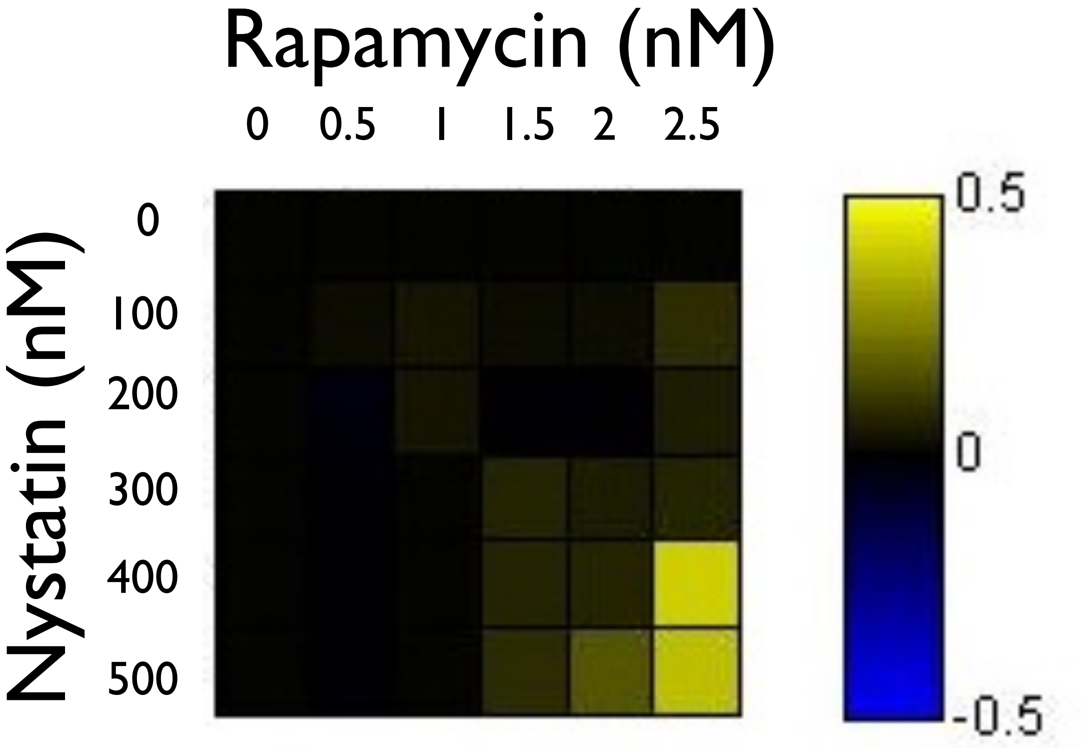

Rapamycin (nM)
0
0.5
1
1.5
2
2.5
Nystatin (nM)
0
100
200
300
400
500
0.5
0
-0.5

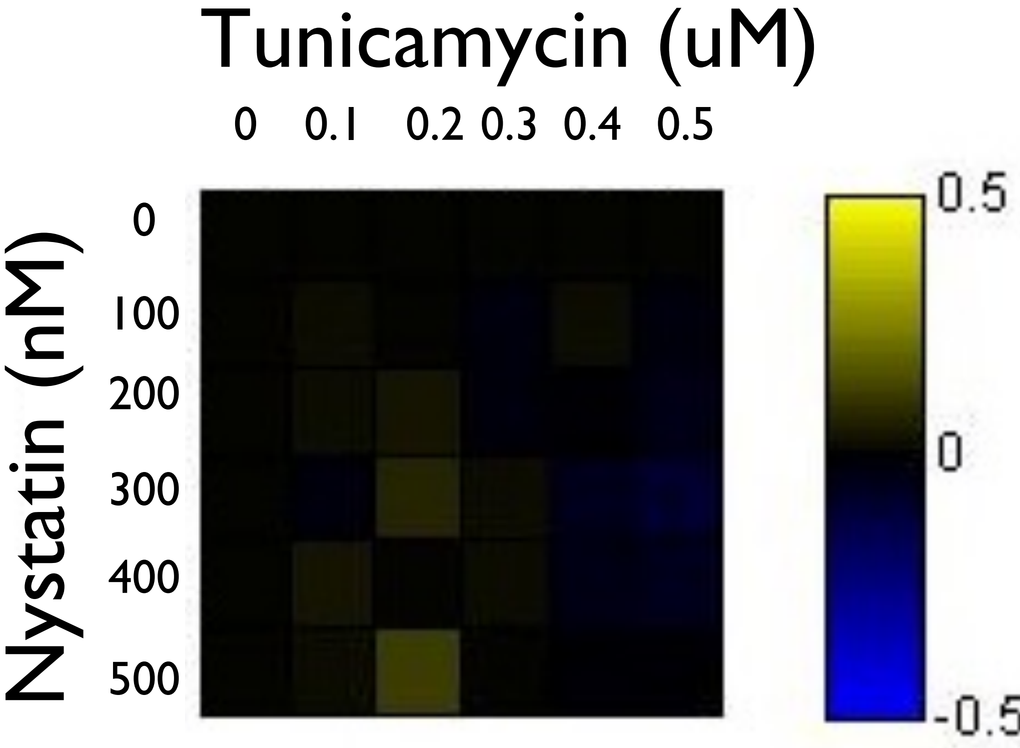

Tunicamycin (uM)
0
0.1
0.2
0.3
0.4
0.5
Nystatin (nM)
0
100
200
300
400
500
0.5
0
-0.5

Repeat - Lower Dosage

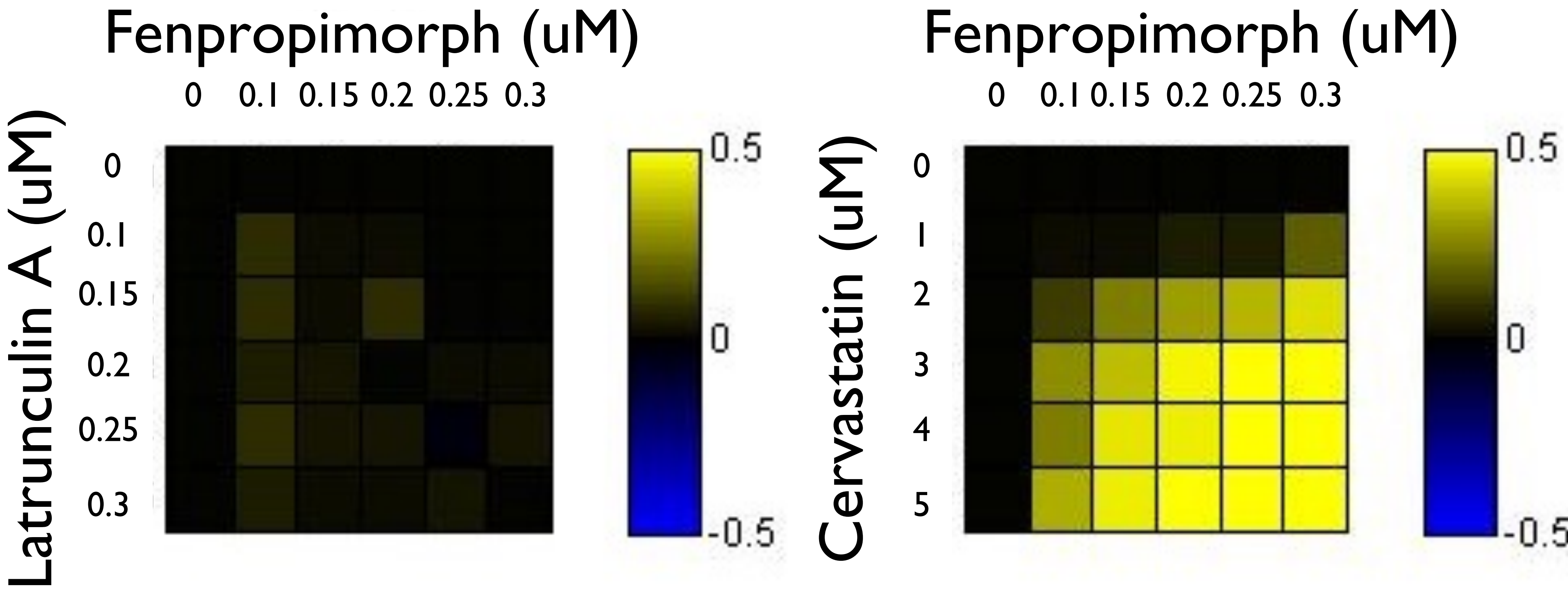

Repeat - Lower Dosage

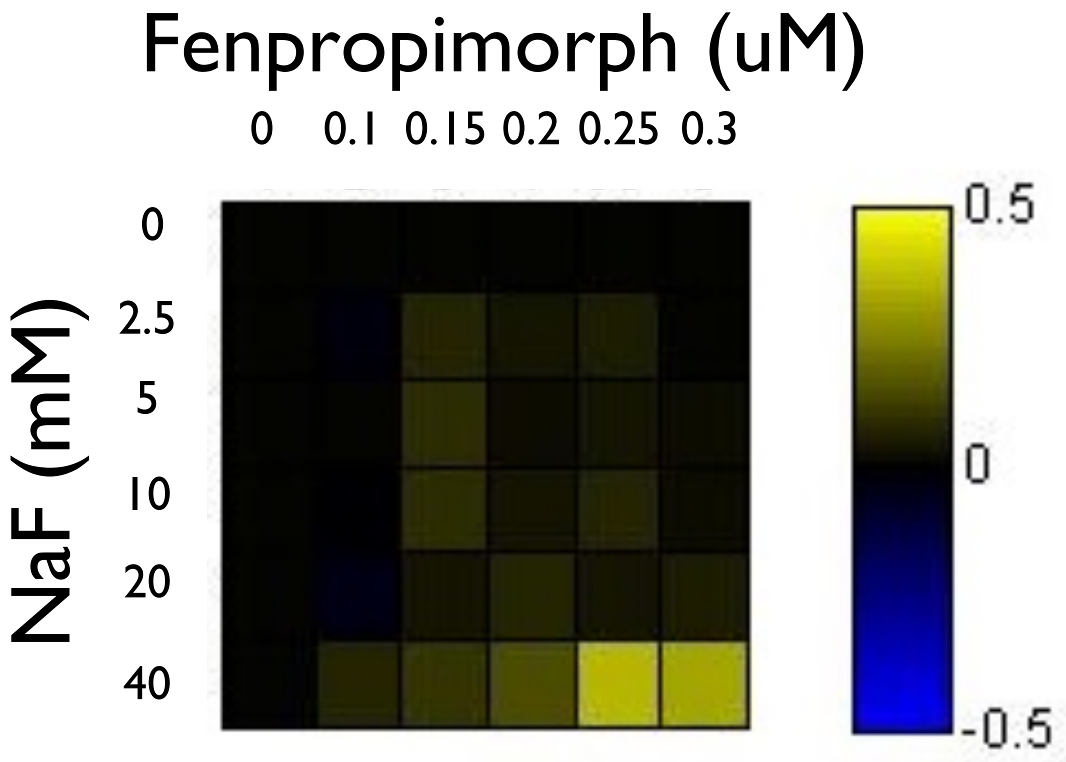

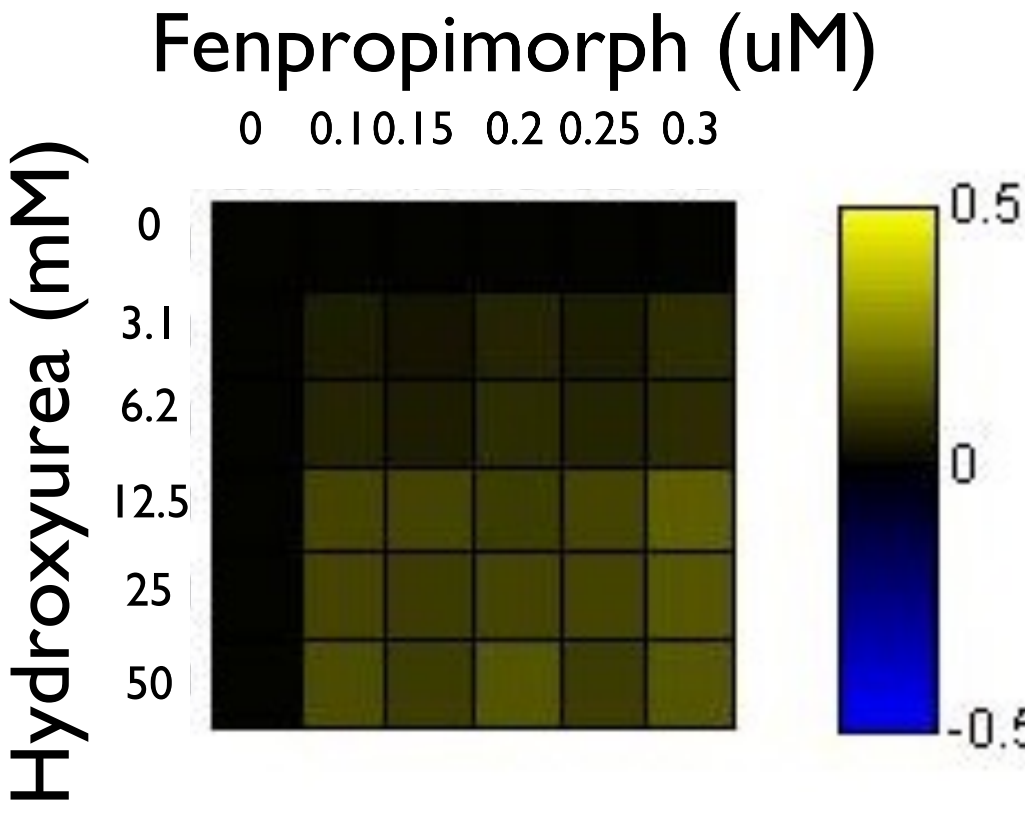

Repeat - Lower Dosage

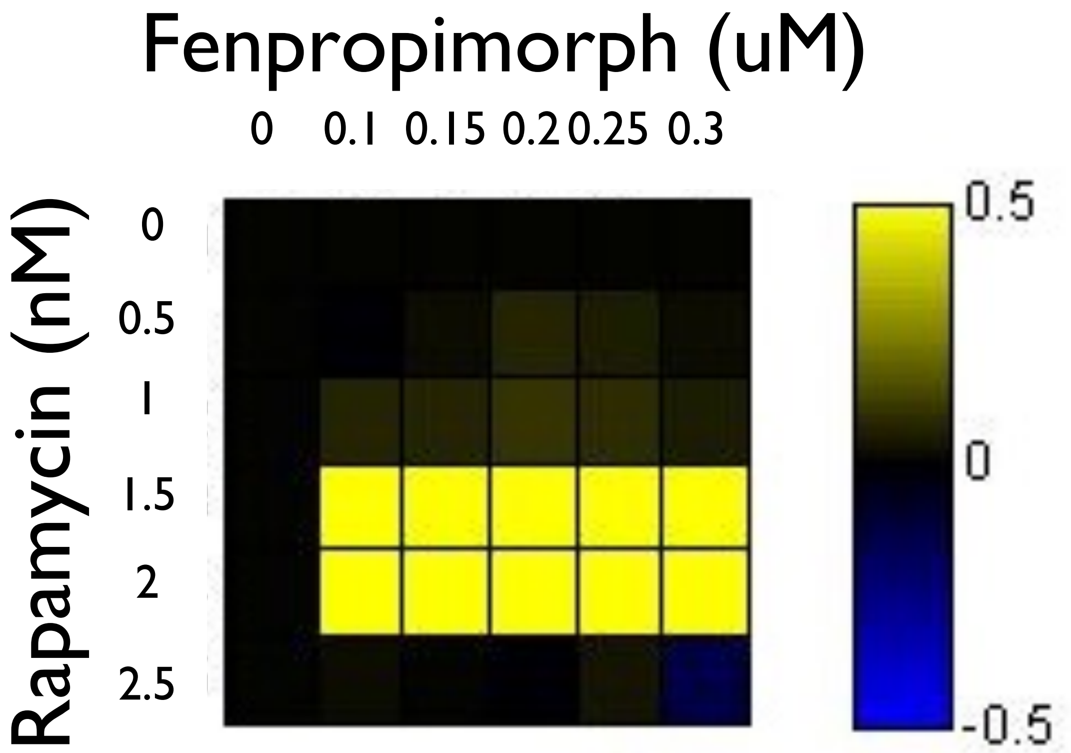

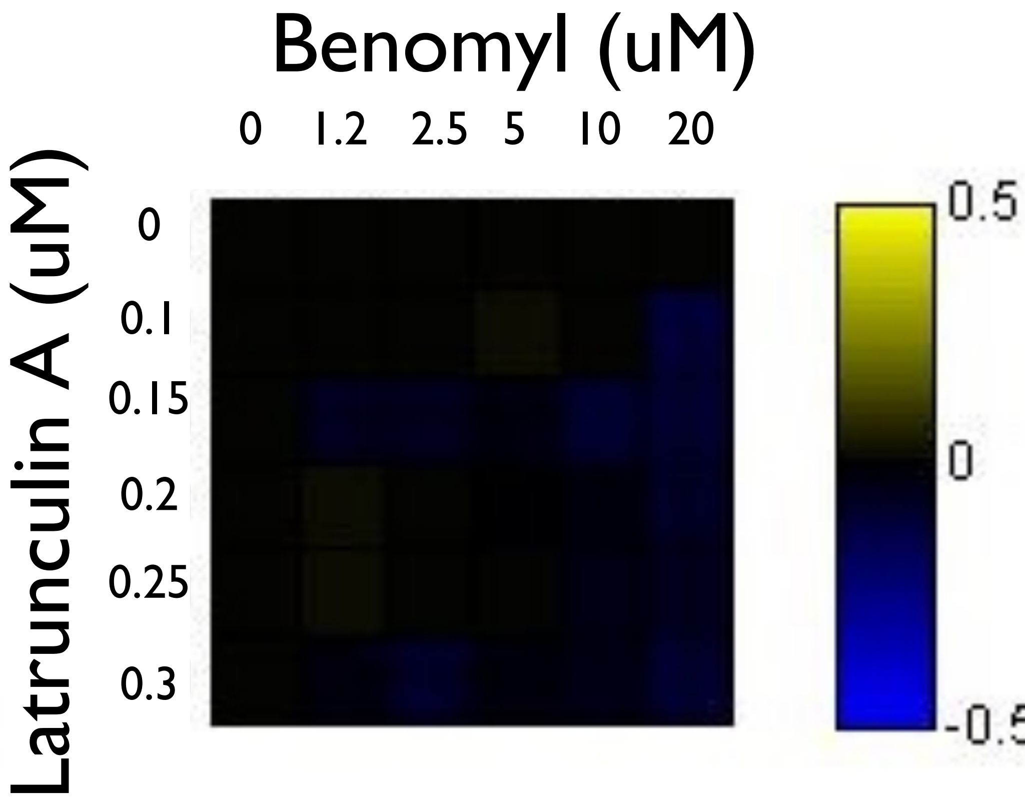

Repeat - Lower Dosage

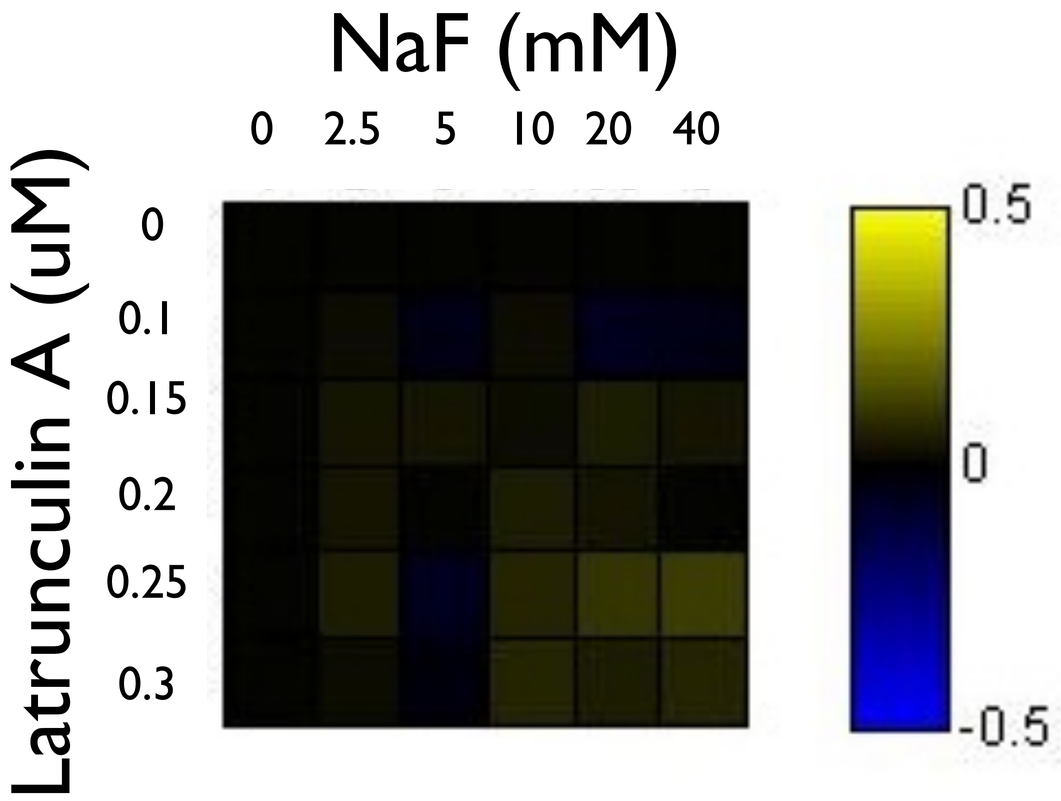

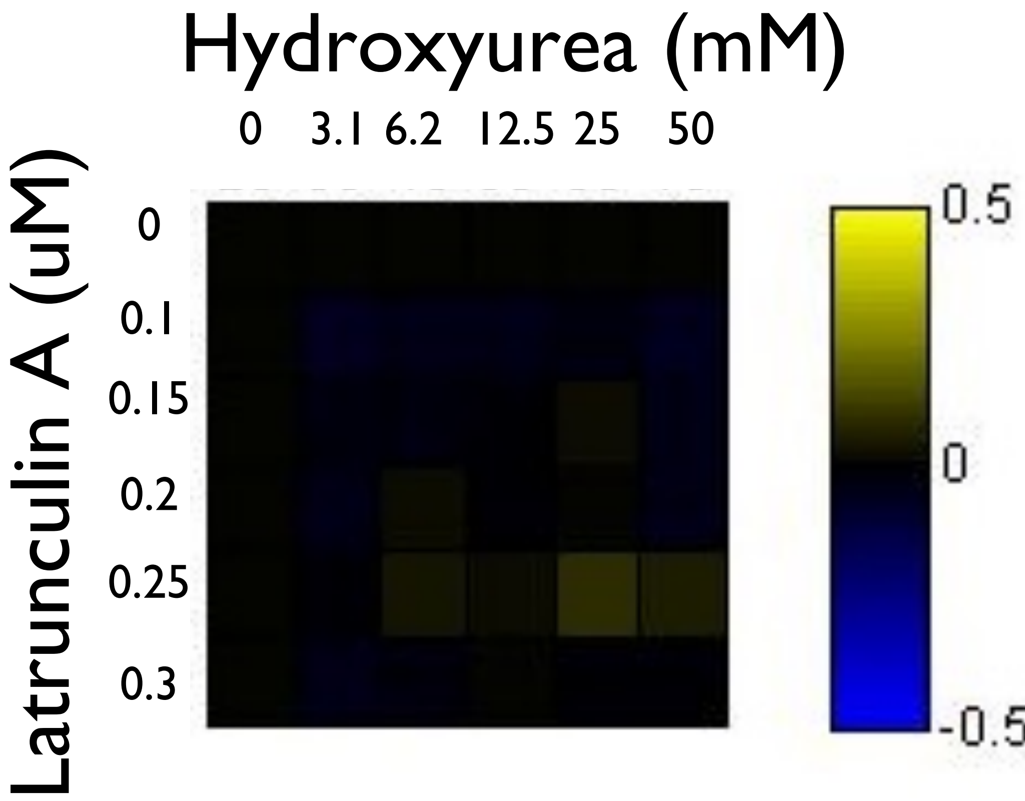

Repeat - Lower Dosage

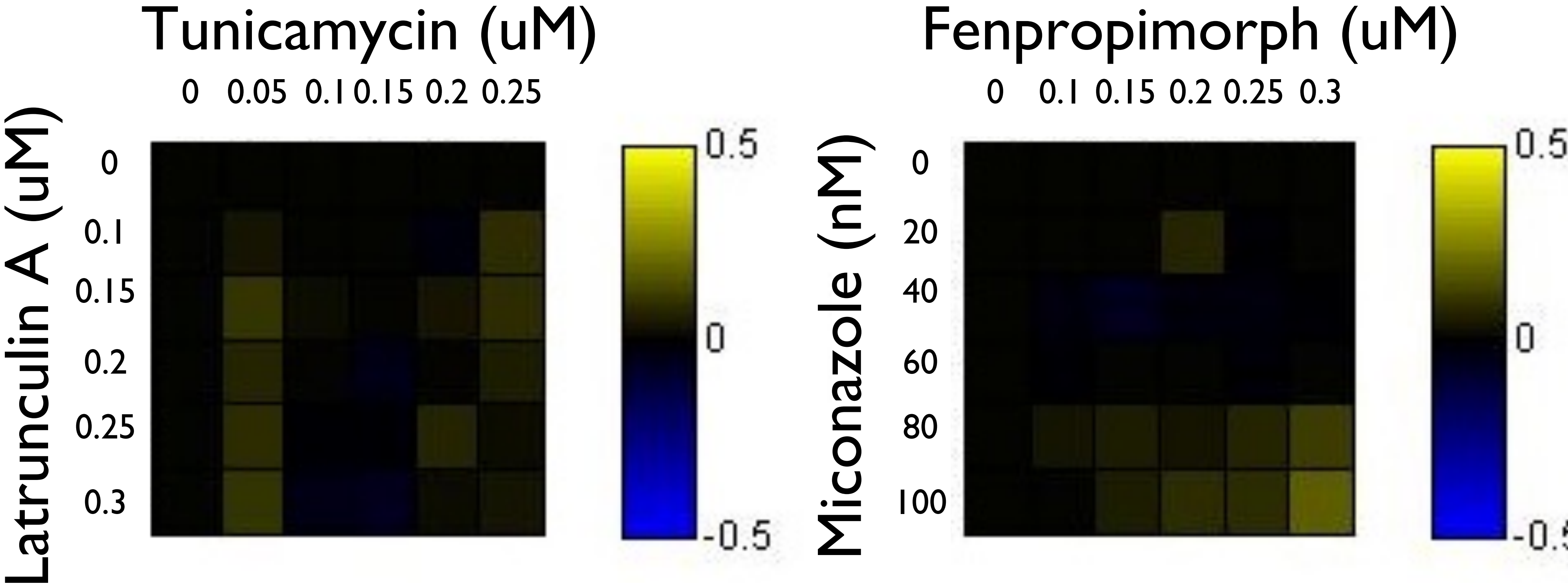

Repeat - Lower Dosage

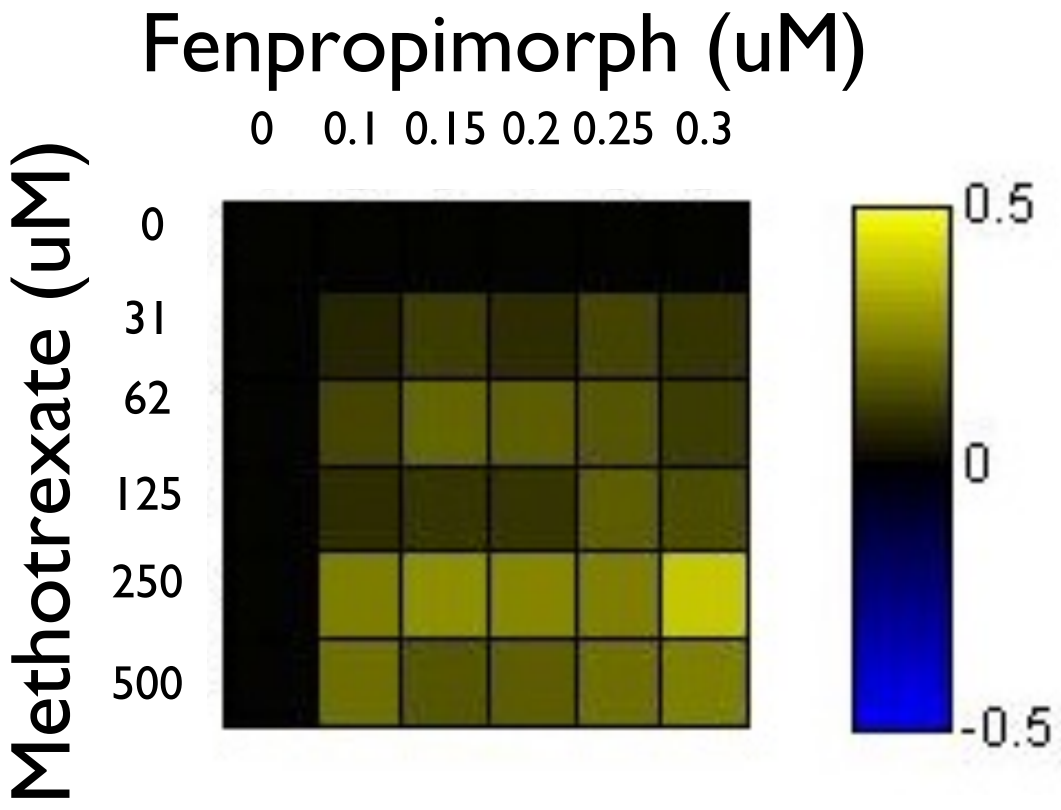

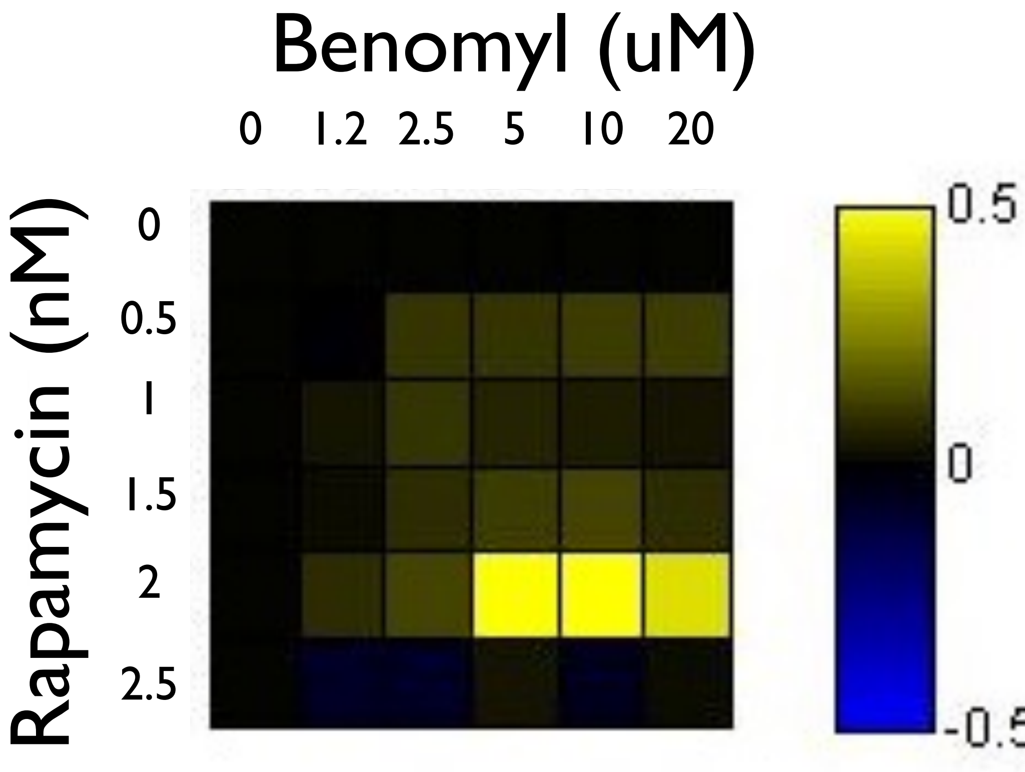

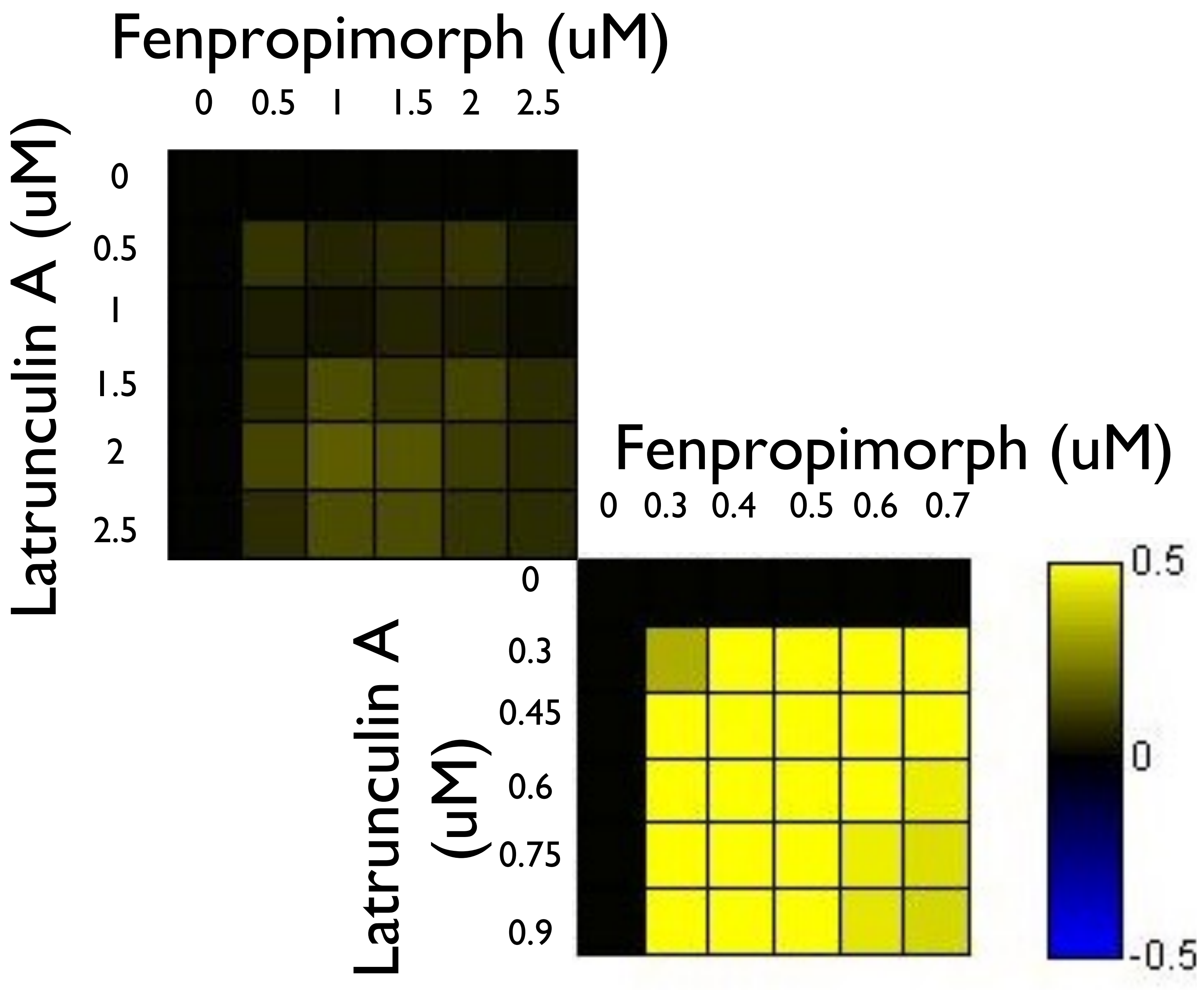
Fenpropimorph (uM)
0 0.5 1 1.5 2 2.5
Latrunculin A (uM)
0 0.5 1 1.5 2 2.5
Fenpropimorph (uM)
0 0.3 0.4 0.5 0.6 0.7
Latrunculin A (uM)
0 0.3 0.45 0.6 0.75 0.9
0.5
0
-0.5

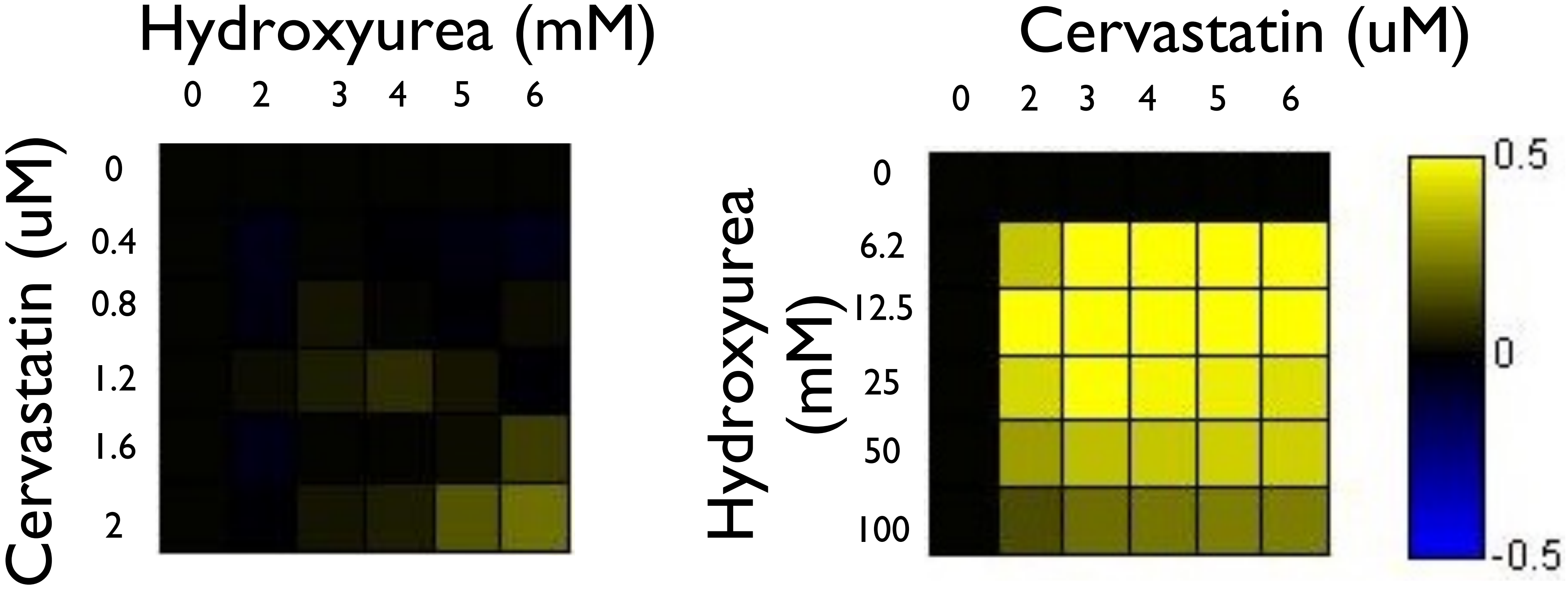

Hydroxyurea (mM)
0 2 3 4 5 6
Cervastatin (uM)
0
0.4
0.8
1.2
1.6
2
Cervastatin (uM)
0 2 3 4 5 6
Hydroxyurea (mM)
0
6.2
12.5
25
50
100
0.5
0
-0.5

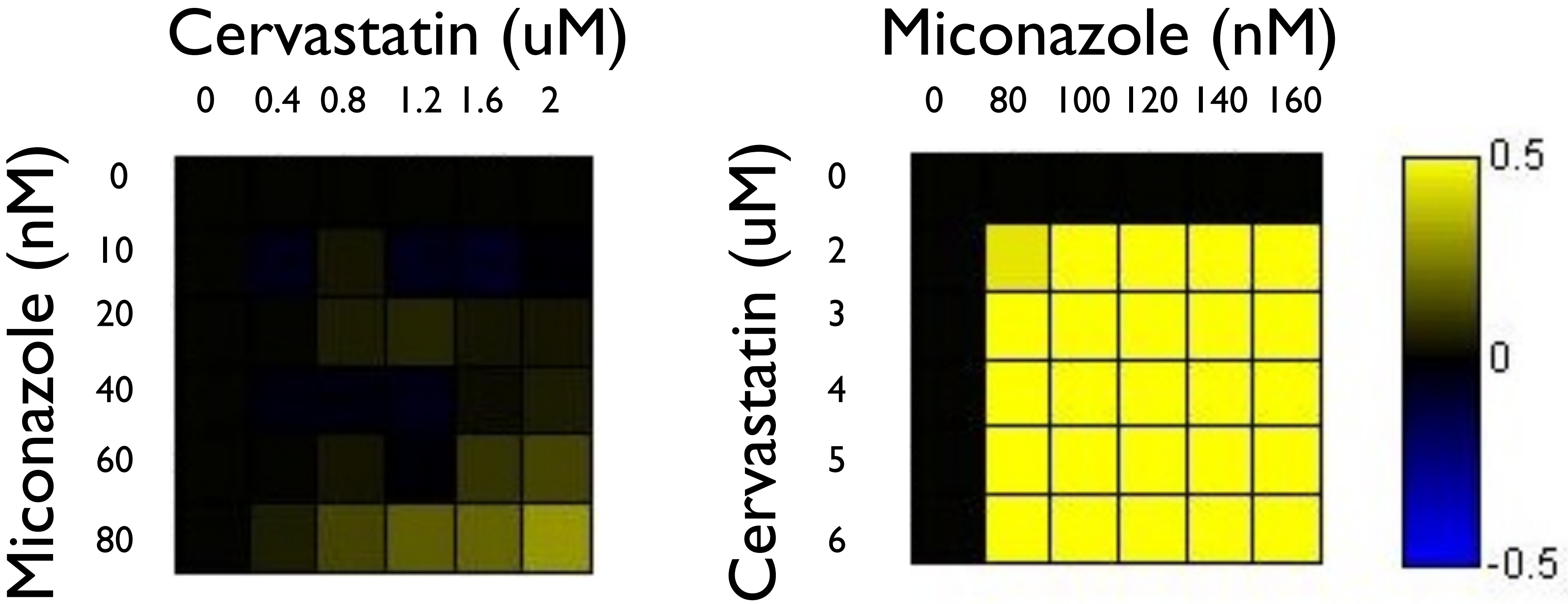

Cervastatin (uM)
0 0.4 0.8 1.2 1.6 2
Miconazole (nM)
0 10 20 40 60 80
Miconazole (nM)
0 80 100 120 140 160
Cervastatin (uM)
0 2 3 4 5 6
0.5
0
-0.5

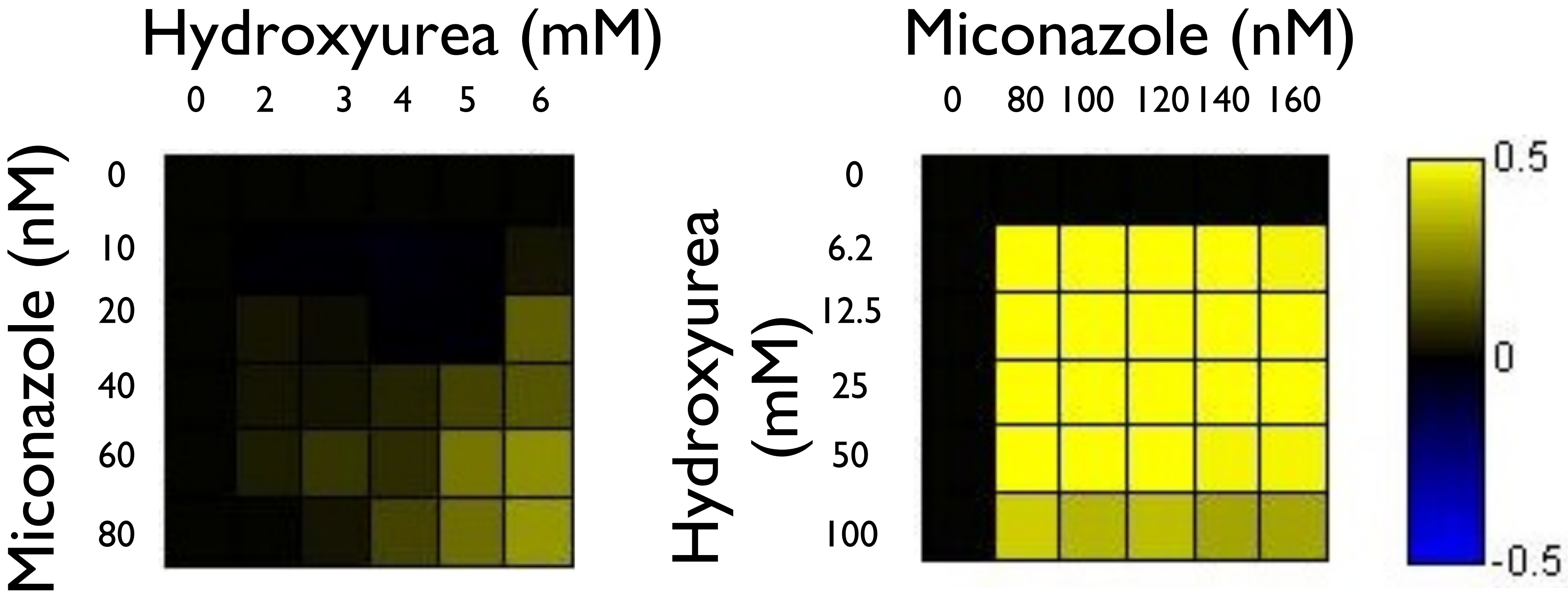

Hydroxyurea (mM)
0 2 3 4 5 6
Miconazole (nM)
0
10
20
40
60
80
Miconazole (nM)
0 80 100 120 140 160
Hydroxyurea (mM)
0
6.2
12.5
25
50
100
0.5
0
-0.5

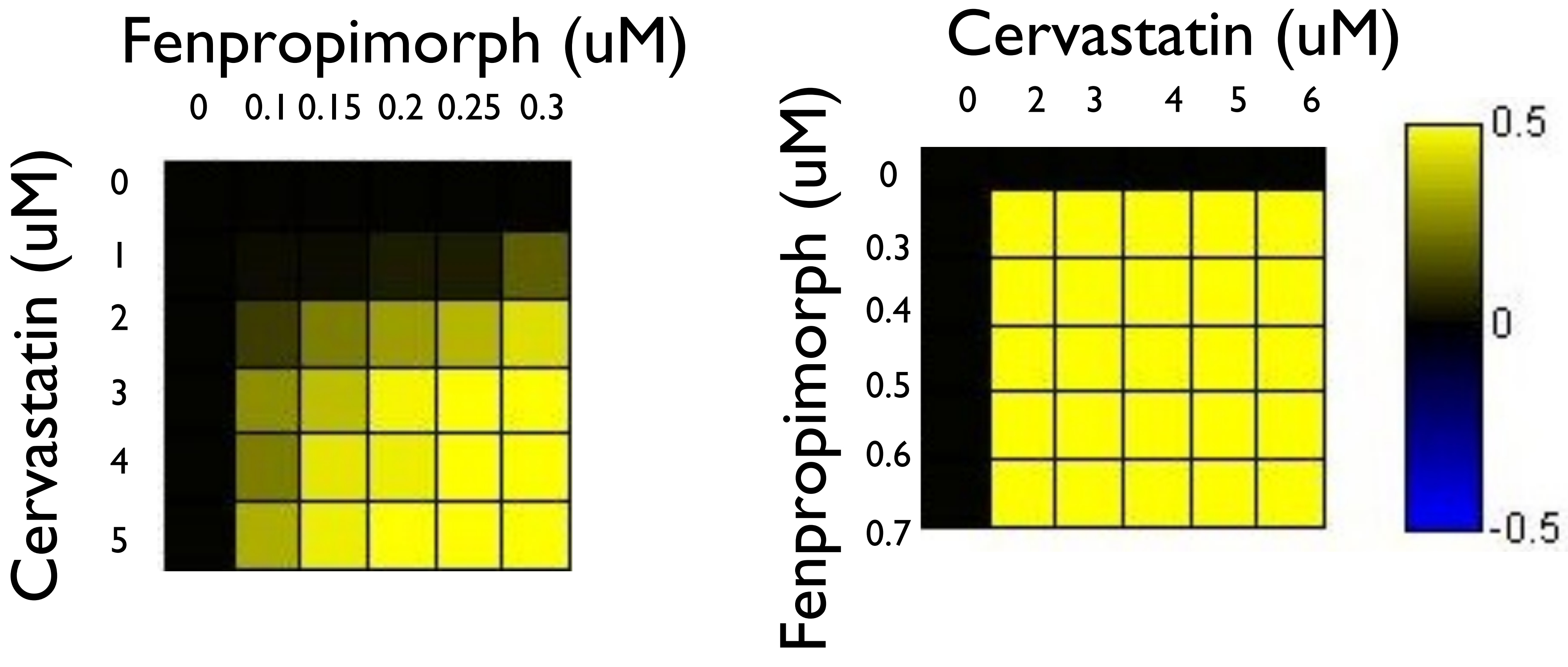

Fenpropimorph (uM)
0 0.1 0.15 0.2 0.25 0.3
Cervastatin (uM)
0
1
2
3
4
5
Cervastatin (uM)
0 2 3 4 5 6
Fenpropimorph (uM)
0
0.3
0.4
0.5
0.6
0.7
0.5
0
-0.5

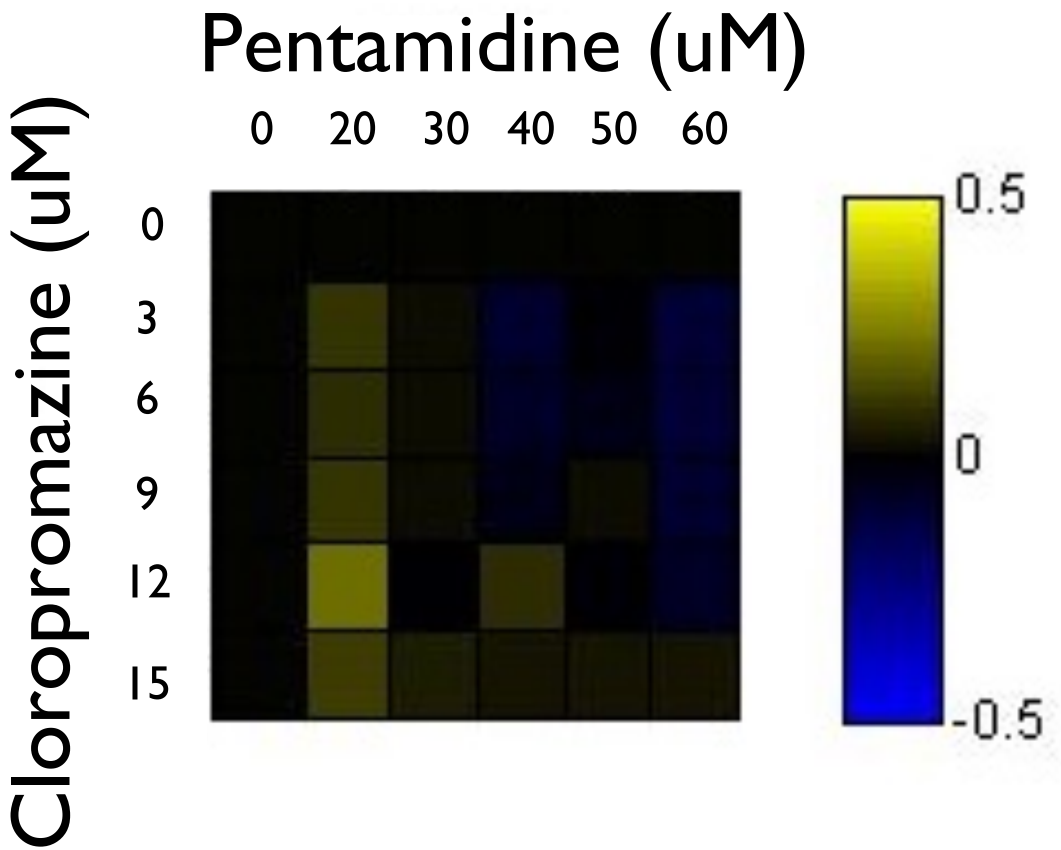

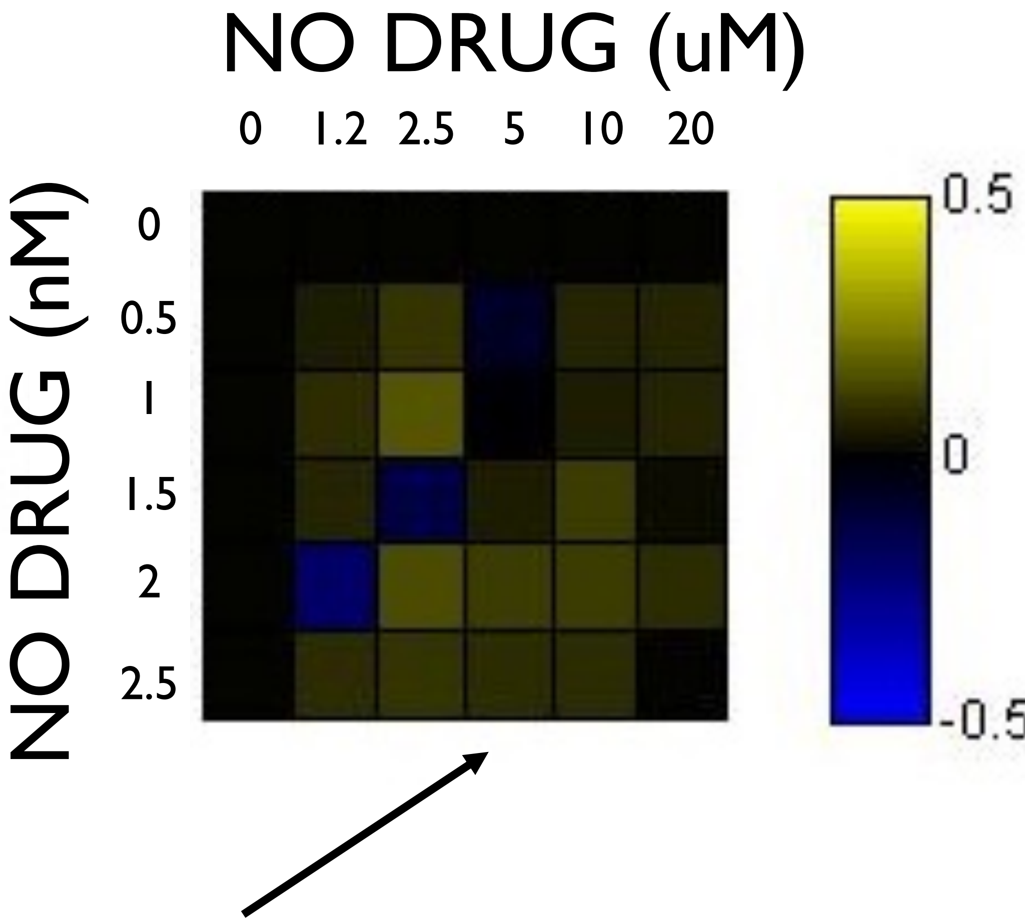

NO YEAST ON THIS SIDE OF PLATE!

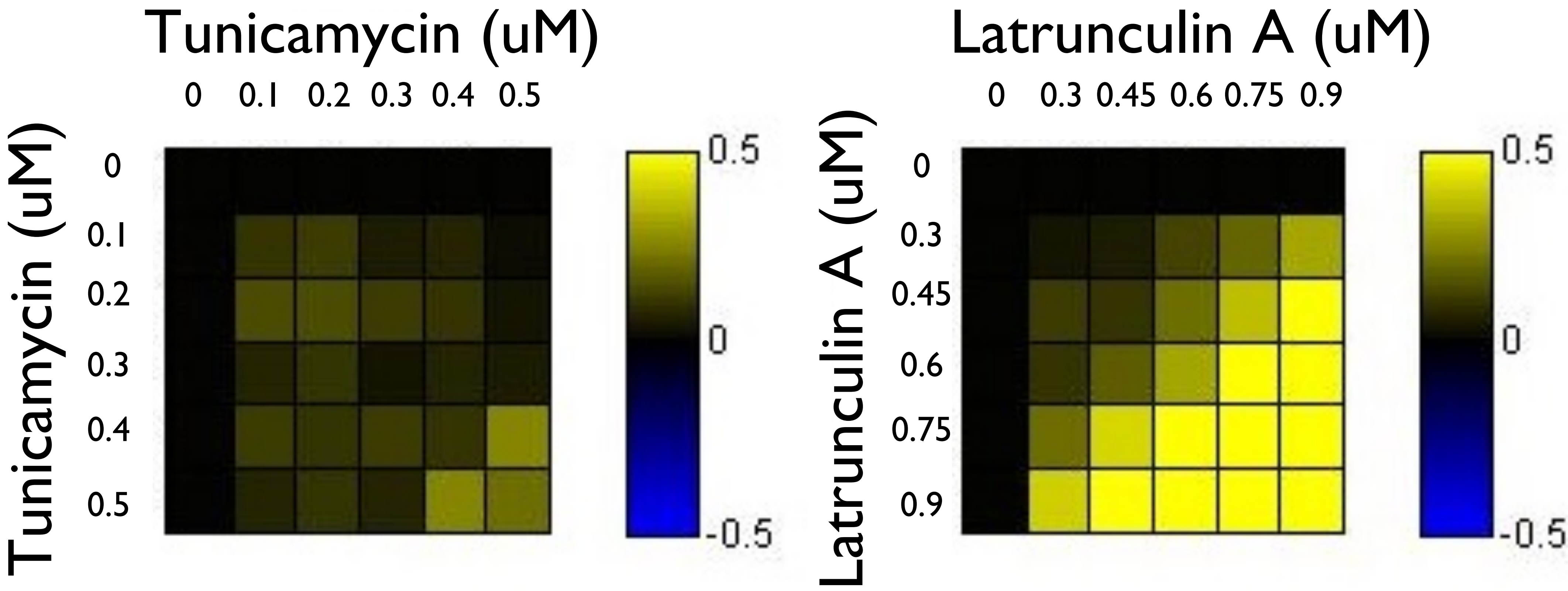
Tunicamycin (uM)
0 0.1 0.2 0.3 0.4 0.5
Tunicamycin (uM)
0 0.1 0.2 0.3 0.4 0.5
0.5
0
-0.5
Latrunculin A (uM)
0 0.3 0.45 0.6 0.75 0.9
Latrunculin A (uM)
0 0.3 0.45 0.6 0.75 0.9
0.5
0
-0.5

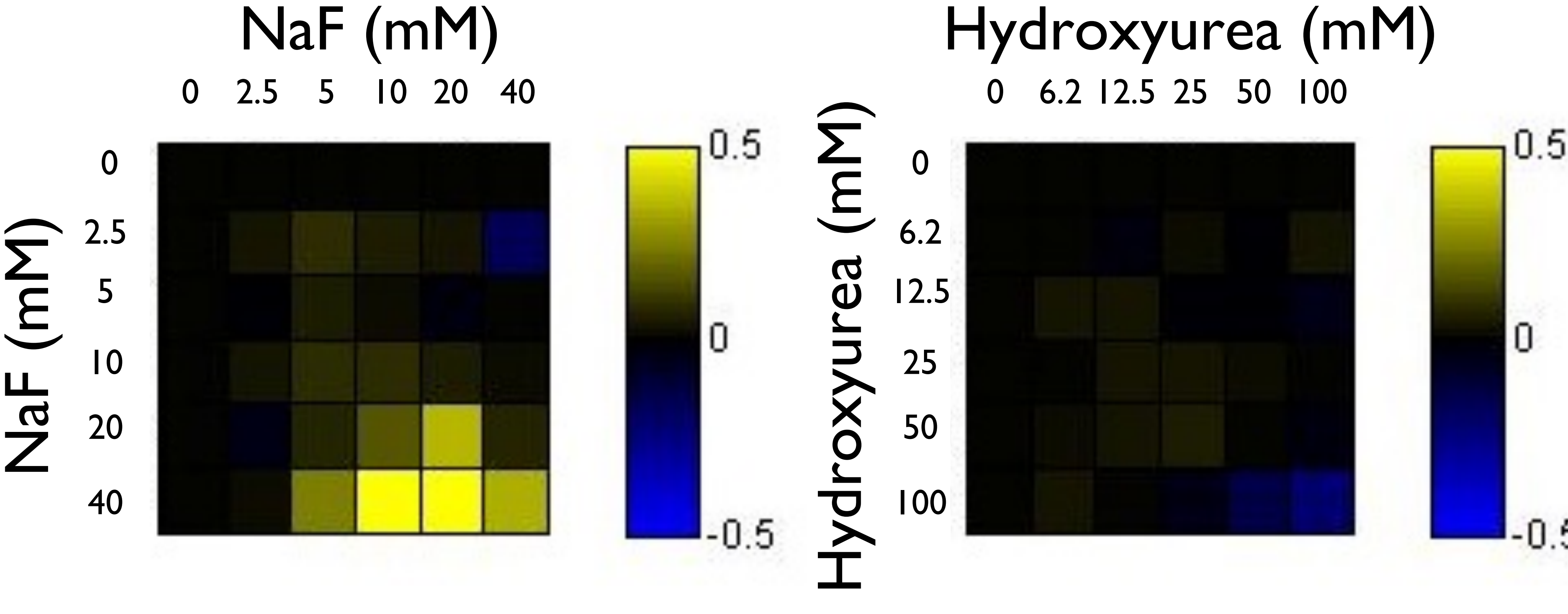
NaF (mM)
0 2.5 5 10 20 40
NaF (mM)
0 2.5 5 10 20 40
0.5
0
-0.5
Hydroxyurea (mM)
0 6.2 12.5 25 50 100
Hydroxyurea (mM)
0 6.2 12.5 25 50 100
0.5
0

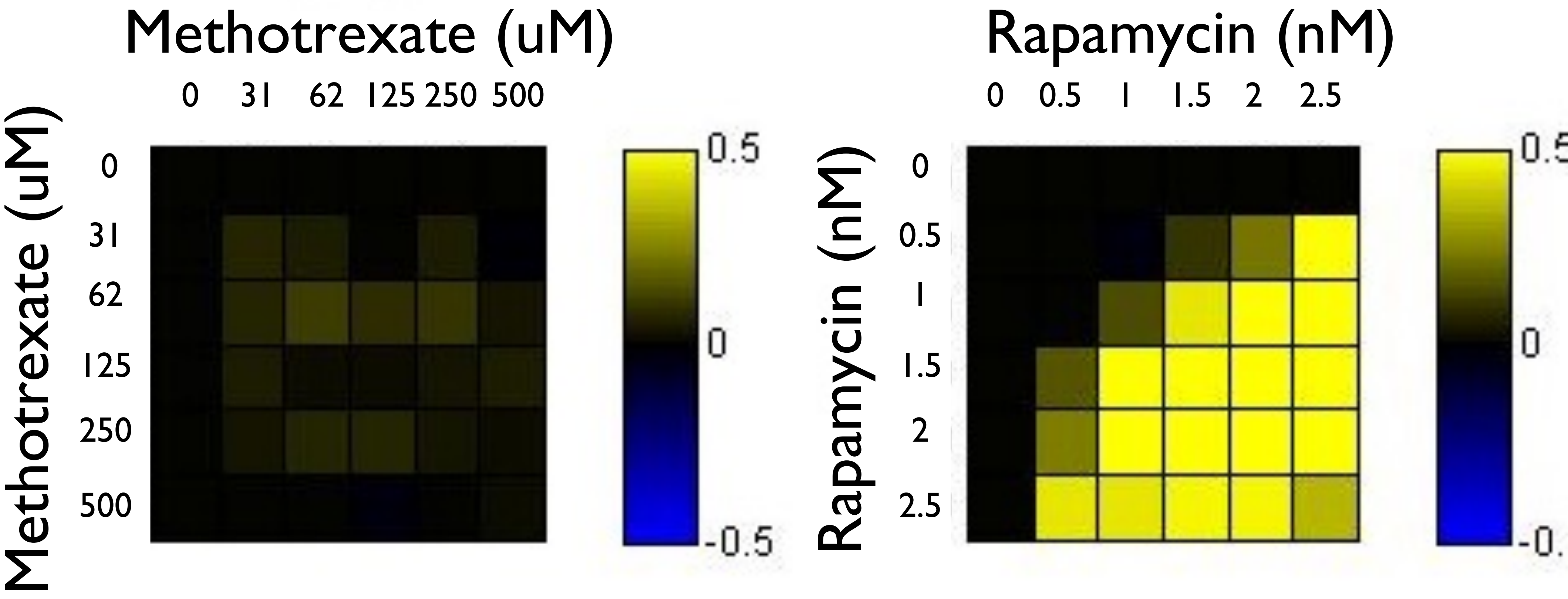

Methotrexate (uM)
0 31 62 125 250 500
Methotrexate (uM)
0 31 62 125 250 500
0.5
0
-0.5
Rapamycin (nM)
0 0.5 1 1.5 2 2.5
Rapamycin (nM)
0 0.5 1 1.5 2 2.5
0.5
0

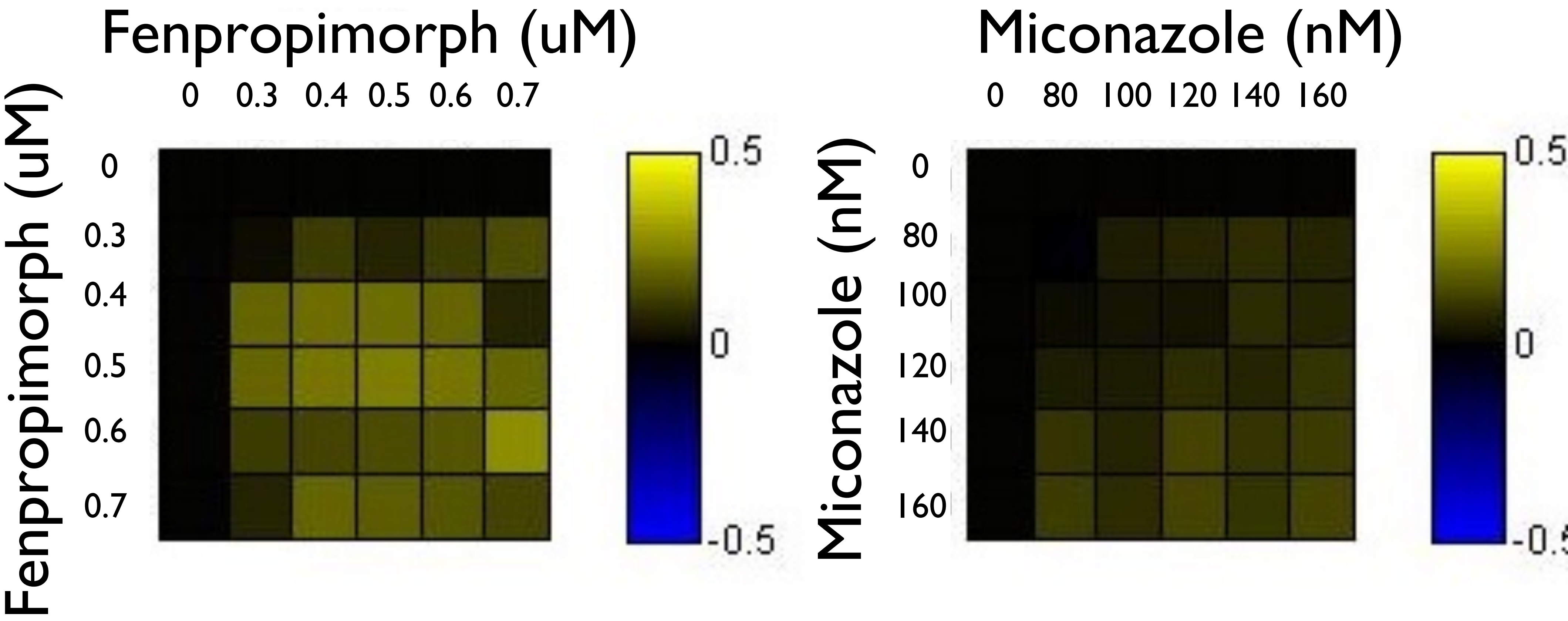

Fenpropimorph (uM)
0 0.3 0.4 0.5 0.6 0.7
Fenpropimorph (uM)
0 0.3 0.4 0.5 0.6 0.7
0.5
0
-0.5
Miconazole (nM)
0 80 100 120 140 160
Miconazole (nM)
0 80 100 120 140 160
0.5
0

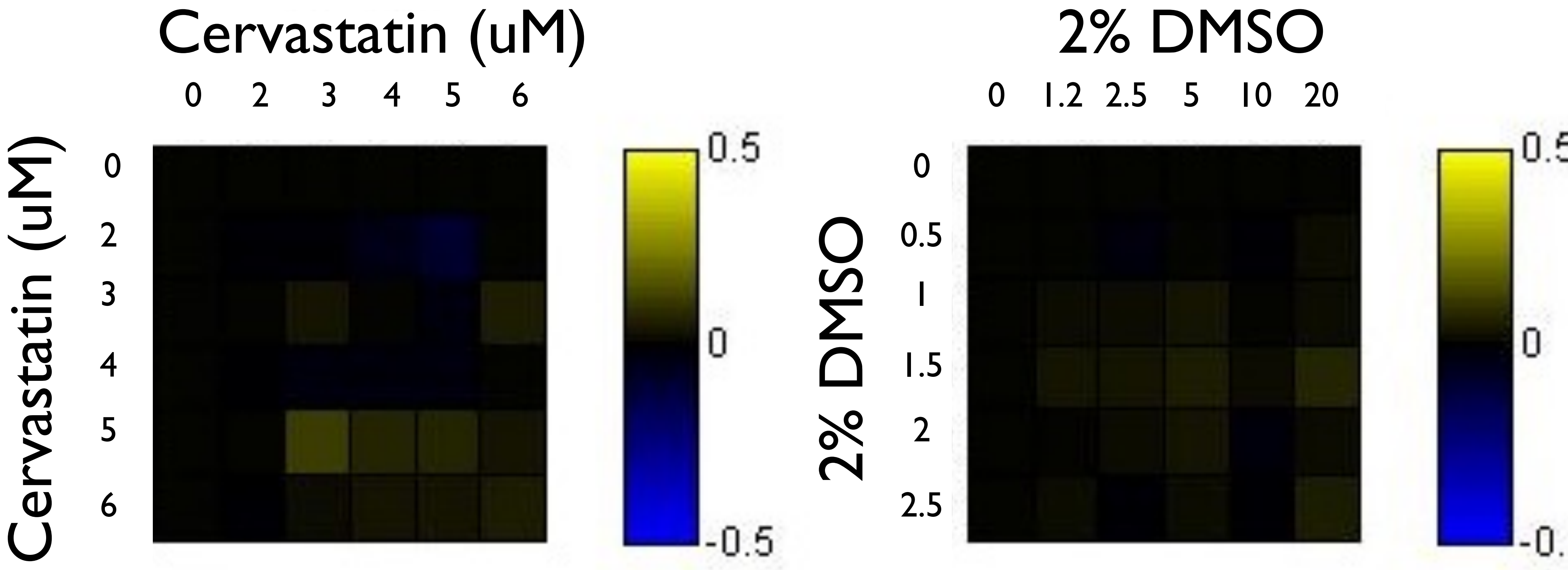

Cervastatin (uM)
0 2 3 4 5 6
Cervastatin (uM)
0 2 3 4 5 6
0.5
0
-0.5
2% DMSO
0 1.2 2.5 5 10 20
2% DMSO
0 0.5 1 1.5 2 2.5
0.5
0

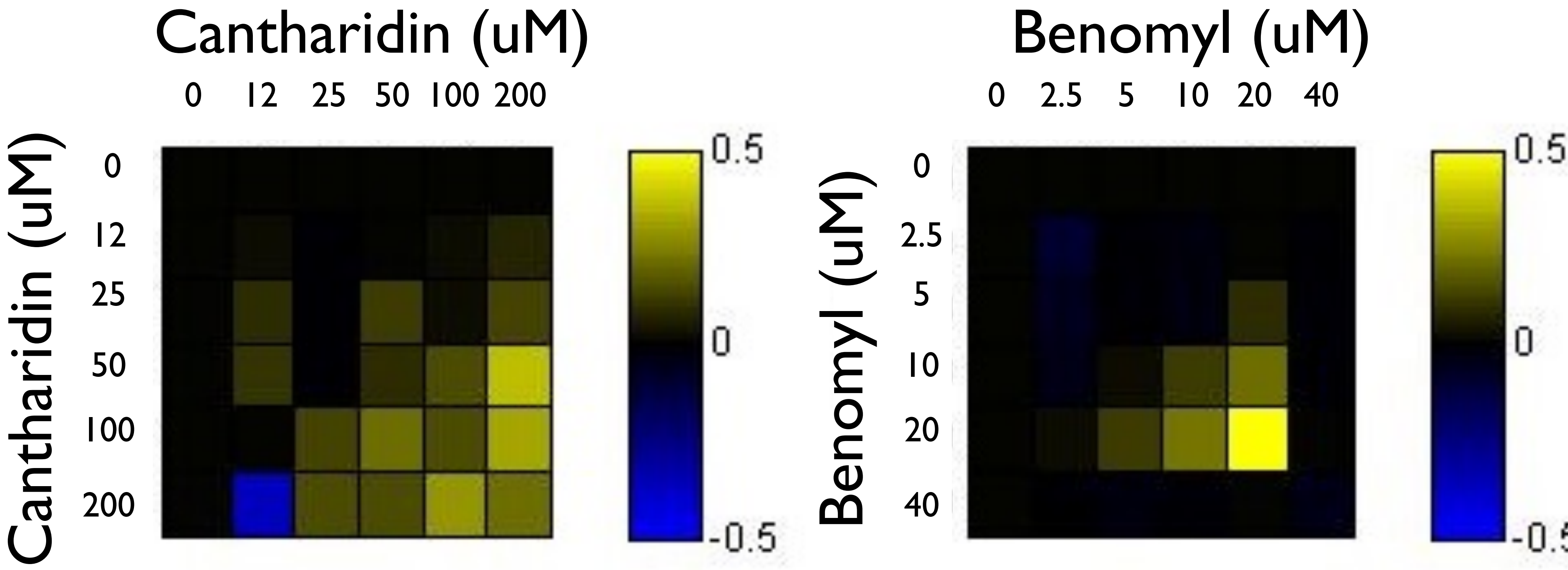
Cantharidin (uM)
0 12 25 50 100 200
Cantharidin (uM)
0 12 25 50 100 200
0.5
0
-0.5
Benomyl (uM)
0 2.5 5 10 20 40
Benomyl (uM)
0 2.5 5 10 20 40
0.5
0

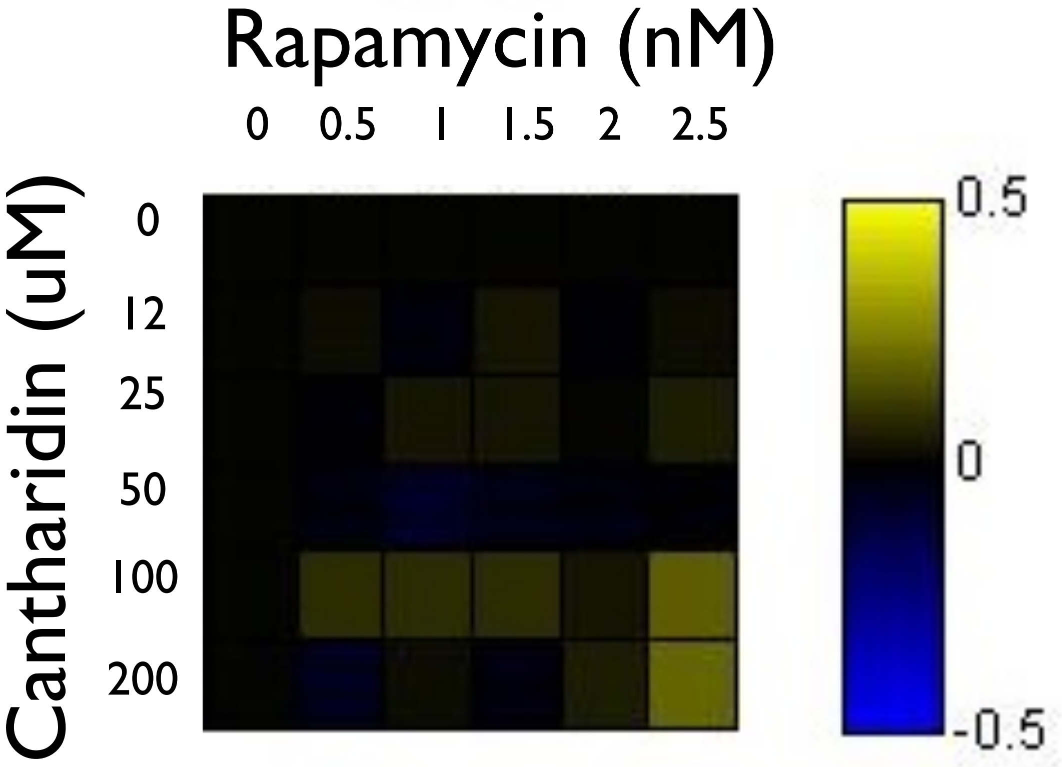
Rapamycin (nM)
0 0.5 1 1.5 2 2.5
Cantharidin (uM)
0 12 25 50 100 200
0.5
0
-0.5

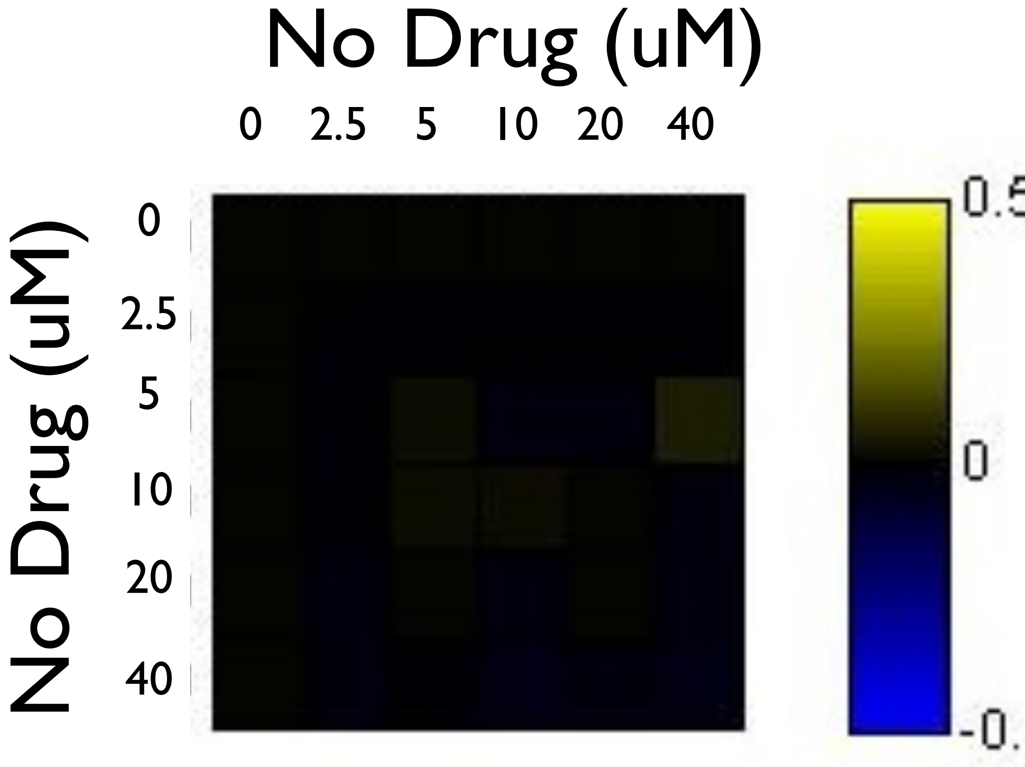
No Drug (uM)
0 2.5 5 10 20 40
No Drug (uM)
0 2.5 5 10 20 40
0.5
0

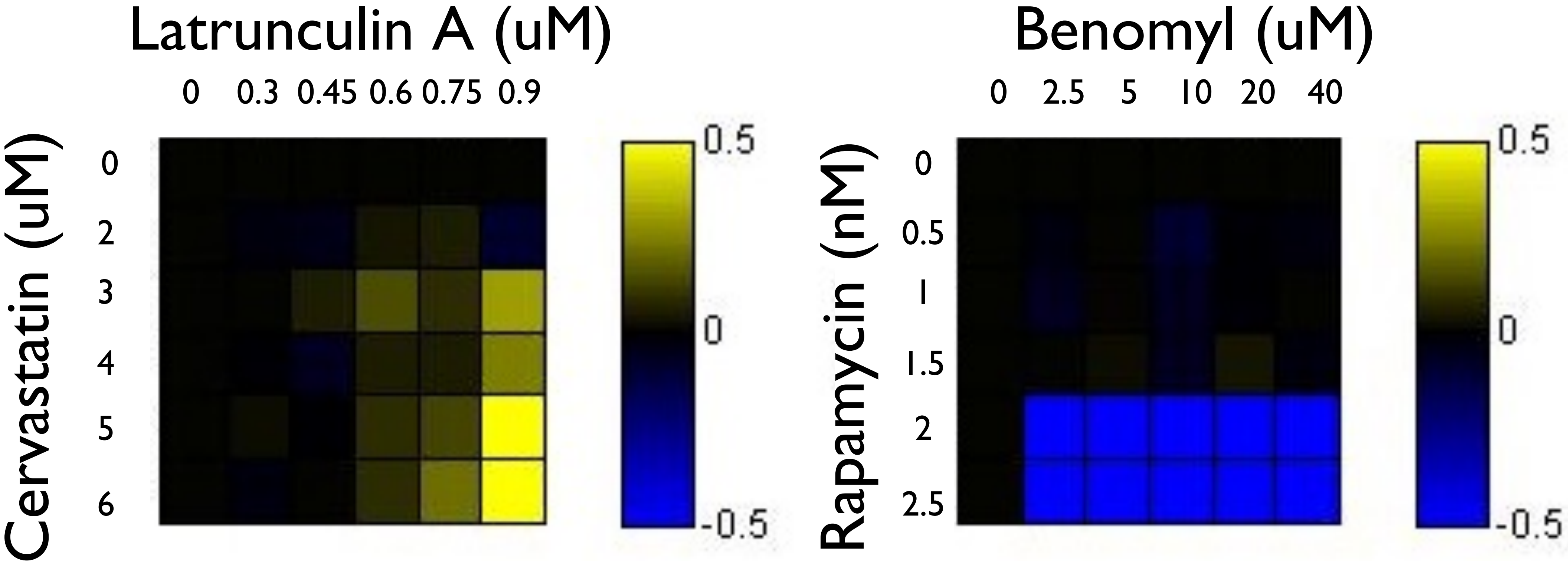

Latrunculin A (uM)
0 0.3 0.45 0.6 0.75 0.9
Cervastatin (uM)
0 2 3 4 5 6
0.5
0
-0.5
Benomyl (uM)
0 2.5 5 10 20 40
Rapamycin (nM)
0 0.5 1 1.5 2 2.5
0.5
0
-0.5

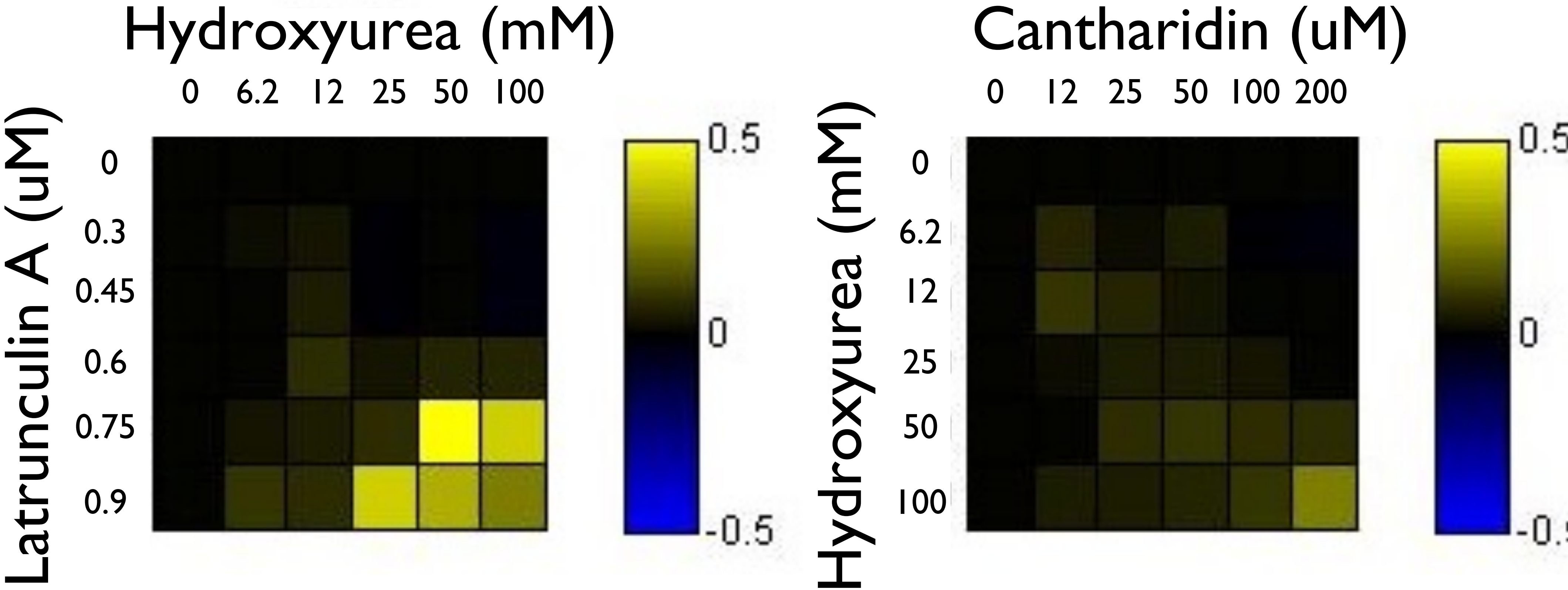
Hydroxyurea (mM)
0 6.2 12 25 50 100
Latrunculin A (uM)
0 0.3 0.45 0.6 0.75 0.9
0.5
0
-0.5
Cantharidin (uM)
0 12 25 50 100 200
Hydroxyurea (mM)
0 6.2 12 25 50 100
0.5
0

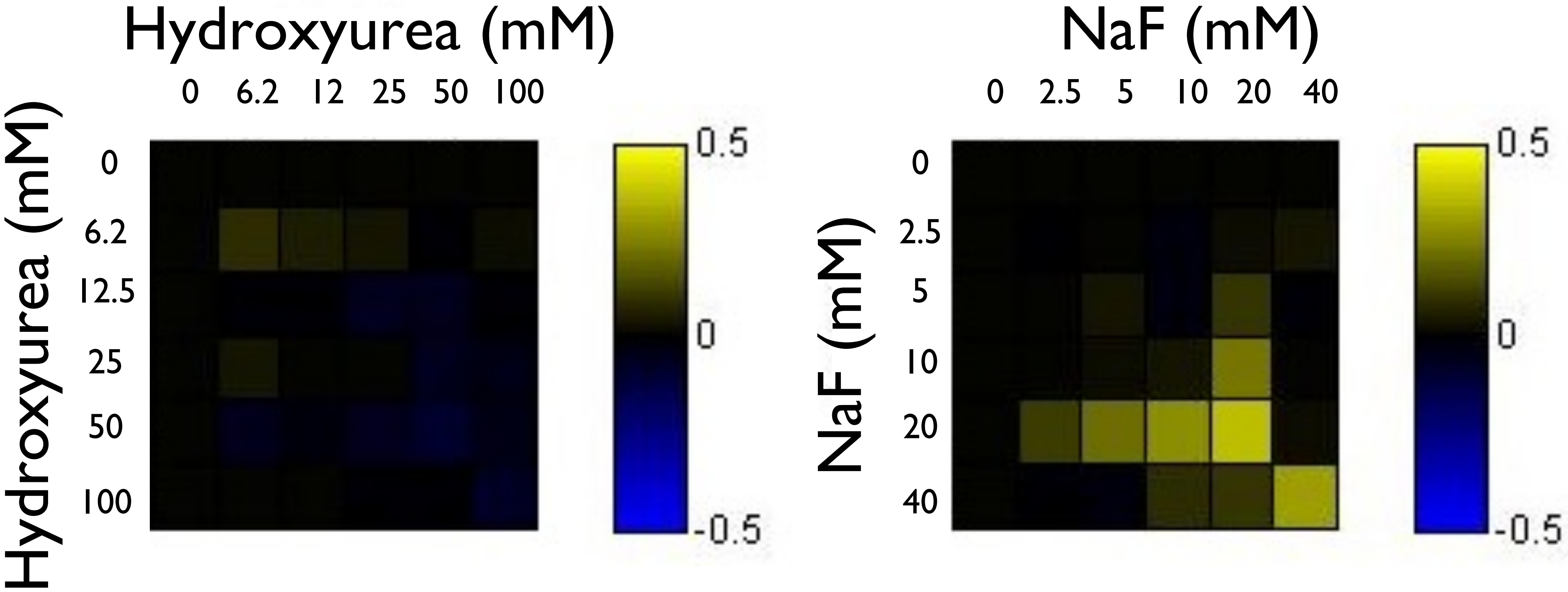
Hydroxyurea (mM)
0
6.2
12
25
50
100
Hydroxyurea (mM)
0
6.2
12.5
25
50
100
0.5
0
-0.5
NaF (mM)
0
2.5
5
10
20
40
NaF (mM)
0
2.5
5
10
20
40
0.5
0
-0.5

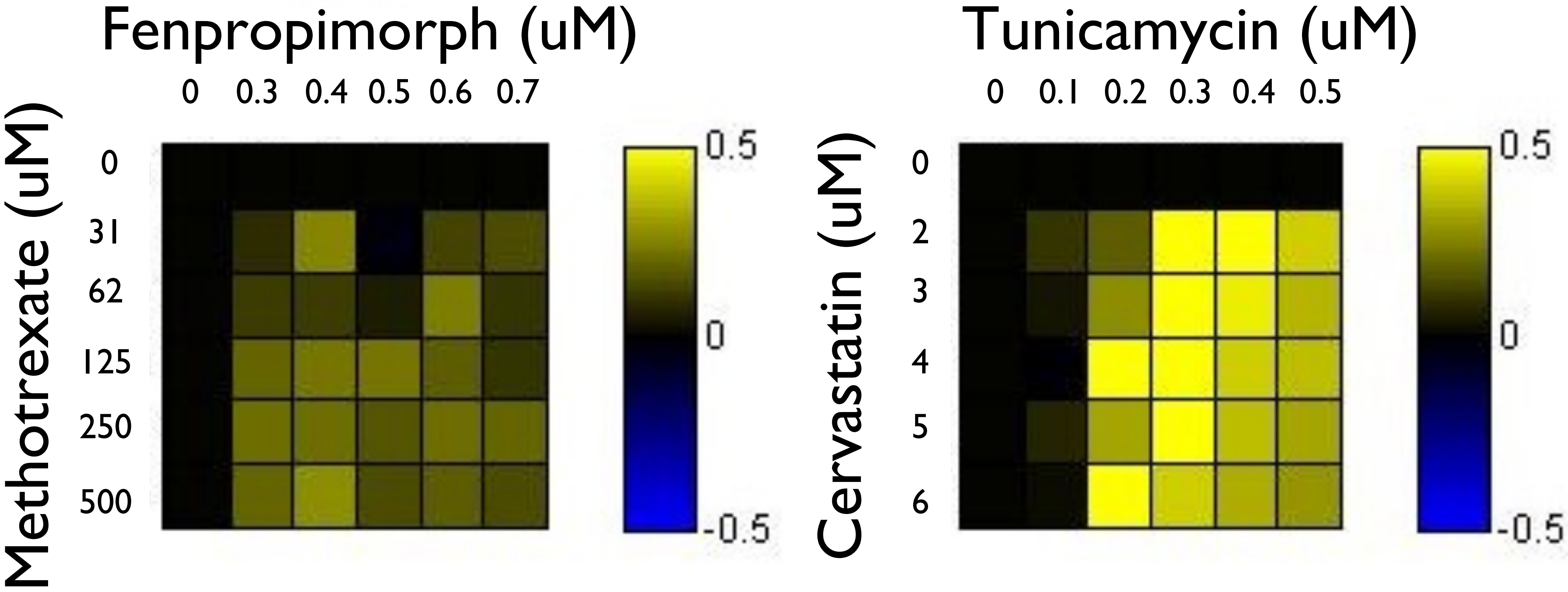
Fenpropimorph (uM)
0 0.3 0.4 0.5 0.6 0.7
Methotrexate (uM)
0
31
62
125
250
500
0.5
0
-0.5
Tunicamycin (uM)
0 0.1 0.2 0.3 0.4 0.5
Cervastatin (uM)
0
2
3
4
5
6
0.5
0
-0.5

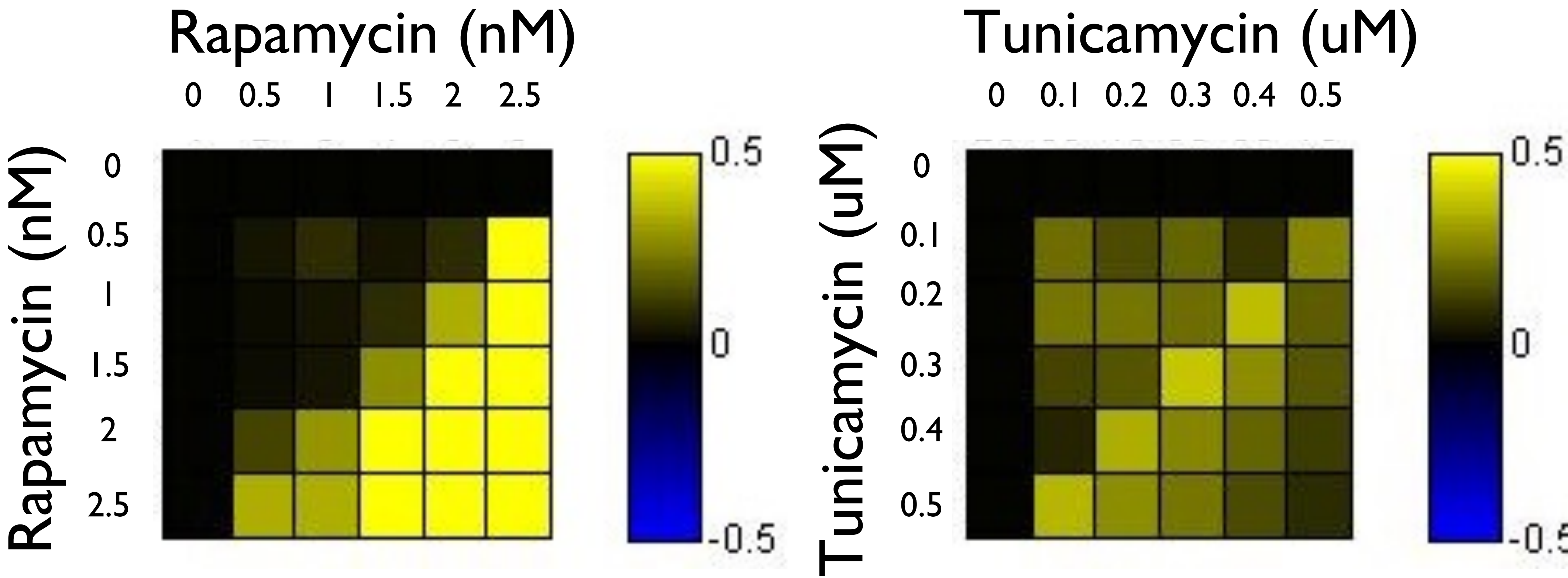

Rapamycin (nM)
0 0.5 1 1.5 2 2.5
Rapamycin (nM)
0 0.5 1 1.5 2 2.5
0.5
0
-0.5
Tunicamycin (uM)
0 0.1 0.2 0.3 0.4 0.5
Tunicamycin (uM)
0 0.1 0.2 0.3 0.4 0.5
0.5
0
-0.5

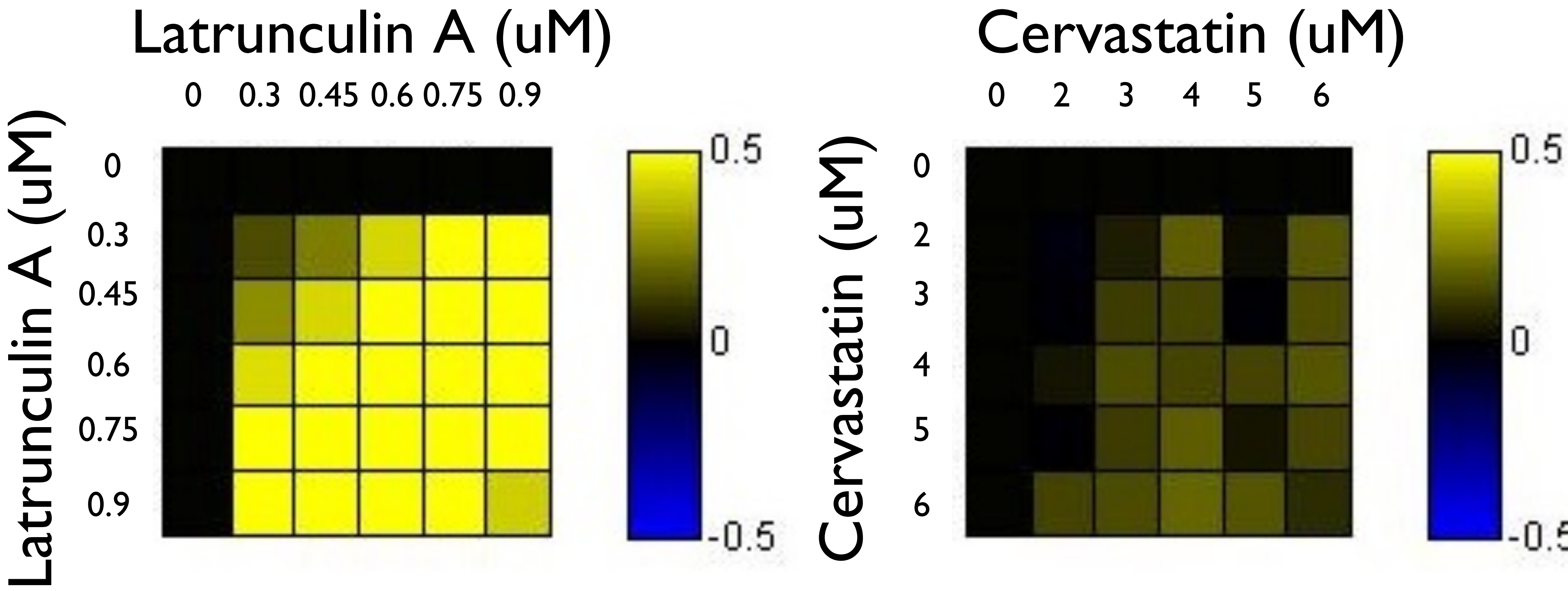
Latrunculin A (uM)
0 0.3 0.45 0.6 0.75 0.9
Latrunculin A (uM)
0 0.3 0.45 0.6 0.75 0.9
0.5
0
-0.5
Cervastatin (uM)
0 2 3 4 5 6
Cervastatin (uM)
0 2 3 4 5 6
0.5
0

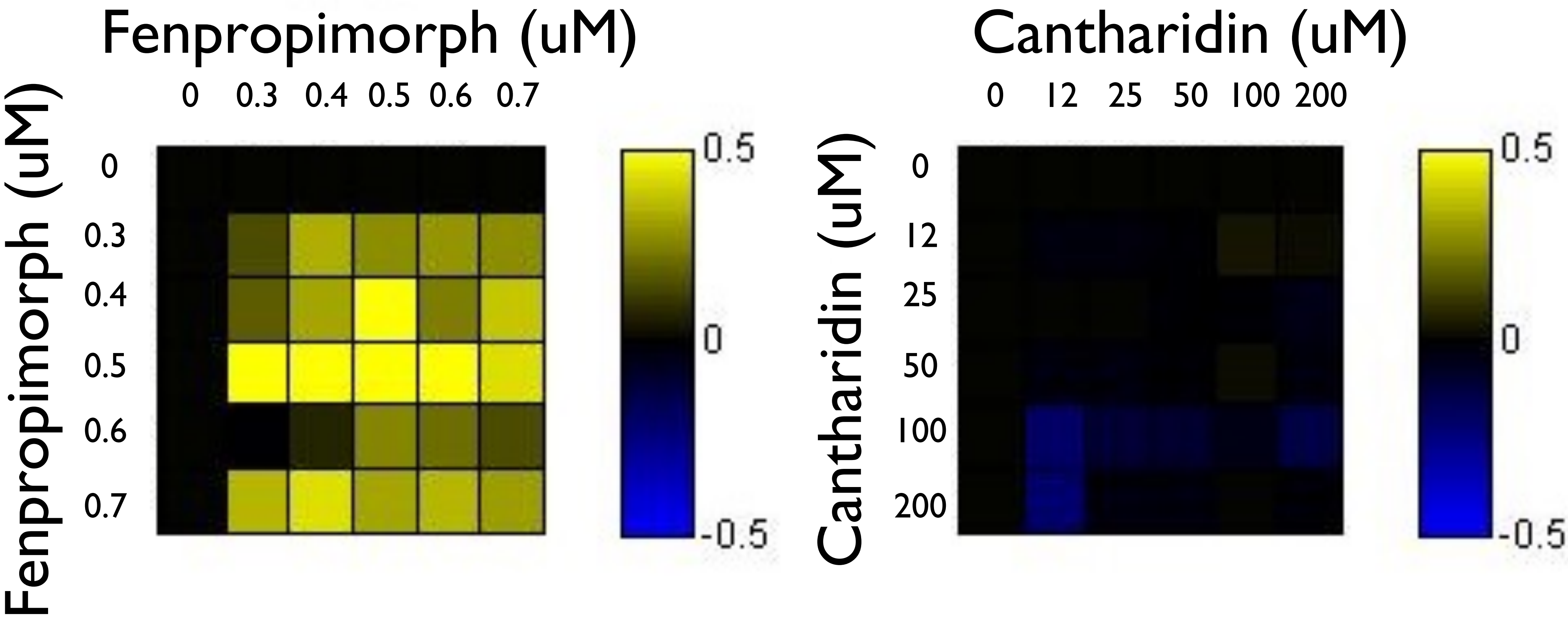
Fenpropimorph (uM)
0 0.3 0.4 0.5 0.6 0.7
Fenpropimorph (uM)
0 0.3 0.4 0.5 0.6 0.7
0.5
0
-0.5
Cantharidin (uM)
0 12 25 50 100 200
Cantharidin (uM)
0 12 25 50 100 200
0.5
0
-0.5

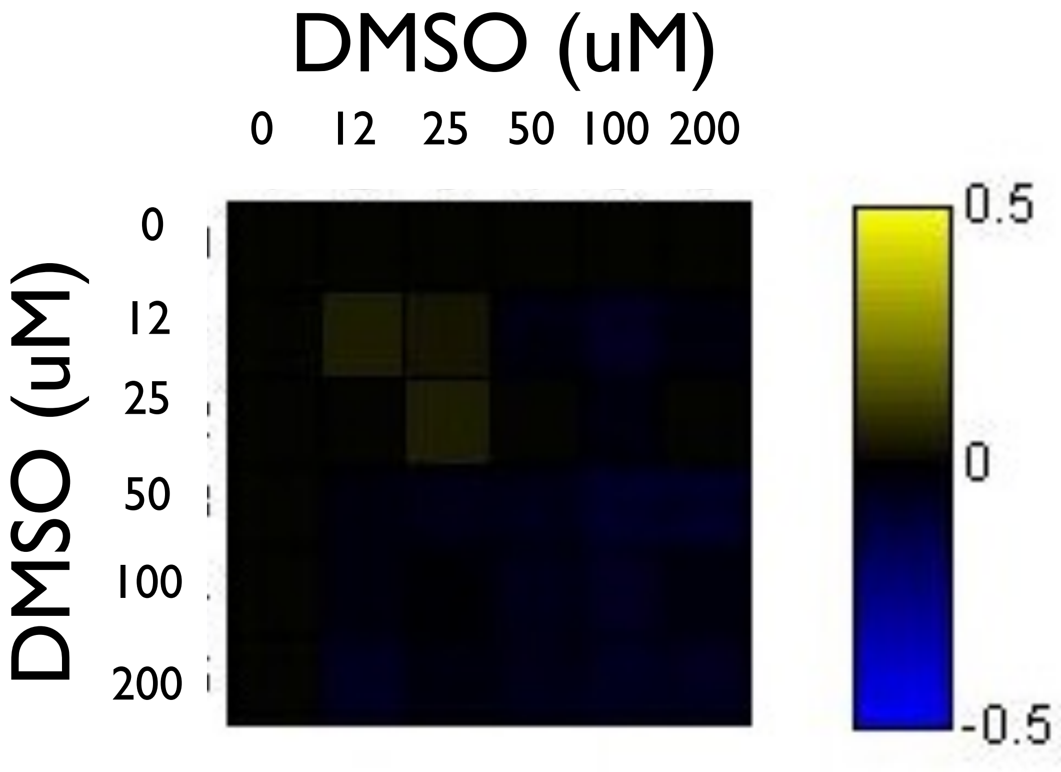
DMSO (uM)
0 12 25 50 100 200
0 12 25 50 100 200
DMSO (uM)
0.5
0
-0.5

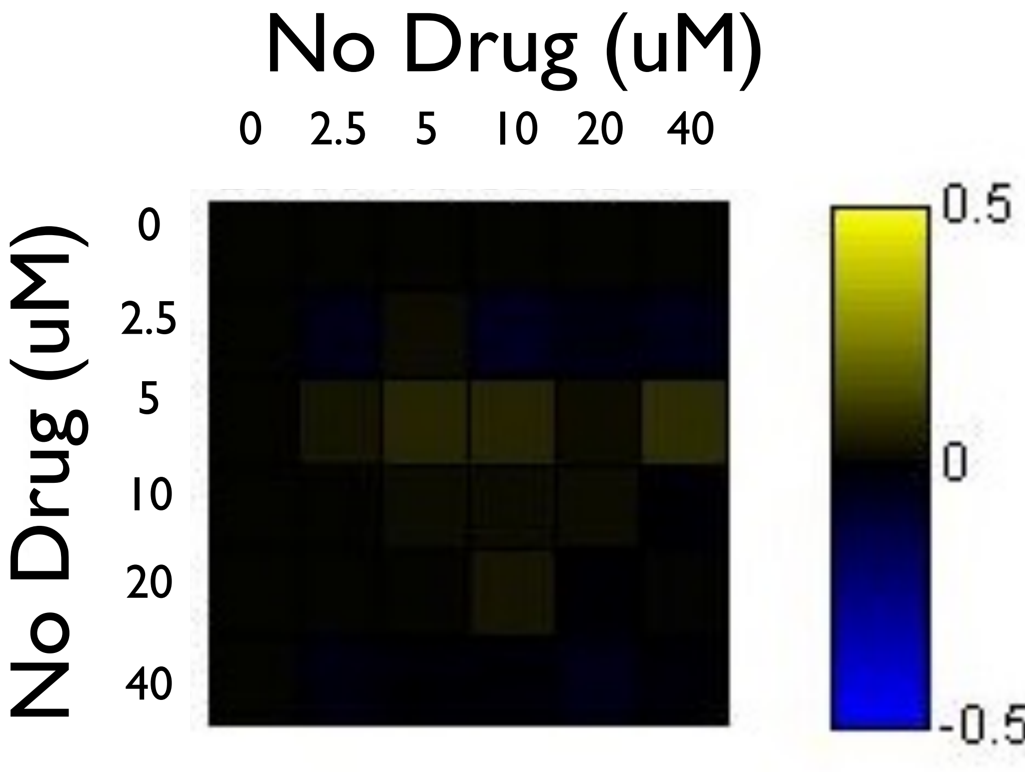
No Drug (uM)
0 2.5 5 10 20 40
0 2.5 5 10 20 40
No Drug (uM)
0.5
0
-0.5

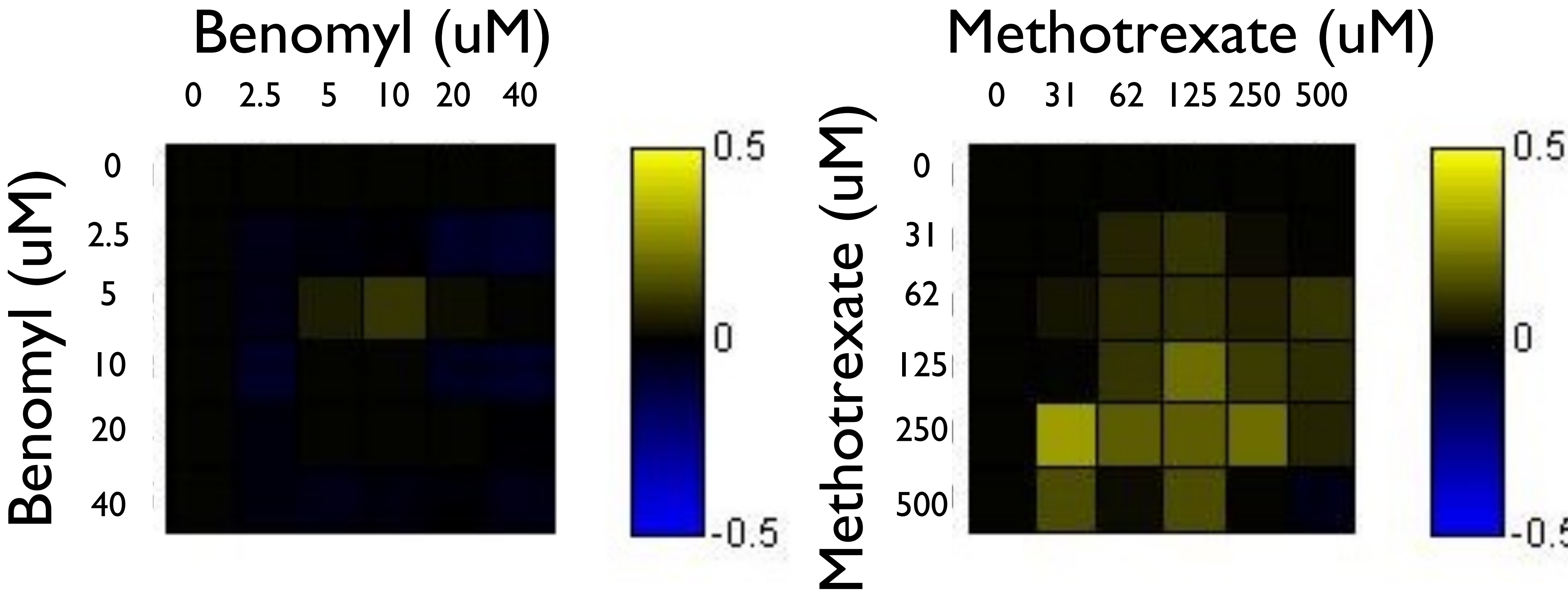
Benomyl (uM)
0 2.5 5 10 20 40
Benomyl (uM)
0
2.5
5
10
20
40
0.5
0
-0.5
Methotrexate (uM)
0 31 62 125 250 500
Methotrexate (uM)
0
31
62
125
250
500
0.5
0

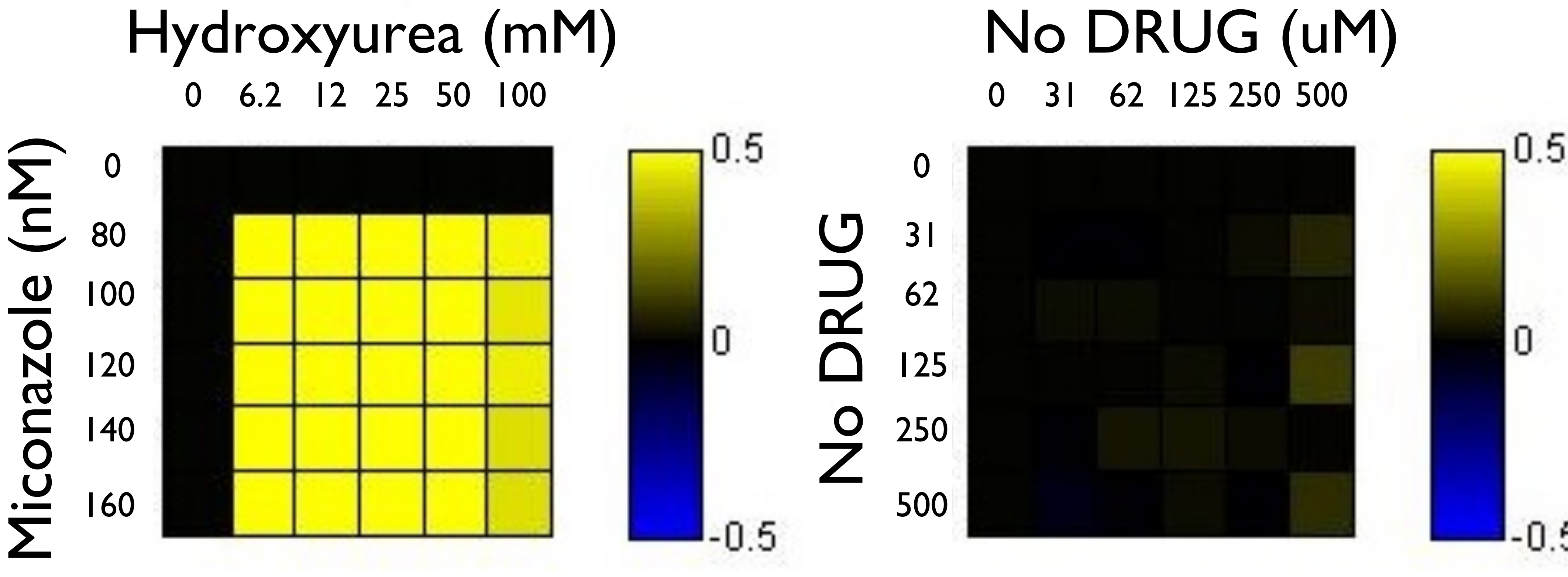

Hydroxyurea (mM)
0 6.2 12 25 50 100
Miconazole (nM)
0
80
100
120
140
160
0.5
0
-0.5
No DRUG (uM)
0 31 62 125 250 500
No DRUG
0
31
62
125
250
500
0.5
0

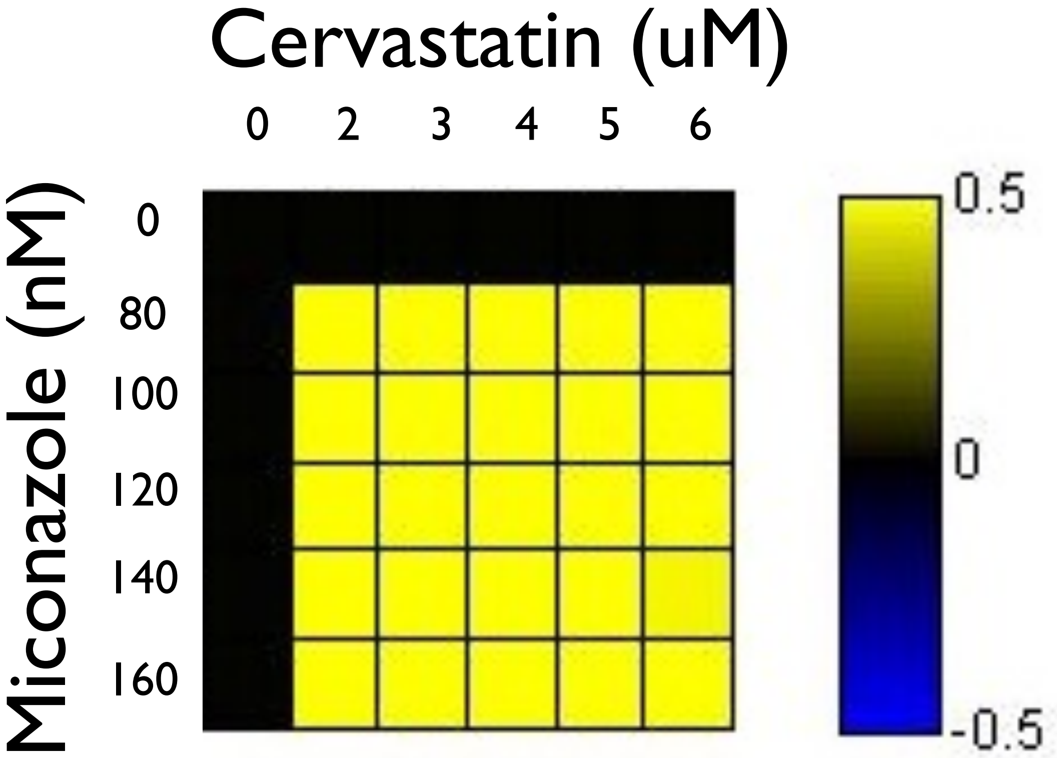

Cervastatin (uM)
0 2 3 4 5 6
Miconazole (nM)
0
80
100
120
140
160
0.5
0
-0.5

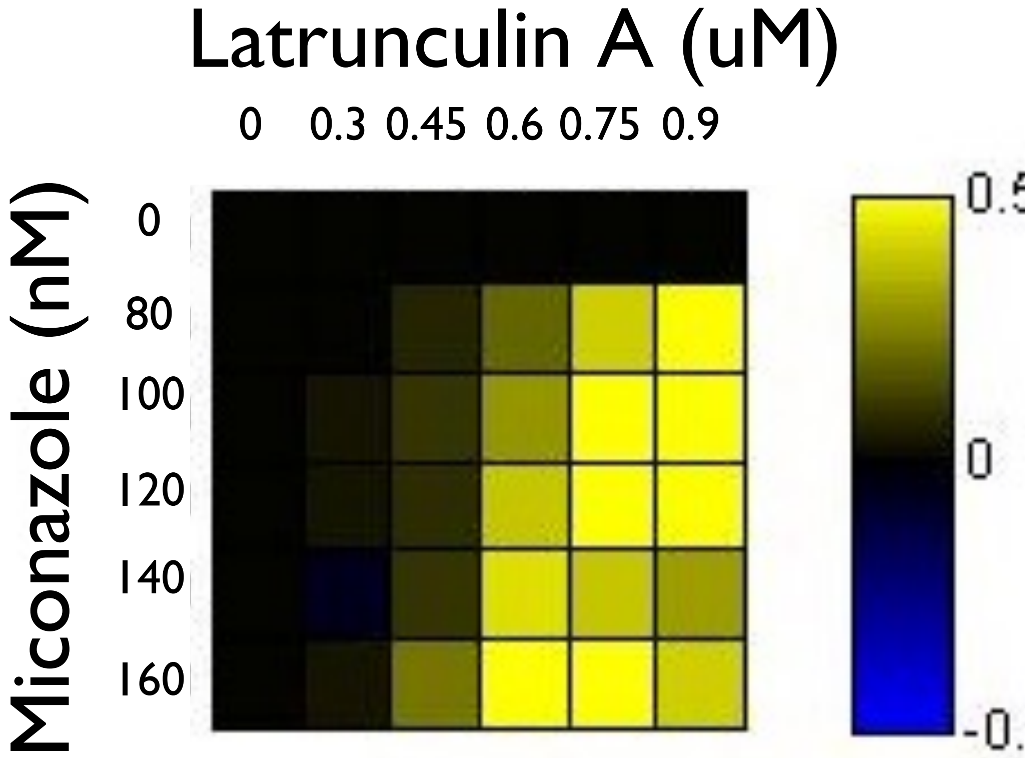

Latrunculin A (uM)
0 0.3 0.45 0.6 0.75 0.9
Miconazole (nM)
0
80
100
120
140
160
0
