# Supplementary figures and images for: Systematic Prediction of Antifungal Drug Synergy by Chemogenomic Screening in Saccharomyces cerevisiae
Source: Front Fungal Biol. 2021 Jul 2;2:683414. doi: 10.3389/ffunb.2021.683414 (PMC10512392; doi:10.3389/ffunb.2021.683414)

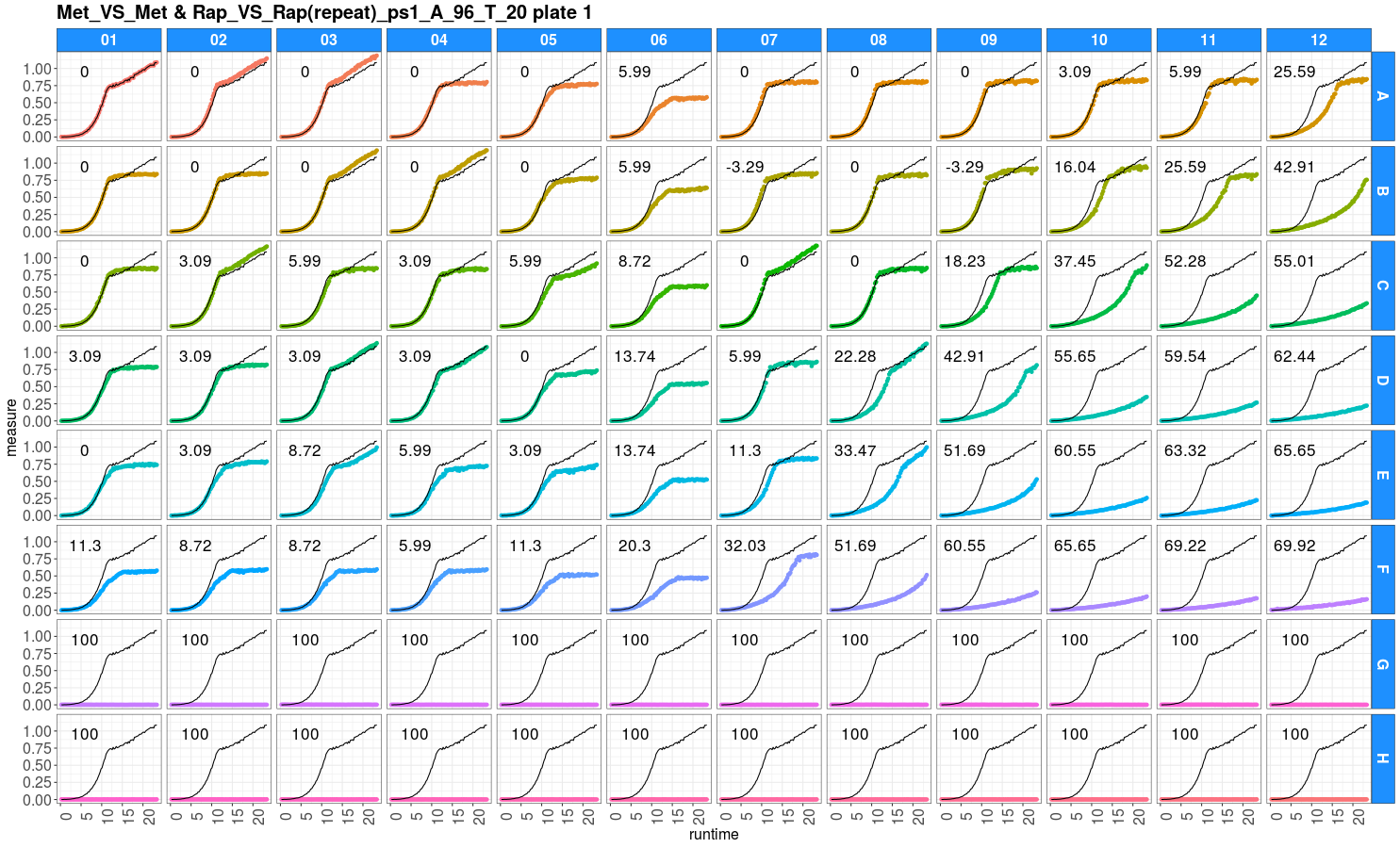

Supplement: Supplementary file 1 [file Data_Sheet_1.ZIP › Supplementary data/Sypplementary_data_1_(Drug_synergy_assays)/met_VS_met.png]

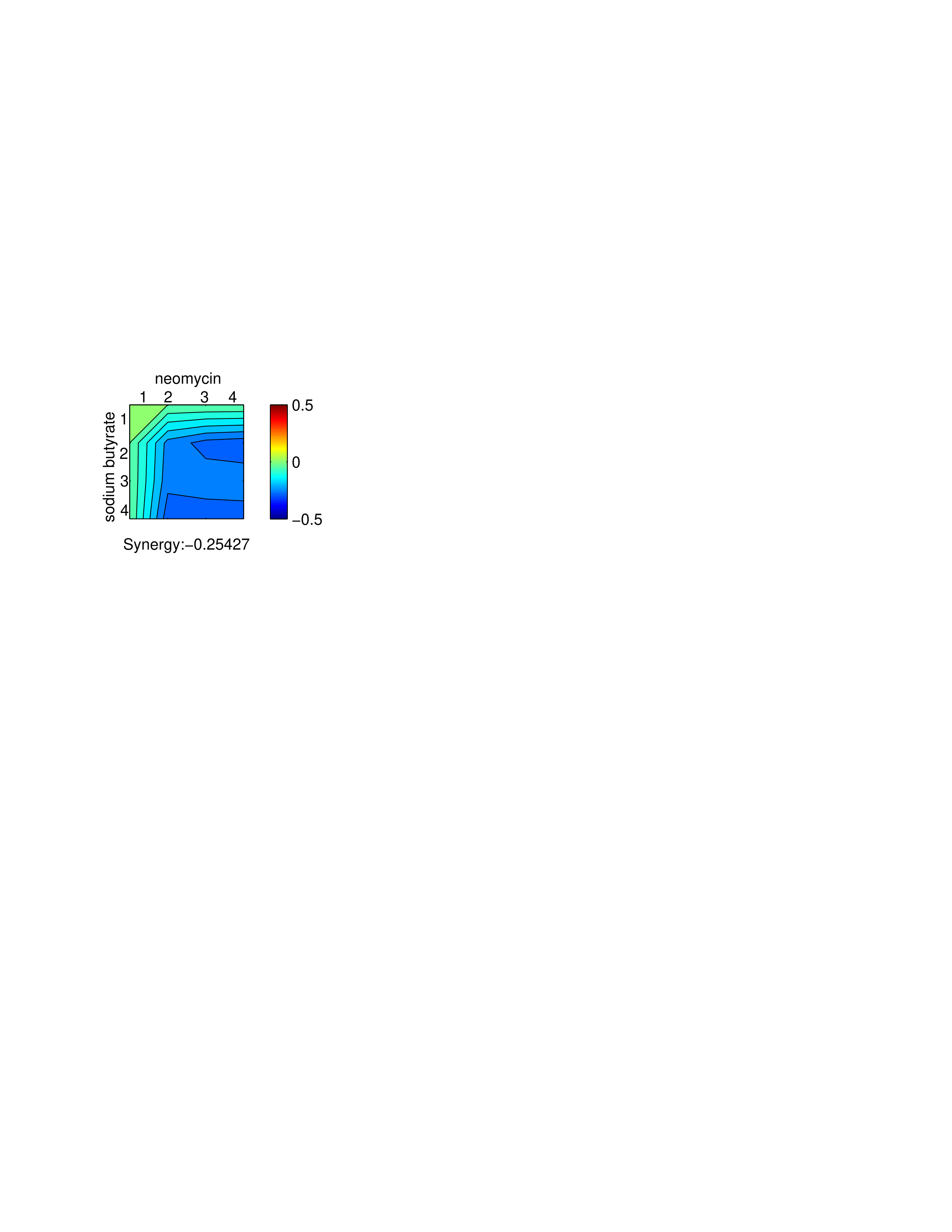

Supplement: Supplementary file 1 [file Data_Sheet_1.ZIP › Supplementary data/Sypplementary_data_3_(SGA_heatmaps)/SGA_drug_combo_plate_7_ps1_A_96_T_25.png]

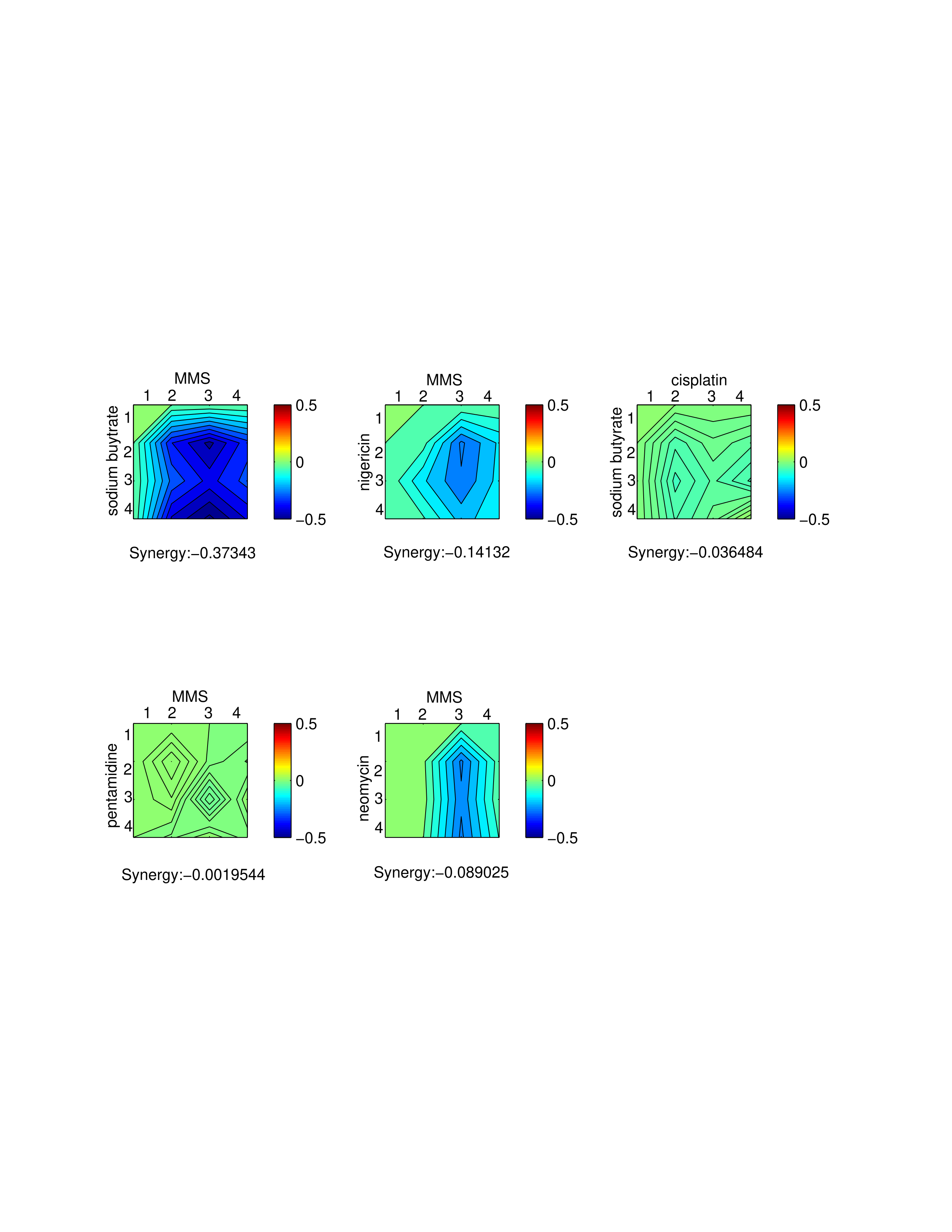

Supplement: Supplementary file 1 [file Data_Sheet_1.ZIP › Supplementary data/Sypplementary_data_3_(SGA_heatmaps)/SGA_drug_combo_plate_3_ps1_A_96_T_22.png]

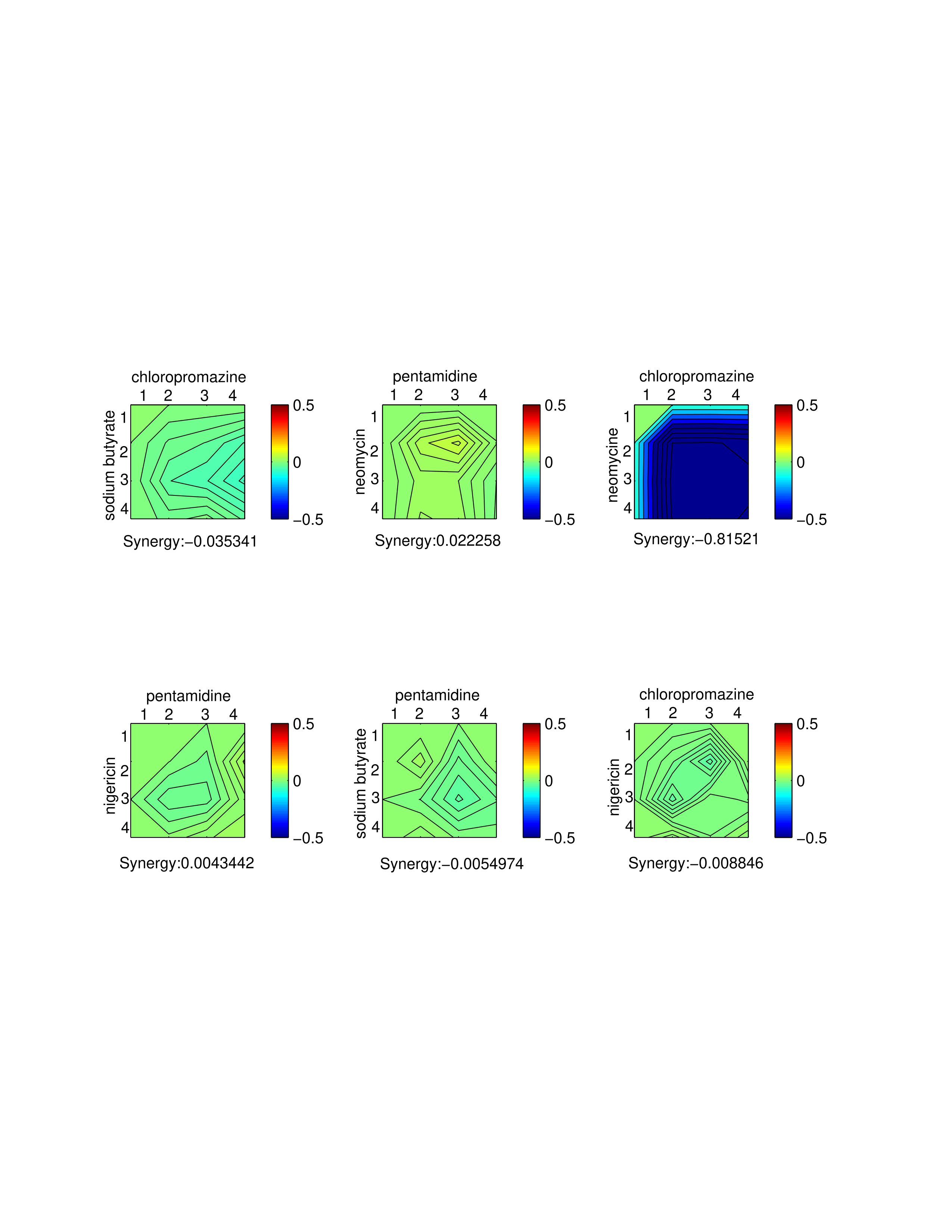

Supplement: Supplementary file 1 [file Data_Sheet_1.ZIP › Supplementary data/Sypplementary_data_3_(SGA_heatmaps)/SGA_drug_combo_plate_6_ps1_A_96_T_21.png]

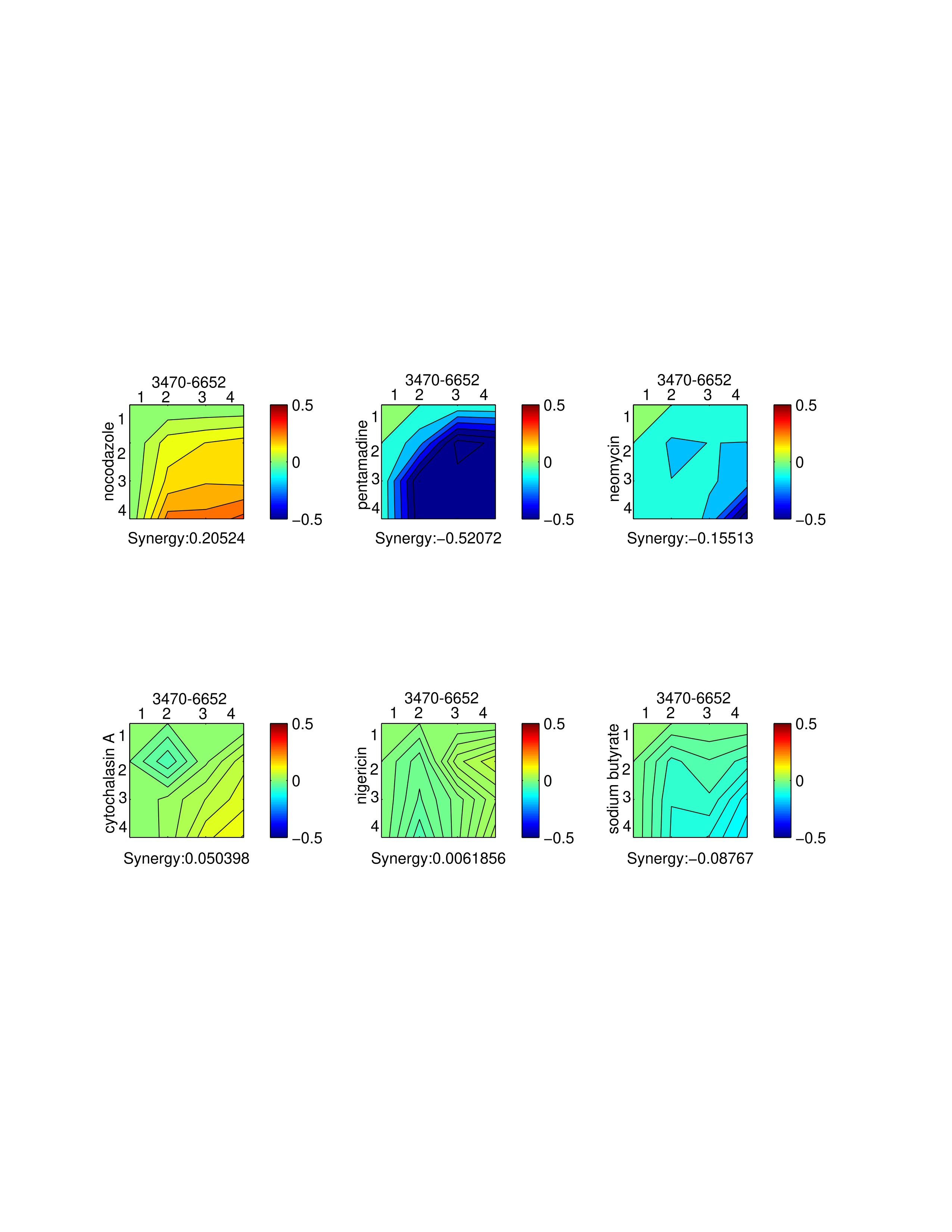

Supplement: Supplementary file 1 [file Data_Sheet_1.ZIP › Supplementary data/Sypplementary_data_3_(SGA_heatmaps)/SGA_drug_combo_plate_18_ps1_A_96_T_23.png]

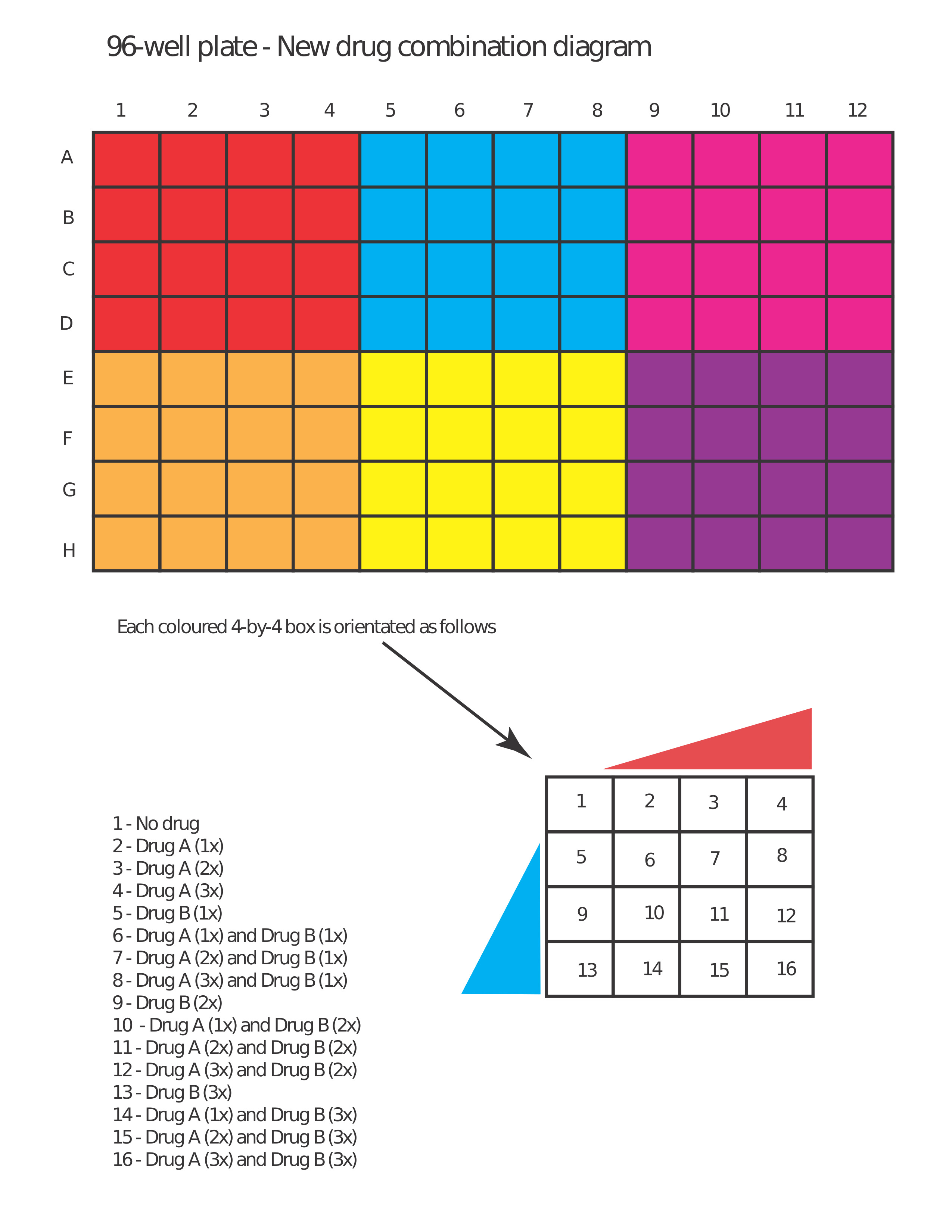

Supplement: Supplementary file 1 [file Data_Sheet_1.ZIP › Supplementary data/Sypplementary_data_3_(SGA_heatmaps)/Chemical-Combinations-SGA.jpg]

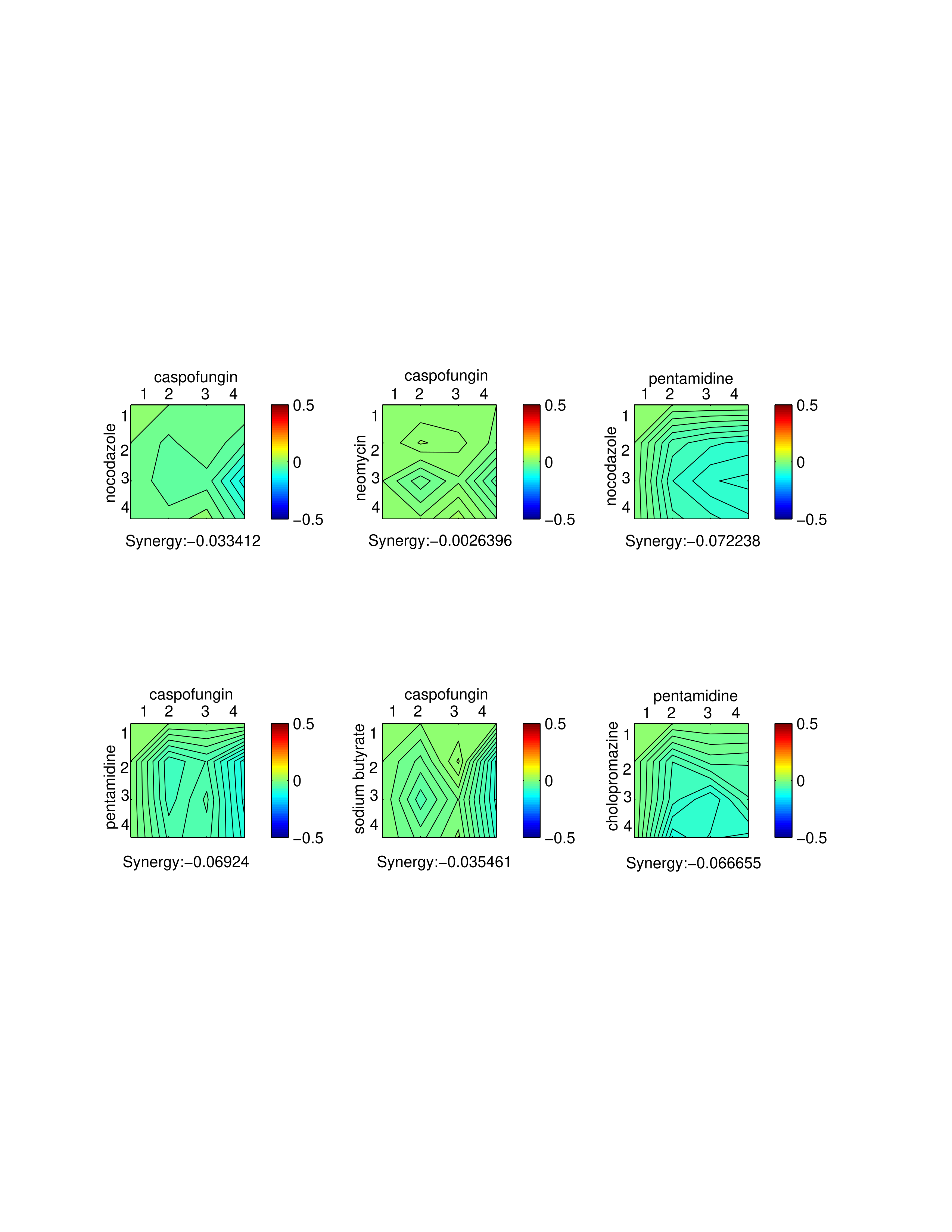

Supplement: Supplementary file 1 [file Data_Sheet_1.ZIP › Supplementary data/Sypplementary_data_3_(SGA_heatmaps)/SGA_drug_combo_plate_4_ps1_A_96_T_20.png]

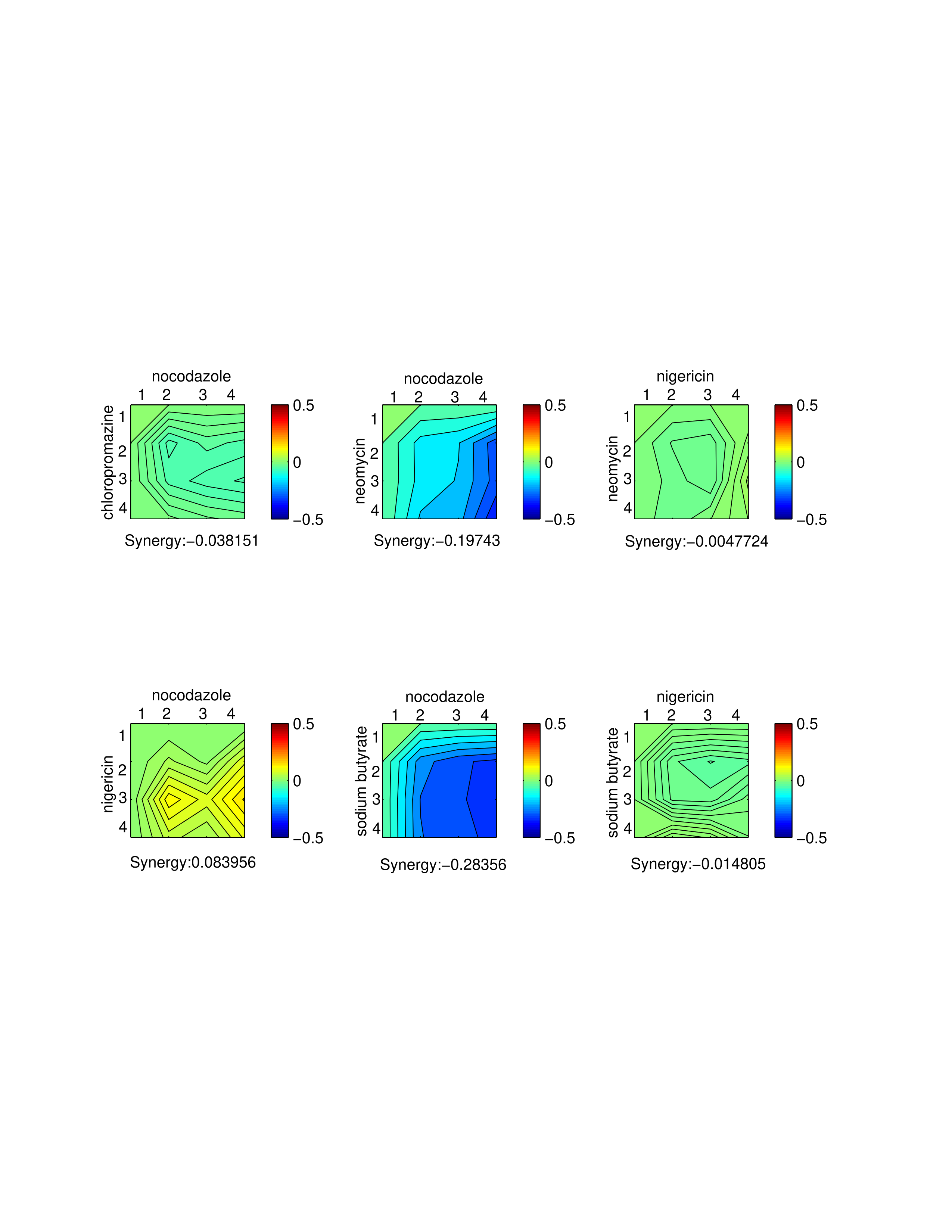

Supplement: Supplementary file 1 [file Data_Sheet_1.ZIP › Supplementary data/Sypplementary_data_3_(SGA_heatmaps)/SGA_drug_combo_plate_5_ps1_A_96_T_23.png]

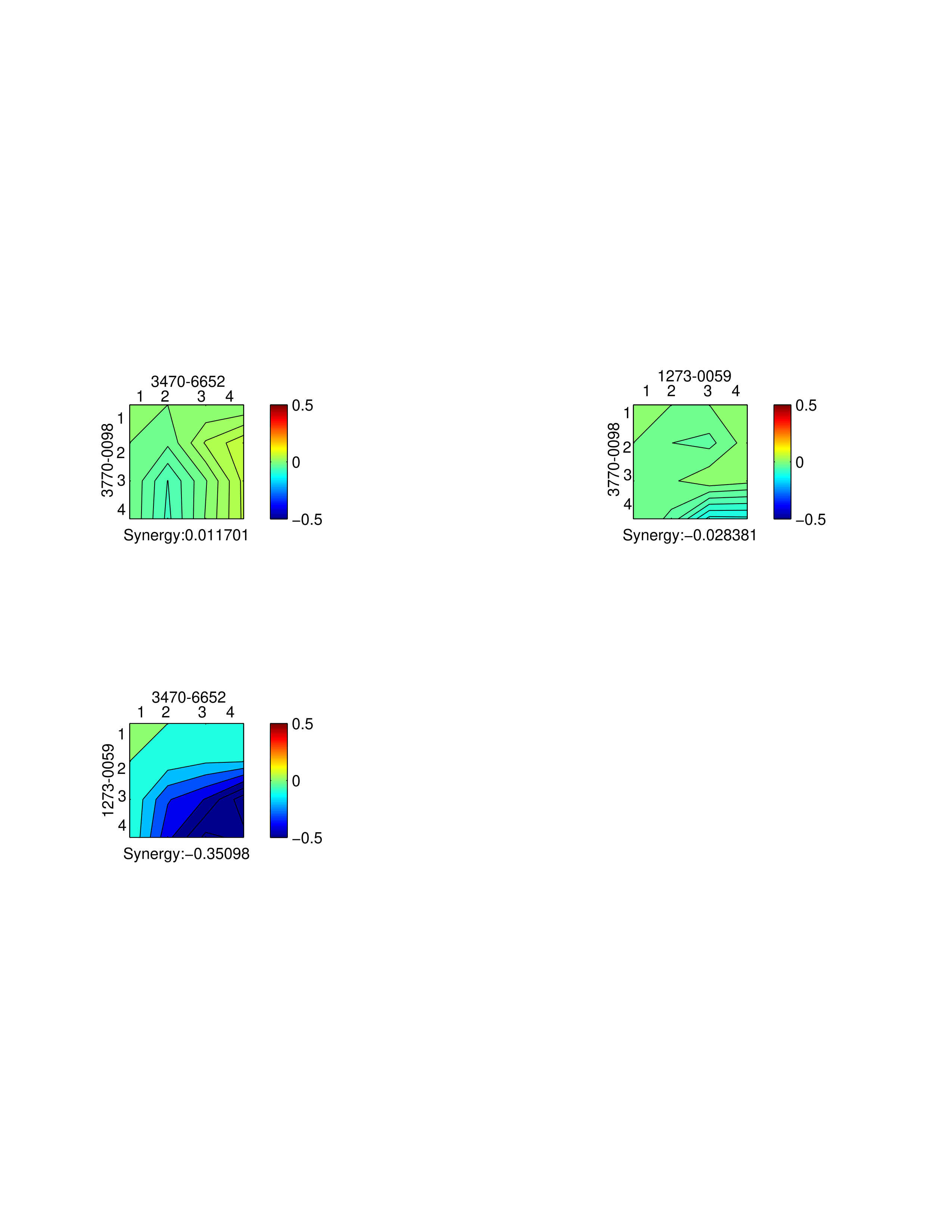

Supplement: Supplementary file 1 [file Data_Sheet_1.ZIP › Supplementary data/Sypplementary_data_3_(SGA_heatmaps)/SGA_drug_combo_plate_19_ps1_A_96_T_21.png]

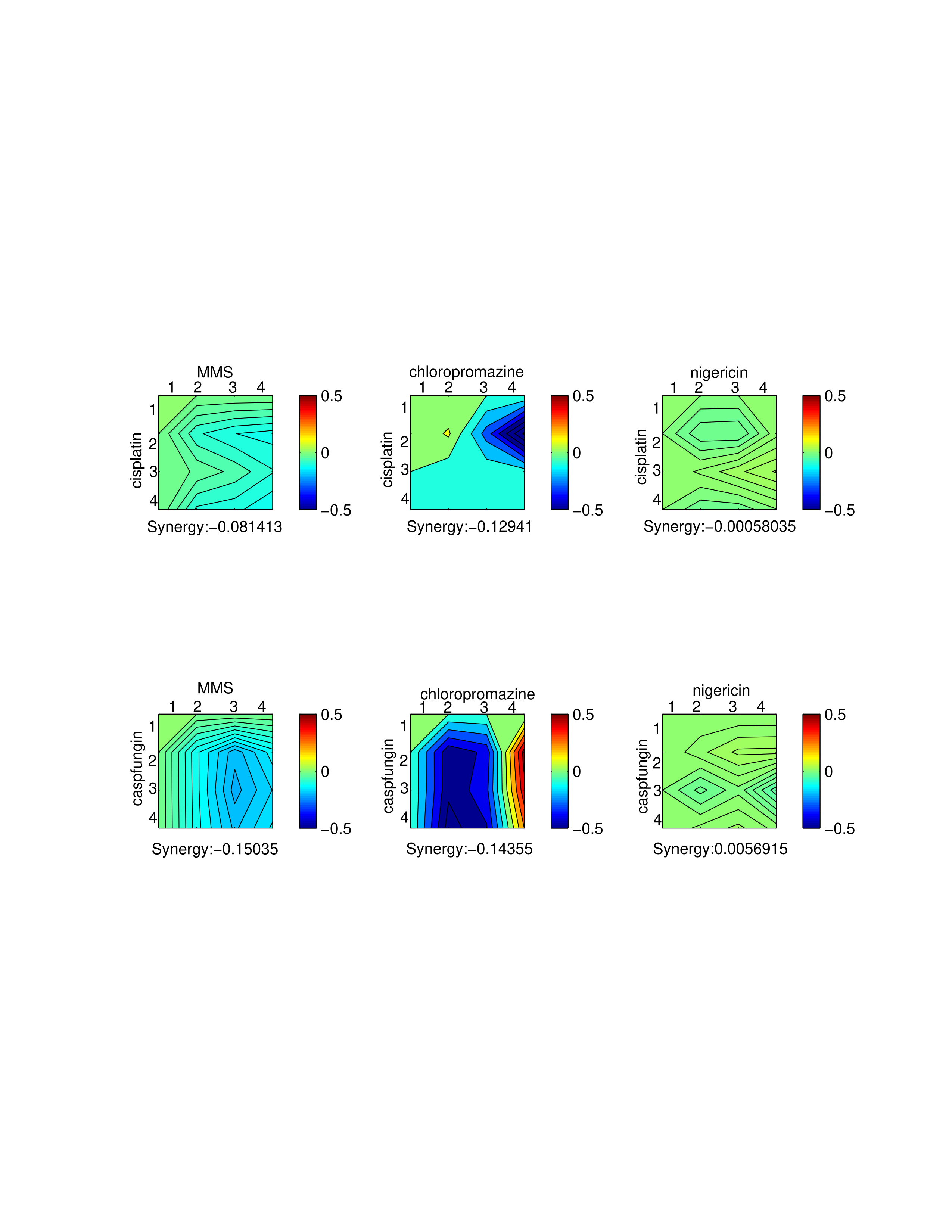

Supplement: Supplementary file 1 [file Data_Sheet_1.ZIP › Supplementary data/Sypplementary_data_3_(SGA_heatmaps)/SGA_drug_combo_plate_1_ps1_A_96_T_19.png]

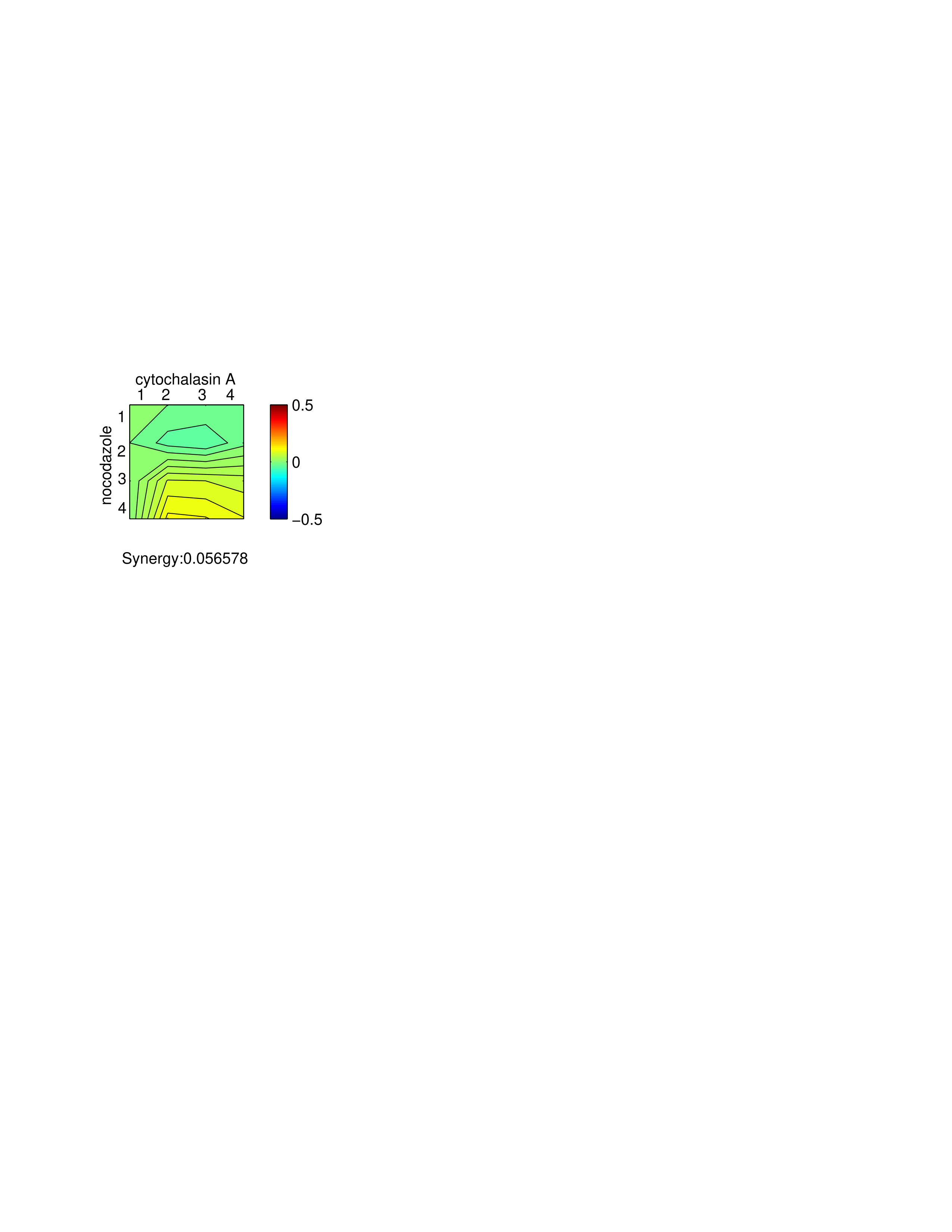

Supplement: Supplementary file 1 [file Data_Sheet_1.ZIP › Supplementary data/Sypplementary_data_3_(SGA_heatmaps)/SGA_drug_combo_plate_10_ps1_A_96_T_21.png]

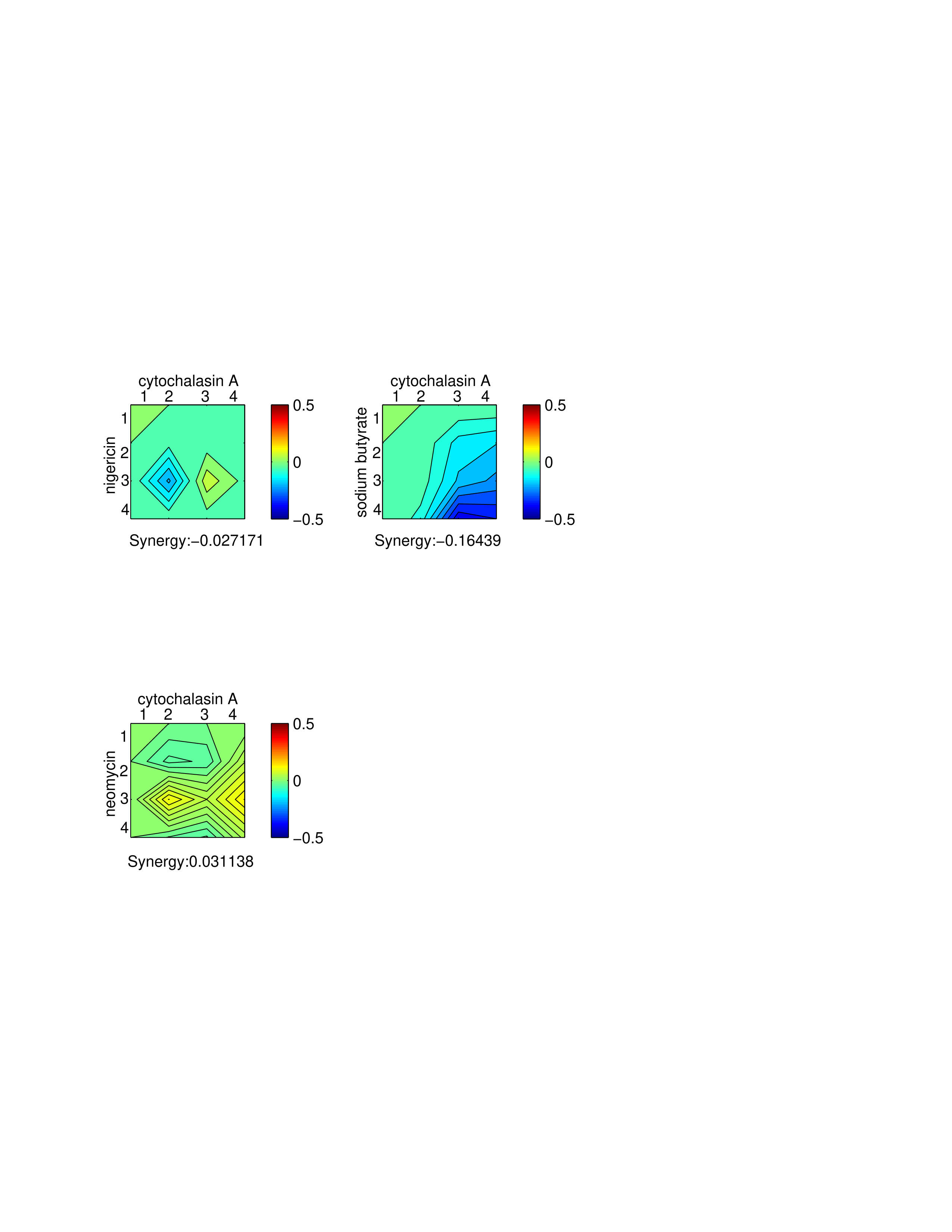

Supplement: Supplementary file 1 [file Data_Sheet_1.ZIP › Supplementary data/Sypplementary_data_3_(SGA_heatmaps)/SGA_drug_combo_plate_9_ps1_A_96_T_23.png]

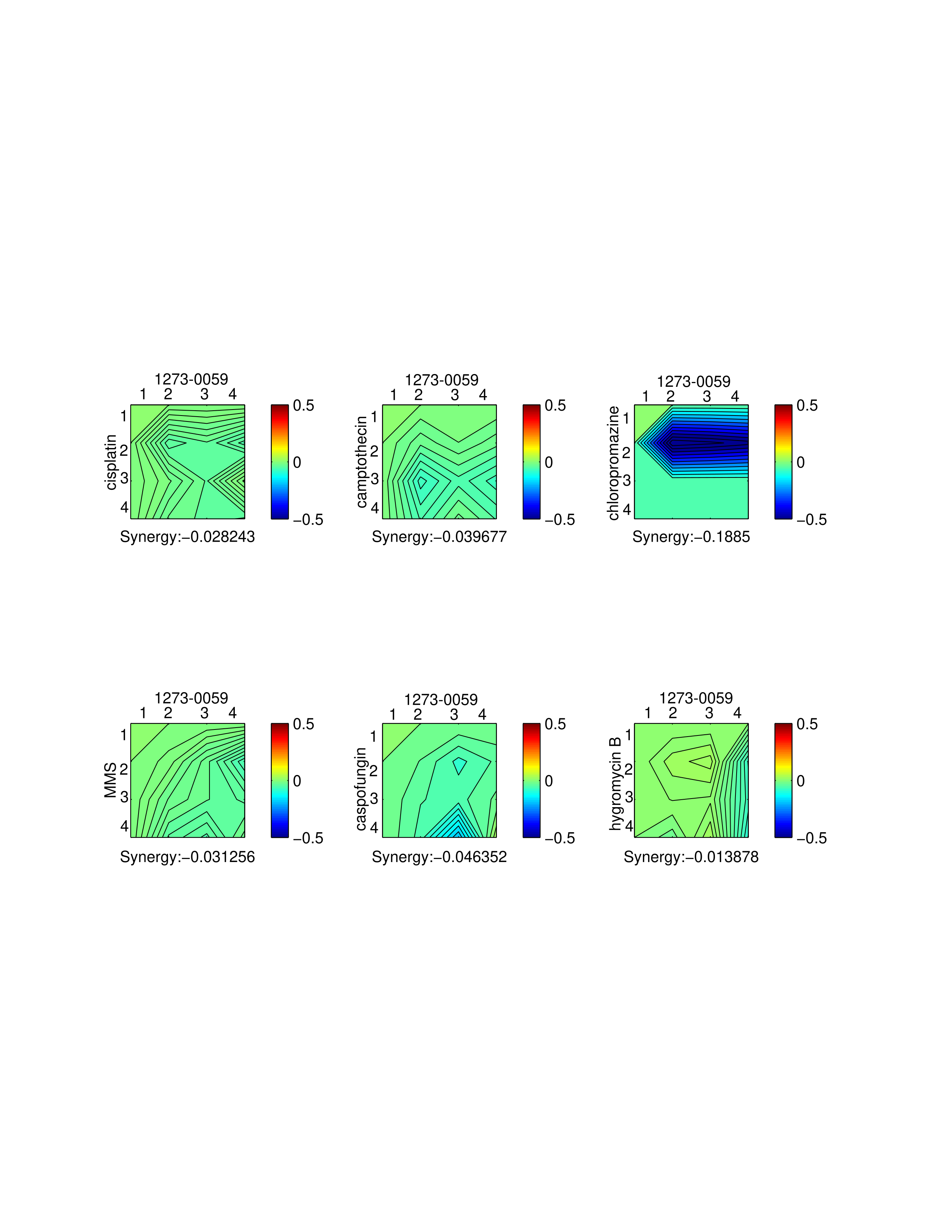

Supplement: Supplementary file 1 [file Data_Sheet_1.ZIP › Supplementary data/Sypplementary_data_3_(SGA_heatmaps)/SGA_drug_combo_plate_20_ps1_A_96_T_18.png]

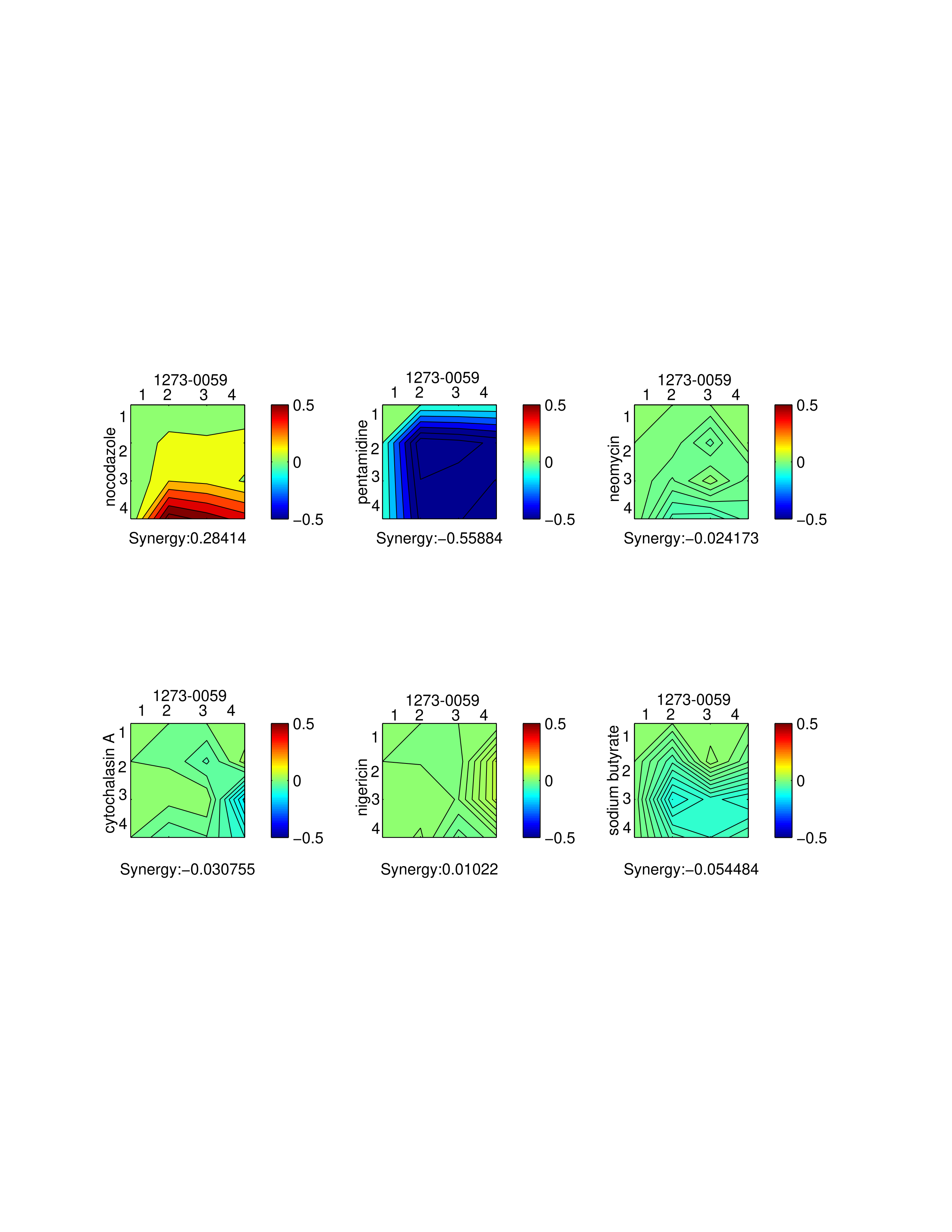

Supplement: Supplementary file 1 [file Data_Sheet_1.ZIP › Supplementary data/Sypplementary_data_3_(SGA_heatmaps)/SGA_drug_combo_plate_21_ps1_A_96_T_20.png]

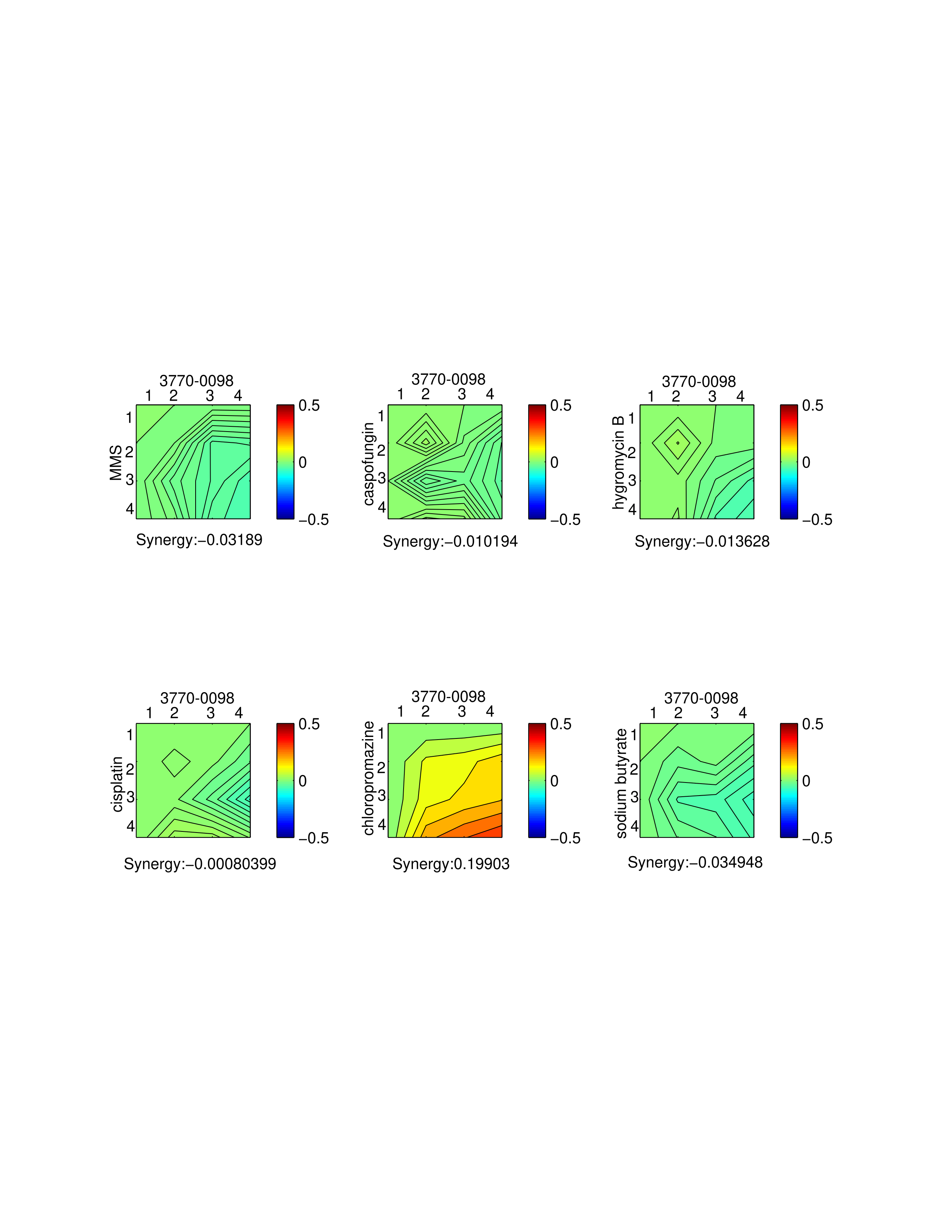

Supplement: Supplementary file 1 [file Data_Sheet_1.ZIP › Supplementary data/Sypplementary_data_3_(SGA_heatmaps)/SGA_drug_combo_plate_15_ps1_A_96_T_21.png]

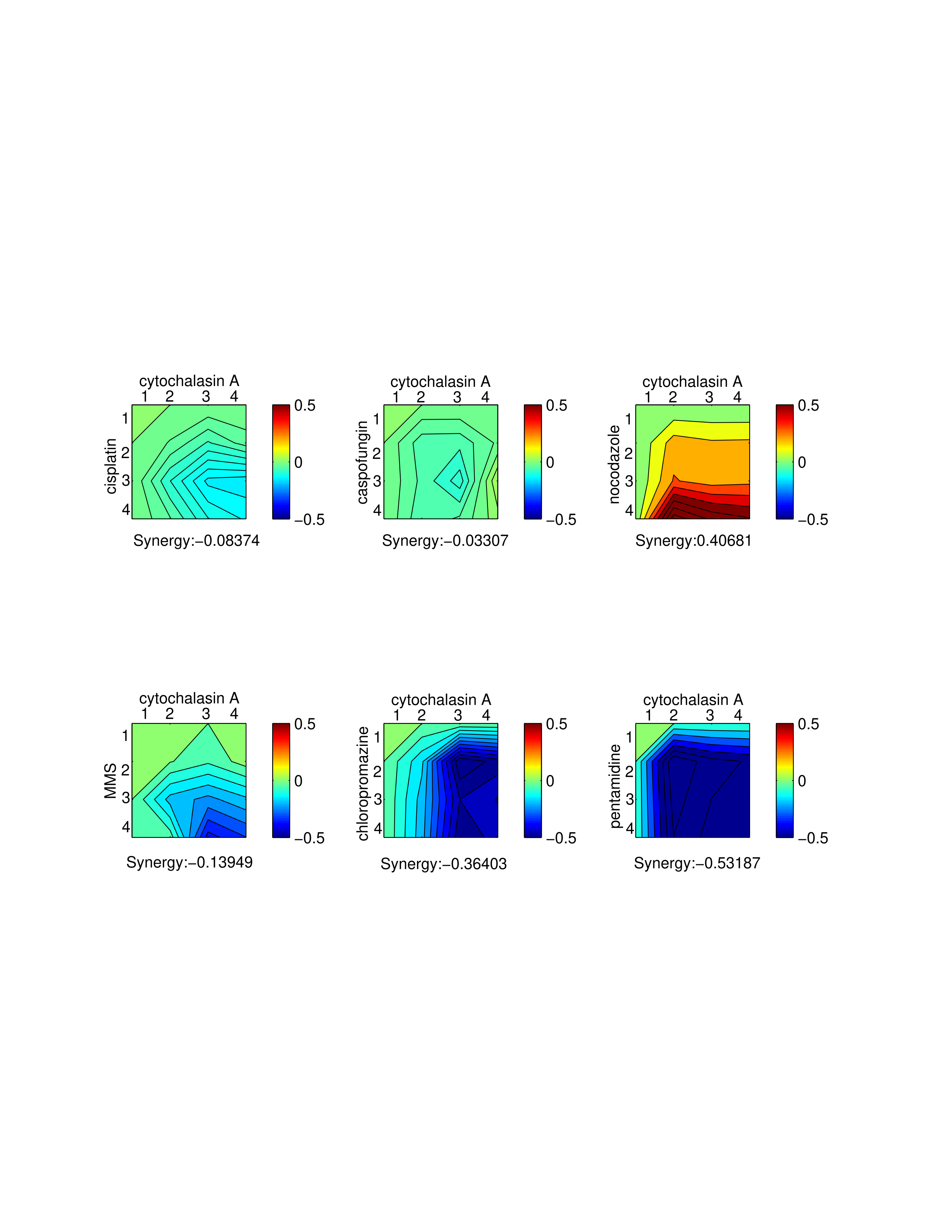

Supplement: Supplementary file 1 [file Data_Sheet_1.ZIP › Supplementary data/Sypplementary_data_3_(SGA_heatmaps)/SGA_drug_combo_plate_8_ps1_A_96_T_22.png]

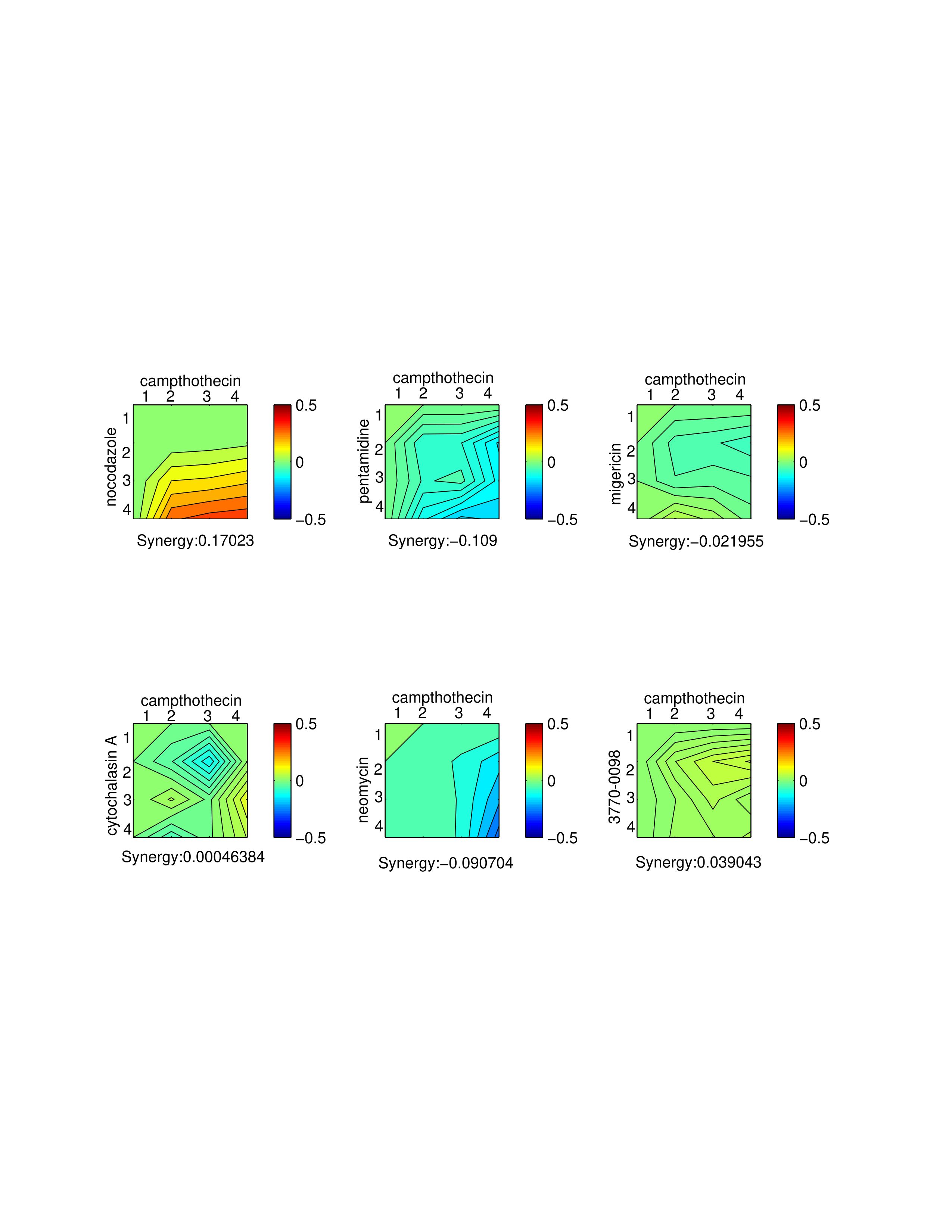

Supplement: Supplementary file 1 [file Data_Sheet_1.ZIP › Supplementary data/Sypplementary_data_3_(SGA_heatmaps)/SGA_drug_combo_plate_14_ps1_A_96_T_23.png]

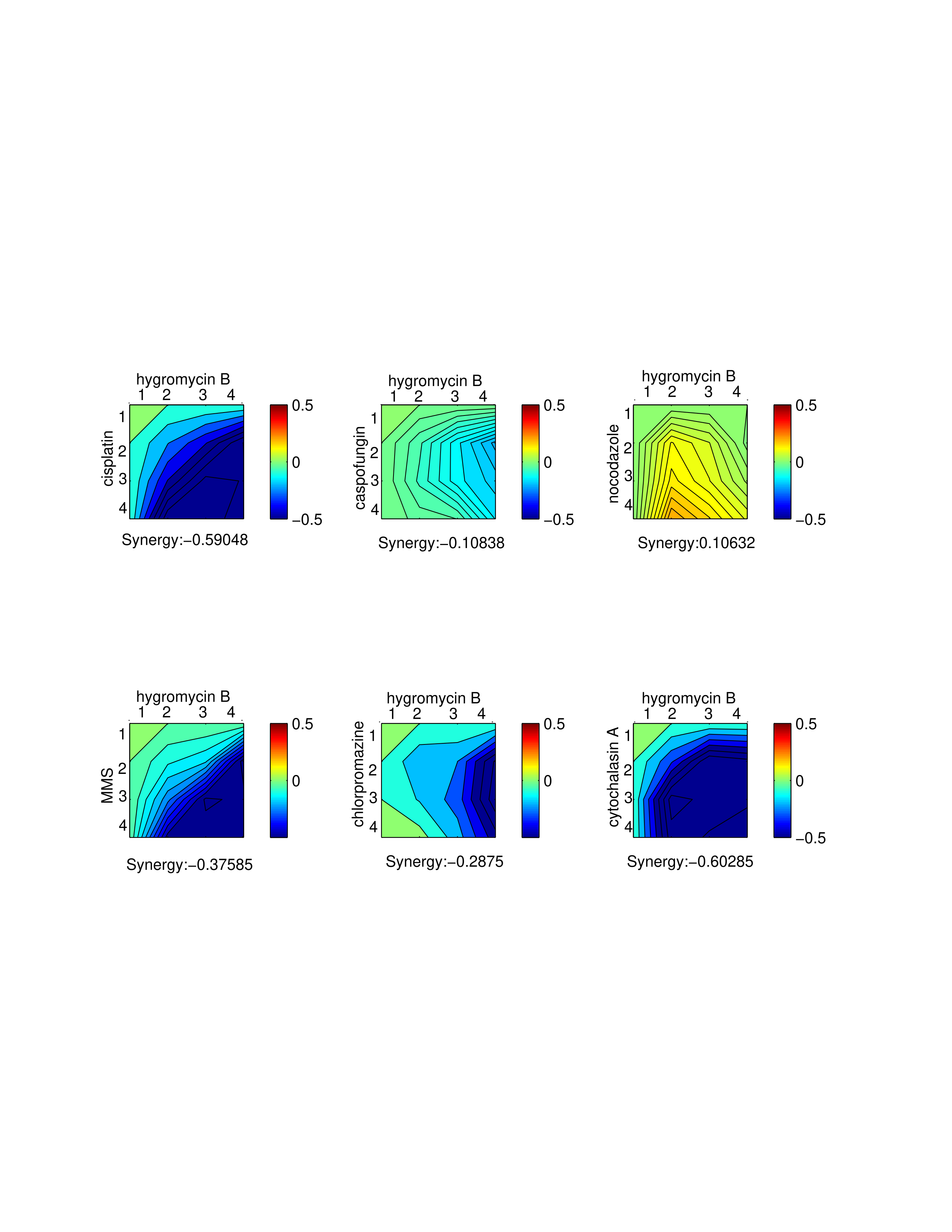

Supplement: Supplementary file 1 [file Data_Sheet_1.ZIP › Supplementary data/Sypplementary_data_3_(SGA_heatmaps)/SGA_Combo_Plate11_ps1_A_96_T_22.png]

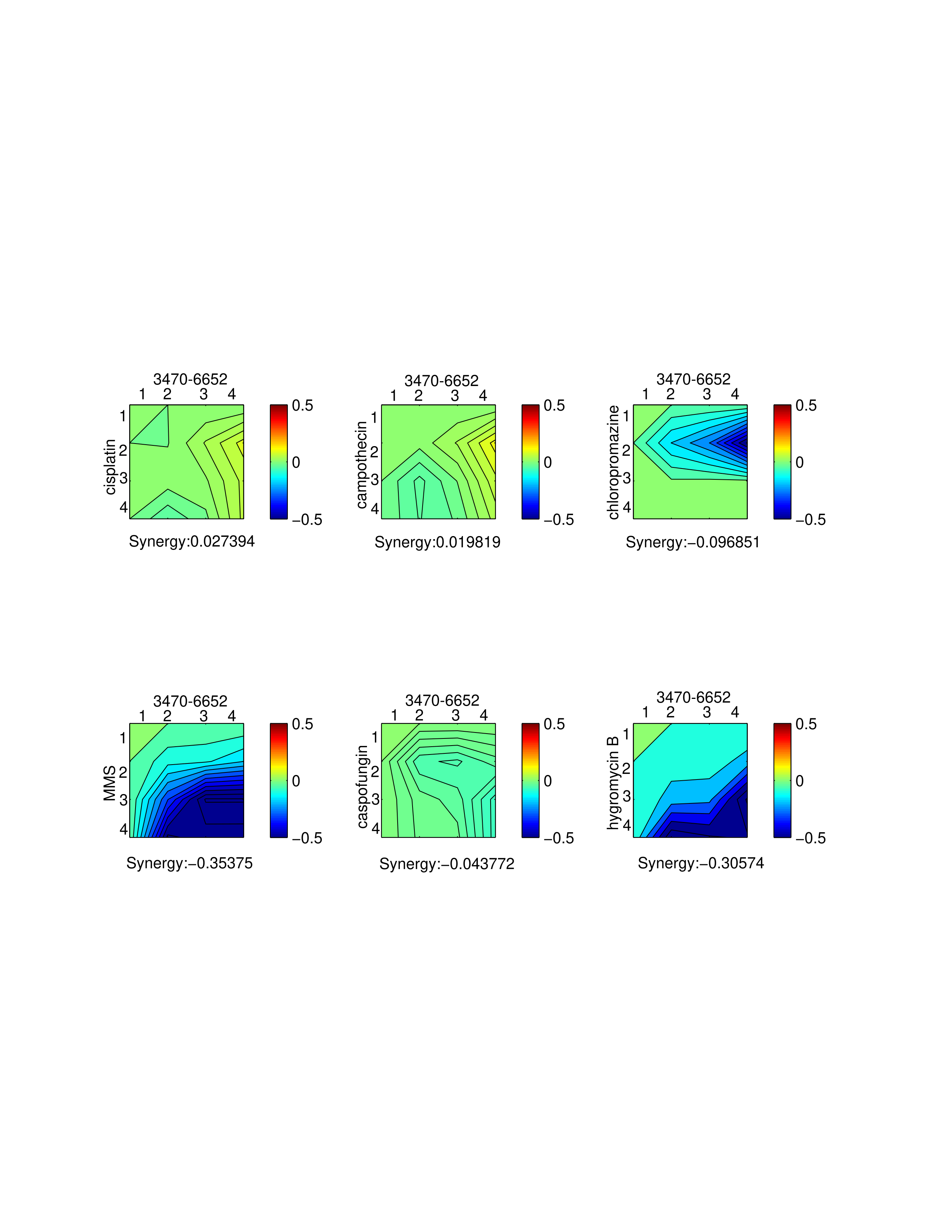

Supplement: Supplementary file 1 [file Data_Sheet_1.ZIP › Supplementary data/Sypplementary_data_3_(SGA_heatmaps)/SGA_drug_combo_plate_17_ps1_A_96_T_22.png]

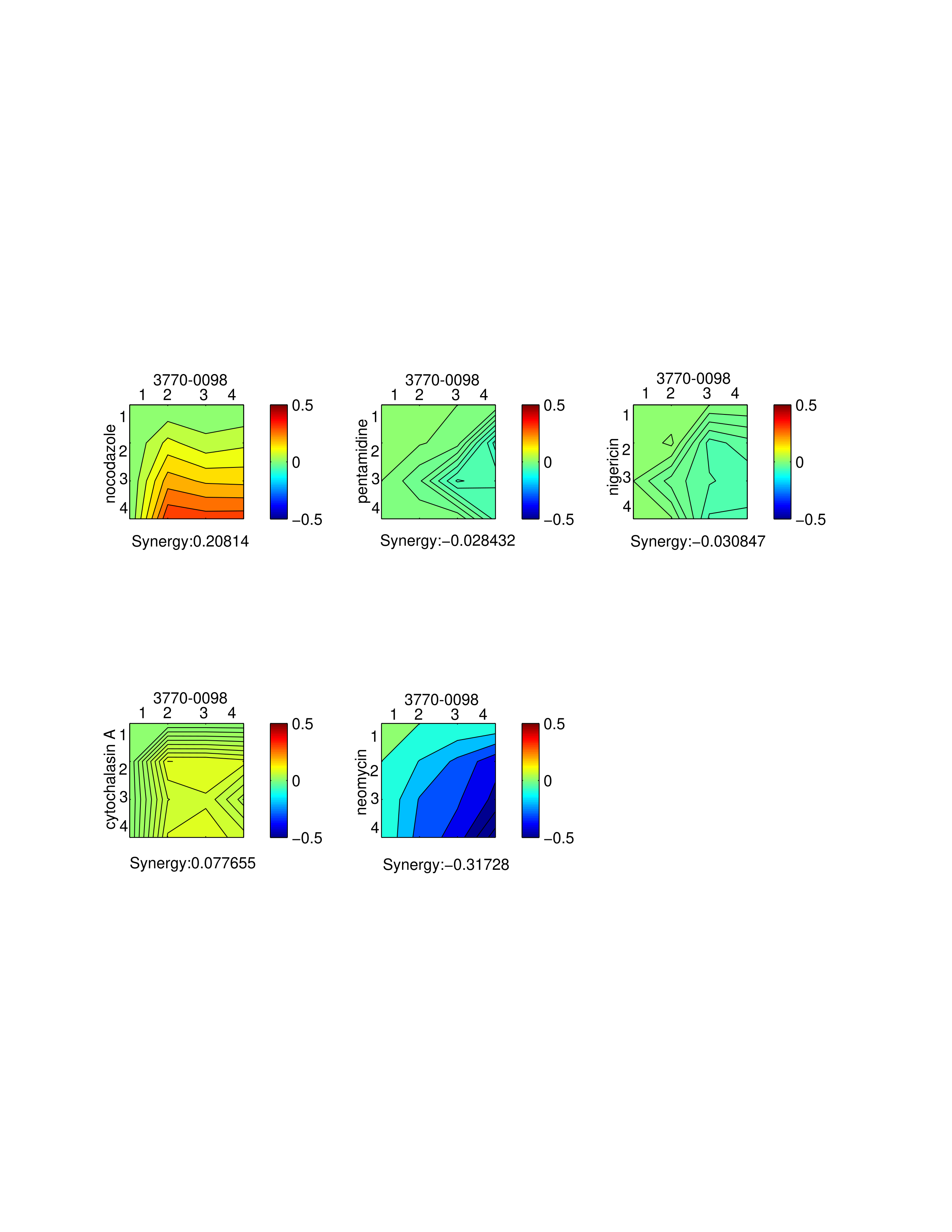

Supplement: Supplementary file 1 [file Data_Sheet_1.ZIP › Supplementary data/Sypplementary_data_3_(SGA_heatmaps)/SGA_drug_combo_plate_16_ps1_A_96_T_18.png]

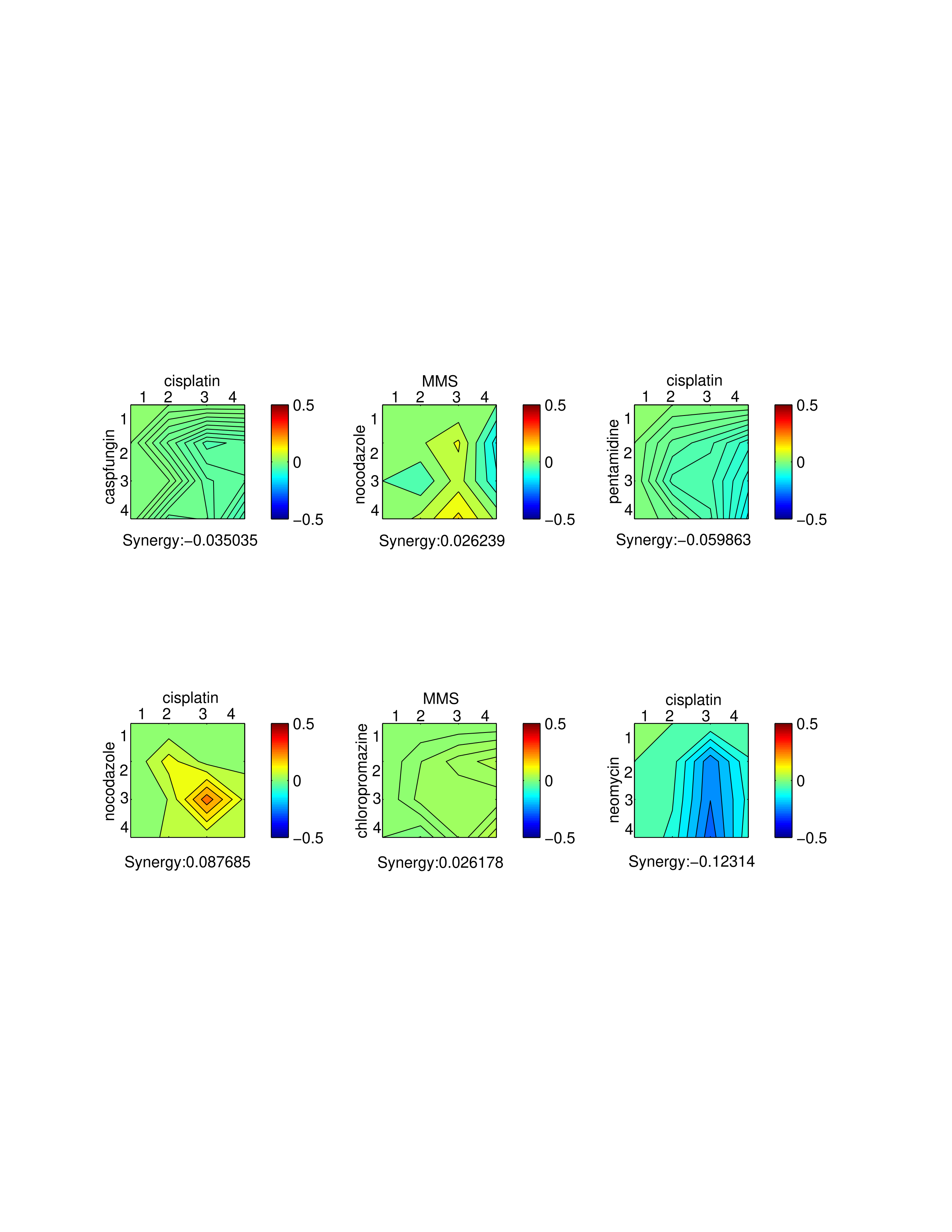

Supplement: Supplementary file 1 [file Data_Sheet_1.ZIP › Supplementary data/Sypplementary_data_3_(SGA_heatmaps)/SGA_drug_combo_plate_2_ps1_A_96_T_10.png]

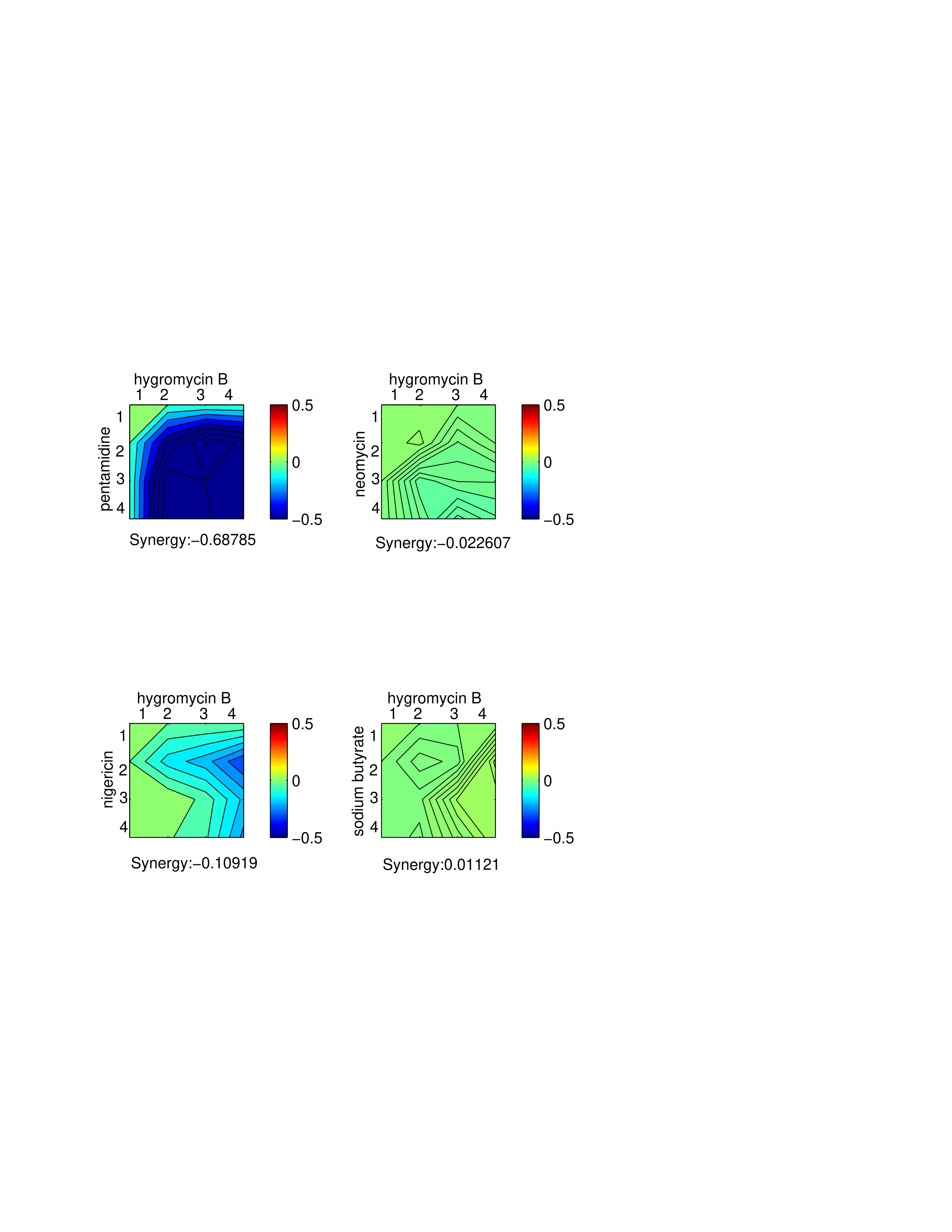

Supplement: Supplementary file 1 [file Data_Sheet_1.ZIP › Supplementary data/Sypplementary_data_3_(SGA_heatmaps)/SGA_drug_combo_plate_12_ps1_A_96_T_23.png]

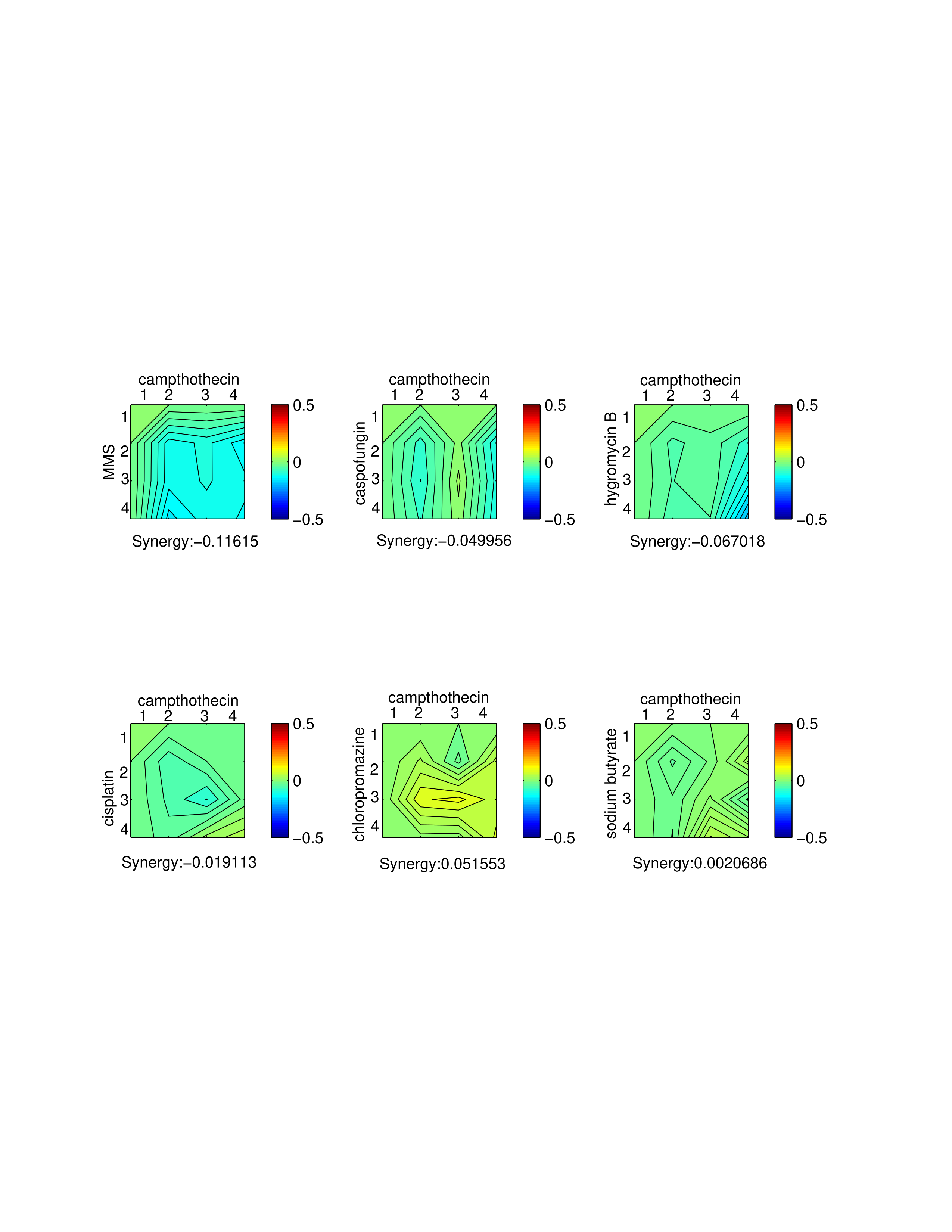

Supplement: Supplementary file 1 [file Data_Sheet_1.ZIP › Supplementary data/Sypplementary_data_3_(SGA_heatmaps)/SGA_drug_combo_plate_13_ps1_A_96_T_22.png]
