# Supplementary material for: Systematic Prediction of Antifungal Drug Synergy by Chemogenomic Screening in Saccharomyces cerevisiae
Source: Front Fungal Biol. 2021 Jul 2;2:683414. doi: 10.3389/ffunb.2021.683414 (PMC10512392; doi:10.3389/ffunb.2021.683414)
Supplement: Supplementary file 2 [file Data_Sheet_2.ZIP › Sypplementary_data_4_(Single_compounds)/0 Read me.rtfd/TXT.rtf]

In each excel file, spreadsheet C2-unique shows strains from the deletion library that show sensitivity to the drug combination. Among them, we only keep the strains showing ≥ inhibition (Z_score) to the combination while the Z_score for each single compound is less than 2. There are referred to as combination-specific strains in the manuscript.
