# Supplementary material for: Systematic Prediction of Antifungal Drug Synergy by Chemogenomic Screening in Saccharomyces cerevisiae
Source: Front Fungal Biol. 2021 Jul 2;2:683414. doi: 10.3389/ffunb.2021.683414 (PMC10512392; doi:10.3389/ffunb.2021.683414)
Supplement: Supplementary file 3 [file Data_Sheet_3.ZIP › Sypplementary_data_5_(HIP_HOP_assays)/0 Read me.rtf]

Read me	
	
Tables contain genome-wide HIP-HOP assays. Each file can be uploaded in our shiny app are it will generate the HIP and HOP plots.	
	
	
The shiny app address:	
https://ggshiny.shinyapps.io/GOappCN/	
	
In the match column, the value is 1 only for combination-specific strains.	
